# Wavy Whiskers in Wakes: Explaining the Trail‐Tracking Capabilities of Whisker Arrays on Seal Muzzles

**DOI:** 10.1002/advs.202203062

**Published:** 2022-11-20

**Authors:** Xingwen Zheng, Amar M. Kamat, Ming Cao, Ajay Giri Prakash Kottapalli

**Affiliations:** ^1^ Discrete Technology and Production Automation Group Engineering and Technology Institute Groningen Faculty of Science and Engineering University of Groningen Groningen 9747AG The Netherlands; ^2^ Advanced Production Engineering Group Engineering and Technology Institute Groningen Faculty of Science and Engineering University of Groningen Groningen 9747AG The Netherlands; ^3^ MIT Sea Grant College Program Massachusetts Institute of Technology 77 Massachusetts Avenue Cambridge MA 02139 USA

**Keywords:** flow–structure interaction, microelectromechanical system sensor, seal whisker, vortex‐induced vibration, wake‐induced vibration

## Abstract

Seals can detect prey up to 180 m away using only their flow‐sensing whiskers. The unique undulating morphology of Phocid seal whiskers reduces vortex‐induced vibrations (VIVs), rendering seals highly sensitive to biologically relevant flow stimuli. In this work, digital models of harbor and grey seal whiskers are extracted using 3D scanning and a mathematical framework that accurately recreates their undulating geometry is proposed. Through fluid–structure interaction studies and experimental investigations involving a whisker array mounted on 3D‐printed microelectromechanical systems sensors, the vibration characteristics of the whisker array and the interaction between neighboring whiskers in steady flows and fish‐wake‐like vortices are explained for the first time. Results reveal that the downstream vortices intensity and resulting VIVs are consistently lower for grey than harbor seal whiskers and a smooth cylinder, suggesting that the grey seal whisker geometry can be an ideal template for the biomimetic design of VIV‐resistant underwater structures. In addition, neighboring whiskers in an array influence one another by resulting in greater flow vorticity fluctuation and distribution area, thus causing increased vibrations than an isolated whisker, which indicates the possibility of a signal‐strengthening effect in whisker arrays.

## Graphical abstract

It is known that seals can detect prey up to 180 m away using their flow‐sensing whiskers, which feature undulating morphologies that reduce vortex‐induced vibrations. In this work, through fluidstructure interaction studies and experimental investigations involving seal whisker arrays mounted on 3D‐printed microelectromechanical systems sensors, the vibration characteristics of the whisker array and the interaction between neighboring whiskers in steady flows and fish‐wake‐like vortices are explained.

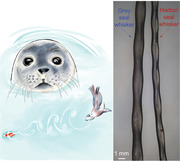

## 1 Introduction

**Figure 1:**
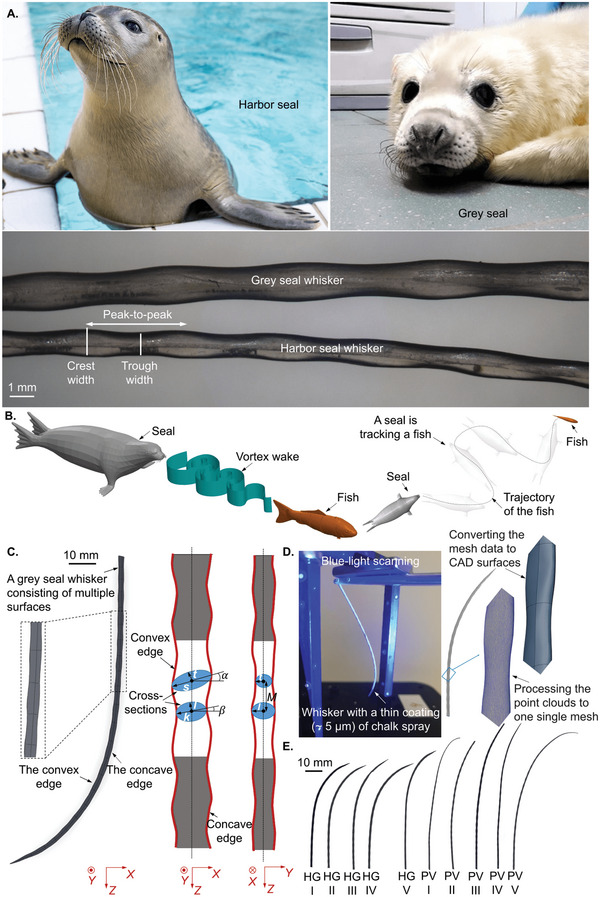
The unique undulating surface structure of the seal whisker enables ultrasensitive wake‐tacking capability. A) Harbor seal (*Phoca vitulina*), grey seal (*Halichoerus grypus*), and their whiskers’ close‐ups show the unique undulating morphology with parameters including crest width, trough width, and peak‐to‐peak distance. Seal images were provided by the Zeehondencentrum, Pieterburen, The Netherlands, and are reproduced with permission. B) A diagrammatic sketch showing the fish trail tracking capability of the seal whisker. C) An existing framework^[^ ^3^ ^]^ for describing seal whisker morphology. The convex and concave edges are defined when considering the semiplanes delimited by the edges. The convex and concave edges (looking at a whisker along the *Y*‐axis) contain peaks and troughs along the length. The oblique cross‐section between a peak of the convex edge and an adjacent peak of the concave edge forms an ellipse oriented at an angle *α* to the whisker axis. By contrast, the cross‐section between a convex edge trough and an adjacent concave edge trough forms an ellipse oriented at an angle *β* to the whisker axis. *s* and *t* denote the major and minor radii of the oblique ellipse joining the peaks, respectively. *k* and *l* denote the major and minor radii of the oblique ellipse joining the troughs, respectively. Finally, *M* denotes the distance between adjacent peaks and troughs of the cross‐sections (i.e., half the wavelength of the periodic undulations). D) Diagrammatic sketch to explain the blue light scanning technology. E) 2D images of all whiskers used for this study. Grey seal (*Halichoerus grypus*), HG, I–V and harbor seal (*Phoca vitulina*), PV, I–V. Scale bar: upper left. 3D CAD models are available in Data S1 of the Supporting Information as SOLIDWORKS SLDPRT files and 3D PDF files.

Most mammals, including pinnipeds (seals, sea lions, and walruses), rats, cats, and otters, have arrays of whiskers on their muzzle that serve as mechanosensors capable of sensing and interpreting flow or tactile information, thus creating situational awareness of the surrounding environment.^[^ ^1^ ^]^ Whiskers of some Phocid seal species, such as grey seals (*Halichoerus grypus*) and harbor seals (*Phoca vitulina*),^[^ ^2^ ^]^ feature unique undulating surface structures that resemble beads on a string (**Figure** **1**A).^[^ ^3^ ^]^ However, not all the seal species feature whiskers with such undulations. In an oncoming flow or when towed in still water, a high‐aspect‐ratio bluff body usually vibrates in the cross‐flow direction due to instabilities caused by the flow separation from the bluff body. The flow separation results in alternate vortices shed from either side of the bluff body, forming a Kármán vortex street in the wake of the bluff body. This vortex shedding generates periodic alternate loads on the bluff body transversely from both sides. If the structure's damping is low enough, the bluff body will vibrate due to the shedding vortices‐generated reaction, thus causing the vortex‐induced vibration (VIV).

Although the seal whisker is also a bluff body that experiences water flow, previous studies have shown that the seal whisker's unique undulating surface structure (Figure 1A) can suppress VIV. Using a camera mounted on a harbor seal's head, Hanke et al. observed no considerable whisker vibrations when the seal actively tracked a hydrodynamic trail.^[^ ^3^ ^]^ Furthermore, by comparing the whisker vibrations of the harbor seal (with undulations) and California sea lion (without undulations) via a piezoelectric sensor at the whisker base, they found that the California sea lion whisker vibrated 6× more than the harbor seal whisker.^[^ ^3^ ^]^ Since the Reynolds (*Re*) number was similar for the two whisker species, the different VIV responses were attributed to the harbor seal whisker's undulations, indicating the whisker's critical role in VIV suppression. Because of this capability, the undulating whiskers do not vibrate significantly as the seal swims forward during its hunting behavior, thus enhancing the whiskers’ sensitivity to minute flow disturbances such as those generated by an escaping prey. In this manner, the undulating whiskers attain a high signal‐to‐noise ratio and remain sensitive to biologically relevant signals, such as the vortex wake of an escaping fish (Figure 1B).^[^ ^4^, ^5^, ^6^, ^7^ ^]^ Experiments^[^ ^6^, ^7^ ^]^ conducted on live seals demonstrated that the harbor seal (*Phoca vitulina*) could use its whiskers to accurately track the hydrodynamic trajectory of a swimming fish robot up to 35 s after the fish passed.

The capacity of seal whiskers to suppress VIV has inspired several researchers to develop flow sensors that mimic their undulating geometry, resulting in low self‐generated noise.^[^ ^8^, ^9^, ^10^, ^11^, ^12^, ^13^, ^14^, ^15^, ^16^ ^]^ Seal whisker‐inspired structures have also shown promise for marine and aviation applications where VIV often contributes to structural fatigue and failure.^[^ ^17^, ^18^ ^]^ For example, the structure of offshore structures, such as underwater cables and oil platform bases, could be designed in the shape of a wavy cylinder to reduce vibrations. In addition, undulating power turbine blades inspired by the seal whisker can significantly reduce structural fatigue and drag by up to 50% in aero propulsion.^[^ ^18^ ^]^ Due to such vast biomimetic potential, research on seal whiskers’ geometrical characteristics and the VIV suppression capability has attracted increasing interest in recent years. A recent review^[^ ^19^ ^]^ summarizes seal whiskers’ flow‐sensing mechanisms and biomimetic potential.

As mentioned above, the seal whisker's undulating morphology contributes to the seal's hydrodynamic trail‐tracking capability. Therefore, to investigate the form‐function relationships of the seal whisker system, it is essential to characterize the whisker's undulating morphology. Furthermore, the biomimetic potential of the whisker can be realized by developing a geometric framework with mathematical formulations that can fully describe the morphological parameters of the seal whisker, using which a generalized whisker‐inspired structure can be constructed. In our previous work,^[^ ^20^ ^]^ various modified seal whisker structures highlighting each morphological parameter were first constructed according to the proposed geometrical framework. The influence of the morphological parameters on the VIV suppression abilities of these various modified whisker structures were then studied using flow–structure simulations. In this work, we conducted comprehensive 3D measurements of harbor and grey seal whiskers’ cross‐sectional geometric parameters in this work using blue light scanning. We proposed mathematical formulations of the seal whisker's undulating geometry. Furthermore, through fluid–structure interaction (FSI) studies and experimental investigations in a recirculating water flume featuring an array of seal whiskers on microelectromechanical systems (MEMS) sensors, the vibration characteristics of the harbor and grey seal whiskers were studied, and the mechanisms of VIV suppression were identified. The FSI study reported here presents a convenient alternative to experimental measurements due to the relative ease in calculating the whisker deformation and flow parameters compared to experimental approaches such as laser Doppler vibrometry and particle image velocimetry (PIV), which are challenging at the whisker's millimeter‐scale.^[^ ^21^, ^22^ ^]^ In addition to quantifying the VIVs, the FSI study enabled us to investigate the effect of whisker vibrations on the surrounding vortical structure, which has rarely been studied previously. Finally, 3D‐printed MEMS cantilever sensors featuring graphene nanomaterial piezoresistors were designed, fabricated, and employed to measure 3D‐printed whiskers’ self‐generated vortex‐induced vibrations (vortex‐induced vibrations, VIVs) and upstream fish‐wake‐like vortex‐induced vibrations (wake‐induced vibrations, WIVs), the latter of which was more similar to the actual scenario of fish trail tracking behavior of seal whiskers. However, how the seal whisker senses the fish trail has not been well studied. Beem & Triantafyllou^[^ ^11^ ^]^ measured the WIV of one single scaled‐up (20×) whisker structure and observed that the WIV response of undulating seal whiskers resulted from its “slaloming” movement to extract energy from an upstream vortex generator efficiently. Specifically, the seal whisker structure was first pulled to the closest oncoming vortex on one side because of the low‐pressure area associated with the vortex. Then it was pulled to the other side as the wake shed by the upstream circular cylinder progressed forward. The “slaloming” movement hypothesized that the seal whisker could sense the fish vortex wake by vibrating with a frequency locked to the dominant frequency of the wake. The WIV measurements of seal whiskers in an array supported this hypothesis and gained more insights into interactions of neighboring whiskers in the fish wake. Our key contributions are as follows.

i) Measurement and numerical framework formulation of complex 3D whisker morphology: Detailed geometric characterizations of the harbor (*Phoca vitulina*) and grey seal (*Halichoerus grypus*) whiskers were conducted, the latter of which has not been the subject of investigation before. Five harbor and five grey seal whiskers were scanned using high‐resolution blue light scanning technology. The morphological parameters of each cross‐section along the whisker length were determined through 3D measurements and then used to formulate numerical frameworks that were general enough to fully capture the 3D whisker geometry across at least two different Phocid seal species.
ii) FSI studies of scanned real‐scale seal whiskers: FSI studies were employed to study the VIV response of cylinders, including digital models of the harbor (*Phoca vitulina*) and grey seal (*Halichoerus grypus*) whiskers and one similar‐sized smooth circular cylinder. Notably, a comparative quantification of the VIV suppression capability across two different Phocid seal species (i.e., harbor and grey seals) is reported for the first time in this work. Our approach represents the first attempt to model the whisker deformation due to the oncoming flow and differs from prior studies in the literature that only modeled the flow field surrounding a seal whisker structure constructed using an idealized geometric framework, which neglected important geometric aspects such as the varying transverse (cross‐sectional) geometric parameters along the whisker length. The vorticity distributions, velocity fluctuations, enstrophy variations around the structures, and the tip displacements of the structures were compared qualitatively and quantitatively to reveal the interaction between the seal whisker's vibrations and the downstream flow field. This allowed us to study the VIV suppression mechanism of the undulating whiskers (compared to the smooth circular cylinder) in great detail. Finally, the FSI studies, for the first time, simulated the VIV response of an array of full‐length seal whiskers on the seal snout.
iii) VIV and WIV measurements of seal whiskers: The MEMS sensors were used for measuring the VIVs and WIVs of seal whiskers constructed using the proposed numerical framework as well as the scanned whiskers arranged in an array, thus 1) validating the VIV suppression capability of constructed whiskers, 2) explaining how the seal whisker interacts with fish wake‐like vortices to provide a mechanistic explanation for the ultrasensitive fish trail tracking ability of the seal whisker, and 3) gaining insights into how VIV responses of seal whiskers in an array on the seal muzzle could influence or be influenced by neighboring whiskers.

## 2 Results and Discussion

### 2.1 Morphometrics Using 3D Blue Light Scanning

Geometrically, the seal whisker has a tapered elliptical cylinder‐like structure with undulations, which causes the major and minor axes of the cross‐sectional ellipse to vary along the length.^[^ ^21^ ^]^ Furthermore, the cross‐sectional ellipse can change its orientation (to the longitudinal axis) along the whisker's length.^[^ ^21^ ^]^ Finally, the seal whisker shows a significant curvature (e.g., in the downstream direction for harbor and elephant seals and the cross‐flow direction for sea lions^[^ ^21^ ^]^). The three distinct geometrical aspects (undulations, taper, and curvature) mentioned above are illustrated in Figure 1C,D. Because of the unique nature of undulations, many researchers^[^ ^21^, ^23^, ^24^, ^25^ ^]^ have focused on quantifying and comparing morphological undulations for various seal species.

Early works^[^ ^3^, ^23^, ^24^ ^]^ reported 2D measurements of the seal whisker geometry by laying it flat on its major axis under an optical microscope. However, this method is time‐consuming because it requires repetitive measurements on multiple whiskers. In addition, 2D measurements fail to fully capture the 3D geometry of the whisker, including the varying angle of the major axis from horizontal along the length. Recently, techniques such as computed tomography (CT) scanning^[^ ^25^ ^]^ and laser scanning^[^ ^26^ ^]^ have been employed to conduct 3D measurements and obtain computer‐aided design (CAD) models of seal whiskers, which can be used for 3D measurements of morphological parameters. Morphological parameters, including the peak‐to‐peak distance along the convex edge and the concave edge of the whisker, the crest width, and the trough width (Figure 1A), were measured to report the seal whisker geometry. In this work, we used blue light scanning to capture the 3D geometry of seal whiskers (Figure 1D; Scans of Seal Whiskers section of the Experimental Section) and generated 3D CAD models from the scans (Figure 1E). All CAD models are available in Data S1 of the Supporting Information as SOLIDWORKS SLDPRT files and 3D PDF files.

**Figure 2:**
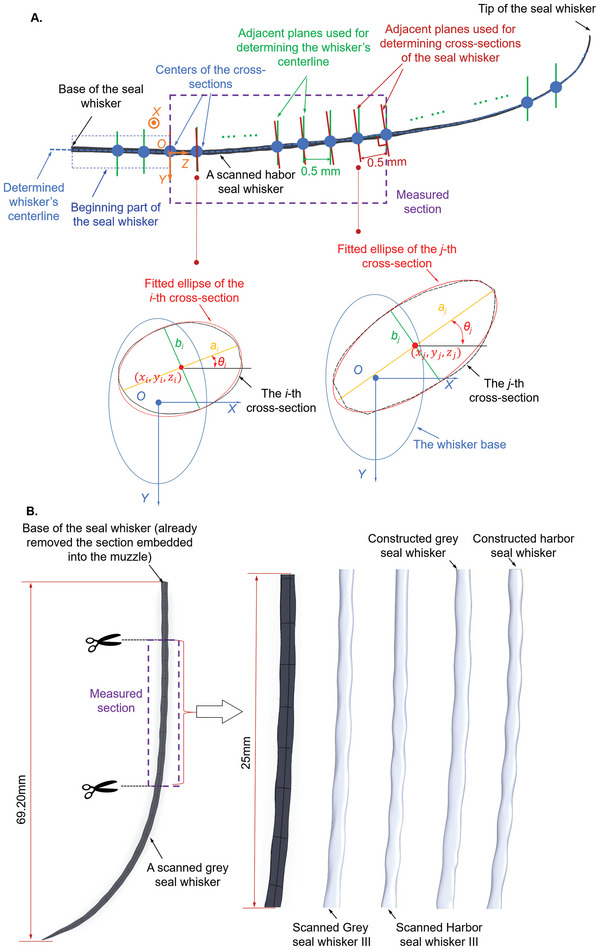
Collection and definitions of morphological parameters. A) Extraction of cross‐sectional morphological parameters (*a*, *b*, *θ*, *x*, *y*, *z*) to create the geometric framework. B) Cutting a segment of length 25 mm as the focal section for comparing constructed and scanned harbor and grey seal whiskers. 3D CAD models are available in Data S1 of the Supporting Information as SOLIDWORKS SLDPRT files and 3D PDF files. Zoomed‐in details of the undulating surfaces are presented in Movie S1 of the Supporting Information.

**Figure 3:**
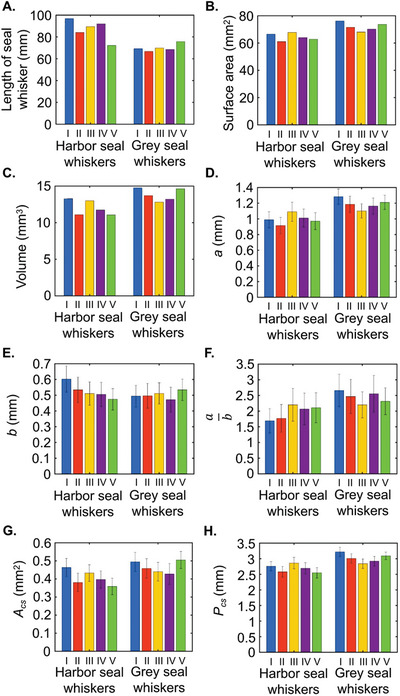
Parameterization of whisker geometries. A) Overall lengths of Grey seal whiskers I–V and Harbor seal whiskers I–V. B) Surface areas of 25 mm segments. C) Volumes of segments. D) Major axes (*a*) of whisker segments (averaged using values of 50 cross‐sections, with error bars indicating standard deviations). E) Minor axes (*b*) of whisker segments. F) Ratios ($\frac{a}{b}$) of major to minor axes. G) Cross‐sectional areas (*A*_cs_) of whisker segments. H) Perimeters (*P*_cs_) of whisker segment cross‐sections.

**Figure 4:**
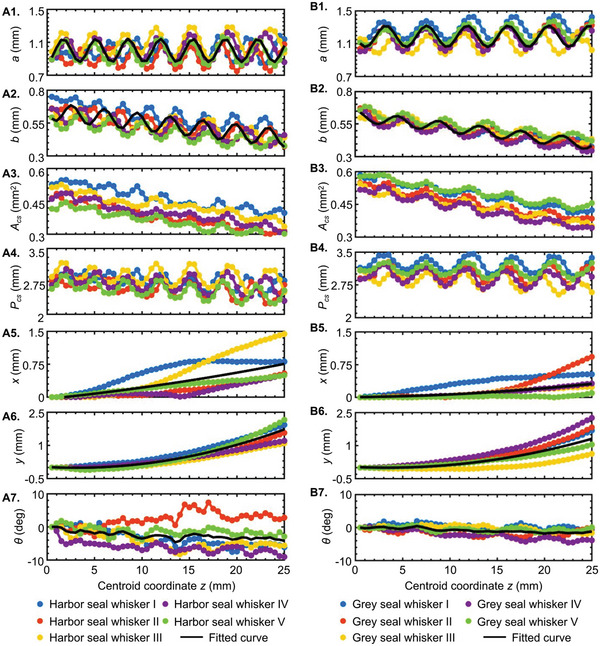
Cross‐sectional morphological parameters (*a*, *b*, *A*_cs_, *P*_cs_, *x*, *y*, *θ*) along the whisker length. A) Harbor seal whiskers. B) Grey seal whiskers.

Motivated by the unmet issues discussed above, we measured the cross‐sectional morphological parameters, including the major axis (*a*), minor axis (*b*), and cross‐section orientation (*θ*) to the horizontal and centroid coordinates (*x*, *y*, *z*) (**Figure** **2**A) of 50 scanned seal whiskers over 25 mm of their middle portions (see purple dotted box, Figure 2A,B). The detailed steps of these measurements are presented in Cross‐Sectional Morphological Parameter Measurements section of the Experimental Section. ImageJ was used to measure the morphological parameters above (*a*, *b*, *θ*, *x*, *y*, *z*), while the scanned whiskers’ overall length (Figure 3A), surface area (**Figure** **3**B), and volume (Figure 3C) of the 25 mm segment were calculated using the CAD software SOLIDWORKS (Dassault Systèmes). Except for the overall length measurement, all the other measurements were conducted on the 25 mm long segments. Harbor seal whiskers (86.9 ± 9.43 mm) were typically longer than grey seal whiskers (70.0 ± 3.39 mm) (Figure 3A). Within the 25 mm segment, we observed that harbor seal whiskers had a smaller average surface area (64.5 ± 2.73 mm^2^, Figure 3B) and volume (12.0 ± 1.06 mm^3^, Figure 3C) than those of grey seals (72.0 ± 3.13 mm^2^ and 13.8 ± 0.858 mm^3^). More importantly, harbor seal whiskers had a smaller average major axis (0.996 ± 0.0639 mm, Figure 3D) than the grey seal whiskers (1.19 ± 0.0663 mm, Figure 3D) but a similar average minor axis (0.502 ± 0.0481 mm, Figure 3E) (0.526 ± 0.0232 mm, Figure 3E). As a result, harbor seal whiskers had a smaller average ratio (1.91 ± 0.224, Figure 3F) of major to minor axes than grey seal whiskers (2.44 ± 0.184, Figure 3F). In addition, harbor seal whiskers had a smaller average cross‐sectional area (0.407 ± 0.0419 mm^2^, Figure 3G) and perimeter (2.69 ± 0.128 mm, Figure 3H) than grey seal whiskers (0.465 ± 0.0338 mm^2^ and 3.02 ± 0.147 mm). The above observations of geometric differences across two seal whisker species were valid for the chosen whiskers (available at the Seal Rehabilitation and Research Centre, Pieterburen, The Netherlands) and not necessarily true in general. Based on the measured cross‐sectional morphological parameters, including the major axis (*a*), minor axis (*b*), cross‐sectional orientation (*θ*), and centroid coordinates (*x* and *y*) along the whisker length (shown in **Figure** **4A,B**), the following observations were concluded.

i) The major axis *a* and the minor axis *b* featured sinusoidal variations along the whisker length (Figure 4A1,A2,B1,B2). *a* and *b* varied more smoothly, with a larger period, for grey seal whiskers than for those of harbor seals. The major axis a varied around an approximately constant mean for both species, varying from ≈0.9 to 1.5 mm for grey seals and from 0.7 to 1.3 mm for harbor seals. By contrast, the minor axis *b* varied around a linearly decreasing mean from whisker base to tip. As a result, the cross‐sectional area (Figure 4A3,B3) decreased along the length. Further inspection revealed that the major and minor axes appeared in the opposite phase. As the major axis reached its maximum value, the minor axis reached its minimum and vice versa, resulting in a roughly consistent cross‐sectional perimeter with only a minor decrease along the whisker length (Figure 4A4,B4).
ii) The *x*‐coordinate (Figure 4A5,B5) and *y*‐coordinate (Figure 4A6,B6) of the centroid of the whisker cross‐section appeared to increase gradually, meaning that the seal whisker gradually bent along the length and was not strictly straight. The characteristics mentioned above (taper and curvature) are expected to be important in VIV suppression but were largely neglected by existing descriptions^[^ ^3^ ^]^ of the seal whisker geometry.
iii) In all the cases of the harbor and grey seal whiskers, the variation in *θ* along the whisker length did not exhibit any specific trend or regularity (Figure 4A7,B7). It ranged from −10° to 10° for the harbor seal whisker's cross‐sections and from −5° to 2° for the grey seal whiskers.

Because the undulating morphology has not been observed in other whiskered mammals apart from some seal species such as grey seal (*Halichoerus grypus*) and harbor seal (*Phoca vitulina*), much attention was understandably paid to investigating the contributions of such undulations to the seal whisker's flow sensing capability, without much attention to a framework that can capture the phocid whisker geometry across various seal species. Therefore, the above comprehensive 3D measurement data of cross‐sectional morphological parameters for harbor (*Phoca vitulina*) and grey seal (*Halichoerus grypus*) whiskers (five from each species) are expected to be a valuable database for researchers. Based on such 3D measurements, a novel geometrical framework that allows a close representation of the seal whisker is proposed, and discussed below.

### 2.2 Mathematical Formulation of Harbor and Grey Seal Whiskers’ 3D Geometry

To obtain a geometrical framework, Equations (1)–(5) were used to fit *a*, *b*, *x*, *y*, and *θ* with the *z‐*coordinate along the length, for each whisker, thus determining the geometrical framework parameters *V_i_* (*i* = 1, 2, 3, …, 14, 15). The exact values of *V_i_* (*i* = 1, 2, 3, …, 14, 15) for each whisker can be found in Table S1 of the Supporting Information. Five values of *V_i_* (*i* = 1, 2, 3, …, 14, 15) were obtained for five harbor and five grey seal whiskers, with their average taken as the final geometrical framework parameter for each species’ whisker (Table S2, Supporting Information).

Since a sinusoidal variation was observed for *a* and *b*, sine functions (Equations (1) and (2)) were used to fit the plots of *a* and *b*. Variables *V*_1_ and *V*_5_ reflected amplitudes of the sine curves. *V*_1_ was similar for harbor (≈0.14, Table S2, Supporting Information) and grey seal whiskers (≈0.14, Table S2, Supporting Information) because the major axis *a* had a similar amplitude (≈0.06, Table S2, Supporting Information) between peaks and troughs. Variables *V*_2_ and *V*_6_ had similar values for both species (*V*_2_ ≈ *V*_6_ ≈ 1.8 for harbor seal and *V*_2_ ≈ *V*_6_ ≈ 1.4 for grey seal, Table S2, Supporting Information), meaning major and minor axis undulations had similar wavelengths along the whisker length

(1) $$a\left(z\right)=V_{1}*\sin\left(V_{2}*z+V_{3}\right)+V_{4}$$

(2) $$b\left(z\right)=V_{5}*\sin\left(V_{6}*z+V_{7}\right)+V_{8}*z+V_{9}$$

(3) $$x\left(z\right)=V_{10}*z^{2}+V_{11}*z+V_{12}$$

(4) $$y\left(z\right)=V_{13}*z^{2}+V_{14}*z+V_{15}$$

(5) $$\theta \left(z\right)=\frac{\sum_{i=0}^{5}\theta_{i,z}}{5}$$

In the case of the parameters *x* and *y*, a quadratic polynomial best fitted the plots’ average. However, *θ* did not exhibit regularity across the centroid coordinate *z* for various seal whiskers, so no mathematical function was found to describe *θ*. Thus, the general regularity of *θ* was characterized by the mean of the five values (*θ_i,z_*, *i* = 1, 2, …, 5, Data S2, Supporting Information) obtained from five seal whiskers, as in Equation (5). Therefore, it is worth investigating the mathematical formulations of the seal whisker's geometry, using which a generalized seal whisker structure with VIV resistance can be constructed for the various engineering applications mentioned above. However, a geometric framework consisting of mathematical formulations of the morphological parameters was not found in previous works^[^ ^3^, ^21^, ^23^, ^24^, ^25^ ^]^ for the seal whisker. Equations (1)–(5) are the first records of mathematical formulations of the seal whisker's 3D geometry to the best of our knowledge. The previously used geometrical framework^[^ ^3^ ^]^ resulted in an idealized structure, flat in one plane, with no curvature or taper along the whisker length. By contrast, the proposed mathematical formulations can generate a seal whisker structure that fully captures the 3D whisker geometry by including the varying transverse (cross‐sectional) geometric parameters along the whisker length. The transverse (cross‐sectional) geometric parameters described above include the varying major and minor axes (causing taper), the varying centroid coordinates of the cross‐sections (causing curvature), and the varying angle of the major axis from horizontal along the whisker length.

The parameters (*a, b*, *θ*, *x*, *y*, *z*) obtained from Equations (1)–(5) were used to construct generalized 3D CAD models of two whiskers using the “lofted base” option in SOLIDWORKS (25 mm, Figure 2B) with 50 cross‐sections (*z‐*coordinates of which varied with an interval of 0.5 mm). The CAD models are available in Data S1 of the Supporting Information as SOLIDWORKS SLDPRT files and 3D PDF files. Movie S1 of the Supporting Information shows zoomed‐in details of the undulating surfaces. To confirm the accuracy of the constructed seal whisker models, we compared the overall morphology, volume, surface area, major axis, and minor axis of the constructed seal whiskers’ undulations to those of the scanned real‐scale seal whiskers (Table S3, Supporting Information). Table S3 of the Supporting Information was obtained by averaging the five values from five scanned harbor (grey) seal whiskers. It can be seen from the measurements presented in Table S3 of the Supporting Information that the values of the volume, surface area, and average major and minor axes of the constructed whiskers matched well (error ≈ 0.11–15.7%) with those of both the scanned digital models of harbor and grey seal whiskers, thus verifying the accuracy of the proposed framework.

### 2.3 FSI Studies of 3D‐Scanned Harbor and Grey Seal Whisker Segments

**Figure 5:**
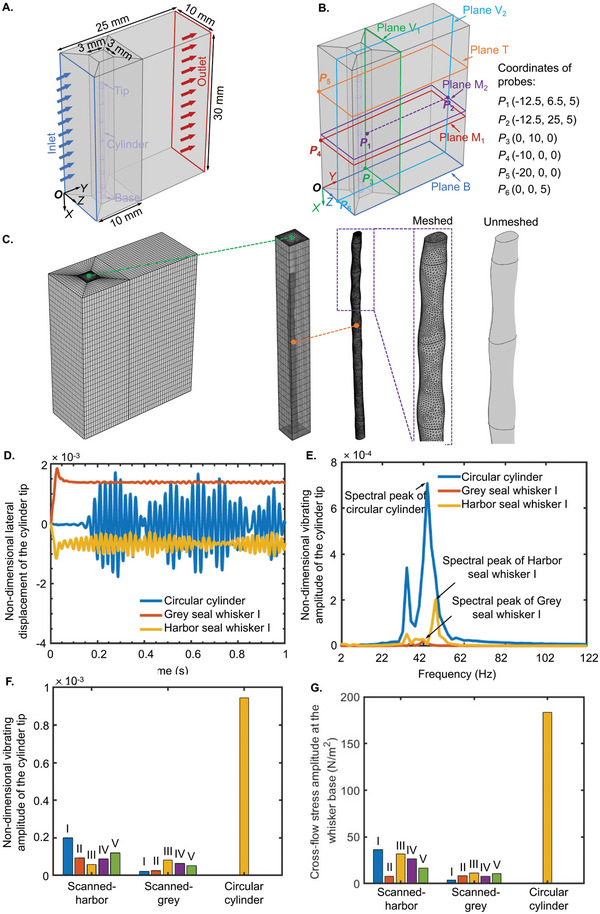
The calculation domains in COMSOL Multiphysics‐based FSI studies and quantification of VIVs of seal whisker segments. A) Dimensions of the calculation domains. B) Definitions of planes used to present the 2D vorticity field (planes B, M_1_, and T) and detect the enstrophy in the shedding vortices (planes M_2_, V_1_, and V_2_). C) The generated mesh of the calculation domains. D) Time series of the nondimensional lateral displacement of the cylinder tip in cross‐flow direction. E) FFT‐based analyses of frequency components of the nondimensional vibrating amplitudes of the cylinders. F) Nondimensional vibrating amplitudes of the cylinders. G) Cross‐flow stress amplitude at the cylinder base.

FSI studies were conducted to investigate the vibrations of one smooth circular cylinder and undulating seal whisker structures in an oncoming steady flow of velocity *U_∞_* = 0.2 m s^−1^ (**Figure** **5**A). Detailed settings, including calculation domains and mesh (Figure 5B,C) of the FSI studies, are presented in FSI Study: High‐Performance Computing section of the Experimental Section. The flow generated a drag force that was the combination of viscous and especially pressure drag, which elicited deformations of the cylinders (circular cylinder and seal whisker). Meanwhile, the cylinders vibrated in the cross‐flow direction because of the unsteady and periodic reaction caused by the shed vortices. Based on the FSI studies, the circular cylinder and seal whisker's cross‐flow tip displacements were monitored and compared to quantify the seal whiskers’ VIV suppression capability. In addition, the lateral tip displacement (*T*) in the cross‐flow direction was normalized to the characteristic diameter (*D*) of the cylinders in the cross‐flow direction to ensure comparability of values for the various modeled structures (Nondimensional Cross‐Flow Tip Displacement and Vibrating Amplitude section of the Experimental Section). Because the circular cylinder and the whiskers had similar dimensions (characteristic diameter 0.8 mm and length 25 mm), their VIVs are comparable. However, due to the asymmetric morphology along the cross‐flow direction of the seal whisker, the vortices shed by the seal whisker were not consistent and symmetric along the whisker length, causing asymmetric vortex‐generated drag in the cross‐flow direction. As a result, when in the presence of a steady flow, the shedding vortex‐generated force acting on the seal whisker caused a nonzero lateral displacement of the whisker tip, and the whisker structure vibrated around this specific position in the cross‐flow direction due to the shedding vortices (Figure 5D). By contrast, the circular cylinder's lateral tip displacement undulated around an almost zero value (Figure 5D).

The difference between the time series and the average ($\bar{T_{N}}$) of the nondimensional cross‐flow tip displacement (*T*_N_) in the last 0.5‐s stable vibrations was defined as the nondimensional vibrating deflection ($V_{a}=T_{N}-\bar{T_{N}}$) of the cylinders. Fast Fourier transform (FFT) was used to analyze the frequency components of the nondimensional vibrating deflection. The FFT featured a peak value at a specific vibrating frequency. And the peak was defined as the nondimensional vibrating amplitude (Figure 5E) of the cylinder. The statistical spectral peaks (Figure 5F) showed the nondimensional vibrating amplitude relation of the cylinders (grey seal whisker < harbor seal whisker < circular cylinder). The nondimensional vibrating amplitude of the circular cylinder was 19.4 and 8.5 times higher than that of the scanned grey seal whisker and harbor seal whisker, respectively (Table S4, Supporting Information), which demonstrated the seal whisker's capability to suppress VIV. Furthermore, the nondimensional vibrating amplitude of the harbor seal whisker was approximately two times higher than that of the grey seal whisker, suggesting that the grey seal whisker had a better capability of suppressing VIV at the tested speed. It must be noted that the above comparison was conducted using geometries arising out of 3D scanning (and hence true to the actual geometry), unlike earlier attempts where the seal whisker‐like structure was idealized without considering the varying transverse (cross‐sectional) geometric parameters along the seal whisker length.^[^ ^3^, ^8^, ^9^, ^10^, ^11^, ^21^, ^22^, ^27^, ^28^, ^29^, ^30^, ^31^ ^]^ The vibrations of seal whiskers caused the stress variation at the whisker base embedded into the muzzle. In the real‐life scenario, while sensing the surroundings, the seal relies on stress variations that manifest as signals (from flow and/or tactile stimuli) in the mechanoreceptors at the whisker base. The stress component in the cross‐flow direction at the cylinder base showed the same relation (grey seal whisker < harbor seal whisker < circular cylinder, Figure 5G) as the nondimensional vibrating amplitude relation. Specifically, the stress at the base of the circular cylinder was 21.7 and 7.7 times higher than that of the scanned grey seal whisker and harbor seal whisker, respectively (Table S4, Supporting Information). In the above analyses, the cross‐flow stress at the cylinder base was calculated by the average value of the traction along the cross‐flow direction at the cylinder base. And the stress amplitude was calculated by noting the FFT peaks of the time series of the stress.

While several methods, including CFD simulations,^[^ ^3^, ^29^, ^32^, ^33^, ^34^ ^]^ PIV,^[^ ^22^, ^23^, ^30^, ^35^ ^]^ and dye injection,^[^ ^10^ ^]^ have previously been employed to investigate VIV suppression in seal whiskers, due to limited access to seal whiskers and the technical challenges of measuring vibrations and flow characteristics at millimeter scale (dimensions ≈ 1 mm), both experiments and numerical simulations^[^ ^3^, ^8^, ^9^, ^10^, ^11^, ^23^, ^27^, ^28^, ^32^, ^33^, ^34^, ^35^ ^]^ have typically used scaled‐up seal whisker‐like structures constructed using the idealized seal whisker structure (Figure 1C), which does not fully capture the 3D whisker geometry, neglecting the important varying transverse (cross‐sectional) geometric parameters mentioned above. Here, we used scanned real‐scale seal whiskers to improve the previous geometrical framework, enabling a more comprehensive and convincing study of the whisker's VIV suppression. In addition, previous research has mainly focused on the VIV suppression capability of harbor seal whiskers,^[^ ^3^, ^4^, ^5^, ^6^, ^7^, ^8^, ^9^, ^10^, ^11^, ^12^, ^13^, ^14^, ^15^, ^16^, ^17^, ^18^, ^21^, ^23^, ^24^, ^25^, ^29^, ^30^, ^32^, ^33^, ^34^ ^]^ paying less attention to that of other species,^[^ ^22^ ^]^ which feature undulating structures with different parameters.

### 2.4 VIV Suppression Ability

#### 2.4.1 Intensity of Vortex Shedding Using 3D Vorticity Isosurface Analysis

**Figure 6:**
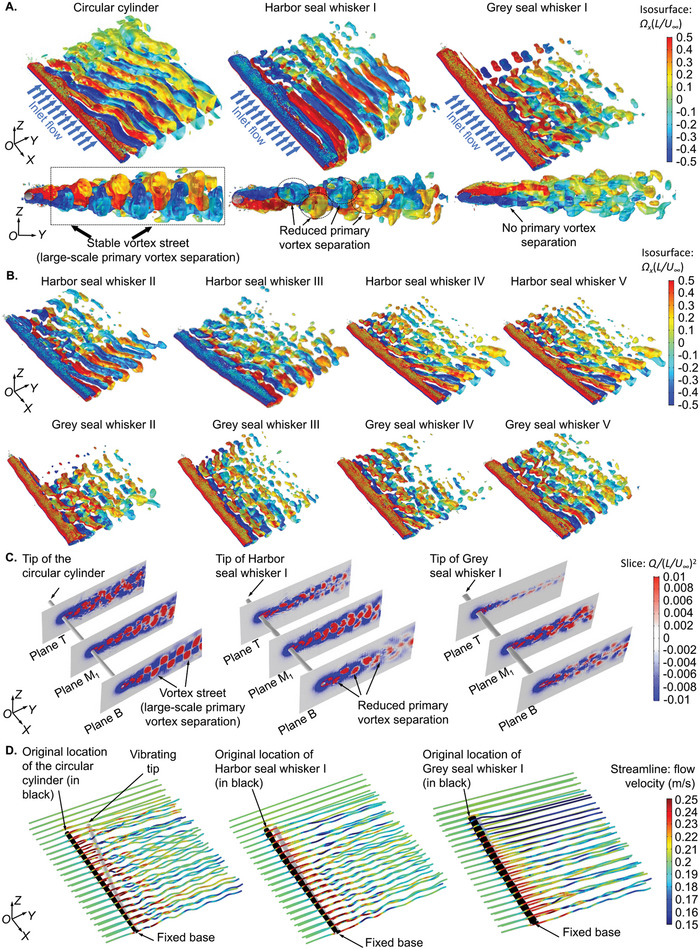
COMSOL Multiphysics‐based FSI studies of scanned real‐size seal whisker segments. A) The vorticity fields obtained using the *Q*‐criterion^[^ ^31^ ^]^ for the cylinders, including circular cylinder, Grey seal whisker I, and Harbor seal whisker I. The *Q*‐criterion values were nondimensionalized by (*U_∞_/D*)^2^. Isosurfaces of the nondimensional *Q*‐criterion value of 0.01 were presented, and the vorticity's *x*‐component of the identified vortical structures was colored. B) The vorticity fields behind Harbor seal whiskers II–V and Grey seal whiskers II–V. C) The vorticity field was characterized by the nondimensional *Q*‐criterion value slices perpendicular to the cylinders. D) The velocity field presented as streamlines surrounding the deformed cylinder (deformation scaled up: 500×).

To reveal the mechanism of VIV suppression employed by the seal whiskers and to comparatively and quantitatively analyze the VIV vibration amplitudes of the various chosen cylinders (grey seal whisker < harbor seal whisker < circular cylinder), parameters including vorticity distributions, velocity fluctuations, and enstrophy variations in the shed vortices, which reflected the vortex‐generated reaction on the upstream cylinders were monitored and compared qualitatively and quantitatively for the circular cylinder, Harbor seal whiskers I–V, and Grey seal whiskers I–V. The vorticity distributions were evaluated by the *Q*‐criterion^[^ ^31^ ^]^ and presented as 3D isosurfaces and slices perpendicular to the cylinders (**Figure** **6**A,B). The definition of the *Q*‐value is presented in Supporting Fluid Dynamics Analyses section of the Experimental Section. The *Q*‐criterion values were nondimensionalized by (*U_∞_*/*D*)^2^. A positive *Q*‐value indicates that the rotational energy exceeds dissipation and denotes a vortical structure in the flow region. The vortical structures were identified by isosurfaces with a nondimensional *Q*‐criterion value of 0.01. Moreover, the vorticity's *x*‐component, which was nondimensionalized by (*U_∞_*/*D*), was colored on the vortical structures (Figure 6A). From the point of view of the 3D vorticity of the isosurfaces around the cylinder, alternately shedding separated vortices formed a stable vortex street behind the circular cylinder. By contrast, only reduced primary vortex separations, which were the detachment of a boundary layer from the cylinder surface into a wake, gradually diffused behind the seal whiskers (Figure 6A). This difference in vortex separation reduced the vortex‐generated reaction acting on the seal whisker, thus causing VIV suppression compared to the circular cylinder. Furthermore, harbor seal whiskers were observed to have more significant vortex separations and more complicated variations in vorticity values than grey seal whiskers temporally and spatially (Figure 6A,B). As a result, a higher shedding vortex‐generated reaction acted on the harbor seal whiskers and thus caused larger VIVs compared to grey seal whiskers.

#### 2.4.2 Intensity of Vortex Shedding Using 2D Vorticity Slice Analysis

The vorticity distribution difference on planes B, M_1_, and T (defined in Figure 5B) revealed that the scale of vortex structures was reduced near the tips of the circular cylinder and seal whiskers (Figure 6C). This is because the vibrations of the distal tip of the structure destroyed the vortex street formed in the alternate shedding vortices. FSI studies enable us to investigate the effect of the whisker vibration on the surrounding vortical structure, which has been rarely investigated in earlier works in which only the flow field surrounding a rigid seal whisker structure was modeled. On the other hand, inferring from the varying trends of the major axis (*a*, Figure 4A1,B1) and minor axis (*b*, Figure 4A2,B2), the seal whisker's characteristic diameter ($D=\frac{a+b}{2}$) decreased from the whisker base to the tip and featured sinusoidal variations. As a result, the local Reynolds numbers also decreased from the base to the tip, causing different states of vortex separation (Figure 6C) to simultaneously exist in different locations from the base to the tip for seal whiskers. Specifically, the scale of vortex structures showed the relation—plane M_1_ > plane B > plane T for Grey seal whisker I and plane M_1_ > plane T > plane B for Harbor seal whisker I (Figure 6C). By contrast, there was a strictly monotonically decreasing trend in the scale of vortex structures along the axis of the circular cylinder.

Furthermore, although the overall scale of vortex structures decreased from the base to the tip of the cylinders, the alternate vortex street still existed to a large extent for the circular cylinder compared to the seal whiskers (Figure 6C). In addition, overall, the scale of vortex structures was reduced more behind the grey seal whisker than behind the harbor seal whisker (Figure 6C). Therefore, it can be inferred from the above characteristics that the shedding vortex intensity showed the relation (grey seal whisker < harbor seal whisker < circular cylinder).

#### 2.4.3 Intensity of Vortex Shedding Using Velocity Streamline Analysis

The reduced vortex intensity near the cylinder tip was also reflected in the velocity streamlines from the near field to the far field, which gradually became smoother from the cylinder base to the tip (Figure 6D). In addition, compared to the circular cylinder, smoother streamlines were observed near the seal whisker tip. Furthermore, the streamlines at the distal end were smoother for the grey seal whisker than for the harbor seal whisker (Figure 6D). The above observations reflected the velocity fluctuation relation (grey seal whisker < harbor seal whisker < circular cylinder), which characterized the turbulent intensity of the flow field.^[^ ^36^ ^]^ Animations of the vorticity and velocity fluctuations behind the cylinders, including one circular cylinder, harbor seal whisker I, and Grey seal whisker I, are presented in Movies S2–S4 of the Supporting Information.

#### 2.4.4 Intensity of Vortex Shedding Using Enstrophy Analysis

**Figure 7:**
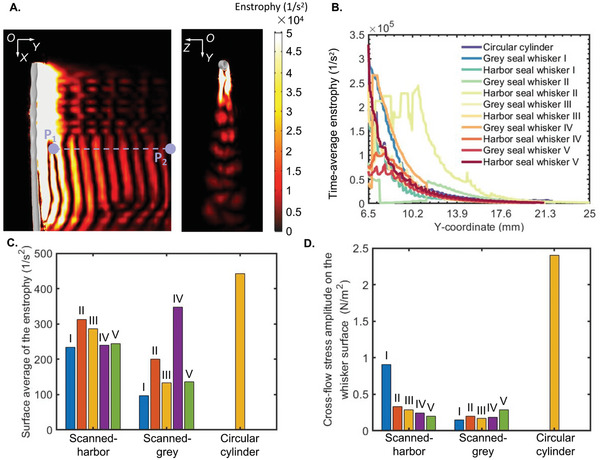
Quantification of the intensity of vortex shedding using enstrophy analysis. A) Enstrophy distribution on plane M_2_ and plane V_2_ of Harbor seal whisker I. B) Time‐average enstrophy along P_1_P_2_ (in purple, (A)) from the near field to the far field. C) Statistical FFT spectral peaks of the surface‐average amplitude of the enstrophy on plane V_1_. D) Statistical FFT spectral peaks of the cross‐flow stress amplitude on the whisker surface.

In addition to the qualitative analyses of the vorticity field and velocity field mentioned above, segment *P*_1_ *P*_2_ (Figure 5B) was used to monitor the enstrophy^[^ ^37^ ^]^ from the near field and far field. The enstrophy is the squared norm of the vorticity, and its increase or decrease indicates the rotation intensity variation of the vortex. It represents the local rate of decay of the kinetic energy of the flow. The detailed definition of the enstrophy is presented in Supporting Fluid Dynamics Analyses section of the Experimental Section. In general, it was observed that the enstrophy decreased overall with an increasing distance to the upstream cylinder (**Figure** **7**A,B). In addition, the time‐average enstrophy showed fluctuations in the near field (where *y*‐coordinate < 10 mm, Figure 7B) then strictly decreased in the far field (where *y*‐coordinate > 10 mm, Figure 7B) because the vortices were in the shedding process in the near field but formed stably in the far field. The decreasing time‐average enstrophy (Figure 7B) in the far field confirmed the decreasing vortex intensity caused by the gradual diffusion of the shedding vortices with the increasing distance to the upstream cylinder. Furthermore, the surface average of the enstrophy at plane V_1_ (*y* = 10 mm, defined in Figure 5B), where the shed vortices were stable, and the enstrophy intensity was strong, was used to evaluate the intensity of vortex shedding behind the circular cylinder and seal whiskers. A higher surface average of the enstrophy indicated a more substantial vortex intensity in the flow field. The surface average of the enstrophy of the circular cylinder was 1.7 times and 2.4 times higher than that of the harbor seal whisker and grey seal whisker (Figure 7C; Table S4, Supporting Information), indicating a quantitative relation of the shedding vortex intensity (grey seal whisker < harbor seal whisker < circular cylinder).

Based on the above qualitative and quantitative comparisons among the vorticity, velocity, and enstrophy variations behind the circular cylinder, the grey seal whisker, and the harbor seal whisker, the shedding vortices’ intensity showed the relation (grey seal whisker < harbor seal whisker < circular cylinder), thus causing the relation (grey seal whisker < harbor seal whisker < circular cylinder, Figure 7D) of shedding vortices‐generated reaction on the upstream cylinder and finally resulting in the VIV relation (grey seal whisker < harbor seal whisker < circular cylinder, Figure 5F). The reaction mentioned above was characterized by the stress on the cylinder surface (Figure 5G; Table S4, Supporting Information). Moreover, the reaction on the circular cylinder was 6.1 times and 12.0 times higher than that of harbor and grey seal whiskers, respectively. The stress on the cylinder surface was calculated following the same methods as the stress at the whisker base.

### 2.5 FSI Studies of One Single Full‐Length Seal Whisker and Seal Whiskers in an Array

**Figure 8:**
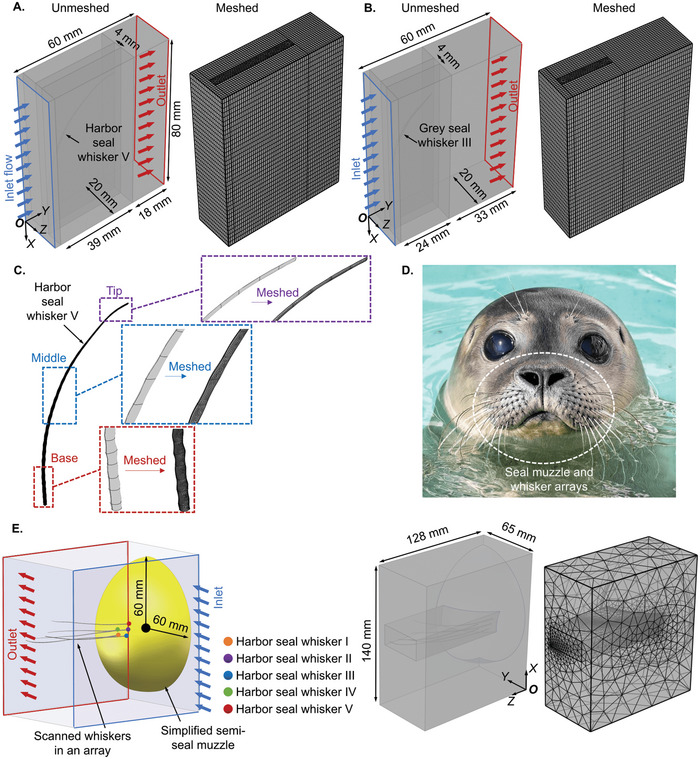
The calculation domains in COMSOL Multiphysics‐based FSI studies of scanned real‐scale seal whiskers with full length and seal whiskers in an array. Dimensions and the generated mesh of the calculation domain for A) Harbor seal whisker V and B) Grey seal whisker III. C) Details of the generated mesh of full‐length Harbor seal whisker V. D) A harbor seal with an array of whiskers mounted on the muzzle. E) A schematic diagram showing locations of five harbor seal whiskers, dimensions of the calculation domains, and the generated mesh in FSI studies for seal whiskers in an array. Seal image was provided by the Zeehondencentrum, Pieterburen, The Netherlands, and are reproduced with permission.

To confirm that the VIV suppression demonstrated on whisker segments holds true for full‐length whiskers, we conducted FSI studies of scanned full‐length Harbor seal whisker V (**Figure** **8**A) and Grey seal whisker III (Figure 8B) (chosen to have similar lengths and characteristic diameters). Details of the mesh (Figure 8C) and calculation domain dimensions are presented in FSI Study: High‐Performance Computing section of the Experimental Section. To gain insights into how VIV responses of seal whiskers in an array on the seal muzzle (Figure 8D) could influence or be influenced by neighboring whiskers, we further conducted FSI studies in which five scanned harbor seal whiskers were mounted at their actual locations on a seal muzzle model (Figure 8E). The above actual locations were recorded during whisker excision from deceased seals. The seal whiskers were located with minor axes parallel to the oncoming flow. Since we did not have access to a scanned real‐scale seal muzzle, we used a simplified seal muzzle (a quarter sphere with a diameter of 60 mm) with dimensions similar to the real seal muzzle in FSI studies (Figure 8E).

**Figure 9:**
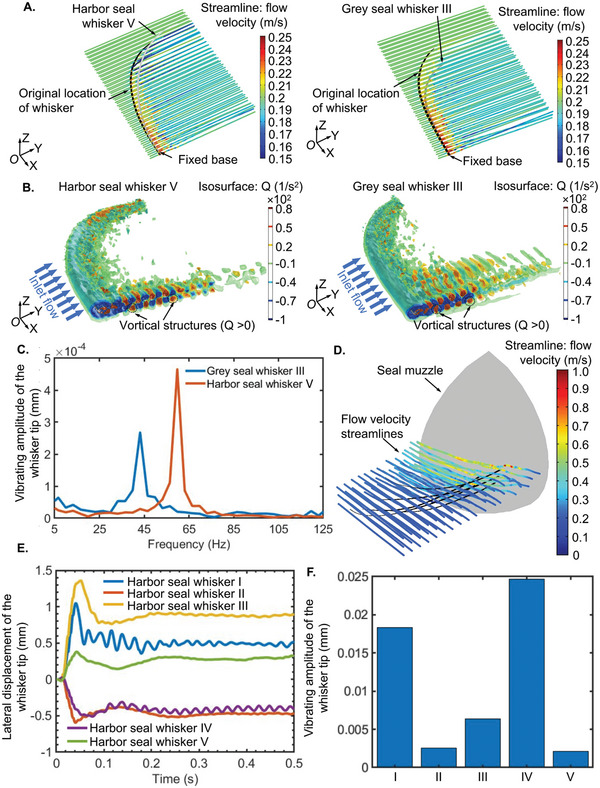
COMSOL Multiphysics‐based FSI studies of full‐length scanned real‐size seal whisker. A) The velocity field. The velocity field was presented as streamlines surrounding the deformed cylinder (deformation scaled up: 500×). B) The vorticity field behind Harbor seal whisker V and Grey seal whisker III. The vorticity field was obtained using the *Q*‐criterion^[^ ^31^ ^]^ and presented as isosurfaces. C) FFT‐based analyses of frequency components of the vibrating amplitudes of Harbor seal whisker V and Grey seal whisker III. D) Flow velocity streamlines around the seal whiskers in an array. E) Cross‐flow tip displacement of seal whiskers in an array. F) Vibrating amplitudes of seal whiskers, which were characterized by FFT spectral peaks of seal whiskers’ cross‐flow displacements.

Concerning the vibration response of one single full‐length seal whisker, the velocity streamlines (**Figure** **9**A; Movie S5, Supporting Information) near the whisker tip remained smooth over time for both seal whiskers, indicating that the velocity fluctuations were tiny in the nearby flow region. This is because the minor axis decreased from the whisker base to the whisker tip, causing a decrease in the local Reynolds number and not allowing primary vortex separation. In addition, the velocity fluctuation range at the near field of Grey seal whisker III (0.2–0.25 m s^−1^, Figure 9A) was smaller than Harbor seal whisker V (0.15–0.25 m s^−1^, Figure 9A). Furthermore, 3D vorticity distributions (Figure 9B; Movie S6, Supporting Information) showed that vortical structures (*Q* > 0) in the downstream region lasted farther for Harbor seal whisker V than Grey seal whisker III (Figure 9B). By contrast, although the vortices shed by Grey seal whisker III had a larger‐scale distribution along the whisker length at the region near the base, they diffused at a shorter distance downstream (Figure 9B). The vorticity distributions and velocity fluctuations mentioned above resulted in a smaller vortex intensity for Grey seal whisker III than Harbor seal whisker V, causing a smaller VIV for Grey seal whisker III, as demonstrated by the FFT spectral peaks of the time series of VIVs (Figure 9C), e.g., for Grey seal whisker III (2.68 × 10^−4^ mm, Figure 9C) as compared to a Harbor seal whisker (4.65 × 10^−4^ mm, Figure 9C). This observation aligned with the VIV relation (grey seal whisker < harbor seal whisker) observed in FSI studies of seal whisker segments, which indicated that a full‐scale scanned seal whisker functioned similarly to whisker segments, thereby further confirming that the above simulations on seal whisker segments were valid for investigating the VIV mechanism of a full‐scale whisker.

To gain more insights into the actual scenario in which seal whiskers were located in an array on the seal muzzle, FSI studies of an array of full‐length seal whiskers (Figure 9D) were conducted. An animation showing the velocity streamline variations and VIVs of seal whiskers in an array is presented in Movie S7 of the Supporting Information. Based on FFT analyses of time series (Figure 9E) of lateral whisker tip displacements, it was found that VIVs of seal whiskers (Figure 9F) in an array were ≈5–50 times larger than that of one single seal whisker (Harbor seal whisker V, 4.65 × 10^−4^ mm, Figure 9C), indicating the effects of neighboring whiskers (upstream: Harbor seal whiskers II, III, and V, downstream: Harbor seal whiskers I and IV) on VIVs of each other.

### 2.6 VIV and WIV Measurements of Whiskers Using 3D‐Printed Graphene‐Based Sensors

**Figure 10:**
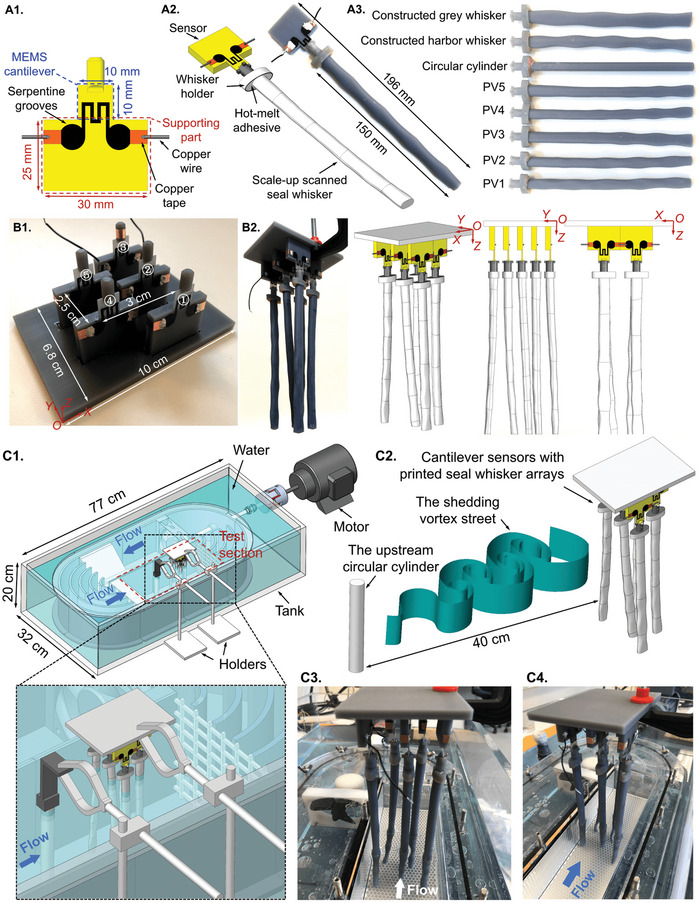
A–C) The 3D‐printed cantilever sensor and whisker vibration measurements in the recirculating water flume. (A1) Dimensions of the designed sensor. (A2) The CAD model and prototype of the sensor with a 3D‐printed seal whisker structure. (A3) The 3D‐printed seal whisker structures. (B1) Five 3D‐printed sensors in an array mounted on a 3D‐printed plate. (B2) The CAD model and prototype of five sensors with 3D‐printed seal whisker structures in an array. (C1) A diagrammatic sketch showing the whisker vibration measurements in which the seal whisker structure arrays were mounted above the test section of the recirculating water flume. (C2) A diagrammatic sketch showing the seal whisker structure arrays in a wake generated by an upstream circular cylinder. The (C3) in‐flow and (C4) axonometric views of seal whisker structure arrays.

In order to validate the VIV suppression capability of constructed seal whiskers (right, Figure 2B) and further explain how the seal whisker interacts with fish wake‐like vortices, and thereby provide a mechanistic explanation for the ultrasensitive fish trail tracking ability of the seal whisker, we developed fully‐3D‐printed MEMS cantilever sensors to conduct experimental measurements of VIV and WIV responses for one single 3D‐printed seal whisker and whiskers in an array (**Figure** **10**; 3D‐Printed MEMS Cantilever Sensor section and VIV and WIV Measurements in the Recirculating Water Flume section of the Experimental Section). Due to the difficulty of measuring tiny vibrations of real‐scale (millimeter‐scale) seal whiskers, we first constructed seal whiskers (length 15 mm) by the proposed geometric framework (Equations (1)–(5)) and cut segments (length 15 mm) of scanned seal whiskers (Harbor seal whiskers I–V), then we scaled the whiskers up ten times. After that, we printed the whiskers and mounted them at the distal tips of fully‐3D‐printed MEMS cantilever sensors embedded with graphene nanomaterial‐based piezoresistors at the hinge (see 3D‐Printed MEMS Cantilever Sensor section of the Experimental Section). Finally, VIV and WIV measurements were conducted in a recirculating water flume (see VIV and WIV Measurements in the Recirculating Water Flume section of the Experimental Section).

**Figure 11:**
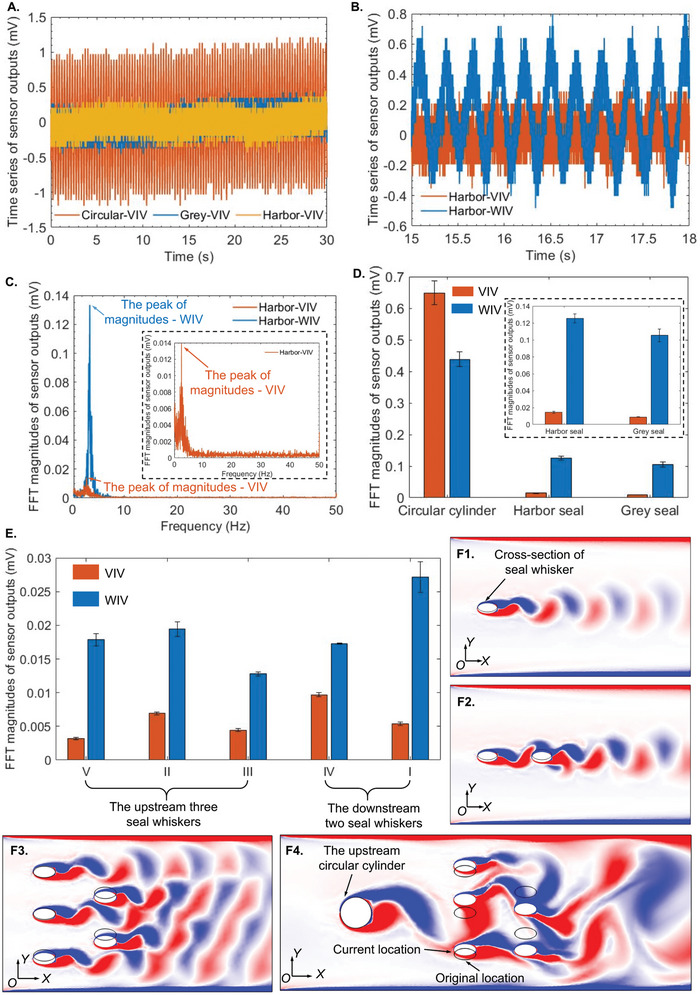
Results of VIV and WIV measurements in the recirculating water flume and their corresponding mechanistic explanations by COMSOL Multiphysics‐based simulations. A) Time series of sensor outputs of one single circular cylinder, one constructed grey seal whisker, and one constructed harbor seal whisker. B) Time series of one constructed harbor seal whisker in VIV and WIV measurements. C) FFT‐based analyses on time series in (B). The left *y*‐axis and the right axis indicated the VIV and WIV, respectively. D) FFT magnitudes of sensor outputs of one single circular cylinder, one constructed grey seal whisker, and one constructed harbor seal whisker. E) FFT magnitudes of sensor outputs of seal whisker structure arrays. F1) Simulated VIV of one single seal whisker (characterized by one elliptical cross‐section). F2) Simulated VIVs of two seal whiskers with an upstream–downstream formation. F3) Simulated VIVs of five seal whiskers (three located upstream and two located downstream). F4) Simulated WIVs of five seal whiskers.

When tested in an open flow generated in the recirculating water flume, the single constructed harbor and grey seal whiskers vibrated approximately three times smaller than a similar‐sized circular cylinder (**Figure** **11**A), validating the VIV suppression capability of constructed whiskers. Furthermore, when located in a wake generated by an upstream vortex generator, the single seal whisker vibrated (WIV) with a primary frequency (≈3.5 Hz) that was nearly locked to the shedding frequency (≈3.75 Hz, calculated by the primary frequency from FFT analysis) of the upstream wake (Figure 11B), which was calculated by the equation $f=\frac{St*v}{D}$ (*f*: the vortex shedding frequency, *St*: Strouhal number (≈0.2), *D*: the diameter of the upstream circular cylinder 8 mm, *v*: the flow velocity 0.15 m s^−1^).^[^ ^38^ ^]^ In addition, by conducting FFT analyses (Figure 11C) on the time series of the sensor to which the constructed seal whisker or circular cylinder was attached, VIVs and WIVs were characterized by noting the dominant peaks in the frequency domain (Figure 11D). It can be found that 1) the VIV trend was—circular cylinder > harbor seal whisker > grey seal whisker, which matched the FSI studies, and 2) WIV of a single isolated circular cylinder was ≈30% smaller than its VIV. By contrast, WIVs (Movie S8, Supporting Information) of single isolated seal whiskers were ≈7–10× more than their VIVs. Finally, the WIVs (Movie S9, Supporting Information) of whiskers in an array were ≈1.5–6× larger than their VIVs (Figure 11E). Their WIV frequencies (2.4 ± 0.5 Hz, calculated by the primary frequency from FFT analysis) locked to the primary frequency (≈2.2 Hz) of the upstream wake as well. The wake frequency was calculated by the equation $f=\frac{St*v}{D}$ (*f*: the wake frequency, *St*: Strouhal number (≈0.22), *D*: the diameter of the upstream circular cylinder 15 mm, *v*: the flow velocity 0.15 m s^−1^).^[^ ^38^ ^]^ The above observations in one single seal whisker and whiskers in an array provided evidence of the seal whisker's efficiency in hydrodynamic signal sensing by suppressing VIV‐induced self‐generated noise and responding with increased vibrations and a vibrating frequency that locked to the primary frequency of the hydrodynamic signal.

### 2.7 FSI Flow Visualization in Whisker Arrays

COMSOL Multiphysics‐based simulations (Figure 11F1–F4) were conducted to visualize the flow vorticity distributions and simulated vibrations of one single seal whisker (Figure 11F1), two whiskers with an upstream–downstream distribution (Figure 11F2), five whiskers with three upstream and two downstream whiskers (Figure 11F3,F4), to provide a mechanistic explanation for the neighboring whiskers’ effects on the vibration of each whisker in an array, which appear to increase compared to the isolated whisker case. In addition, elliptical cross‐sections of whiskers characterized all whiskers, and 2D FSI studies were conducted. Compared to the case of one single whisker in the flow (Figure 11F1), the flow vorticity fluctuation and distribution area surrounding two upstream–downstream whiskers (Figure 11F2) were both larger, resulting in their larger vibrations (Movie S10, Supporting Information), which suggested that the neighboring whiskers affected vibrations with each other and indicated the possibility of a vibration‐strengthening effect in whisker arrays. The neighboring whiskers’ effects were further validated by the larger flow vorticity fluctuation as well as distribution area and the increased vibrations of five whiskers in an array (Figure 11F3) as compared to a single isolated whisker. Furthermore, when located in a wake shed by the upstream circular cylinder, vortices distributions surrounding whiskers were significantly induced by not only neighboring whiskers but also the upstream wake, thus showing larger alternating propagating states (Figure 11F4), which caused larger vorticity fluctuation and distribution area, thus resulting in ≈1.5–6× more vibrations than VIVs in an open flow (Figure 11E).

## 3 Conclusions

In summary, we proposed geometric frameworks for harbor (*Phoca vitulina*) and grey (*Halichoerus grypus*) seal whiskers extracted from 3D measurements of the cross‐sectional morphological parameters of blue light‐scanned harbor and grey seal whiskers. These first records of 3D‐scanned seal whiskers should serve as a valuable database for the interdisciplinary research community interested in learning from seal whiskers, including biologists, physicists, and engineers. Furthermore, FSI studies of scanned real‐scale seal whiskers (three types: 1 – segments, 2 – full‐length, and 3 – in an array) were conducted to gain more insights into the flow variations, including vorticity distributions, velocity fluctuations, and enstrophy variations. In addition, FSI studies enabled us to quantify VIV suppression of seal whiskers and study the effect of whisker vibrations on the vortical structure. Prior to this study, a detailed FSI investigation of scanned seal whiskers at real scale (scanned biological whiskers) have not been conducted. Finally, MEMS cantilever sensor‐based experiments were conducted to measure the VIVs and WIVs, thus 1) validating the VIV suppression capability of whiskers constructed by the proposed geometric frameworks, 2) supporting the hypothesis that seal whisker tracked fish wake by vibrating with a WIV frequency locked to the dominant frequency of the wake, and 3) evidencing that neighboring whiskers in an array affected each other by causing the larger flow vorticity fluctuation as well as distribution area and resulting in increased vibrations compared to a single isolated whisker.

Due to the constitutive response of seal whiskers, this paper simplified the mechanical behavior of seal whiskers. However, the simplicity of this treatment could be a limitation of this study. The mechanical response of whiskers is likely anisotropic, nonlinear, frequency‐dependent, and hysteretic. Future studies will investigate more effects on seal whisker vibrations. Several effects may be observed, including changes in the stiffness of the whiskers and the follicle sinus complex (FSC)^[^ ^39^ ^]^ located at the base. As with twisted fibers used for artificial muscles,^[^ ^40^ ^]^ it might be argued that the undulatory nature of the whiskers is also changing their stiffness, depending on tissue arrangement in the whiskers. Also, the FSC structure, which contains soft and muscular tissue as well as nerve fibers, may affect seal whisker vibration responses.

It is encouraging that the proposed parametric framework applies for two different seal species, suggesting that it may also be extended to other undulating seal whisker morphometrics. Furthermore, the framework can also help design a seal whisker structure with VIV suppression capability, thus causing less self‐generated noise when developing flow sensors or underwater structures. The numerical FSI studies and MEMS 3D‐printed cantilever sensor‐based experiments provide more insights into the fluid‐seal whisker structure response, revealing the undulating morphology's contributions to modifying the shedding vortex states and quantifying the seal whisker's VIV suppression. It was found that downstream vortex shedding intensity and resulting VIVs were consistently lower for grey seal whiskers than harbor seal whiskers and a smooth cylinder, supporting the hypothesis that the grey seal whisker is an ideal template for VIV‐resistant underwater structures. To be more specific, we can use the proposed morphological framework of grey seal whiskers to generate seal whisker‐inspired structures, such as wind turbines, that can reduce much more structural fatigue and drag compared to the existing whisker structures.^[^ ^18^ ^]^ In the future, our focus will be on further investigating and optimizing various cross‐sectional parameters to obtain a better VIV suppression capability using grey whiskers constructed through the proposed geometric framework in this article. Finally, we will design low‐noise flow sensors that can be used in seal‐inspired underwater robots.

## 4 Experimental Section

### Scans of Seal Whiskers

To obtain a comprehensive geometric framework, ten whiskers, five each from the muzzles of deceased harbor seals (*Phoca vitulina*) (age ≈ 10 days to 1–2 years) and grey seals (*Halichoerus grypus*) (adults) were first collected at Seal Rehabilitation and Research Centre, Pieterburen, The Netherlands. The whiskers were scanned using a high‐resolution blue light scanning system (GOM ATOS III Triple Scan 8M)^[^ ^41^ ^]^ (Figure 1D), featuring an 8‐megapixel dual‐camera system with a 90 mm lens for the cameras and a 120 mm projector lens. The machine was used with the “SO MV60” preset configuration (scan volume: 60 mm × 45 mm × 35 mm, measuring point distance: 19 µm, accuracy ≈ 5 µm). Before scanning, a thin coating (≈5 µm) of chalk spray (Figure 1D) was applied to the whisker using an airbrush to enhance reflectance. 0.4 mm photogrammetric targets were used. All the scans were gathered, and the point clouds were processed into a single mesh using GOM ATOS Professional software. Mesh data were converted to CAD surfaces (Figure 1D) in Geomagic Design X. The 3D scanning and reverse engineering processes were performed by a local company (TetraVision BV, Belgium), generating 3D models (Figure 1E, also available in Data S1 of the Supporting Information as SOLIDWORKS SLDPRT files and 3D PDF files).

### Cross‐Sectional Morphological Parameter Measurements

Transverse cross‐section measurements were conducted in the proximal‐distal direction of the whisker segment with a spacing of 0.5 mm between successive transverse planes (in red, Figure 2A). The basal section of the seal whisker, embedded in the muzzle, had a length of ≈10 mm (Figure 2B) and was not considered in the measurements since this section does not feature any undulations. Since the whiskers have a range of lengths, 25 mm segments were chosen for geometric parameterization (in purple, Figure 2B). The detailed steps involved in the measurements are as follows.

i) The whisker was oriented in such a manner that its curvature occurs roughly toward the left (Figure 2A) in SOLIDWORKS, with the centerline of the nearly‐straight beginning part of the seal whisker to be roughly horizontal. A coordinate system named *O‐XYZ* (Figure 2A) was constructed, of which the origin was fixed at the centroid of the base of the measured section (in purple) of the seal whisker. The *OX* axis was perpendicular to the paper and pointed away from it. The *OZ* axis and *OY* axis were pointed right and down, respectively (Figure 2A).
ii) Each seal whisker was first cut through planes (in green, Figure 2A) perpendicular to the screen and parallel to the *OXY* plane. When a CAD model of the seal whisker was imported into the software SolidWorks, the imported seal whisker was rotated with a specific orientation, which ensured that the measured section of the seal whisker was entirely located on one side (left or right) of the defined *OXY* plane. As a result, there will not be multiple cross‐sections for the same *z*‐coordinate. The planes were spaced 0.50 mm apart from each other. The first plane was located at *z* = 0.5 mm. For each cross‐section obtained in this manner, the centroid (in blue, Figure 2A) was determined. Since the centerline of the nearly‐straight beginning part of the seal whisker could only be denoted as roughly horizontal, and since this caused a slight nonparallelism between the defined *OZ* axis and the centerline of the whisker, an error of around 0.01 mm occurs in the *z*‐coordinates of several cross‐sections. However, such an error was deemed small, considering that the adjacent cross‐sections feature a separation of 0.5 mm along the *OZ* axis.
iii) The centroids of all the above cross‐sections were connected to generate the entire whisker's centerline (in blue, Figure 2A). New planes (in red, Figure 2A) were then constructed perpendicular to the centerline at each centroid to reseparate the measured section (in purple) of the seal whisker and obtain the new cross‐sections used for final measurements. Each new cross‐section was then captured using the same view and scale and fitted using a standard ellipse in ImageJ software. One set of captured cross‐sections of one scanned harbor seal whisker is provided in Data S3 of the Supporting Information.
iv) Based on the captured cross‐sections mentioned above, five parameters on each fitted ellipse of each cross‐section obtained above, including the major axis (*a*), minor axis (*b*), rotating angle (*θ*), *X*‐coordinate, and *Y*‐coordinate of the centroid (*x*, *y*) (Figure 2A) were measured in ImageJ software. In addition, *θ* was determined as the angle between the *OX* axis and the cross‐section's major axis projection on the *OXY* plane (Figure 2A). The *Z*‐coordinate of the first cross‐section's centroid was *z* = 0.5 mm, and the *Z*‐coordinate of the following cross‐sections had an increasing interval of 0.5 mm. Raw data of the cross‐sectional morphological parameters mentioned above can be found in Data S2 of the Supporting Information.

### FSI Study: High‐Performance Computing

FSI studies were conducted using COMSOL Multiphysics software.^[^ ^42^ ^]^ Peregrine high‐performance computing cluster (running on 2 × Intel(R) Xeon(R) CPU E5‐2680 v4 at 2.40 GHz and using two sockets with 28 cores in total) at the University of Groningen was used to solve transient fully coupled FSI problems of seal whisker segments. A 1‐s simulation for the seal whisker segment took ≈30–50 h. One computer with an AMD Ryzen Threadripper 3960 × 24‐Core Processor at 3.79 GHz and 64 GB internal memory simulated full‐length seal whiskers. A half‐a‐second full‐length seal whisker simulation took ≈72 h. One computer with 2 × Intel(R) Xeon(R) CPU E5‐2678 v3 at 2.50 GHz and 96 GB internal memory was used to simulate the whiskers in an array. A seal whisker array simulation lasting 0.5 s in simulation time took ≈96 h to compute.

### Simulation Settings of Seal Whisker Segments

The circular cylinder used in the simulation had a diameter (*d* = 0.8 mm) that was identical to the seal whisker's characteristic diameter (≈0.86 mm for grey seal whiskers and ≈0.77 mm for harbor seal whiskers), ensuring the comparability of the cylinders’ vibration responses under similar flow conditions (*Re* ≈ 160). The characteristic diameter (*D*) of the seal whisker mentioned above was calculated by averaging the major and minor axes, as defined in Equation (6). The cylinders (one circular cylinder and scanned seal whiskers) had a length of 25 mm (Figure 5A) and were located in a flow domain whose size was 25 mm × 10 mm × 30 mm (length × width × height). The cylinder base was fixed, and the center of the cylinder base was 5 mm away from the walls. In each simulation, the seal whisker was located with the major axis of the cross‐section aligning to the flow direction. This is in line with typical vibration analyses on seal whiskers conducted in the literature,^[^ ^3^, ^32^ ^]^ which mention that an angle of attack of 0° might be a more prevalent case when a seal chases prey.

The flow domain was divided into six parts (Figure 5A), including two rectangular and four trapezoidal domains. The trapezoidal domains surrounded a rectangular domain of 3 mm × 3 mm × 30 mm (length × width × height). The cylinder was located in this rectangular domain. Three planes, B, M_1_, and T, were defined to present the 2D vorticity field in the shedding vortices (Figure 5B). Plane B coincided with the whisker base. Plane M_1_ and plane T had distances of 10 and 20 mm from the whisker base, respectively. In addition, planes M_2_ and V_2_ (Figure 5B) were used to present the enstrophy distributions behind the cylinder. Furthermore, segment *P*_1_ *P*_2_ (Figure 5B) was set to detect the local enstrophy in the shedding vortices. *P*_1_ and *P*_2_ were located on plane M_2_, right behind the cylinder, and in a line on plane M_2_. Plane M_2_ was parallel to the cylinder base at a distance of 12.5 mm. The surface average of the enstrophy at plane V_1_ (Figure 5B) was used to evaluate the vortex intensity behind the circular cylinder and seal whiskers.

A tetrahedral mesh with a minimum element size of 0.0632 mm and a maximum element size of 0.585 mm was used for the cylinder. A predefined mesh with a minimum element size of 0.316 mm and a maximum element size of 1.06 mm was used for the rectangular domain surrounding the cylinder (Figure 5C). In addition, a sweep‐mapped mesh was used for the four trapezoidal domains and the other rectangular domain (Figure 5C). The number of elements of the generated mesh is on the order of ≈10^6^, which guarantees that the undulating surface of the seal whisker remains after the meshing operation.

The flow domain was set as water, with a dynamic viscosity of 0.001 Pa s and a density of 1000 kg m^−3^. Apart from the no‐slip wall condition used for the surface of the seal whisker, the slip condition was used for other walls of the flow domain. The Young's modulus, Poisson's ratio, and density of the cylinders were set as 6 GPa, 0.4, and 1300 kg m^−3^, respectively. The material properties above were determined through nanoindentation tests^[^ ^43^ ^]^ and Archimedes’ principle based on seal whiskers. A laminar flow model was used, and linear elasticity was assumed for the whisker deformation to solve transient fully coupled simulations to gain insight into the vibration response of the seal whiskers induced by the shedding vortices. Each case of the simulations lasted 1 s, and the last 0.5‐s stable vibrations of the cylinders were used to analyze and compare the vibrating amplitudes of the cylinder tips for quantifying and validating the VIV suppression capability of the seal whiskers.

### Simulation Settings of Seal Whiskers with Full Length

The full‐length seal whiskers required a larger flow domain (height ≈ 80 mm, length ≈ 60 mm, width ≈ 20 mm, Figure 8A,B). Seal whiskers were located with minor axes roughly paralleling the oncoming flow in a rectangular domain with a tetrahedral mesh (minimum element ≈ 0.347 mm, maximum element ≈ 1.84 mm). Because Harbor seal whisker V and Grey seal whisker III had various curvatures downstream, the above rectangular domain has various dimensions for the two seal whiskers. In addition, sweep‐mapped meshes with a minimum element size of 0.693 mm and a maximum element size of 2.32 mm were used for the four surrounding rectangular domains. Finally, a tetrahedral mesh with a minimum element size of 0.139 mm and a maximum element size of 1.28 mm was used for the full‐length whiskers. The number of elements of the generated mesh is on the order of ≈10^6^, ensuring that the undulating surface of the meshed seal whisker remains from the base to the tip (Figure 8C). The simulations lasted 0.5 s, and vibrations of the undulating seal whiskers became stable after 0.2 s. The material properties, the oncoming flow velocity, the physical model of the flow, the boundary conditions, and the solver were identical to simulations of seal whisker segments.

### Simulation Settings of Scanned Real‐Scale Seal Whisker Array

The flow domain surrounding the seal whisker array used a finer mesh (minimum element ≈ 4.56 mm, maximum element ≈ 18.2 mm). The left domain used a coarser mesh (minimum element ≈ 6.38 mm, maximum element ≈ 30.1 mm). A mesh with a minimum element of 0.137 mm and a maximum element of 2.1 mm was used for the seal whiskers. In addition to the no‐slip wall condition used for the surfaces of the seal whiskers and the simplified seal muzzle model (a quarter sphere with a diameter of 60 mm), the slip condition was used for the remaining walls in the calculation domain.

### Nondimensional Cross‐Flow Tip Displacement and Vibrating Amplitude

The nondimensional cross‐flow tip displacement (*T*_N_) was defined in Equation (6), where *T* and *D* are the cross‐flow tip displacement and the characteristic diameter, respectively. The characteristic diameter (*D*) was defined as the average of the major and minor axes for the seal whisker or the diameter for the circular cylinder, as defined in Equation (7)

(6) $$T_{N}=\frac{T}{D}$$

(7) $$D=\left\{\begin{array}{cc} \frac{a+b}{2}, & \mathrm{for}\;\mathrm{seal}\;\mathrm{whisker}\\ d, & \mathrm{for}\;\mathrm{circular}\;\mathrm{cylinder} \end{array}\right.$$

### Supporting Fluid Dynamics Analyses

Vortical structures behind the seal whisker were identified via the *Q*‐criterion.^[^ ^31^ ^]^ The *Q*‐value is defined in Equation (8). $∥\bar{\Omega }∥$ defined in Equation (9) and $∥\bar{S}∥$ defined in Equation (10), respectively, indicate Frobenius norms of rotation rate tensor/vorticity ($\bar{\Omega })$ and rate‐of‐strain tensor $\left(\bar{S}\right)$. The velocity gradient ∇**u** (Equation (11)) of the velocity **u** (*u*, *v*, *w*) can be decomposed into the sum of a symmetric matrix $\bar{\Omega }$ and a skew‐symmetric matrix $\bar{S}$, as defined in Equations (9) and (10). *u*, *v*, and *w* indicate the flow velocity components along the *x*‐axis, *y*‐axis, and *z*‐axis defined in Figure 5A. A flow region where *Q* > 0 is identified as having a vortical structure. The vorticity ($\bar{\Omega })$ is a vector, and its variation indicates the change in positive or negative vorticity. The enstrophy *ε* is the squared norm of the vorticity, defined in Equation (12). Its increase or decrease represents the vortex's rotation intensity variation^[^ ^44^ ^]^

(8) $$Q=\frac{1}{2}*(∥\bar{\Omega }{∥}^{2}-\left(∥\bar{S}{∥}^{2}\right)$$

(9) $$\bar{S}=\frac{1}{2}*\left[\nabla u+{\left(\nabla u\right)}^{T}\right]$$

(10) $$\bar{\Omega }=\frac{1}{2}*\left[\nabla u-{\left(\nabla u\right)}^{T}\right]$$

(11) $${\left(\nabla u\right)}^{T}=\left[\begin{array}{ccc} \frac{\partial u}{\partial x} & \frac{\partial v}{\partial x} & \frac{\partial w}{\partial x}\\ \frac{\partial u}{\partial y} & \frac{\partial v}{\partial y} & \frac{\partial w}{\partial y}\\ \frac{\partial u}{\partial z} & \frac{\partial v}{\partial z} & \frac{\partial w}{\partial z} \end{array}\right]$$

(12) $$\varepsilon =∥\bar{\Omega }{∥}^{2}$$

### 3D‐Printed MEMS Cantilever Sensor

The fully 3D printed MEMS sensor designed for whisker testing (total sensor length 43 mm, width 30 mm, Figure 10A1) was composed of a MEMS cantilever structure (length 10 mm, width 10 mm, aspect ratio 100, thickness 0.1 mm) and a supporting fixture (length 30 mm, width 25 mm, thickness 3 mm), both printed using the “Grey Pro” material (flexural modulus ≈ 2.2 GPa) of the 3D printer named Formlabs Form 3. A high gauge factor Graphene nanoplatelets piezoresistor sensing element (thickness 7 nm, Figure 10A1) was formed at the hinge through a drop‐casting process, which involved dropping a dilute conductive graphene dispersion^[^ ^45^, ^46^ ^]^ into serpentine grooves (depth 0.1 mm, width 1 mm) of the MEMS cantilever structure. A 3D‐printed seal whisker structure (“Grey Pro”) was then embedded into a hole of a 3D‐printed whisker holder (“Grey Pro”) and surrounded with the hot‐melt adhesive. The whisker holder was thereafter attached to the MEMS cantilever's free end (Figure 10A2).

### VIV and WIV Measurements in the Recirculating Water Flume

Two types of vibration measurements were conducted in the recirculating water flume. Type 1: VIV and WIV measurements of five scanned harbor seal whiskers (PV1–PV5, scaled up ten times real‐scale whisker structures, Figure 10A3). Type 2: VIV and WIV measurements of grey and harbor seal whiskers constructed using the proposed geometric framework (scaled up ten times, Figure 10A3), with comparisons to one similar‐sized circular cylinder (diameter 8 mm). In Type 1, five cantilever sensors were located in an array on a 3D‐printed plate (Figure 10B1), with five 3D‐printed whiskers attached to the sensor arrays (Figure 10B2). The five whiskers were located with the same relative locations on the seal muzzle (Figure 8E). In Type 2, only one cantilever with the whisker (circular cylinder or constructed grey/harbor whiskers) was used.

In VIV and WIV measurements, the above sensor(s) with the 3D‐printed seal whisker(s) was (were) located in a recirculating water flume (5 L Loligo System swim tunnel, Figure 10C1–C4), with the seal whisker(s) or the circular cylinder immersing into the water (depth ≈ 8 cm). In VIV measurements, seal whiskers were located in an open flow, with their overall major radius paralleling the oncoming flow (0.15 m s^−1^). In WIV measurements, another circular cylinder was located upstream, separating 40 mm from the downstream single seal whisker or whiskers in an array. Besides, values of the diameter of the upstream circular cylinder were 8 and 15 mm in WIV measurements of the single seal whisker and whiskers in an array, respectively. Due to the reaction from the self‐shed vortices downstream or the wake (Figure 10C2) generated by the upstream vortex generator (circular cylinder), the downstream seal whisker(s) or circular cylinder vibrated, thus exciting the connected cantilever sensor. Using a voltage divider circuit, the freely vibrating cantilever sensor's piezoresistive outputs (sampling frequency 5 kHz, Figure 11A,B) were recorded using a data acquisition system (National Instruments, USB 6289). The VIVs and WIVs were measured by conducting FFT analyses on the time series of sensor outputs and noting the dominant peaks (Figure 11C) in the frequency domain (Figure 11D,E).

## Conflict of Interest

The authors declare no conflict of interest.

## Authors Contribution

Conceptualization: X.Z., A.K., M.C., A.G.P.K. Methodology: X.Z. Investigation: X.Z. Visualization: X.Z. Supervision: M.C., A.G.P.K. Writing the original draft: X.Z.; Writing, reviewing, and editing: X.Z., A.K., M.C., and A.G.P.K.

## Supporting information

Supporting Information

Supporting Information

Supporting Information

Supporting Information

Supplemental Movie 1

Supplemental Movie 2

Supplemental Movie 3

Supplemental Movie 4

Supplemental Movie 5

Supplemental Movie 6

Supplemental Movie 7

Supplemental Movie 8

Supplemental Movie 9

Supplemental Movie 10

## Data Availability

The data that support the findings of this study are available from the corresponding author upon reasonable request.
