# Supplementary material for: Wavy Whiskers in Wakes: Explaining the Trail‐Tracking Capabilities of Whisker Arrays on Seal Muzzles
Source: Adv Sci (Weinh). 2022 Nov 20;10(2):2203062. doi: 10.1002/advs.202203062 (PMC9839859; doi:10.1002/advs.202203062)
Supplement: Supplementary file 1 — Supporting Information [file ADVS-10-2203062-s010.pdf]

ADVANCED SCIENCE
Open Access

Supporting Information

for *Adv. Sci.*, DOI 10.1002/advs.202203062

Wavy Whiskers in Wakes: Explaining the Trail-Tracking Capabilities of Whisker Arrays on Seal Muzzles

Xingwen Zheng, Amar M. Kamat, Ming Cao and Ajay Giri Prakash Kottapalli**

Supporting Information

Supplementary Data S1: CAD models of the scanned real-scale and constructed seal whiskers

Supplementary Data S2: Raw data of the geometrical framework parameters of each harbor and grey seal whisker

Supplementary Data S3: One set of the captured cross-sections of one scanned harbor seal whisker

Supplementary Table S1: Detailed values of geometrical framework parameters obtained from each seal whisker

Supplementary Table S2: The geometrical framework parameters of harbor and grey seal whiskers

Supplementary Table S3: The volume, surface area, major axis, and minor axis of the scanned and constructed seal whiskers

Supplementary Table S4: VIV, base stress, and enstrophy of the circular cylinder and scanned seal whiskers

Supplementary Movie S1: Zoomed-in details of the undulating surfaces of seal whiskers

Supplementary Movie S2: The vorticity field presented as isosurfaces for grey and harbor seal whisker segments

Supplementary Movie S3: The vorticity field presented as slices for grey and harbor seal whisker segments

Supplementary Movie S4: The velocity field presented as streamlines and vortex-induced vibrations of grey and harbor seal whisker segments

Supplementary Movie S5: The velocity field presented as streamlines and vortex-induced vibrations of single grey and harbor seal whiskers with full length

Supplementary Movie S6: The vorticity field presented as isosurfaces of single grey and harbor seal whiskers with full length

Supplementary Movie S7: The velocity field presented as streamlines and vortex-induced vibrations of full-length harbor seal whiskers in an array

Supplementary Movie S8: The experiments of wake-induced and self-shed vortex-induced vibrations of one single seal whisker

Supplementary Movie S9: The experiments of wake-induced and self-shed vortex-induced vibrations of seal whiskers in an array

Supplementary Movie S10: Fluid-structure interaction simulations of seal whiskers in an array.

Supporting Information

Wavy whiskers in wakes: Explaining the trail-tracking capabilities of whisker arrays on seal muzzles

Xingwen Zheng[1,2], Amar M. Kamat[2], Ming Cao[1], Ajay G. P. Kottapalli[2,3*]*

Table S1. Detailed values of geometrical framework parameters from each seal whisker

Whisker	Parameters							
Harbor seal whisker I	V_1	V_2	V_3	V_4	V_5	V_6	V_7	V_8
	0.13	2.0	2.6π	1.0	0.066	2.0	1.6π	-0.0084
	V_9	V_{10}	V_{11}	V_{12}	V_{13}	V_{14}	V_{15}	
	0.71	-0.0024	0.096	-0.18	-0.0032	0.0024	-0.026	
Harbor seal whisker II	V_1	V_2	V_3	V_4	V_5	V_6	V_7	V_8
	0.13	2.0	0.50π	0.92	0.066	2.0	0	-0.0084
	V_9	V_{10}	V_{11}	V_{12}	V_{13}	V_{14}	V_{15}	
	0.64	0.0012	-0.0074	0.017	-0.0040	0.040	-0.060	
Harbor seal whisker III	V_1	V_2	V_3	V_4	V_5	V_6	V_7	V_8
	0.16	1.6	0.90π	1.1	0.077	1.7	1.3π	-0.0084
	V_9	V_{10}	V_{11}	V_{12}	V_{13}	V_{14}	V_{15}	
	0.64	0.0020	0.014	-0.081	-0.0024	0.017	-0.011	
Harbor seal whisker IV	V_1	V_2	V_3	V_4	V_5	V_6	V_7	V_8
	0.15	1.7	1.9π	1.0	0.069	1.7	1.4π	-0.0084
	V_9	V_{10}	V_{11}	V_{12}	V_{13}	V_{14}	V_{15}	
	0.62	0.0012	-0.016	0.060	-0.0020	-0.0014	0.023	
Harbor seal whisker V	V_1	V_2	V_3	V_4	V_5	V_6	V_7	V_8
	0.15	1.8	1.7π	0.97	0.055	1.8	0.80π	-0.0078
	V_9	V_{10}	V_{11}	V_{12}	V_{13}	V_{14}	V_{15}	
	0.57	-0.00008	0.022	-0.022	-0.0056	0.052	-0.042	
Grey seal whisker I	V_1	V_2	V_3	V_4	V_5	V_6	V_7	V_8
	0.12	1.4	1.1π	1.3	0.038	1.4	0.20π	-0.0080
	V_9	V_{10}	V_{11}	V_{12}	V_{13}	V_{14}	V_{15}	
	0.60	-0.00080	0.042	-0.051	-0.0032	0.015	0.035	
Grey seal whisker II	V_1	V_2	V_3	V_4	V_5	V_6	V_7	V_8
	0.13	1.4	1.2π	1.2	0.038	1.4	0.20π	-0.0092
	V_9	V_{10}	V_{11}	V_{12}	V_{13}	V_{14}	V_{15}	
	0.61	0.0028	-0.038	0.10	-0.0036	0.022	-0.071	
Grey seal whisker III	V_1	V_2	V_3	V_4	V_5	V_6	V_7	V_8
	0.13	1.6	0.60π	1.1	0.038	1.6	1.6π	-0.0076
	V_9	V_{10}	V_{11}	V_{12}	V_{13}	V_{14}	V_{15}	
	0.61	0.00028	0.0050	-0.020	-0.0024	0.038	-0.069	
Grey seal whisker IV	V_1	V_2	V_3	V_4	V_5	V_6	V_7	V_8
	0.14	1.5	1.0π	1.2	0.043	1.4	0.10π	-0.0094
	V_9	V_{10}	V_{11}	V_{12}	V_{13}	V_{14}	V_{15}	
	0.60	0.00040	0.0018	0.0025	-0.0040	0.016	-0.055	
Grey seal whisker V	V_1	V_2	V_3	V_4	V_5	V_6	V_7	V_8
	0.12	1.4	1.2π	1.2	0.043	1.4	0.20π	-0.0084
	V_9	V_{10}	V_{11}	V_{12}	V_{13}	V_{14}	V_{15}	

0.64	0.000080	0.00016	0.0035	-0.0016	0.0018	0.0026

Table S2. The geometrical framework parameters of harbor and grey seal whiskers

Item	Parameters							
Harbor seal whisker	V_1	V_2	V_3	V_4	V_5	V_6	V_7	V_8
	0.14 ± 0.013	1.8 ± 0.18	1.5π ± 0.83π	1.0 ± 0.066	0.067 ± 0.0079	1.8 ± 0.16	1.0π ± 0.64π	-0.0082 ± 0.00026
	V_9	V_{10}	V_{11}	V_{12}	V_{13}	V_{14}	V_{15}	
	0.64 ± 0.050	0.00040 ± 0.0017	0.022 ± 0.044	-0.041 ± 0.093	-0.0036 ± 0.0014	0.022 ± 0.024	-0.023 ± 0.032	
Grey seal whisker	V_1	V_2	V_3	V_4	V_5	V_6	V_7	V_8
	0.13 ± 0.0084	1.4 ± 0.082	1.0π ± 0.25π	1.2 ± 0.071	0.040 ± 0.0027	1.4 ± 0.092	0.46π ± 0.64π	-0.0086 ± 0.00076
	V_9	V_{10}	V_{11}	V_{12}	V_{13}	V_{14}	V_{15}	
	0.61 ± 0.016	0.00040 ± 0.0014	0.0022 ± 0.028	0.0074 ± 0.057	-0.0028 ± 0.00096	0.019 ± 0.013	-0.032 ± 0.048	

Table S3. The volume, surface area, major axis, and minor axis of the scanned and constructed seal whiskers

Items	Constructed grey seal whisker	Scanned grey seal whisker	Errors of grey seal whisker framework	Constructed harbor seal whisker	Scanned harbor seal whisker	Errors of harbor seal whisker framework
Volume (mm^3)	11.65	13.82 ± 0.8582	15.7%	10.29	12.03 ± 1.059	14.5%
Surface area (mm^2)	69.45	71.96 ± 3.127	3.49%	61.60	64.45 ± 2.728	4.42%
Major axis (mm)	1.189	1.188 ± 0.066	0.0842%	0.9994	0.9955 ± 0.064	0.110%
Minor axis (mm)	0.5012	0.5255 ± 0.023	4.62%	0.5335	0.5018 ± 0.048	6.32%
Ratio of the major to minor	2.372	2.440 ± 0.1838	2.79%	1.873	1.968 ± 0.2244	4.83%
Area of the cross-section (mm^2)	0.4678	0.4652 ± 0.0338	0.559%	0.4185	0.4067 ± 0.0419	2.90%
Perimeter of the cross-section (mm)	2.950	3.017 ± 0.1471	2.22%	2.608	2.689 ± 0.1282	3.01%

Table S4. VIV, stress, and enstrophy of the circular cylinder and scanned seal whiskers

Items	Circular cylinder	Scanned grey seal whiskers	Scanned harbor seal whiskers
VIV	9.456×10^{-4}	4.872×10^{-5} ± 2.588×10^{-5}	1.115×10^{-4} ± 5.495×10^{-5}
Ratio of the circular cylinder's VIV to the seal whisker's	N/A	19.4	8.5
Base stress (N/m^2)	183.234	8.433 ± 2.901	23.811 ± 11.633
Ratio of the circular cylinder's stress at the base to the seal whisker's	N/A	21.7	7.7
Surface average of the enstrophy ($1/s^2$)	443.1	183.0 ± 99.4	263.7 ± 34.5
Ratio of the circular cylinder's enstrophy average to the seal whisker's	N/A	2.4	1.7
Surface stress (N/m^2)	2.4000	0.1995 ± 0.05190	0.3945 ± 0.2907
Ratio of the circular cylinder's surface stress to the seal whisker's	N/A	12.0	6.1
