# Supplementary figures and images for: Wavy Whiskers in Wakes: Explaining the Trail‐Tracking Capabilities of Whisker Arrays on Seal Muzzles (part 2 of 2)
Source: Adv Sci (Weinh). 2022 Nov 20;10(2):2203062. doi: 10.1002/advs.202203062 (PMC9839859; doi:10.1002/advs.202203062)

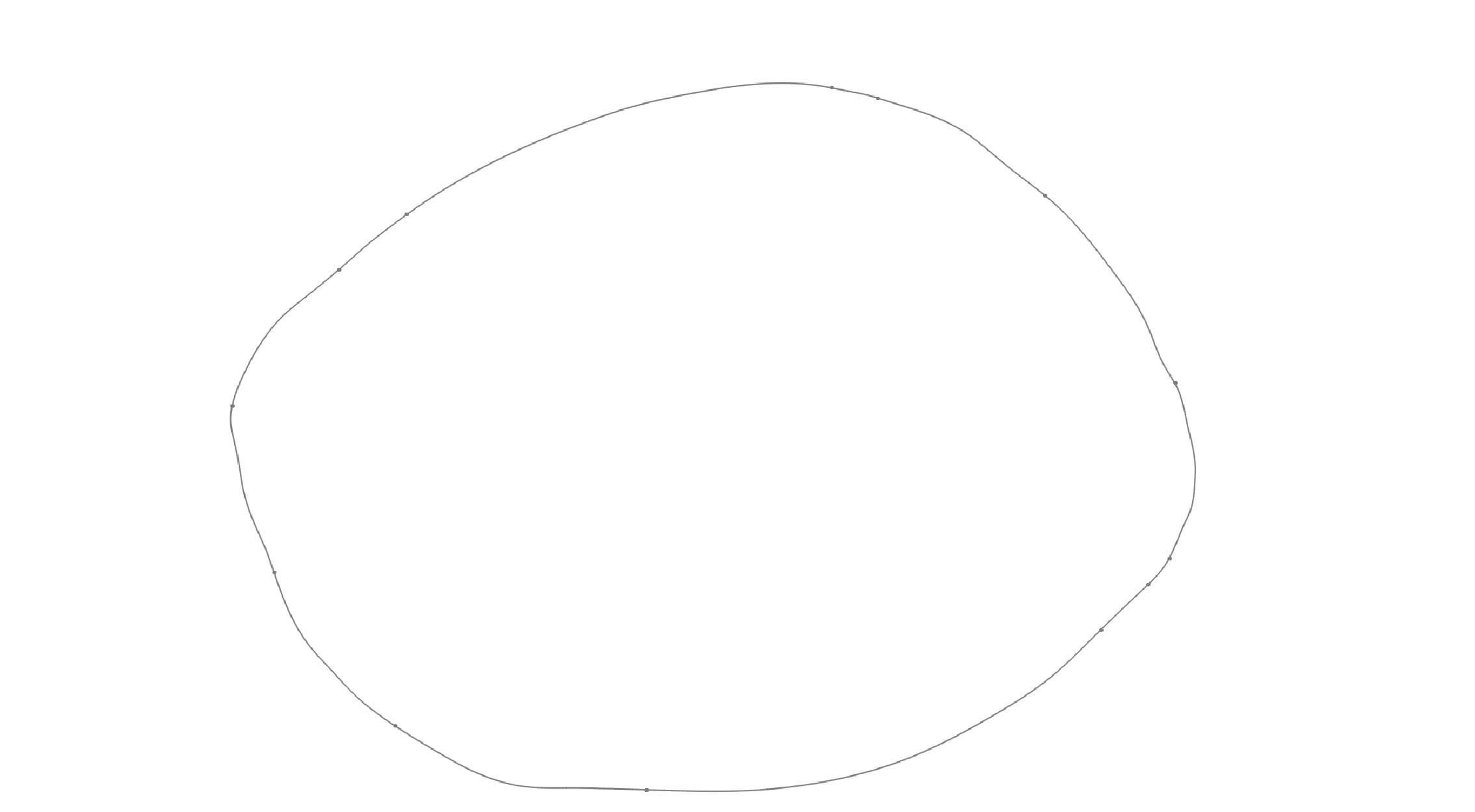

Supplement: Supplementary file 4 — Supporting Information [file ADVS-10-2203062-s013.zip › advs202203062-sup-0004-Supplementary-DataS3/Supplementary Data S3/19.jpg]

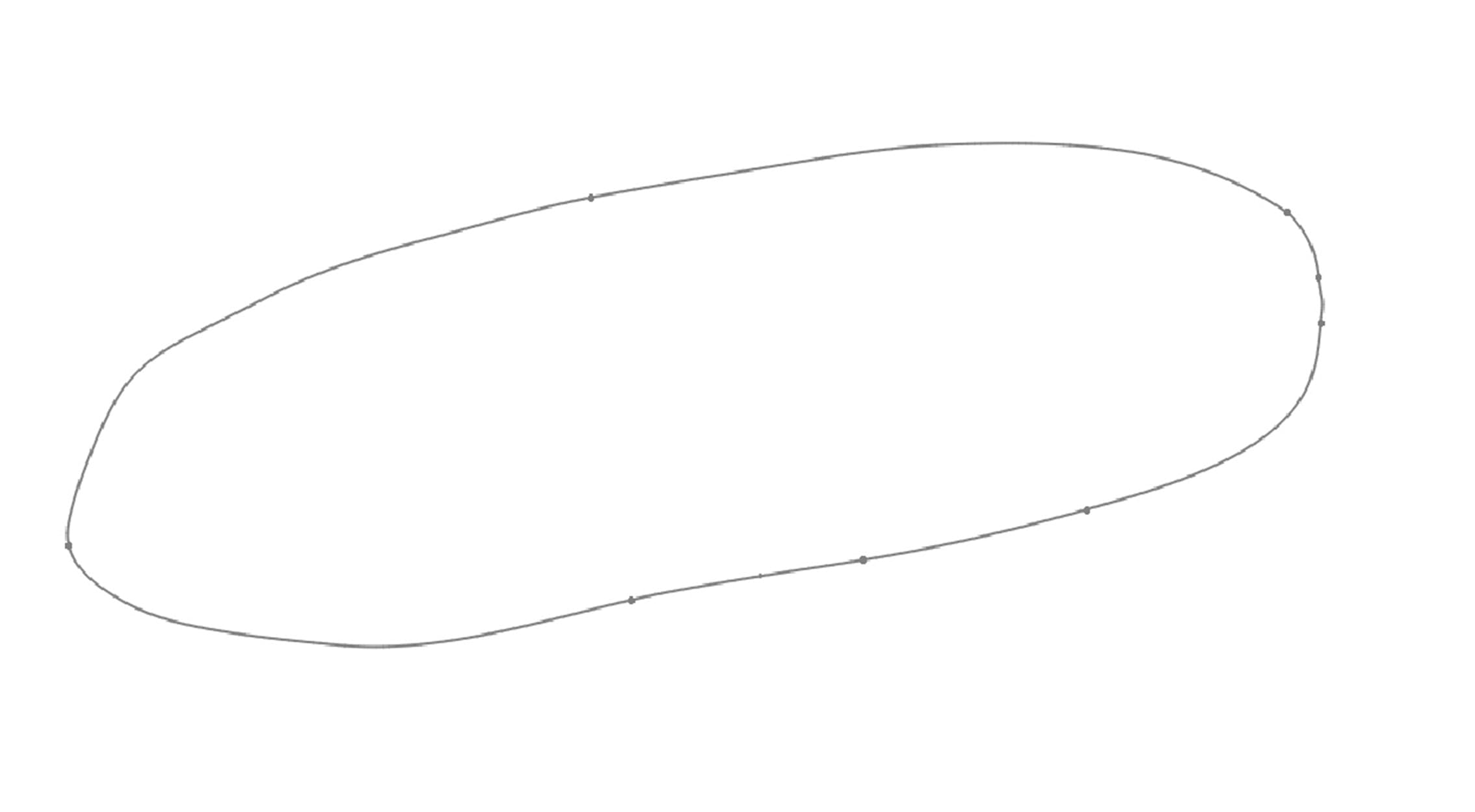

Supplement: Supplementary file 4 — Supporting Information [file ADVS-10-2203062-s013.zip › advs202203062-sup-0004-Supplementary-DataS3/Supplementary Data S3/190.jpg]

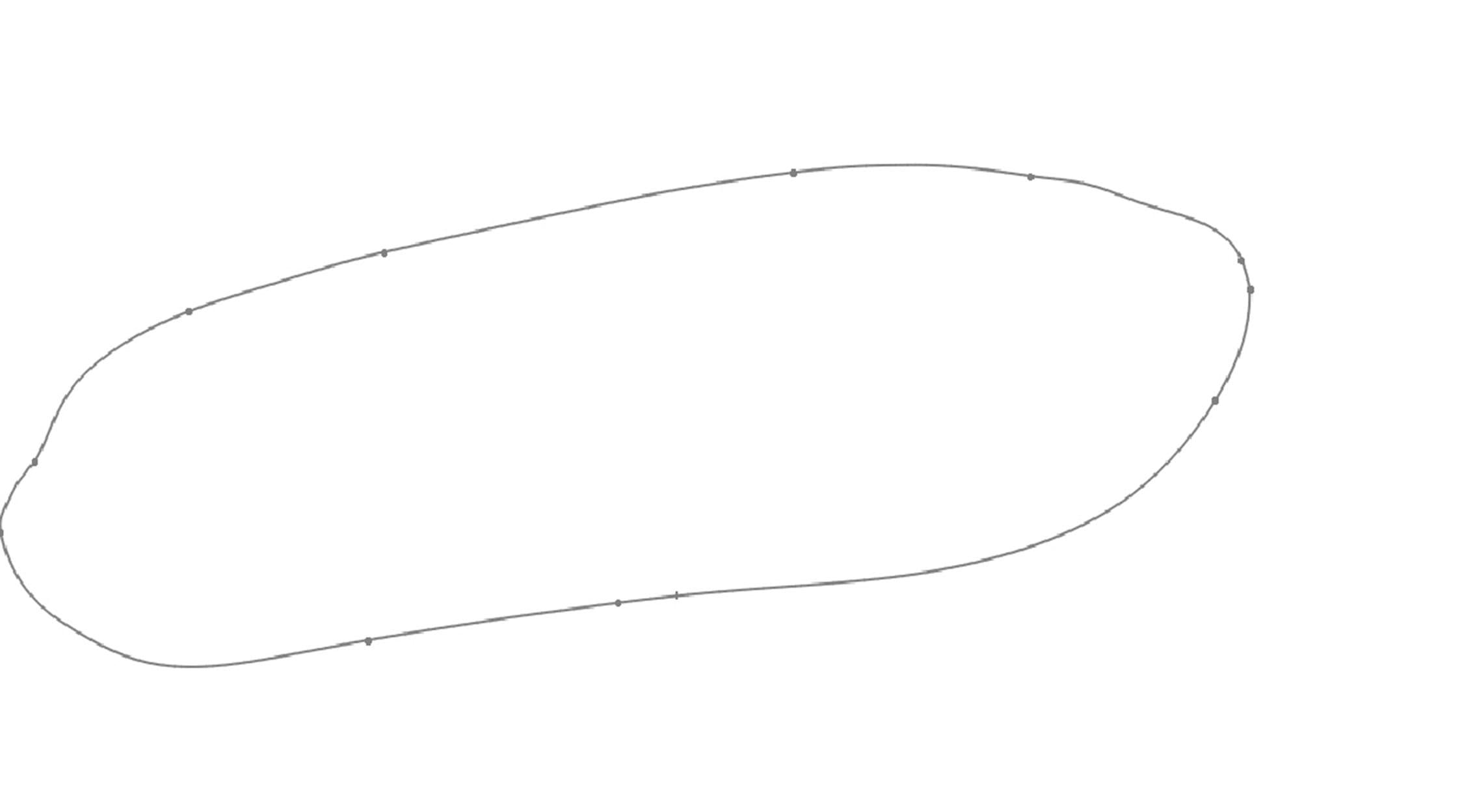

Supplement: Supplementary file 4 — Supporting Information [file ADVS-10-2203062-s013.zip › advs202203062-sup-0004-Supplementary-DataS3/Supplementary Data S3/191.jpg]

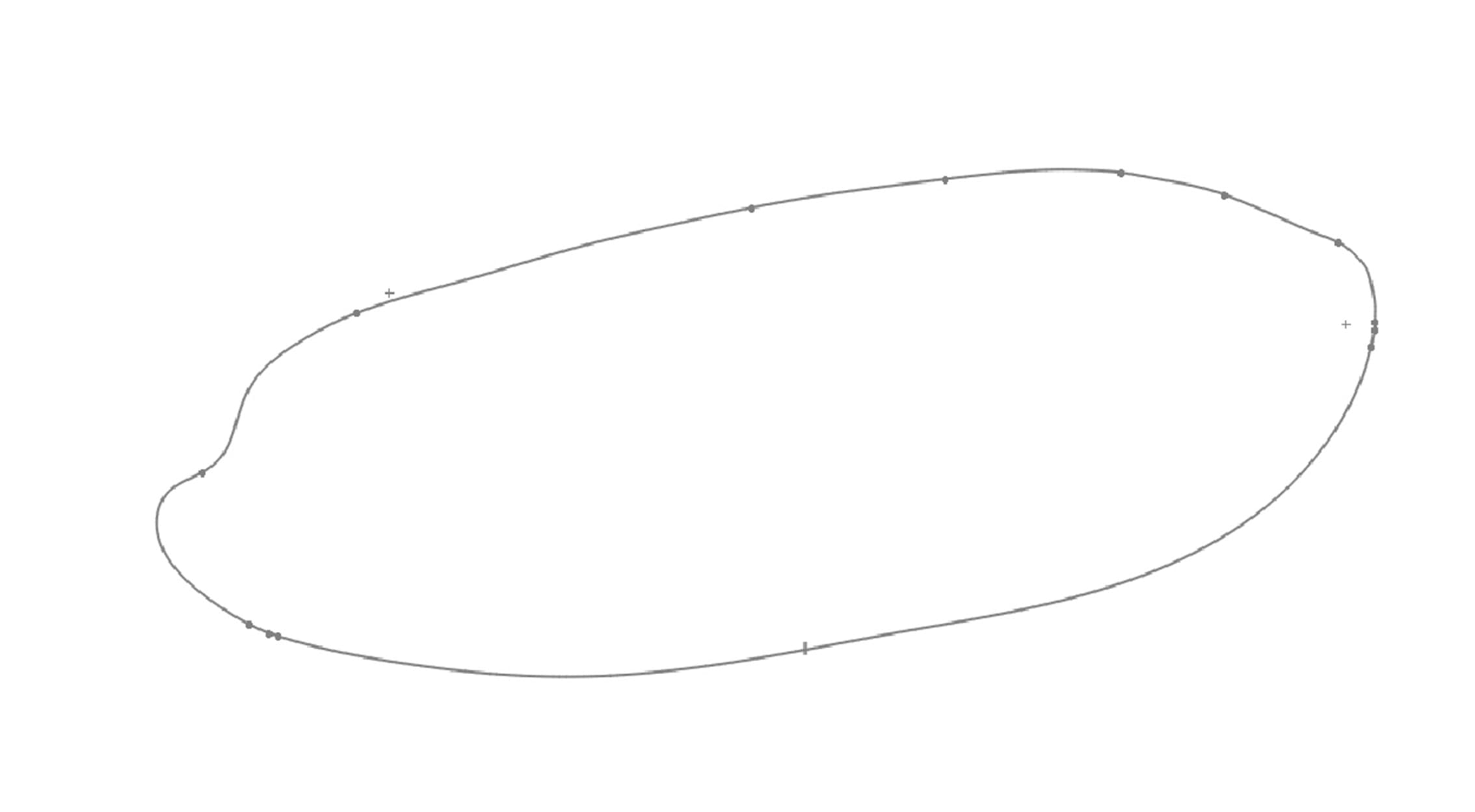

Supplement: Supplementary file 4 — Supporting Information [file ADVS-10-2203062-s013.zip › advs202203062-sup-0004-Supplementary-DataS3/Supplementary Data S3/192.jpg]

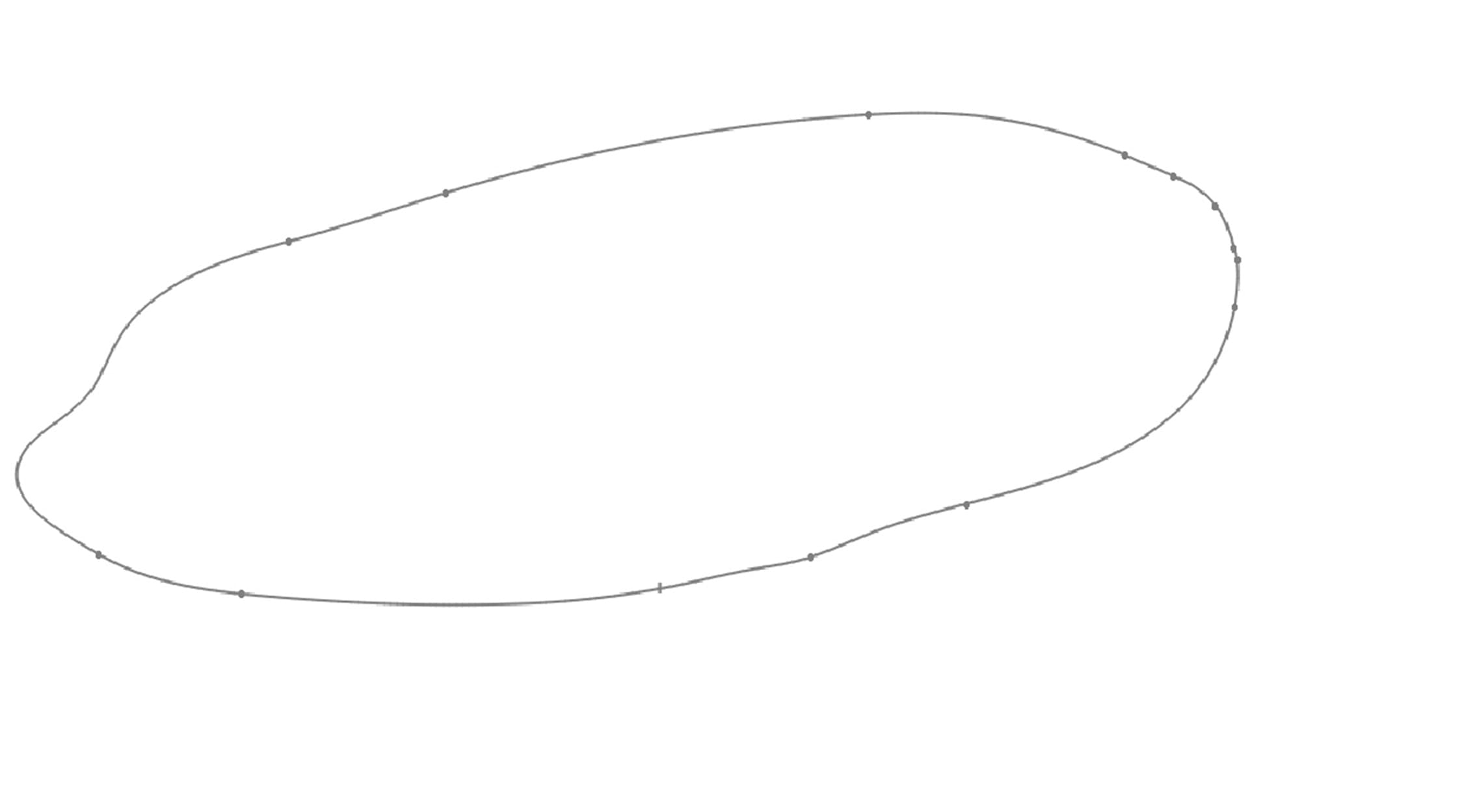

Supplement: Supplementary file 4 — Supporting Information [file ADVS-10-2203062-s013.zip › advs202203062-sup-0004-Supplementary-DataS3/Supplementary Data S3/193.jpg]

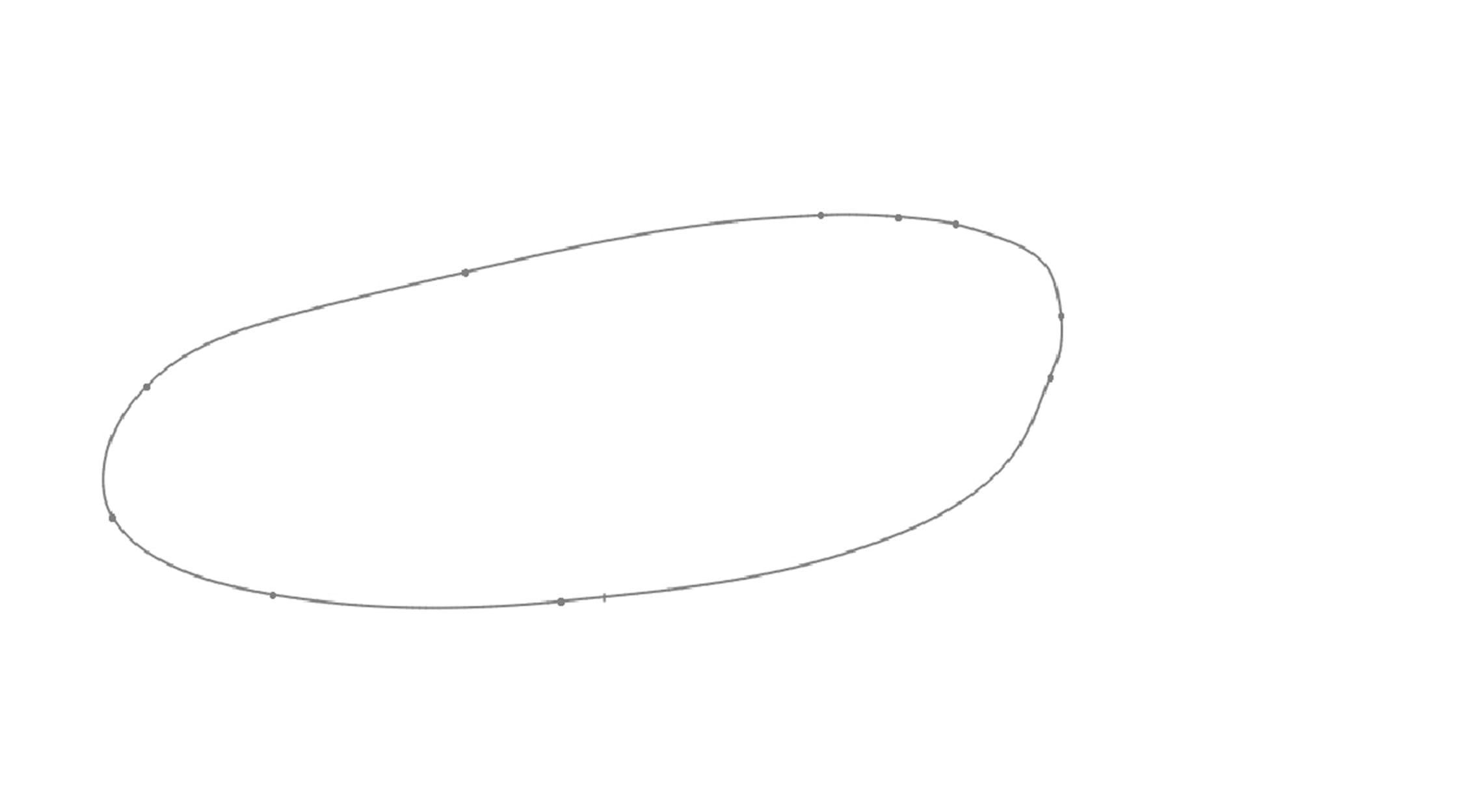

Supplement: Supplementary file 4 — Supporting Information [file ADVS-10-2203062-s013.zip › advs202203062-sup-0004-Supplementary-DataS3/Supplementary Data S3/194.jpg]

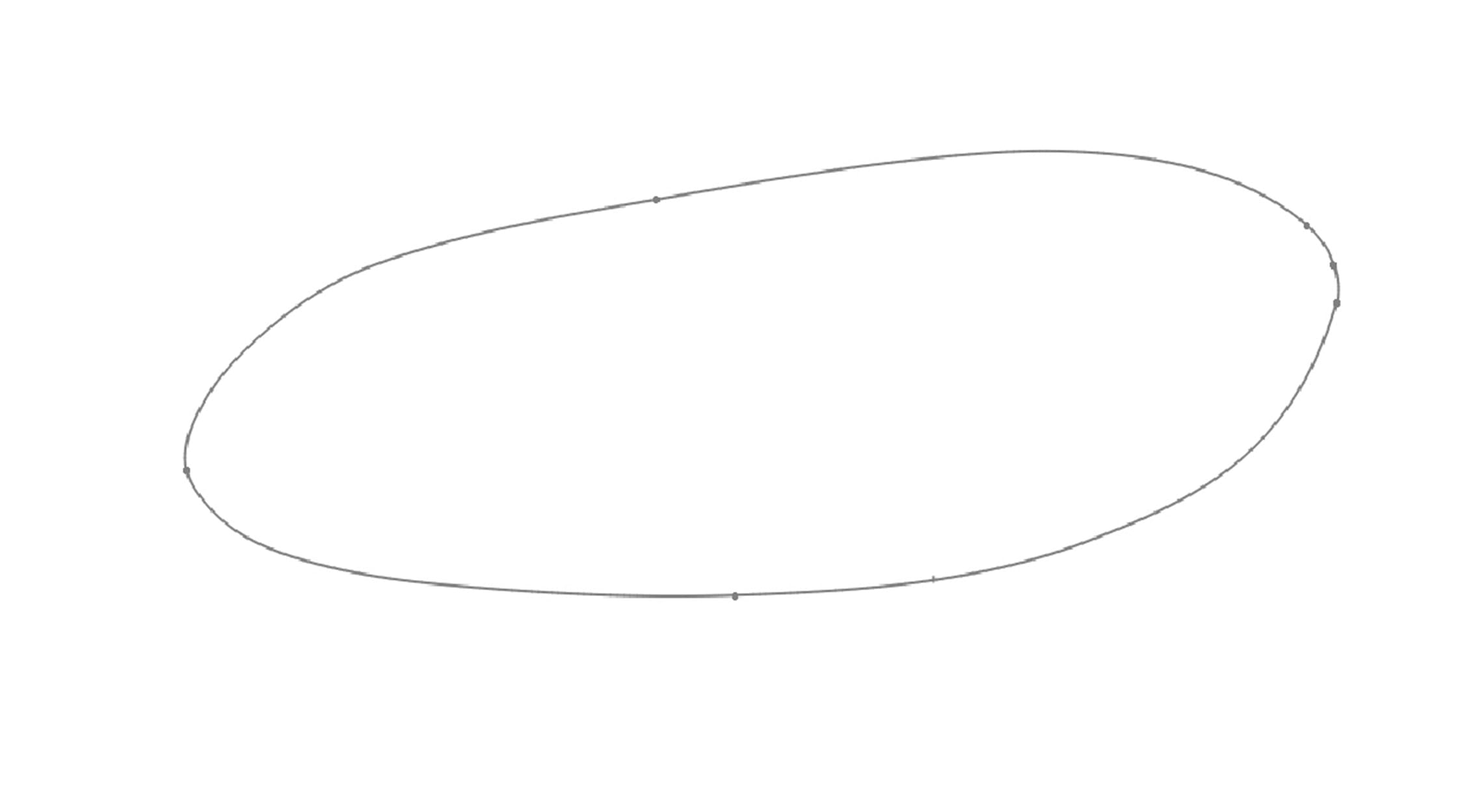

Supplement: Supplementary file 4 — Supporting Information [file ADVS-10-2203062-s013.zip › advs202203062-sup-0004-Supplementary-DataS3/Supplementary Data S3/195.jpg]

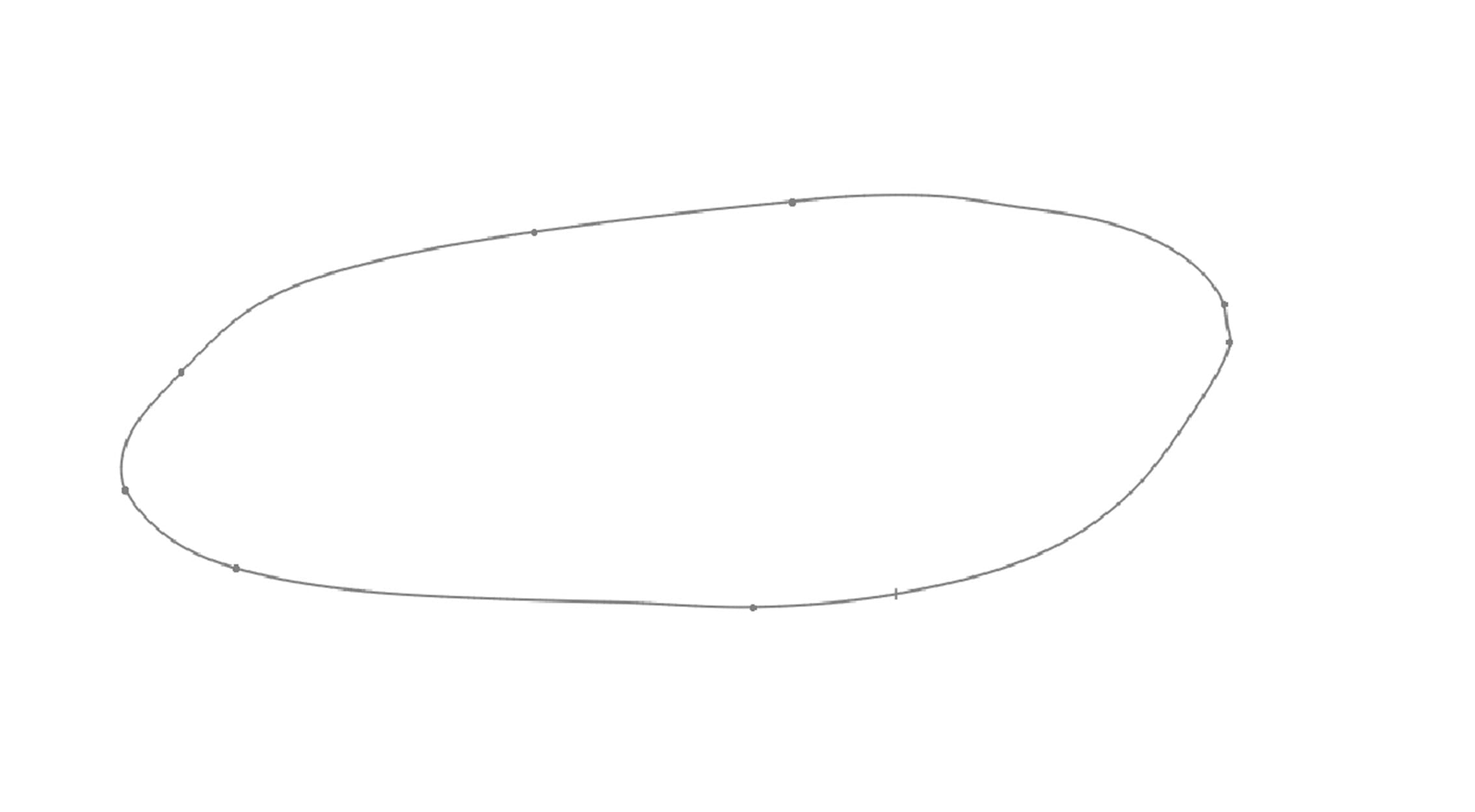

Supplement: Supplementary file 4 — Supporting Information [file ADVS-10-2203062-s013.zip › advs202203062-sup-0004-Supplementary-DataS3/Supplementary Data S3/196.jpg]

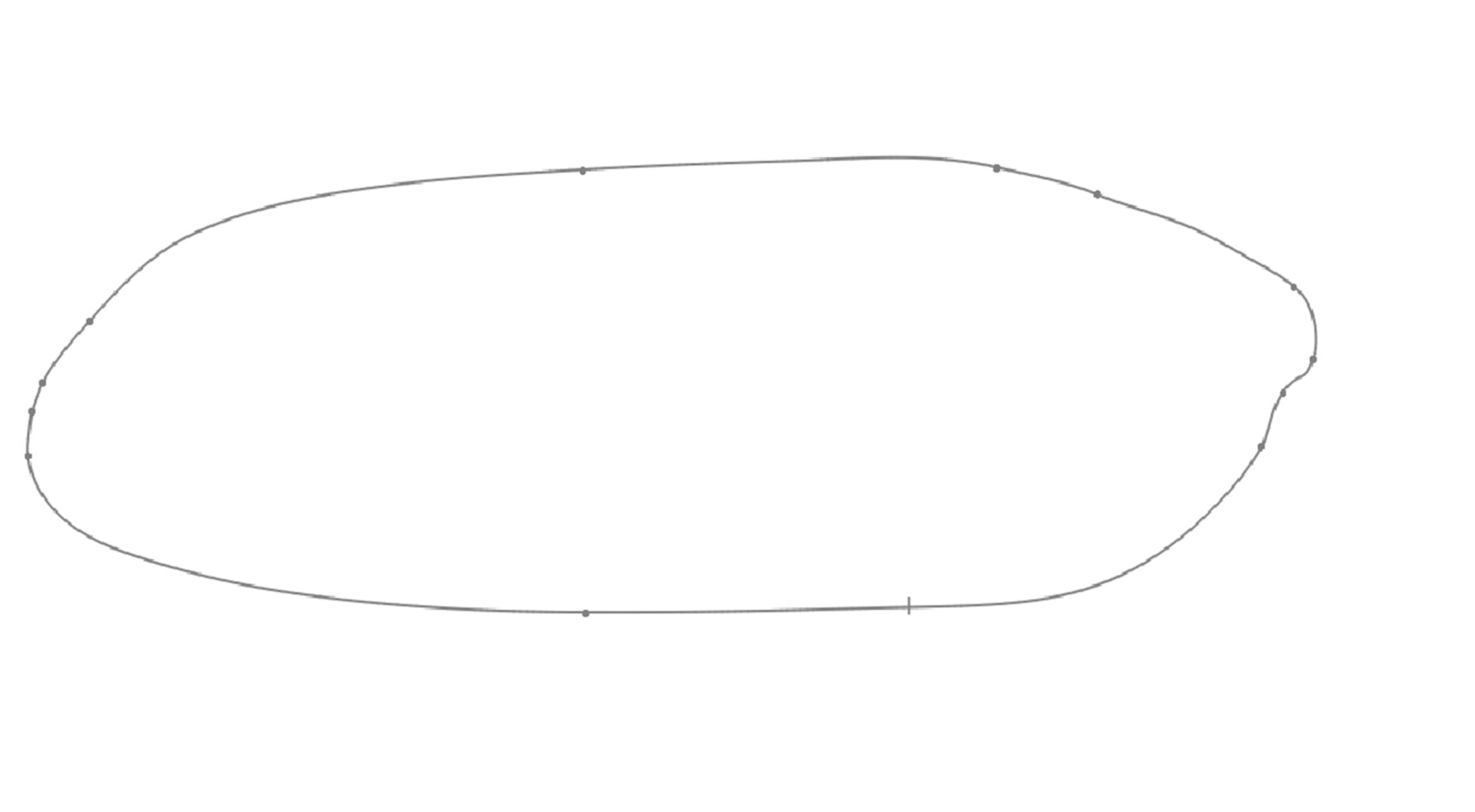

Supplement: Supplementary file 4 — Supporting Information [file ADVS-10-2203062-s013.zip › advs202203062-sup-0004-Supplementary-DataS3/Supplementary Data S3/197.jpg]

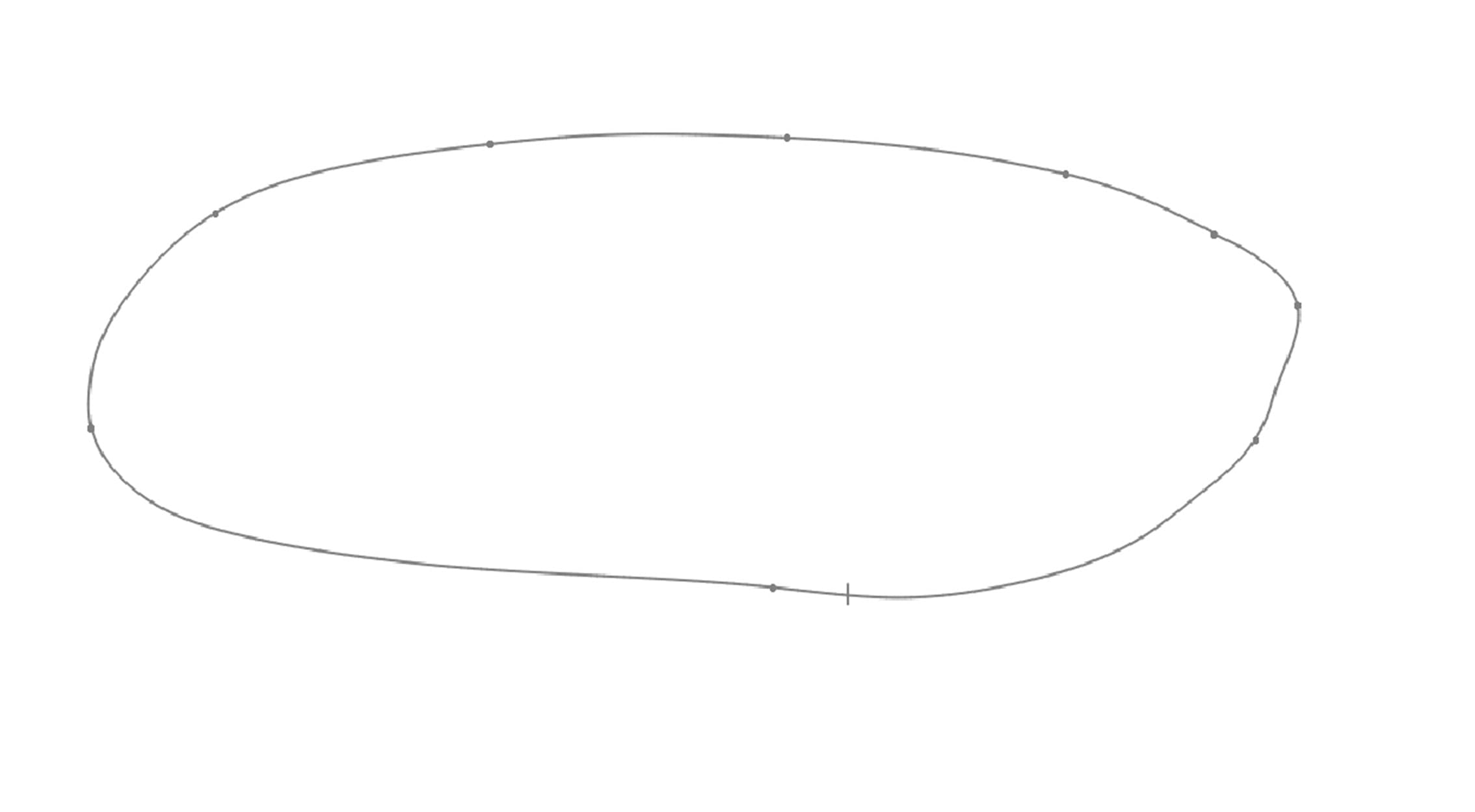

Supplement: Supplementary file 4 — Supporting Information [file ADVS-10-2203062-s013.zip › advs202203062-sup-0004-Supplementary-DataS3/Supplementary Data S3/198.jpg]

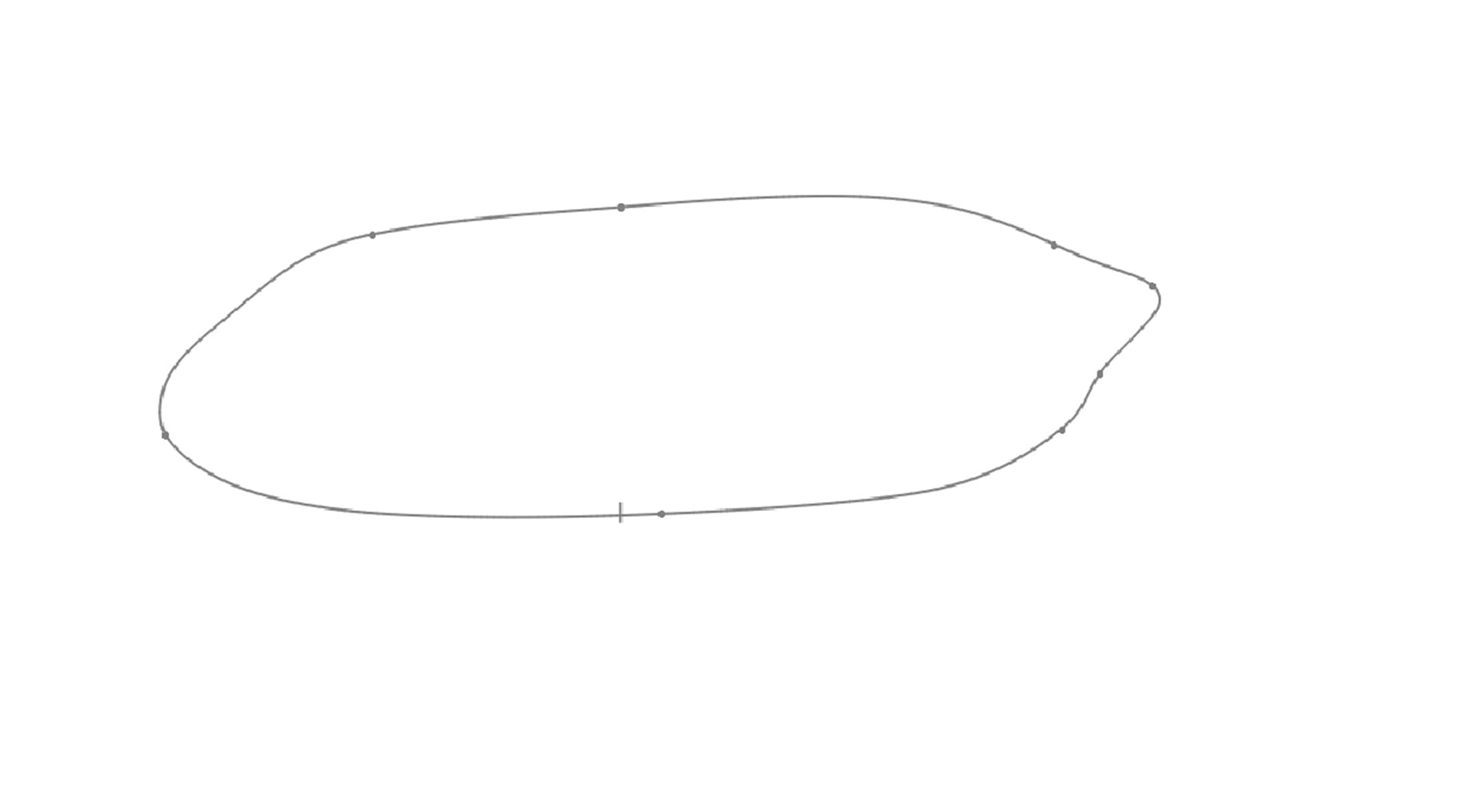

Supplement: Supplementary file 4 — Supporting Information [file ADVS-10-2203062-s013.zip › advs202203062-sup-0004-Supplementary-DataS3/Supplementary Data S3/199.jpg]

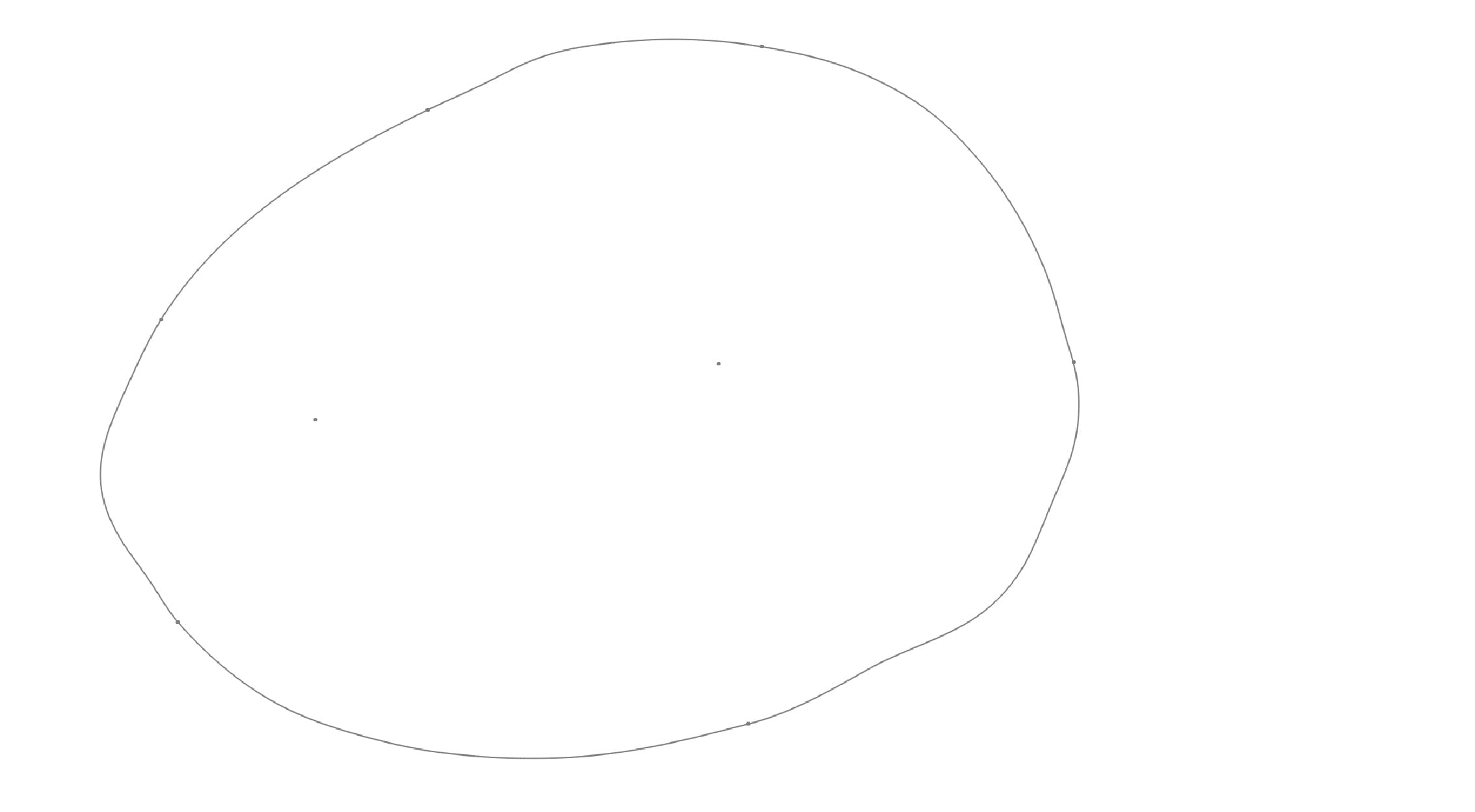

Supplement: Supplementary file 4 — Supporting Information [file ADVS-10-2203062-s013.zip › advs202203062-sup-0004-Supplementary-DataS3/Supplementary Data S3/2.jpg]

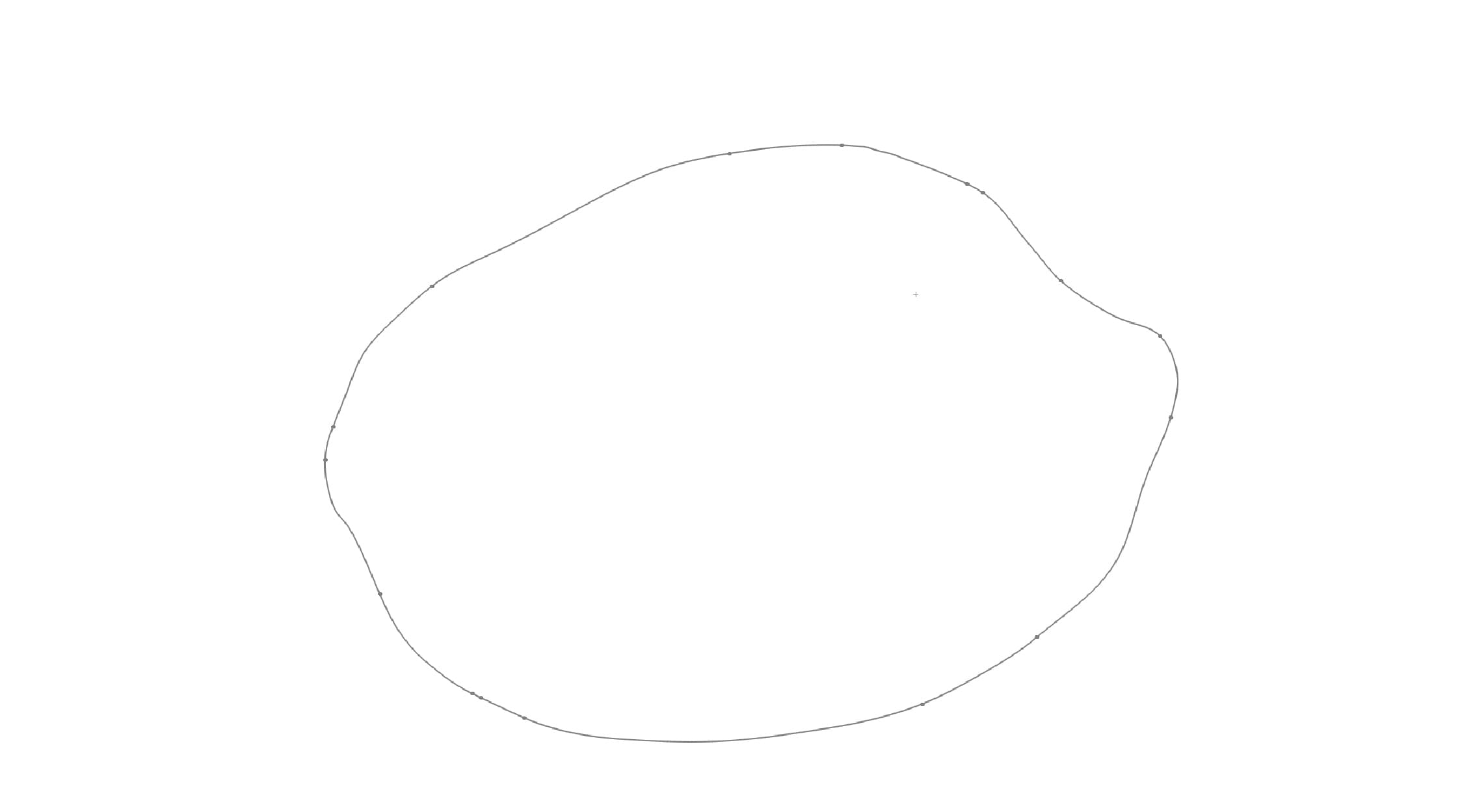

Supplement: Supplementary file 4 — Supporting Information [file ADVS-10-2203062-s013.zip › advs202203062-sup-0004-Supplementary-DataS3/Supplementary Data S3/20.jpg]

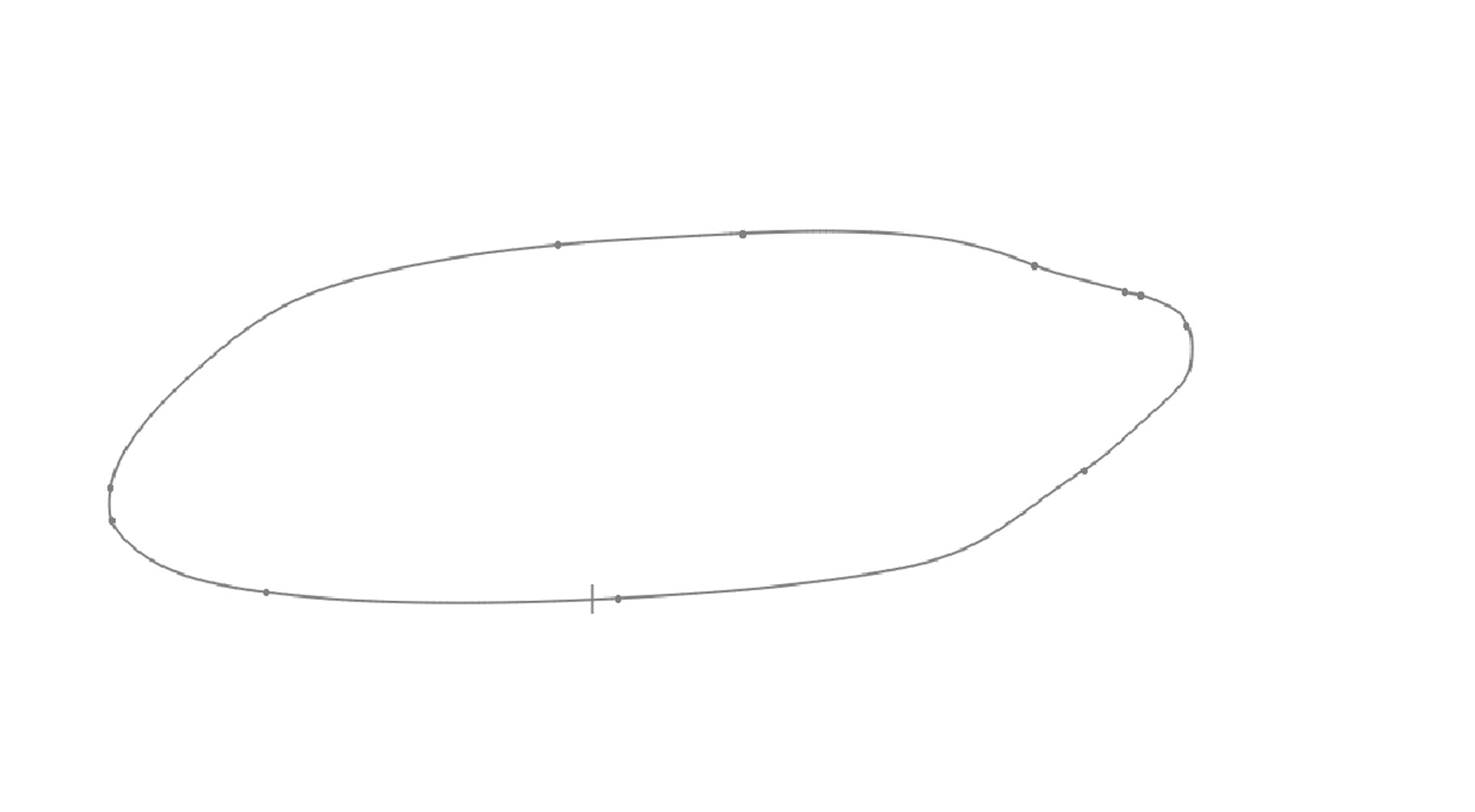

Supplement: Supplementary file 4 — Supporting Information [file ADVS-10-2203062-s013.zip › advs202203062-sup-0004-Supplementary-DataS3/Supplementary Data S3/200.jpg]

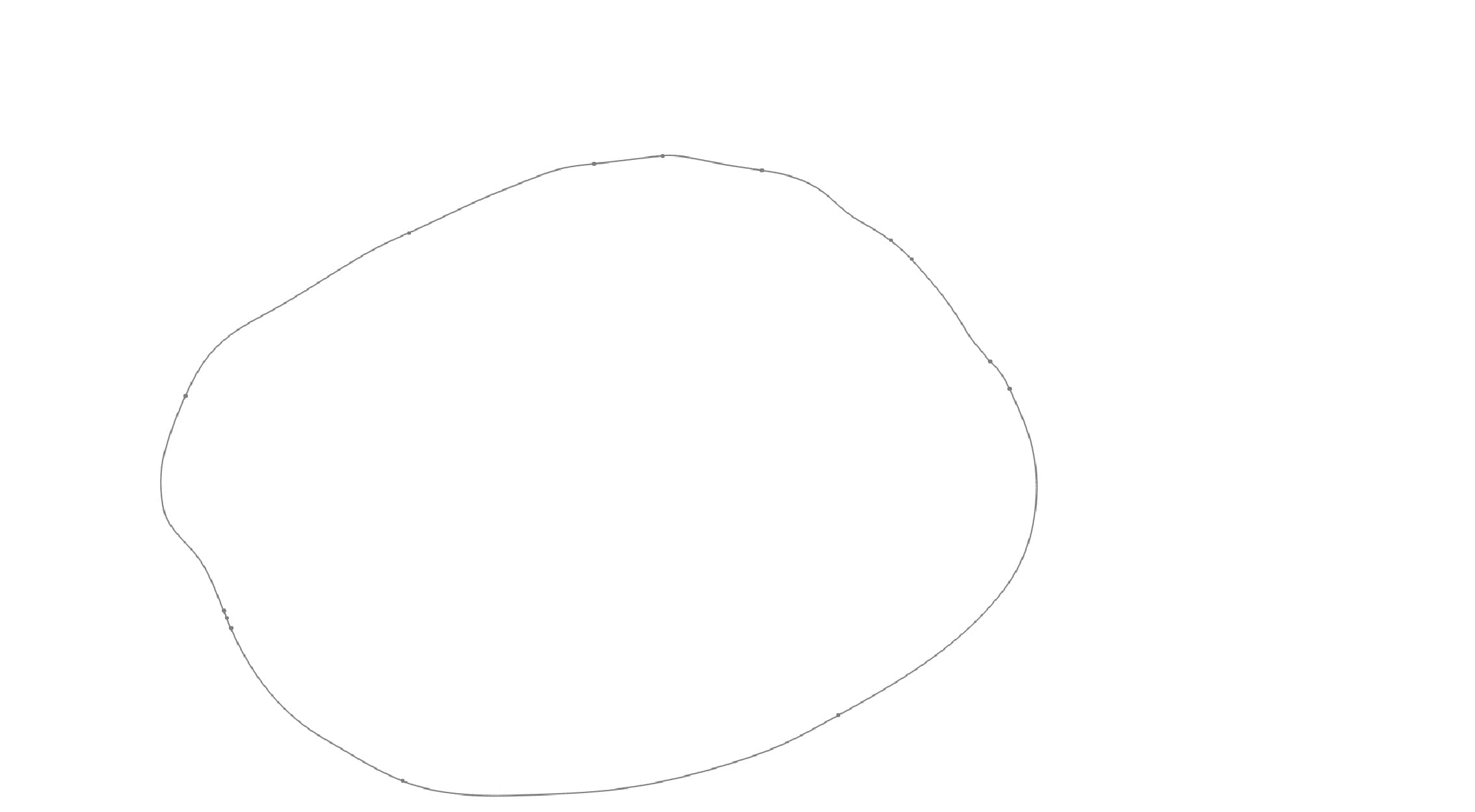

Supplement: Supplementary file 4 — Supporting Information [file ADVS-10-2203062-s013.zip › advs202203062-sup-0004-Supplementary-DataS3/Supplementary Data S3/21.jpg]

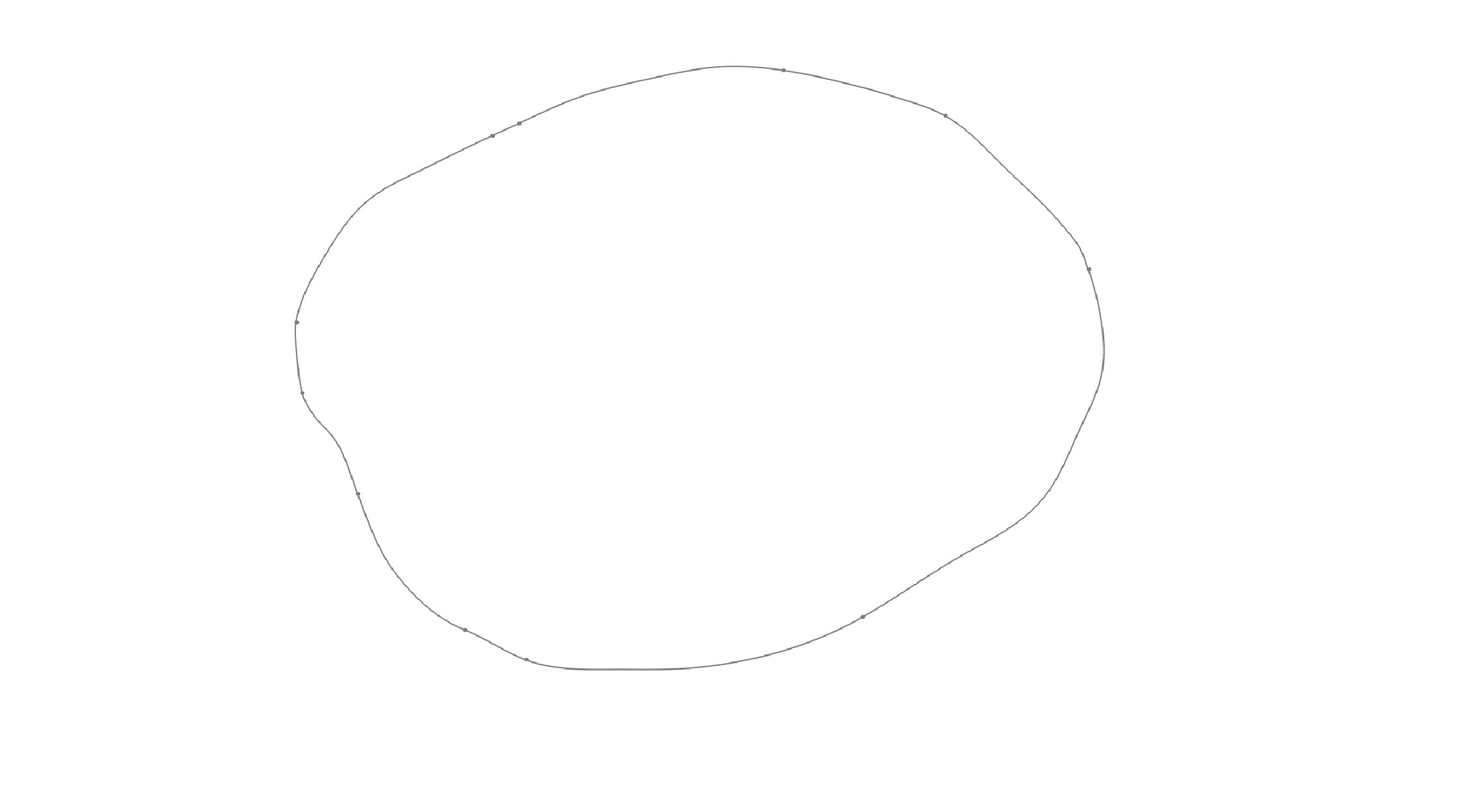

Supplement: Supplementary file 4 — Supporting Information [file ADVS-10-2203062-s013.zip › advs202203062-sup-0004-Supplementary-DataS3/Supplementary Data S3/22.jpg]

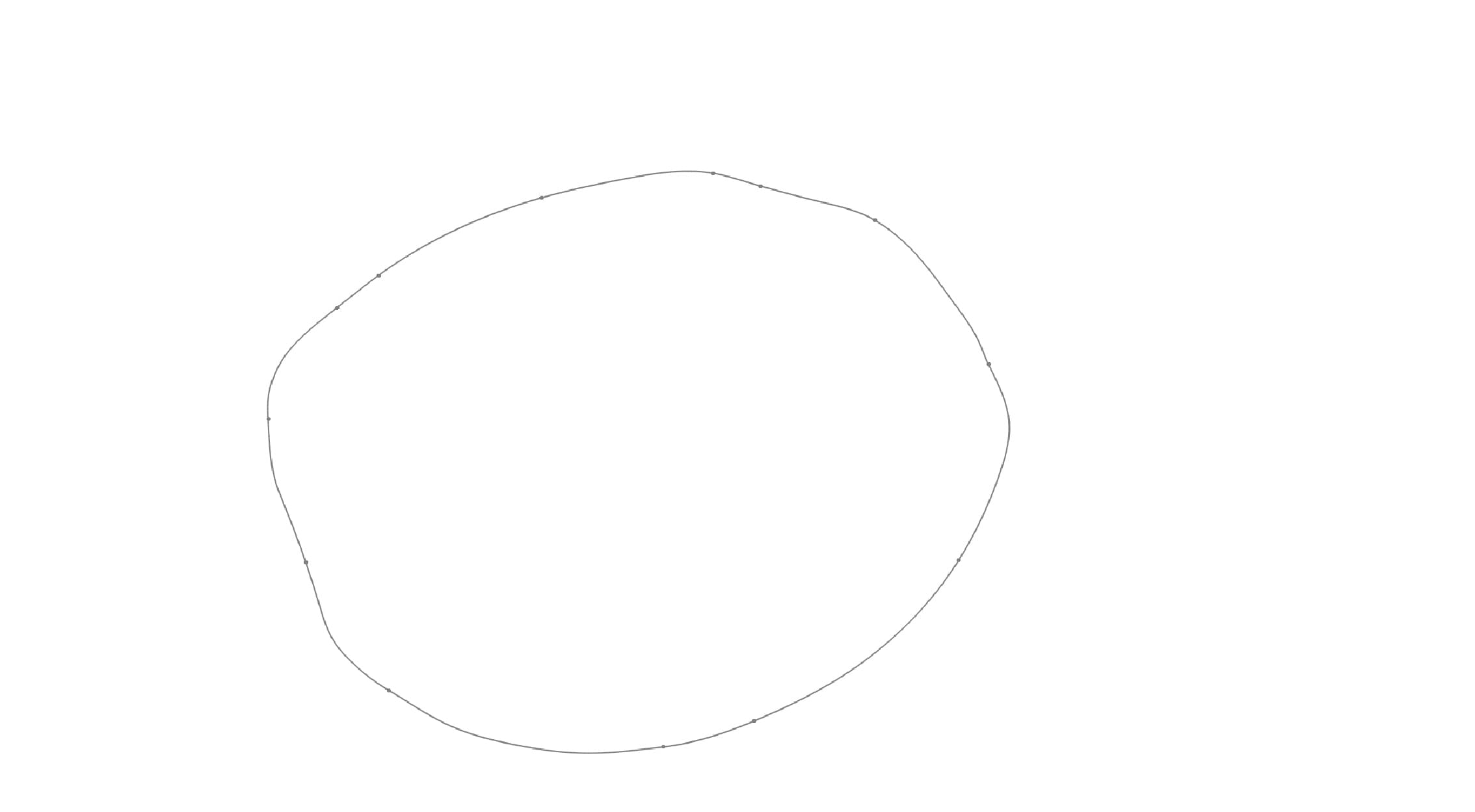

Supplement: Supplementary file 4 — Supporting Information [file ADVS-10-2203062-s013.zip › advs202203062-sup-0004-Supplementary-DataS3/Supplementary Data S3/23.jpg]

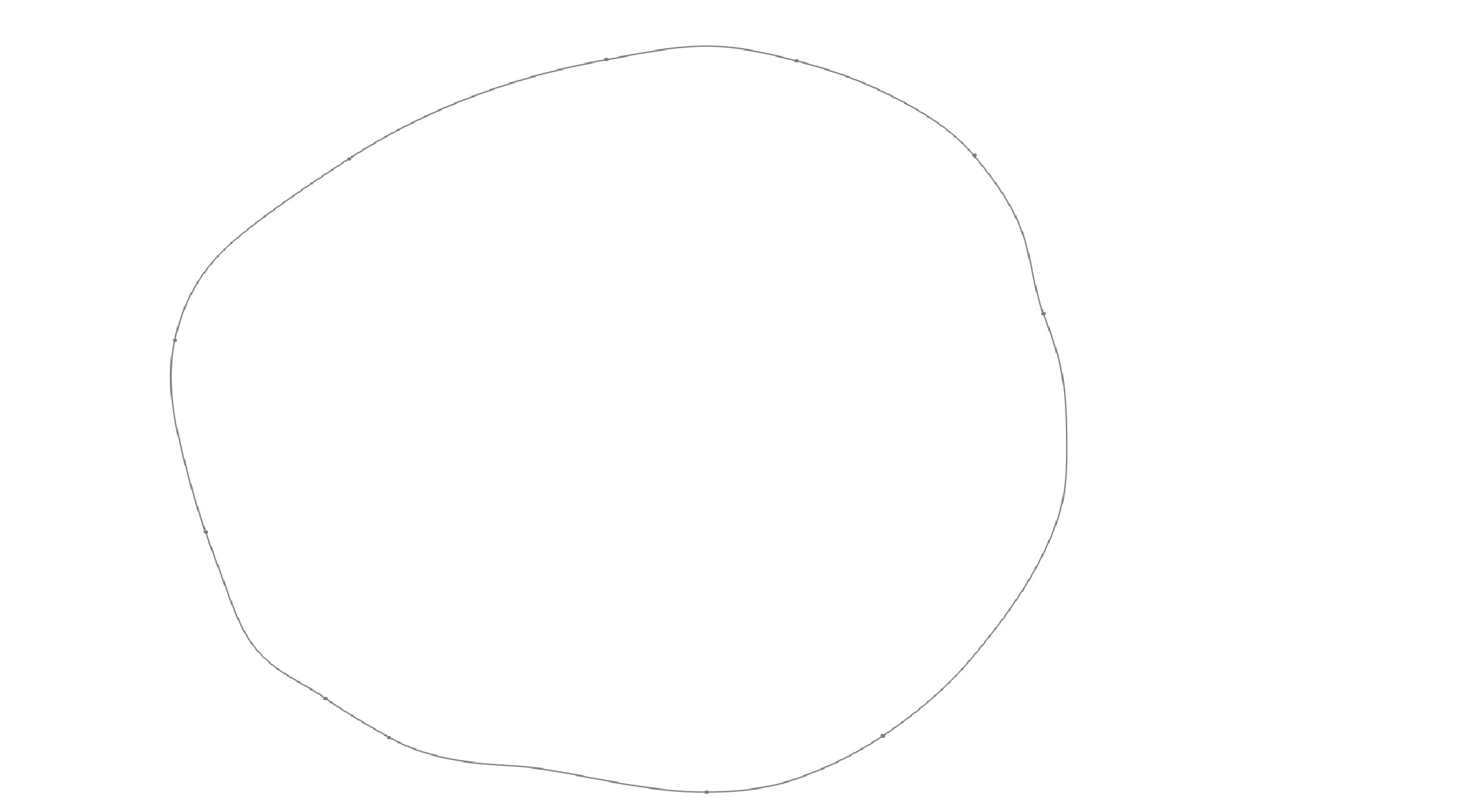

Supplement: Supplementary file 4 — Supporting Information [file ADVS-10-2203062-s013.zip › advs202203062-sup-0004-Supplementary-DataS3/Supplementary Data S3/24.jpg]

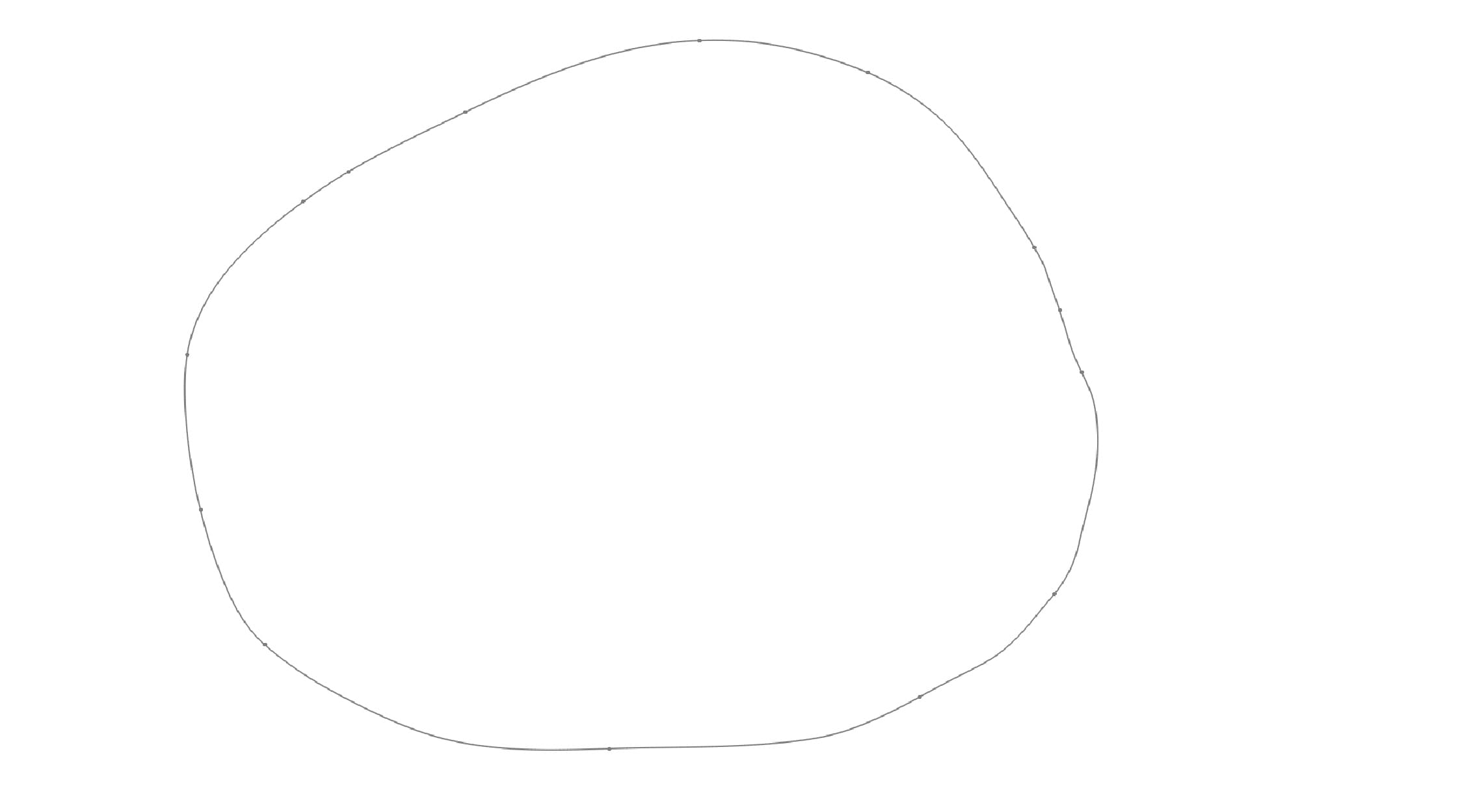

Supplement: Supplementary file 4 — Supporting Information [file ADVS-10-2203062-s013.zip › advs202203062-sup-0004-Supplementary-DataS3/Supplementary Data S3/25.jpg]

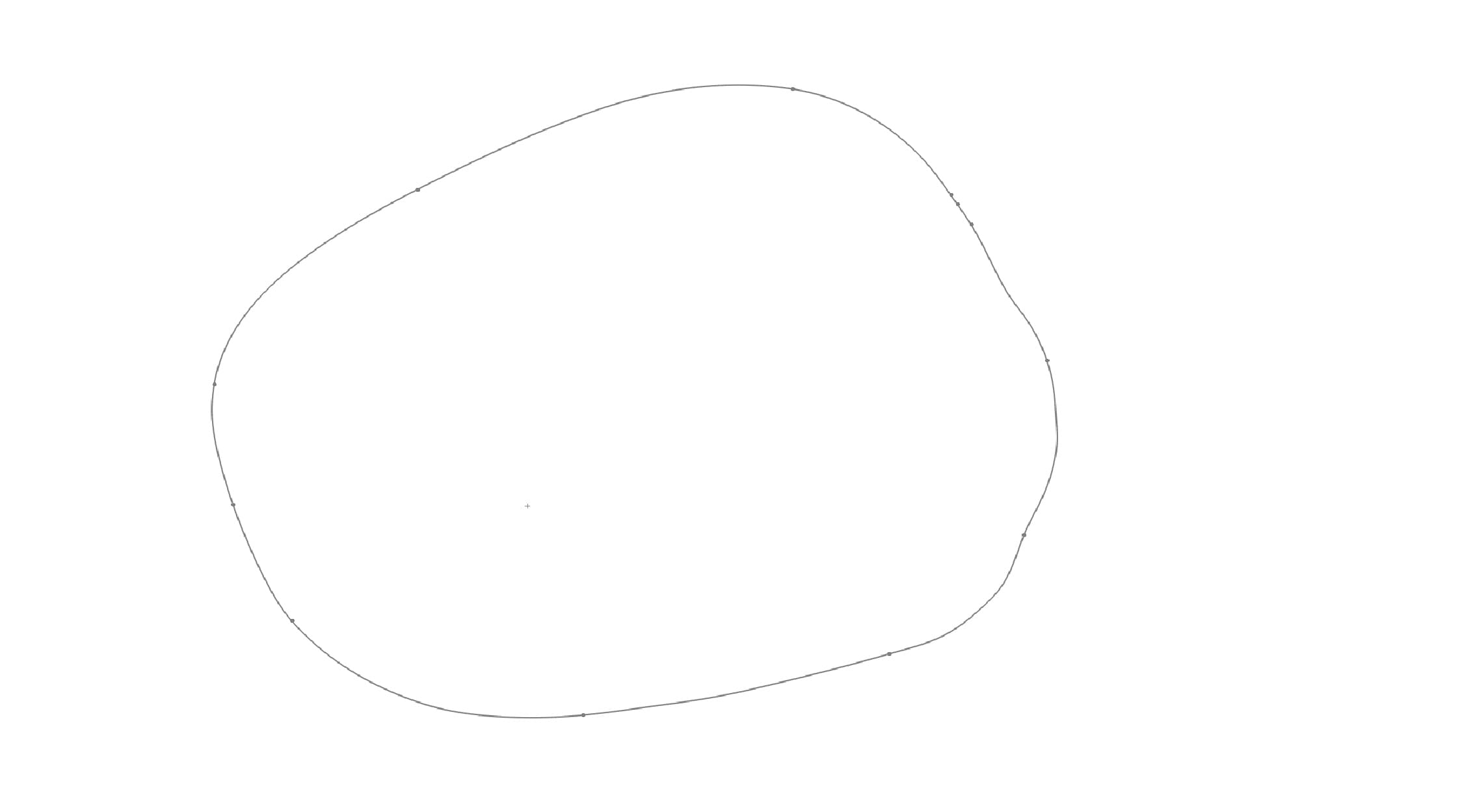

Supplement: Supplementary file 4 — Supporting Information [file ADVS-10-2203062-s013.zip › advs202203062-sup-0004-Supplementary-DataS3/Supplementary Data S3/26.jpg]

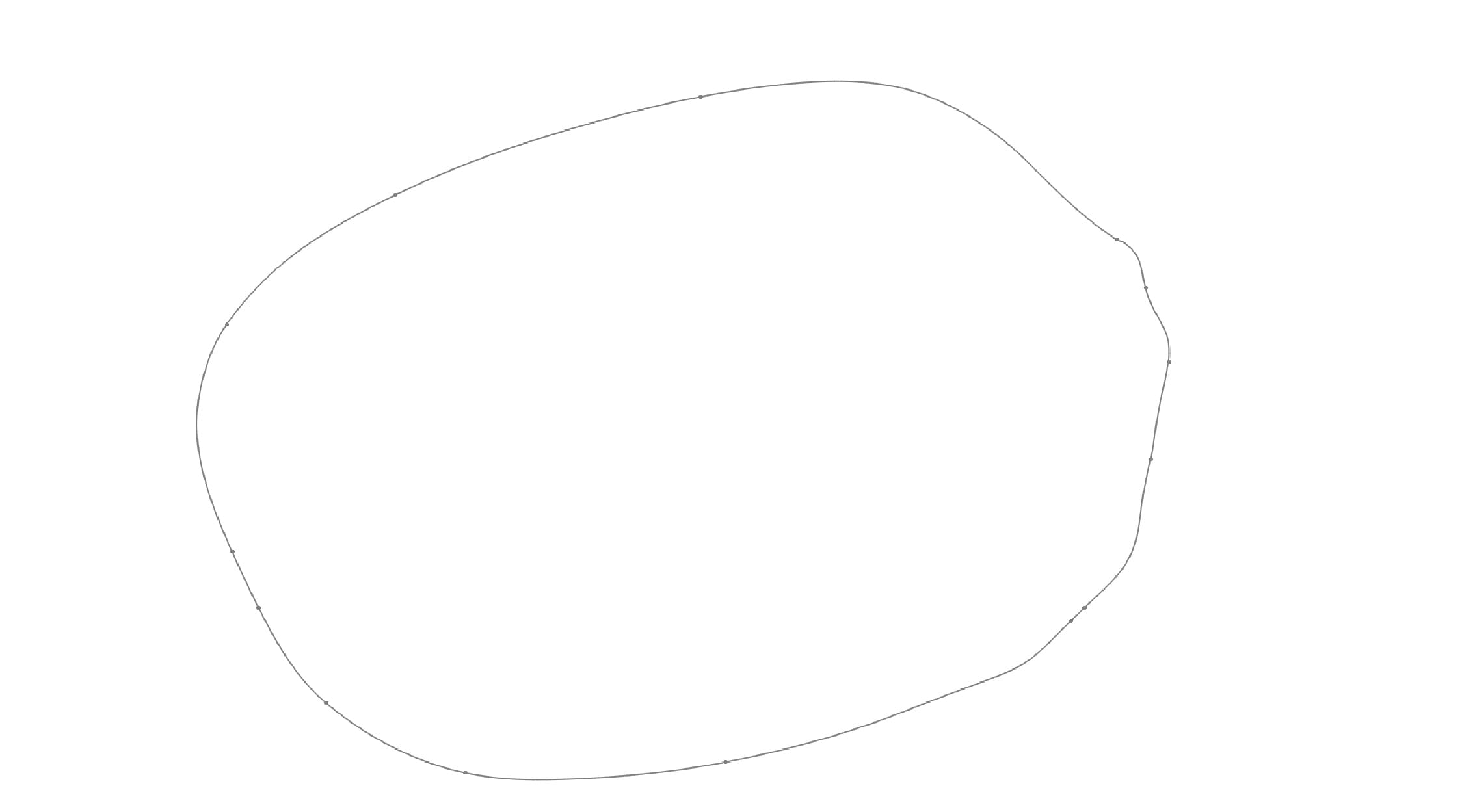

Supplement: Supplementary file 4 — Supporting Information [file ADVS-10-2203062-s013.zip › advs202203062-sup-0004-Supplementary-DataS3/Supplementary Data S3/27.jpg]

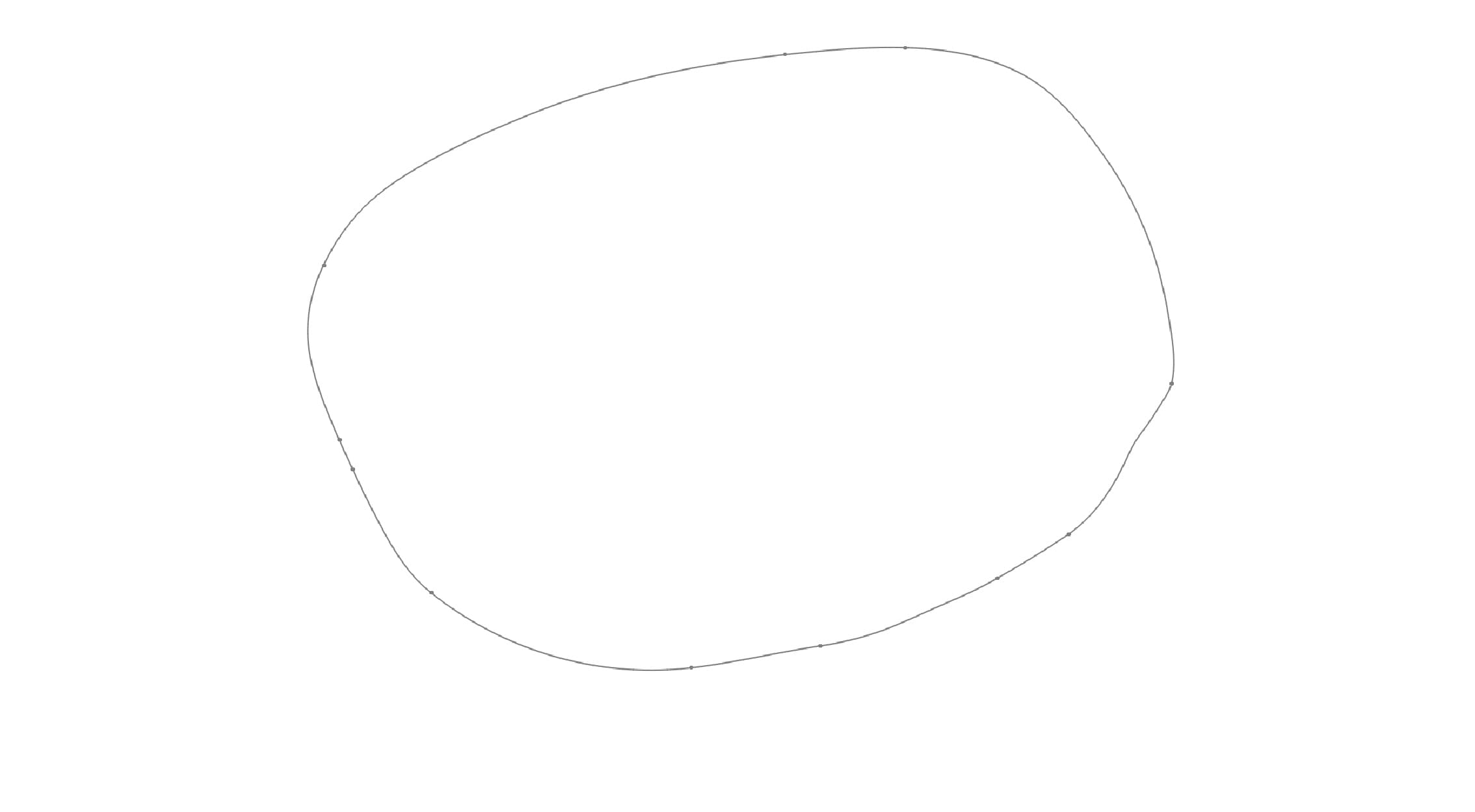

Supplement: Supplementary file 4 — Supporting Information [file ADVS-10-2203062-s013.zip › advs202203062-sup-0004-Supplementary-DataS3/Supplementary Data S3/28.jpg]

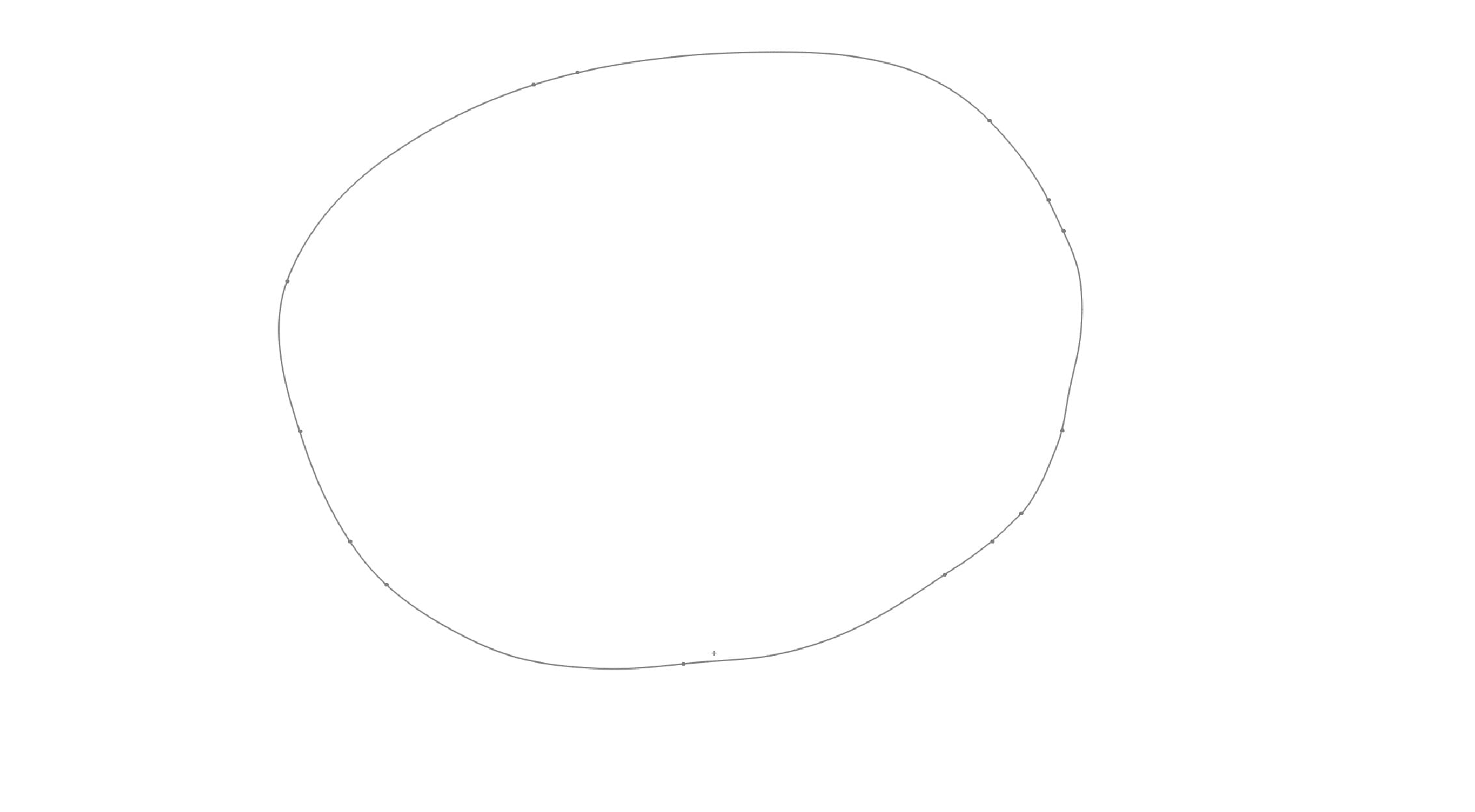

Supplement: Supplementary file 4 — Supporting Information [file ADVS-10-2203062-s013.zip › advs202203062-sup-0004-Supplementary-DataS3/Supplementary Data S3/29.jpg]

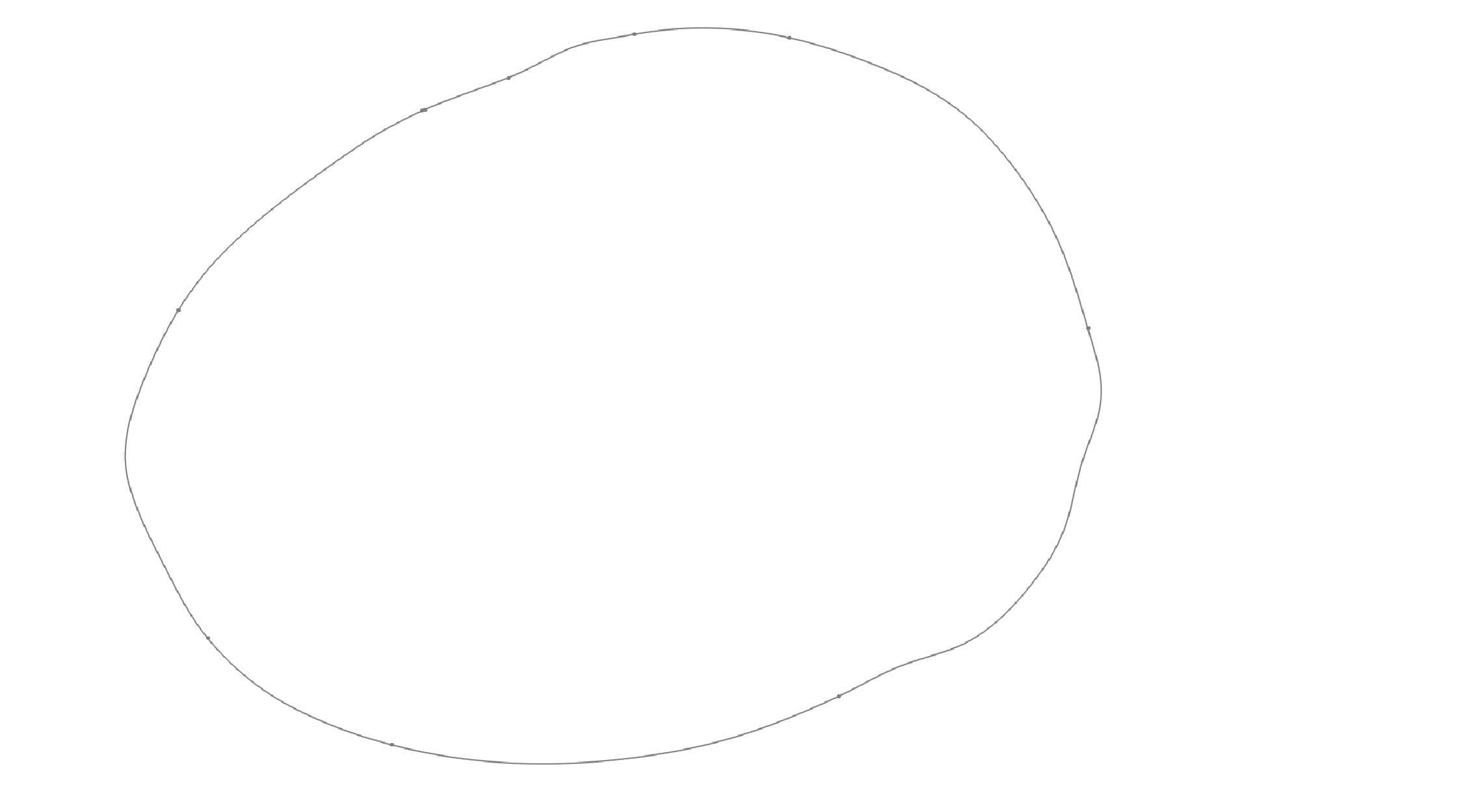

Supplement: Supplementary file 4 — Supporting Information [file ADVS-10-2203062-s013.zip › advs202203062-sup-0004-Supplementary-DataS3/Supplementary Data S3/3.jpg]

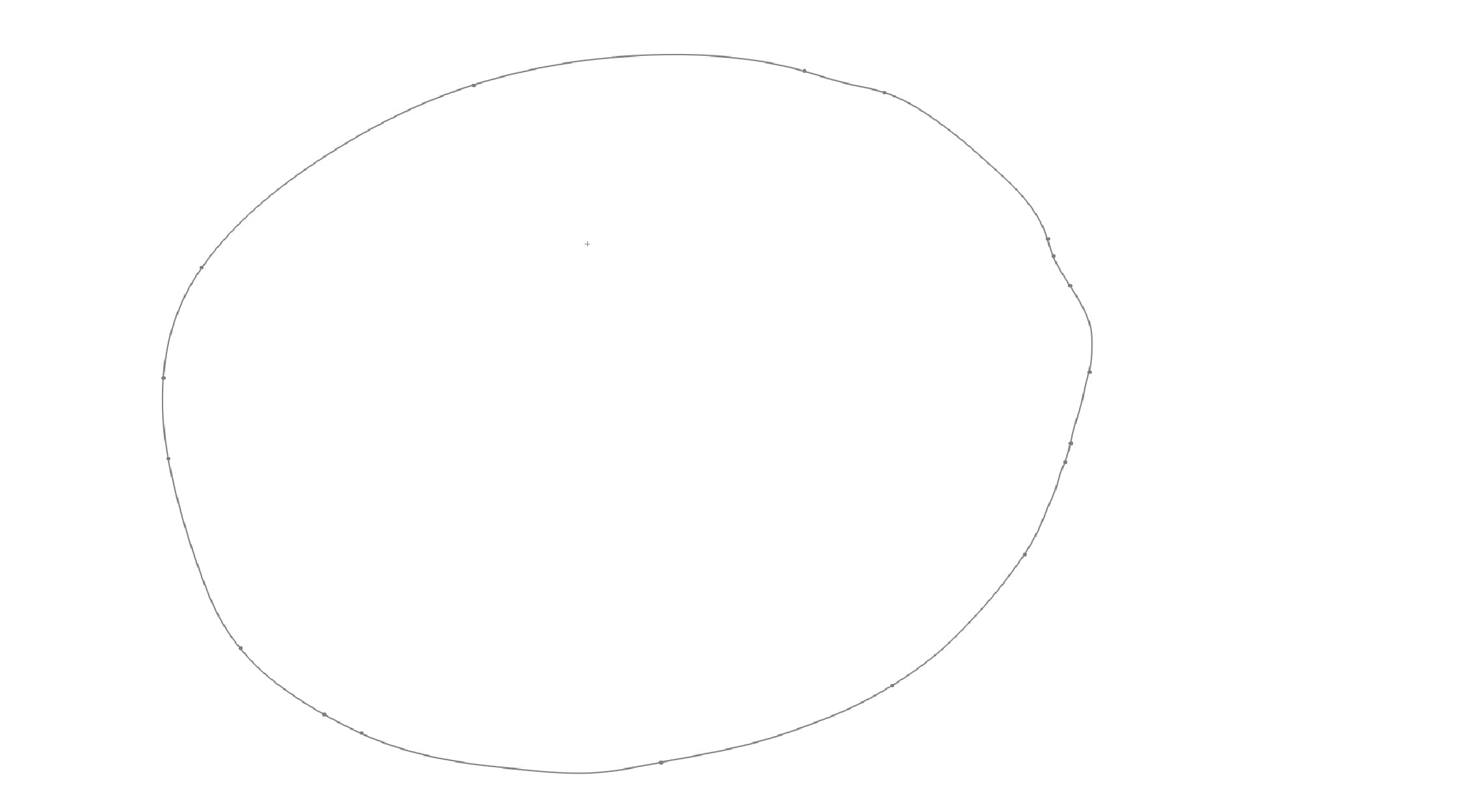

Supplement: Supplementary file 4 — Supporting Information [file ADVS-10-2203062-s013.zip › advs202203062-sup-0004-Supplementary-DataS3/Supplementary Data S3/30.jpg]

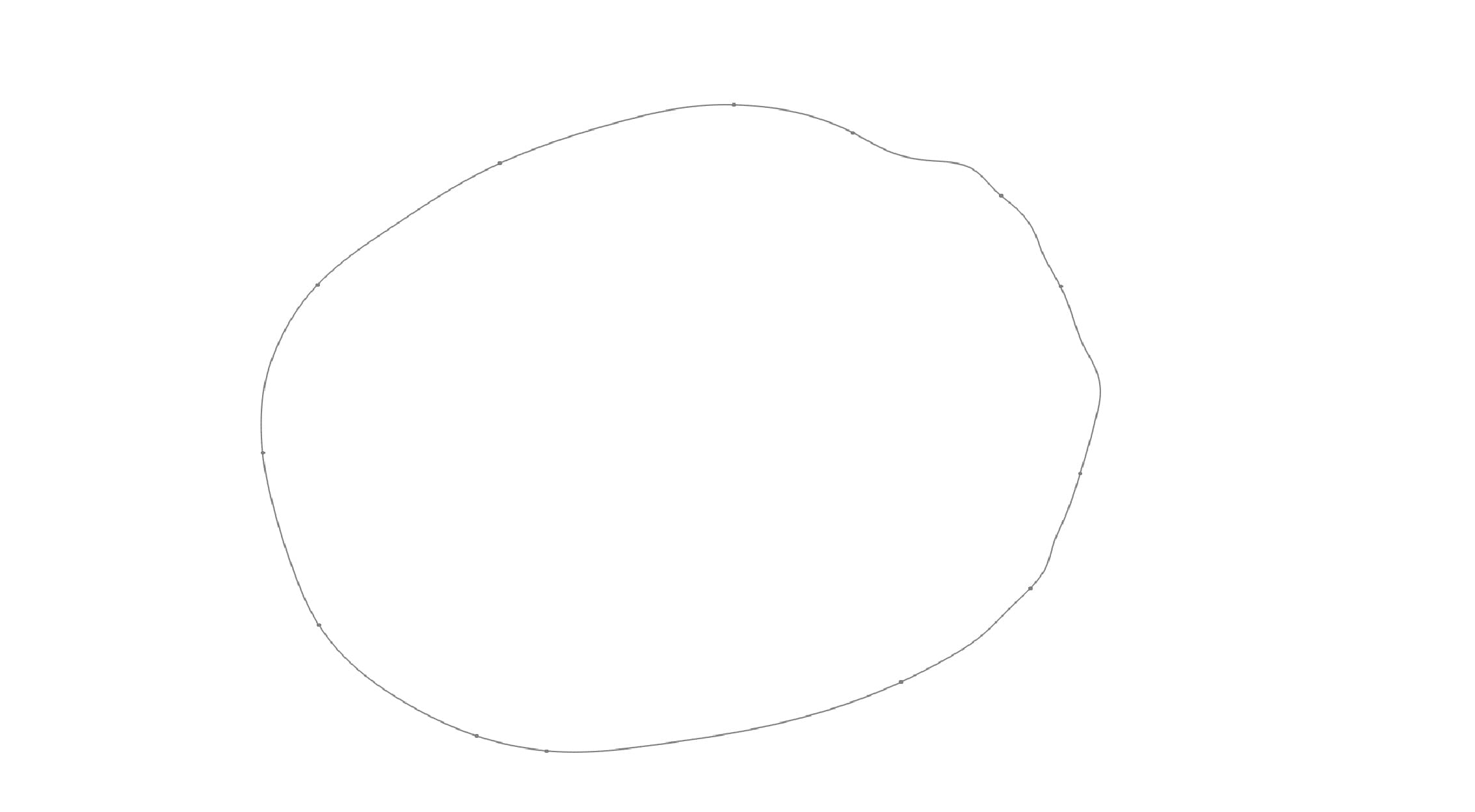

Supplement: Supplementary file 4 — Supporting Information [file ADVS-10-2203062-s013.zip › advs202203062-sup-0004-Supplementary-DataS3/Supplementary Data S3/31.jpg]

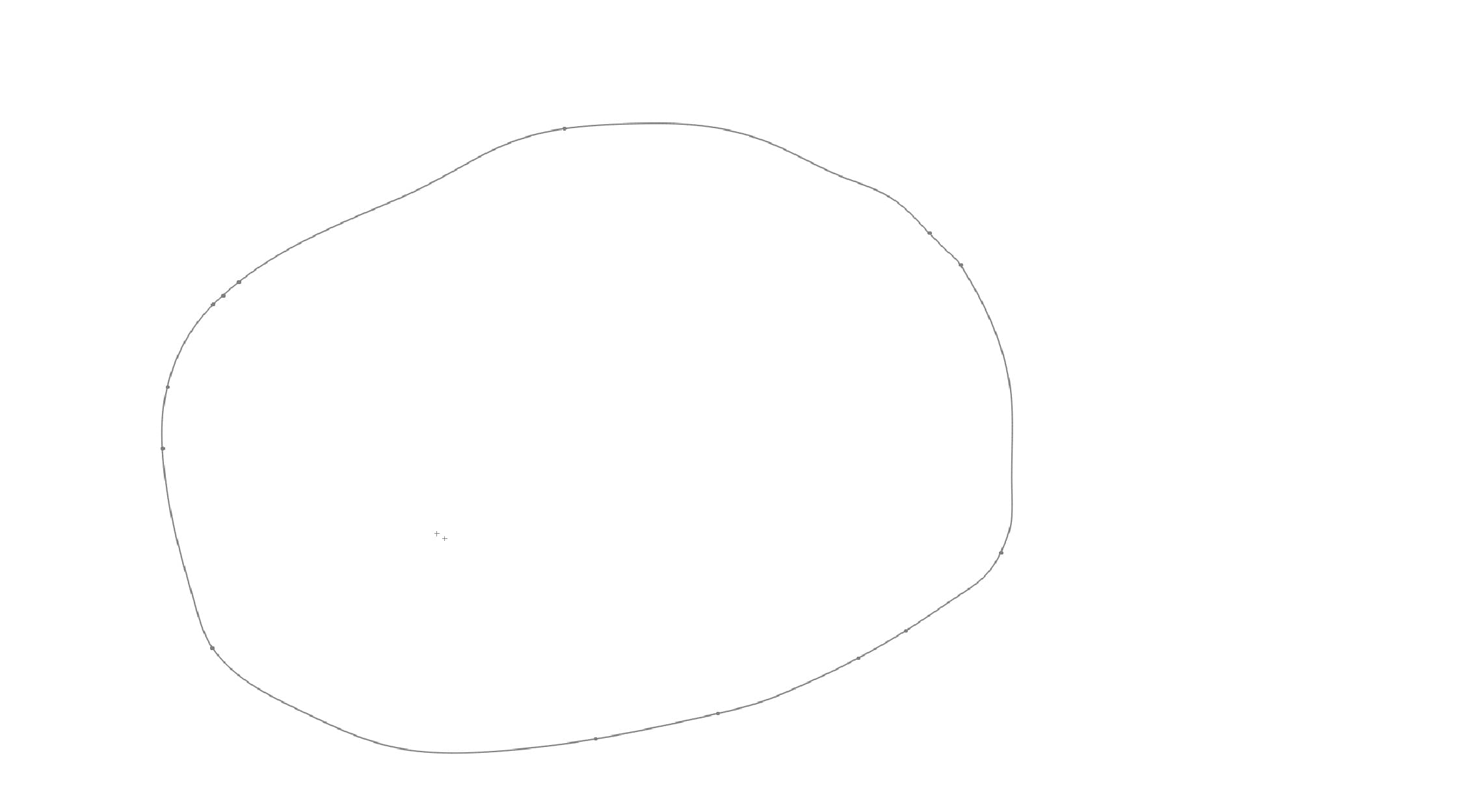

Supplement: Supplementary file 4 — Supporting Information [file ADVS-10-2203062-s013.zip › advs202203062-sup-0004-Supplementary-DataS3/Supplementary Data S3/32.jpg]

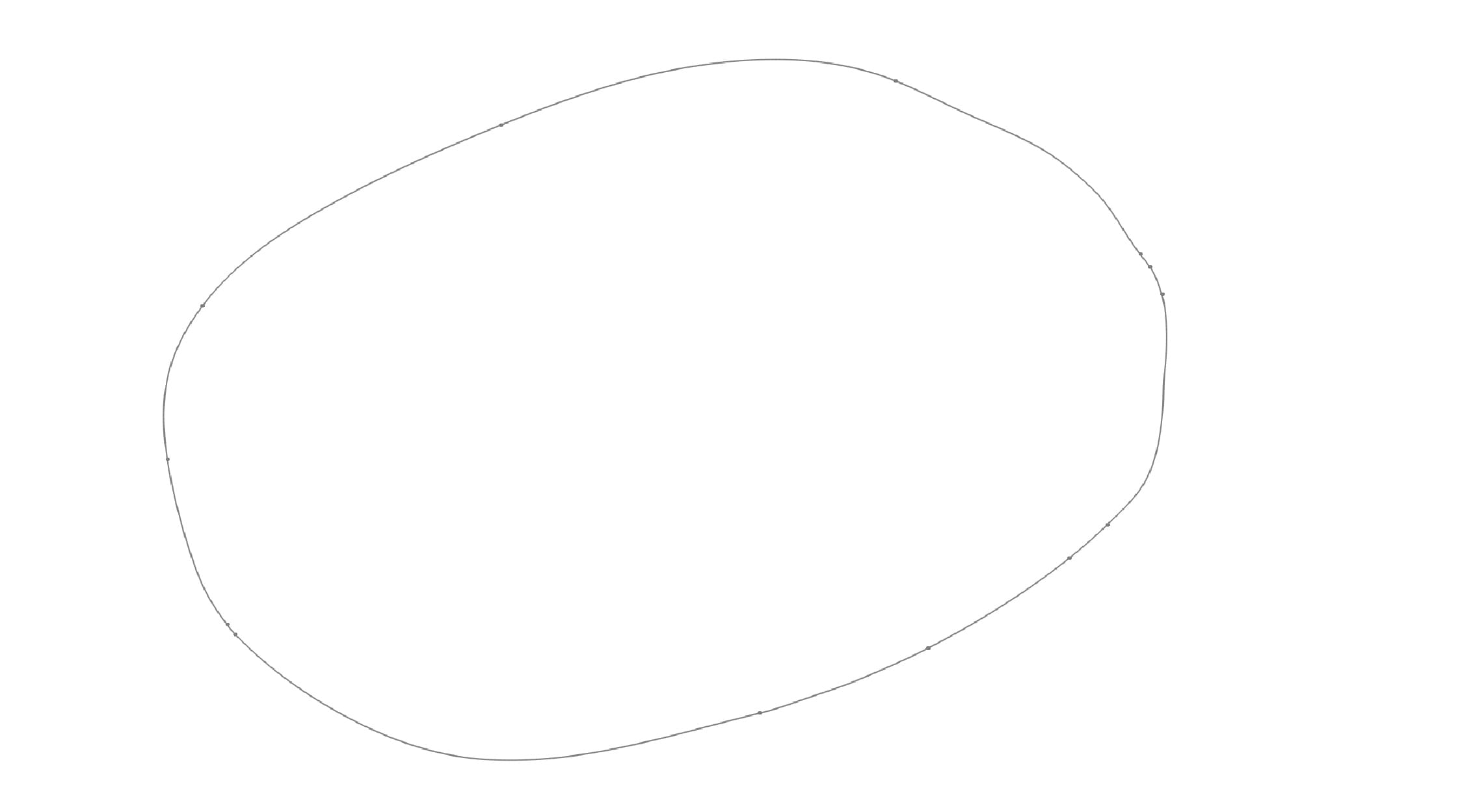

Supplement: Supplementary file 4 — Supporting Information [file ADVS-10-2203062-s013.zip › advs202203062-sup-0004-Supplementary-DataS3/Supplementary Data S3/33.jpg]

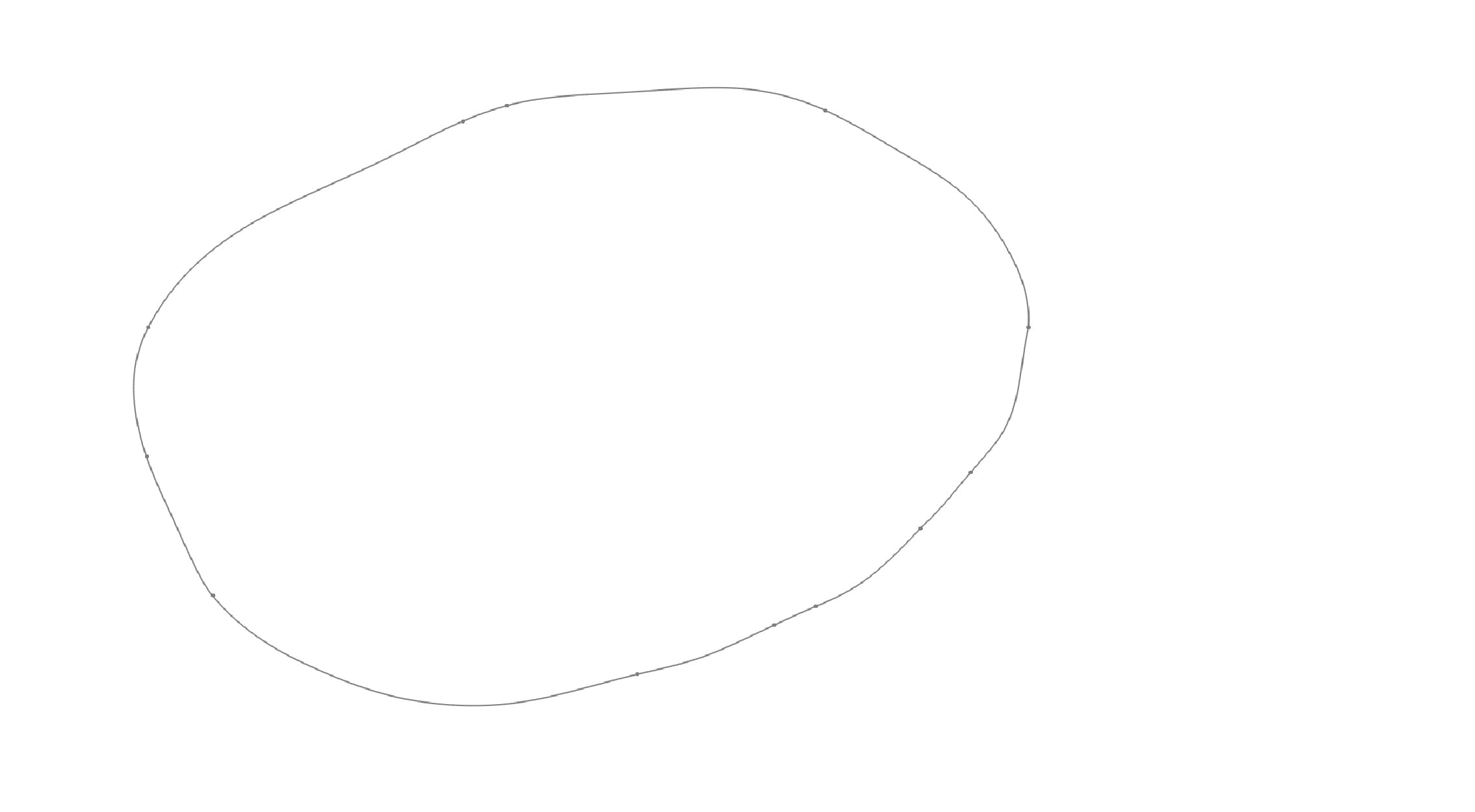

Supplement: Supplementary file 4 — Supporting Information [file ADVS-10-2203062-s013.zip › advs202203062-sup-0004-Supplementary-DataS3/Supplementary Data S3/34.jpg]

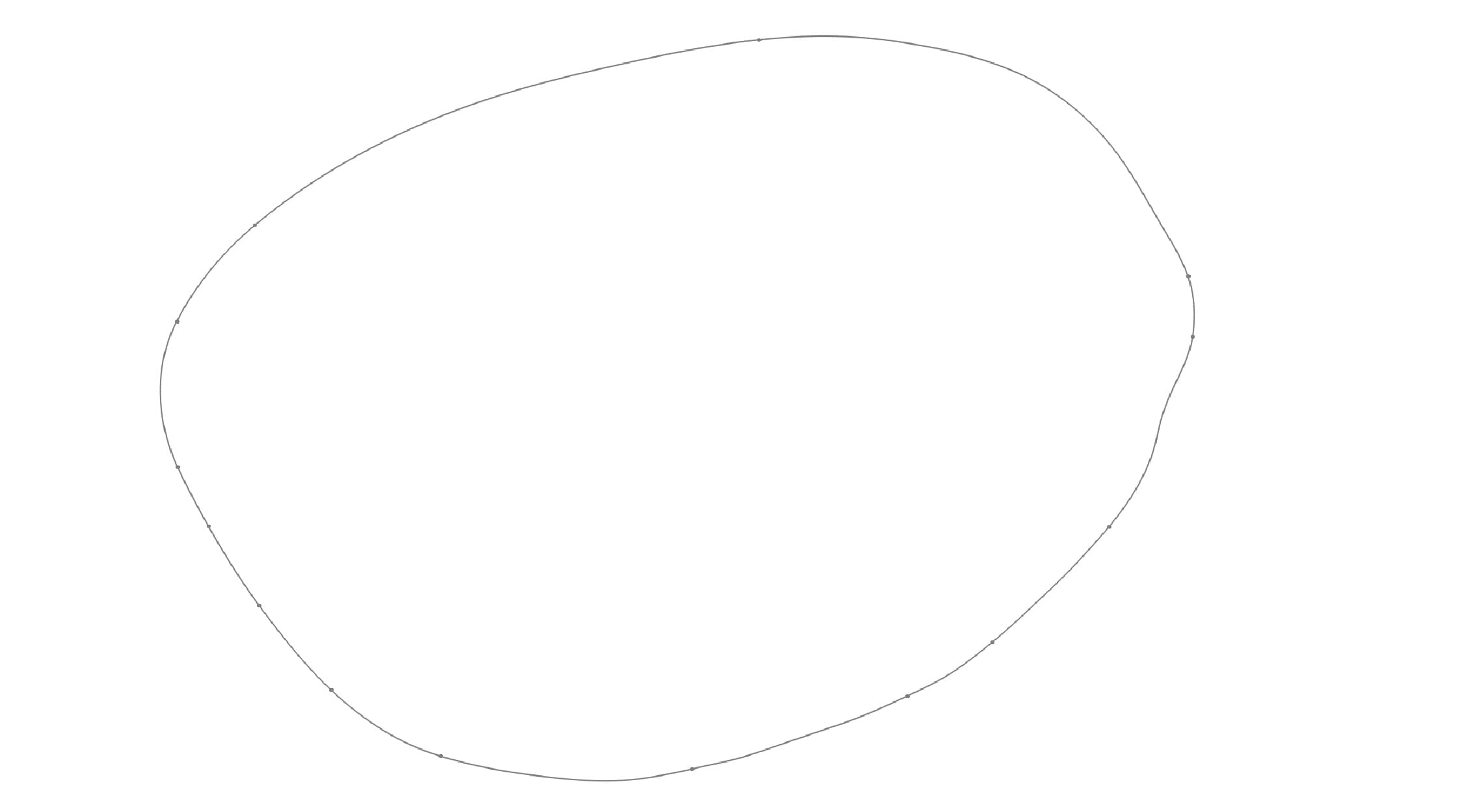

Supplement: Supplementary file 4 — Supporting Information [file ADVS-10-2203062-s013.zip › advs202203062-sup-0004-Supplementary-DataS3/Supplementary Data S3/35.jpg]

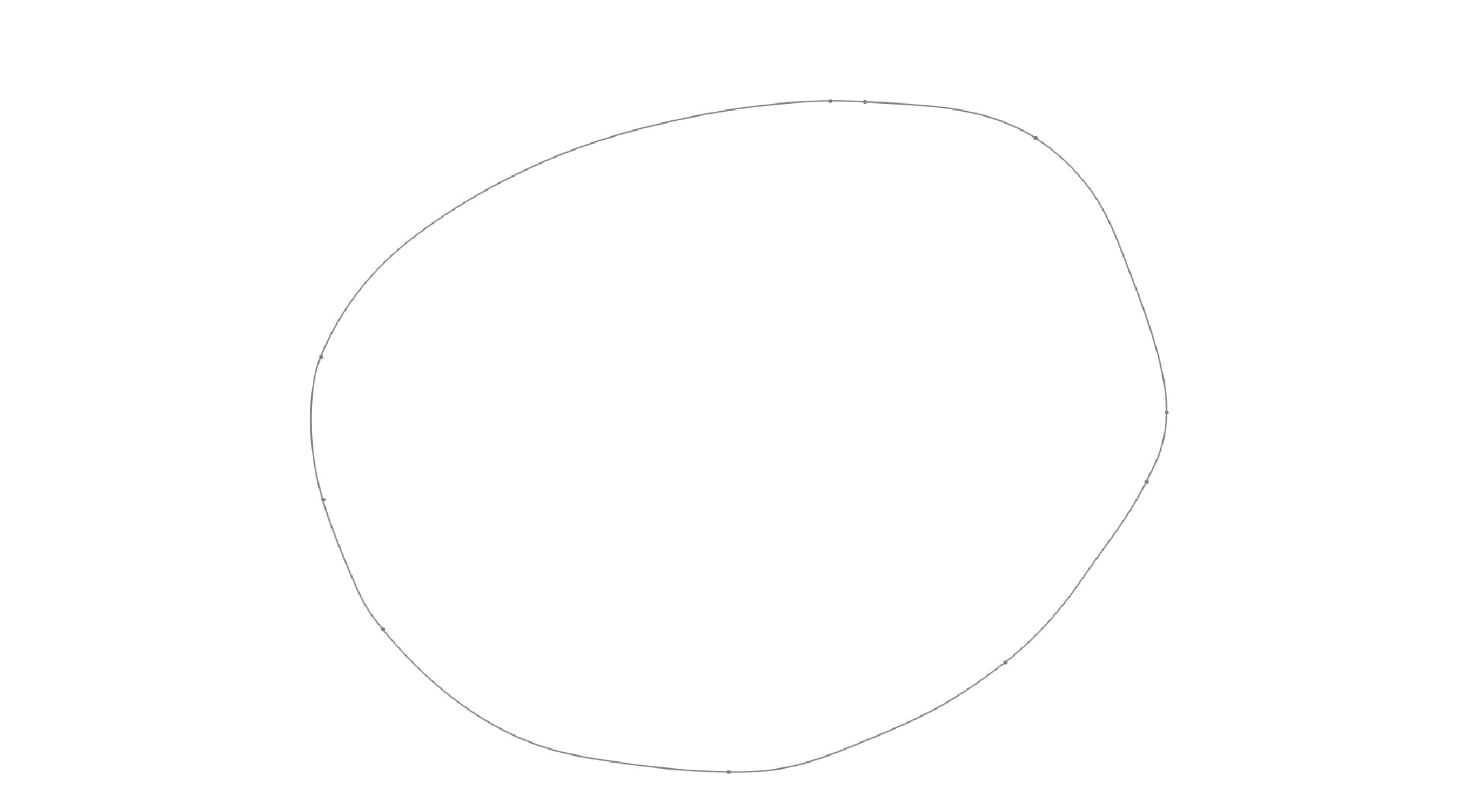

Supplement: Supplementary file 4 — Supporting Information [file ADVS-10-2203062-s013.zip › advs202203062-sup-0004-Supplementary-DataS3/Supplementary Data S3/36.jpg]

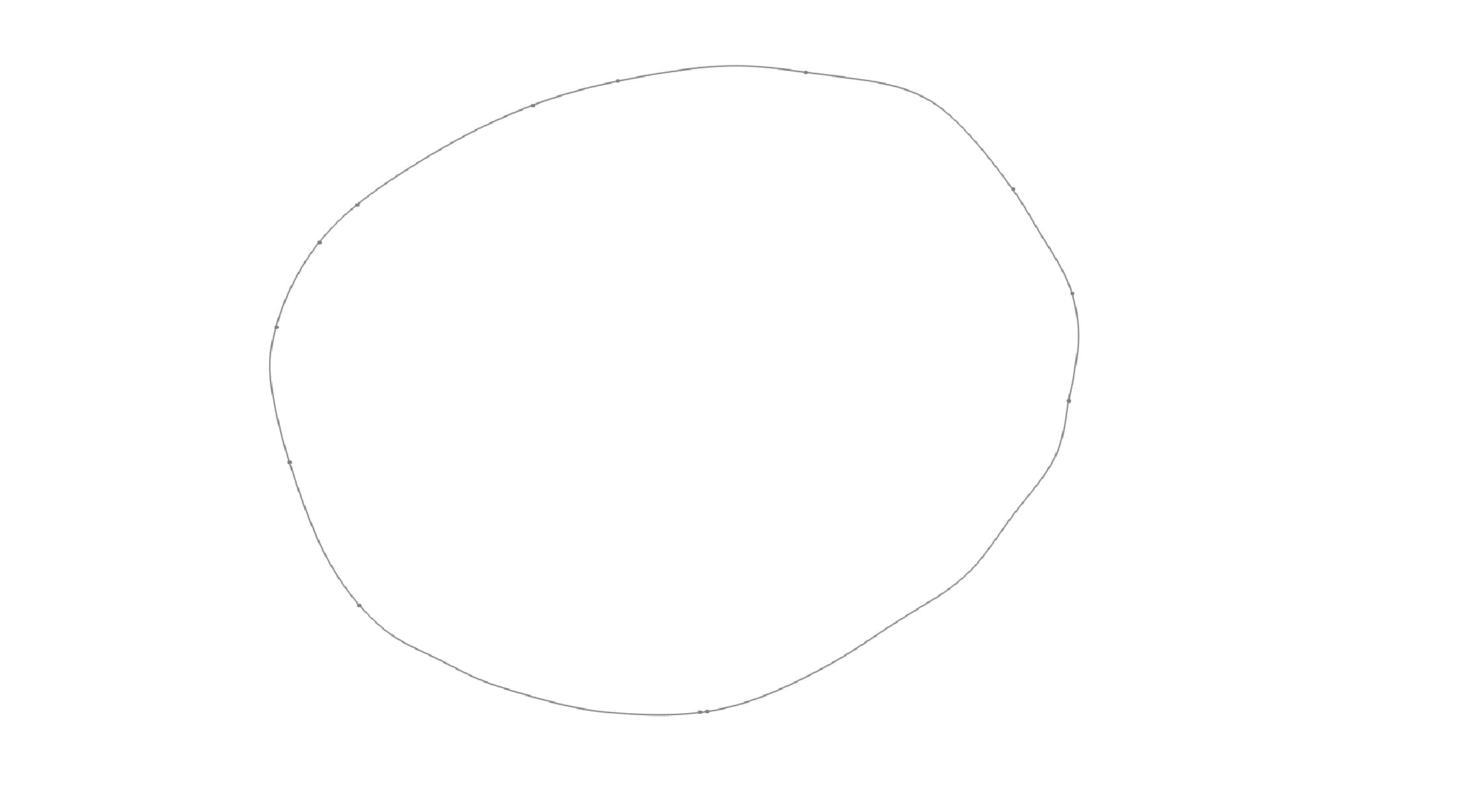

Supplement: Supplementary file 4 — Supporting Information [file ADVS-10-2203062-s013.zip › advs202203062-sup-0004-Supplementary-DataS3/Supplementary Data S3/37.jpg]

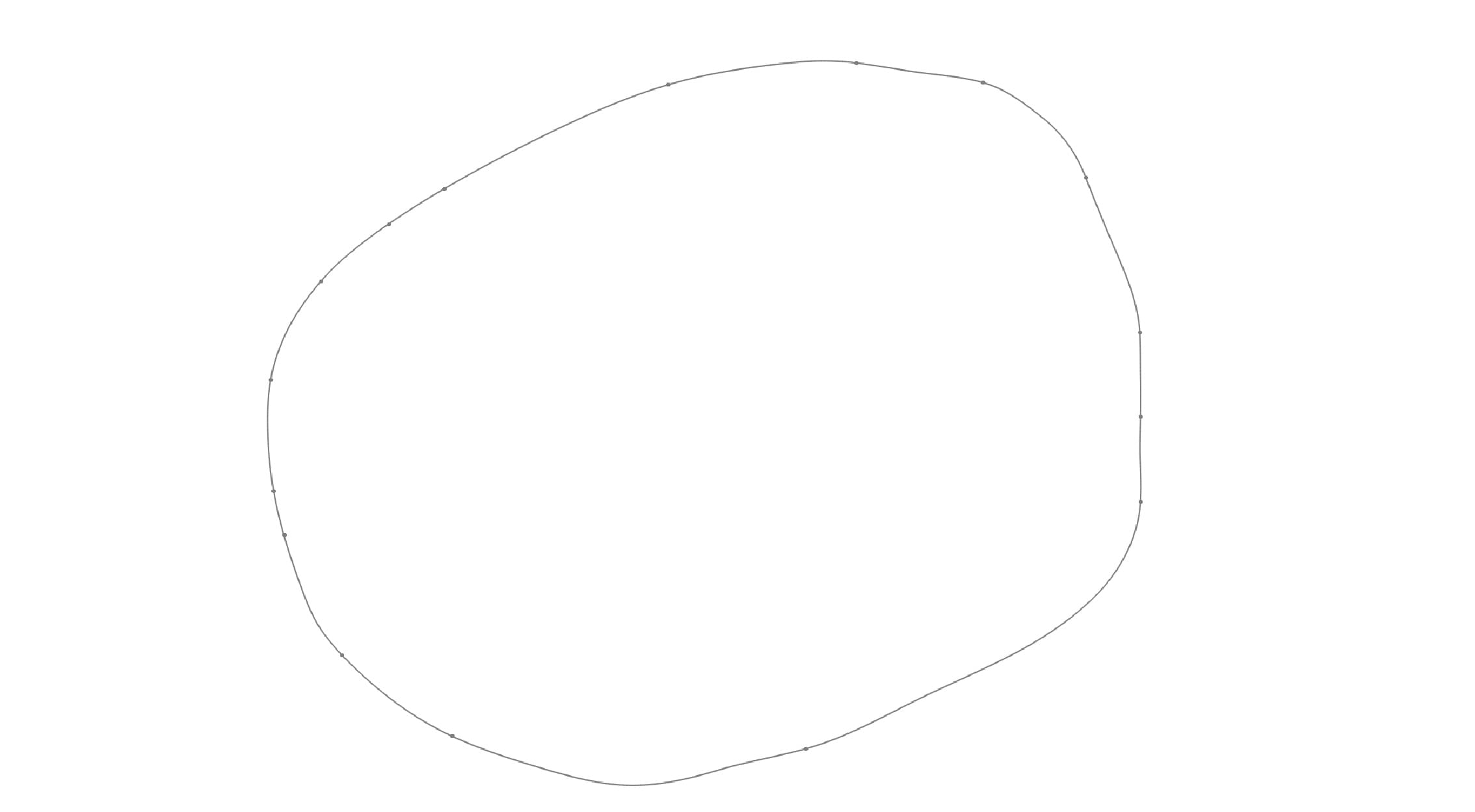

Supplement: Supplementary file 4 — Supporting Information [file ADVS-10-2203062-s013.zip › advs202203062-sup-0004-Supplementary-DataS3/Supplementary Data S3/38.jpg]

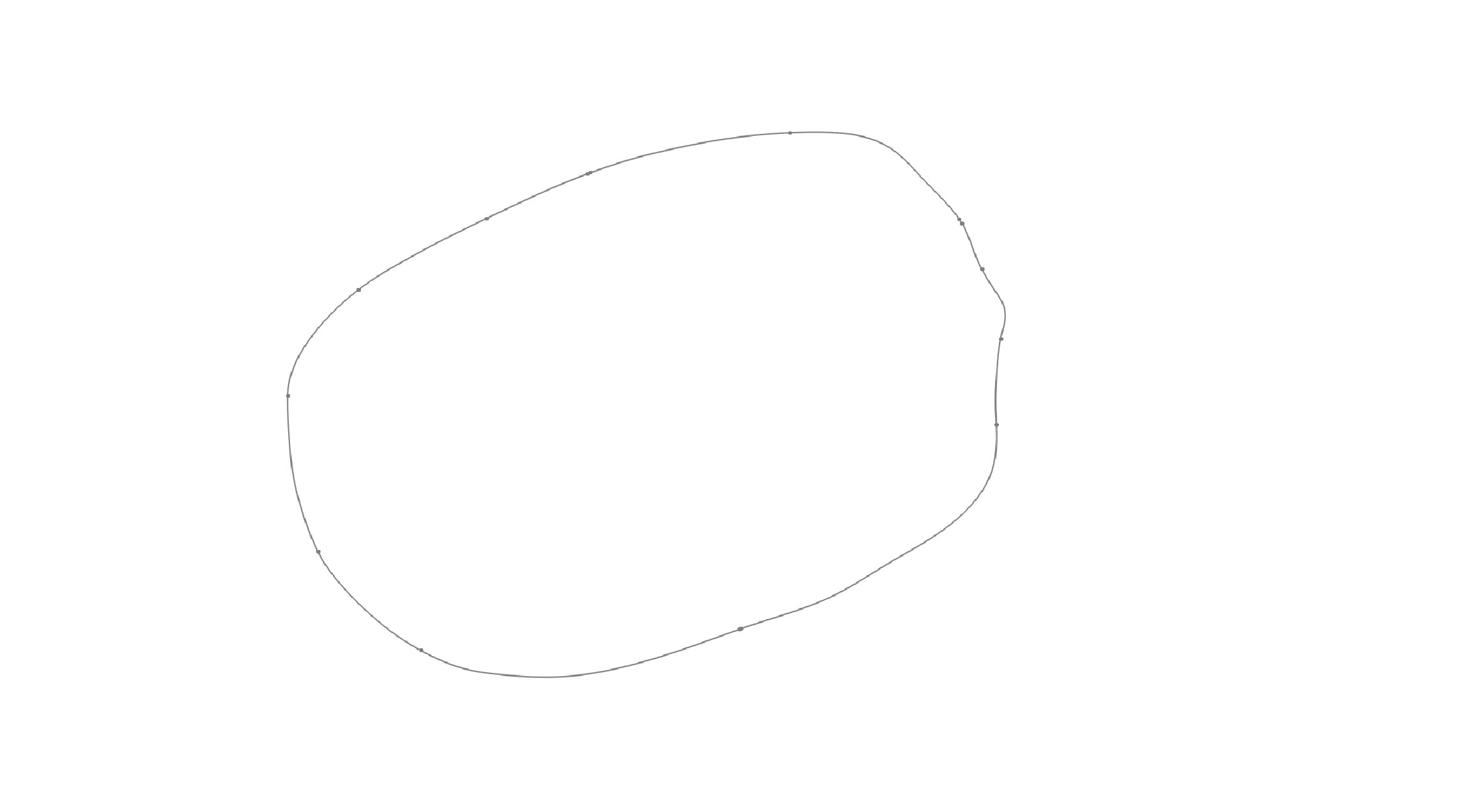

Supplement: Supplementary file 4 — Supporting Information [file ADVS-10-2203062-s013.zip › advs202203062-sup-0004-Supplementary-DataS3/Supplementary Data S3/39.jpg]

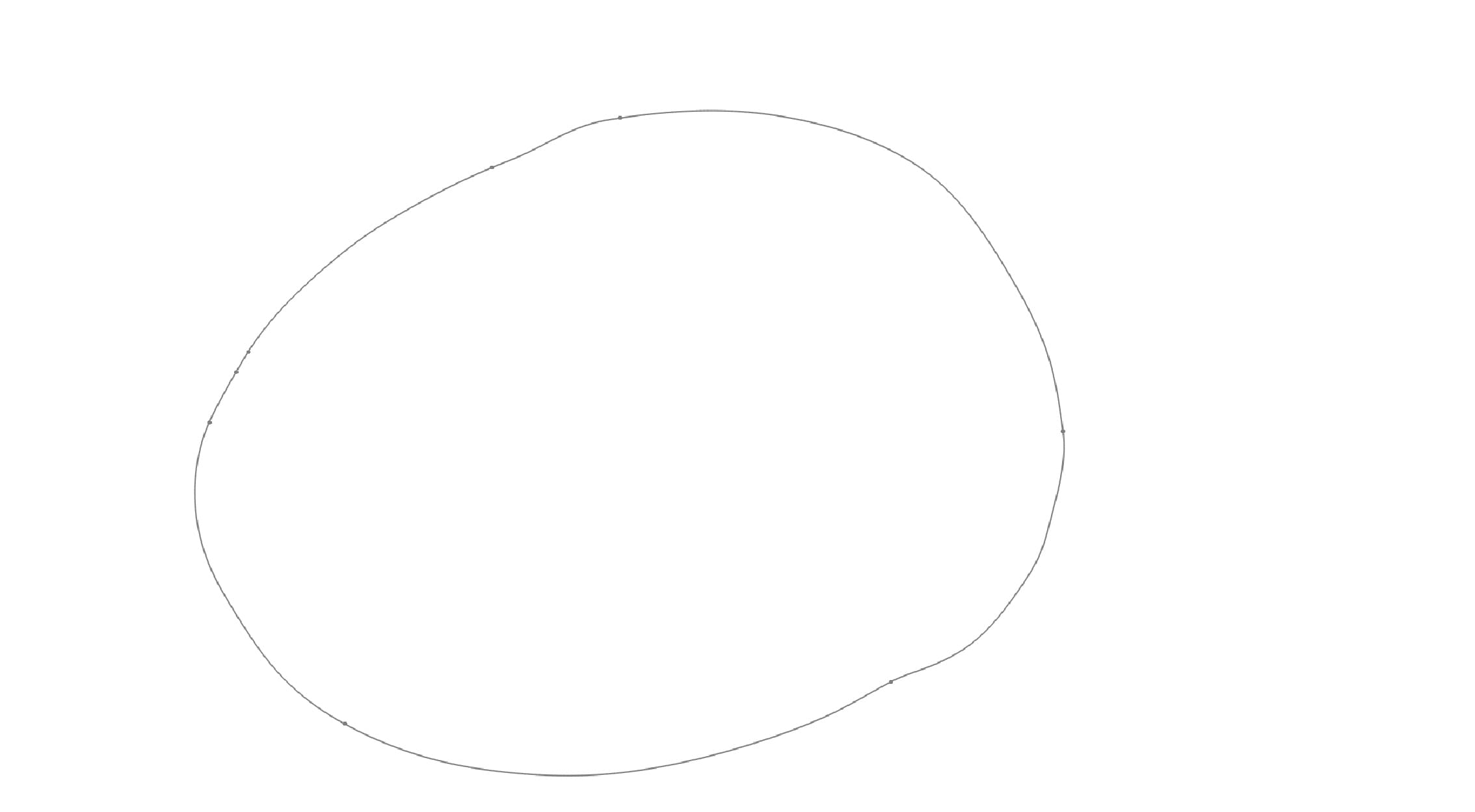

Supplement: Supplementary file 4 — Supporting Information [file ADVS-10-2203062-s013.zip › advs202203062-sup-0004-Supplementary-DataS3/Supplementary Data S3/4.jpg]

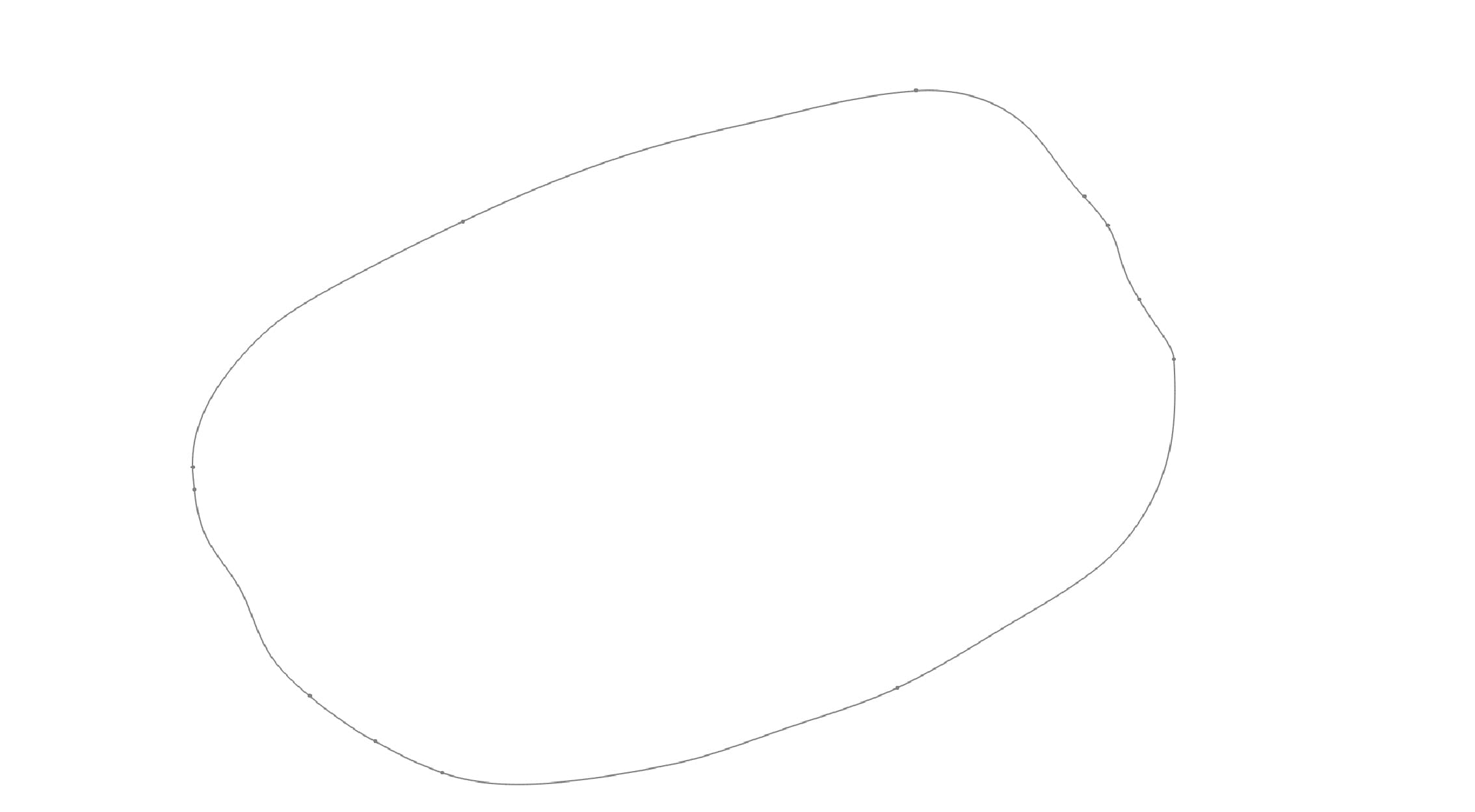

Supplement: Supplementary file 4 — Supporting Information [file ADVS-10-2203062-s013.zip › advs202203062-sup-0004-Supplementary-DataS3/Supplementary Data S3/40.jpg]

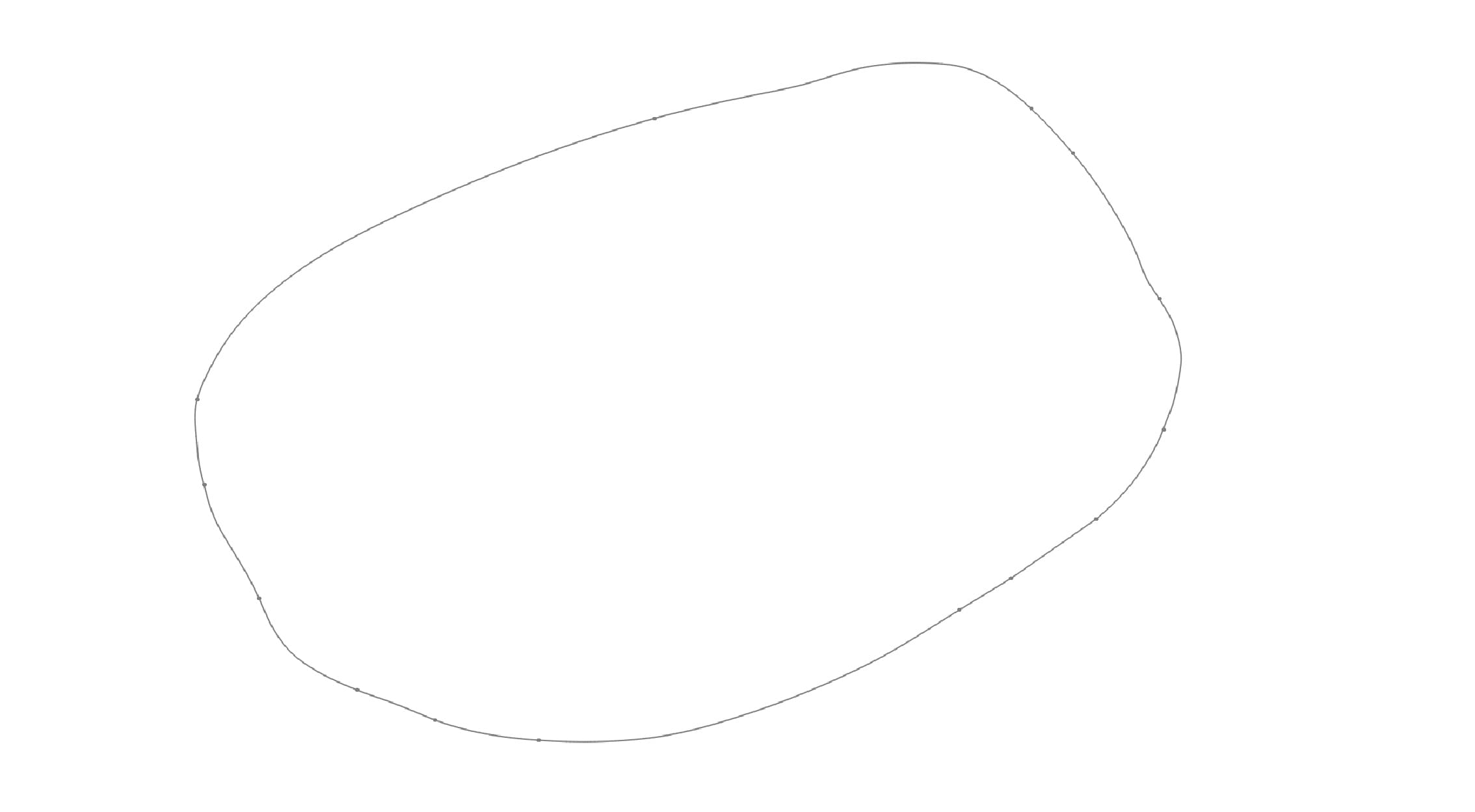

Supplement: Supplementary file 4 — Supporting Information [file ADVS-10-2203062-s013.zip › advs202203062-sup-0004-Supplementary-DataS3/Supplementary Data S3/41.jpg]

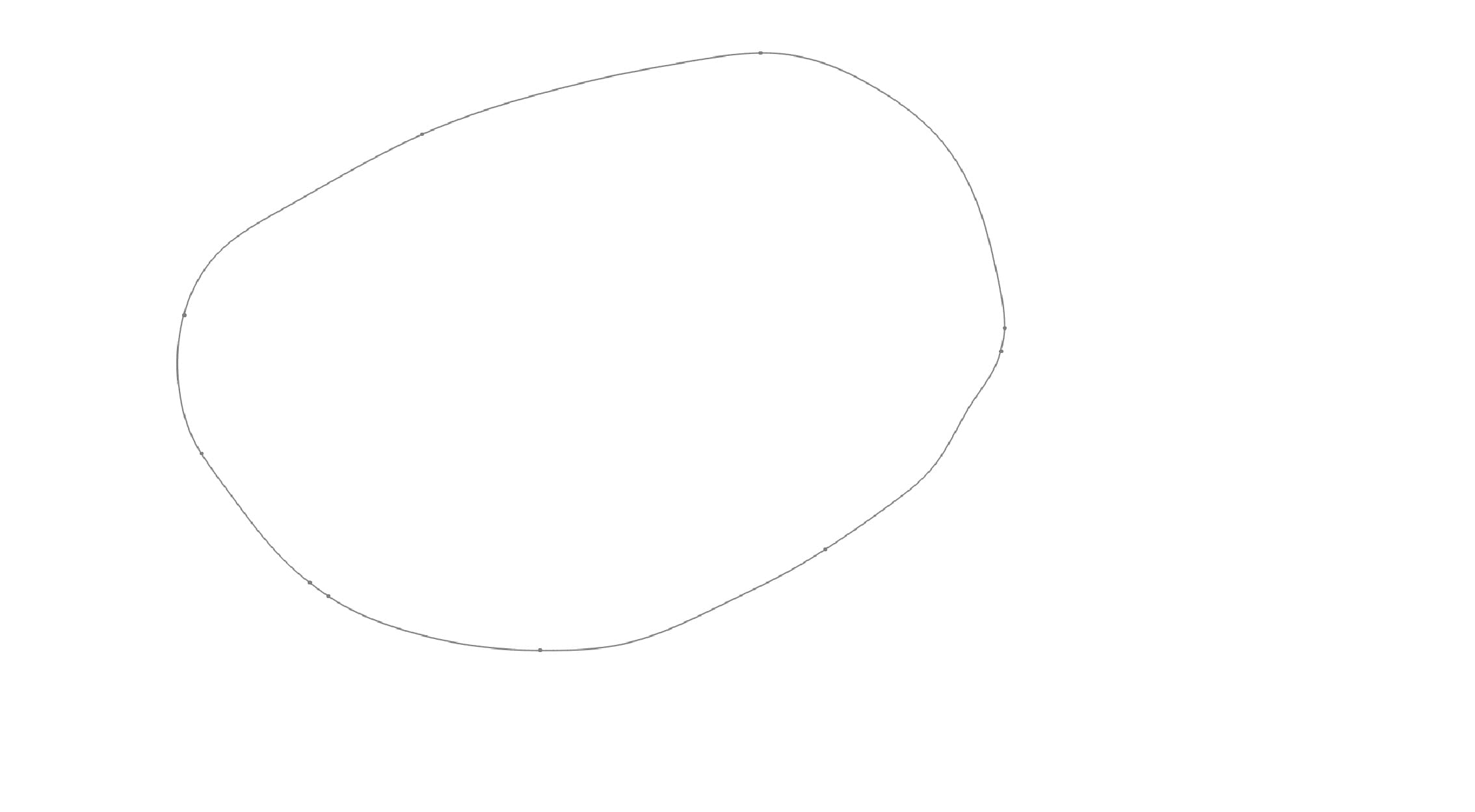

Supplement: Supplementary file 4 — Supporting Information [file ADVS-10-2203062-s013.zip › advs202203062-sup-0004-Supplementary-DataS3/Supplementary Data S3/42.jpg]

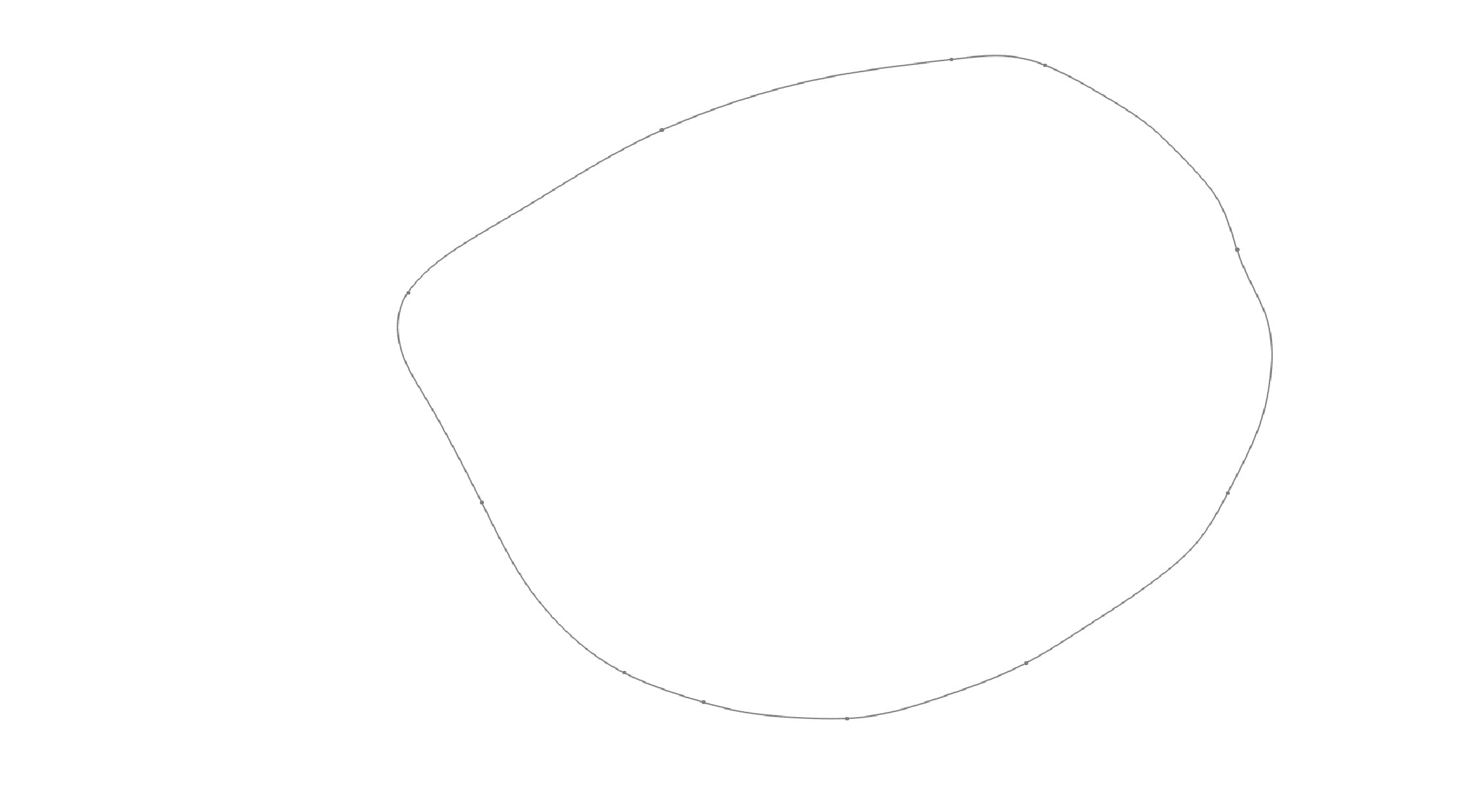

Supplement: Supplementary file 4 — Supporting Information [file ADVS-10-2203062-s013.zip › advs202203062-sup-0004-Supplementary-DataS3/Supplementary Data S3/43.jpg]

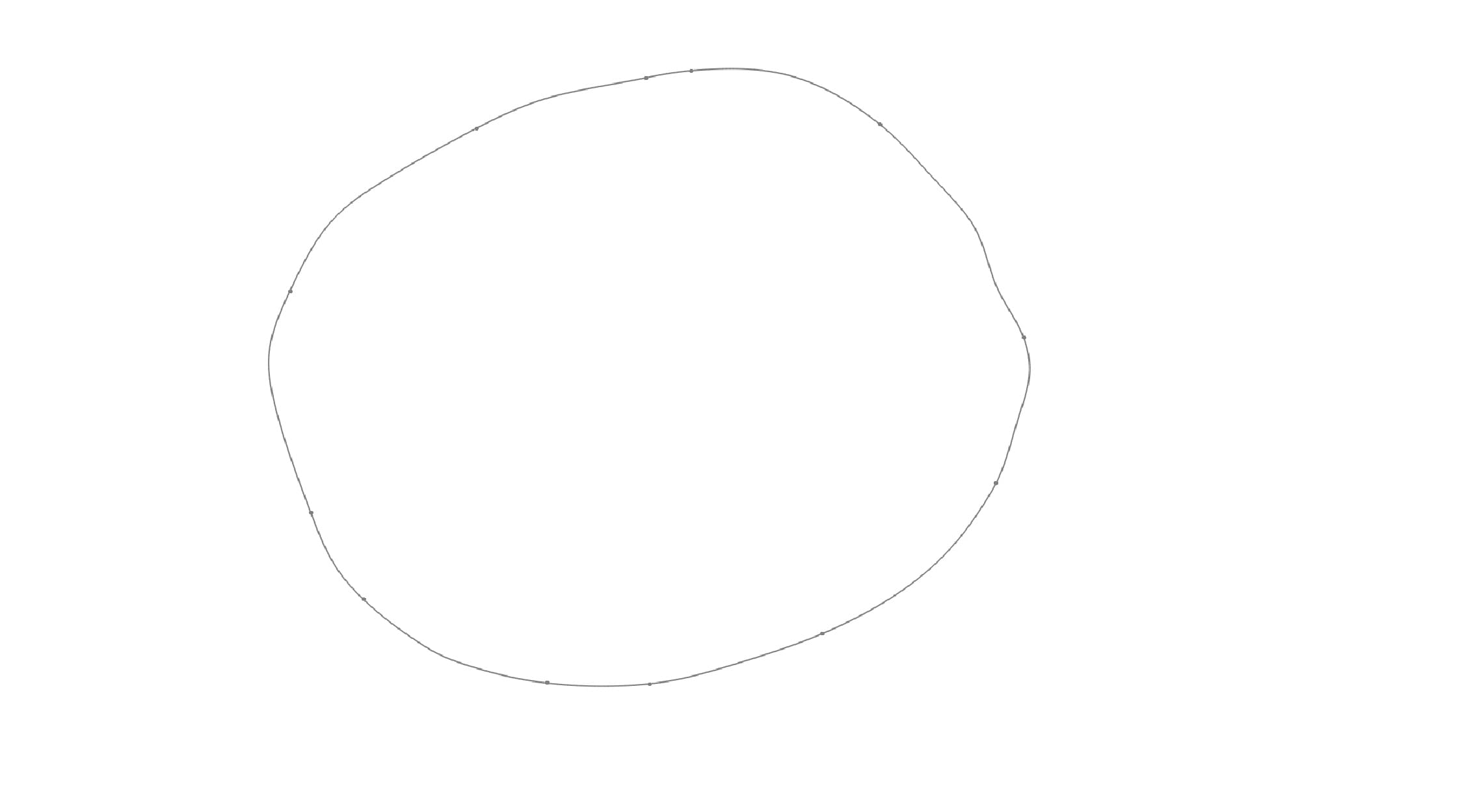

Supplement: Supplementary file 4 — Supporting Information [file ADVS-10-2203062-s013.zip › advs202203062-sup-0004-Supplementary-DataS3/Supplementary Data S3/44.jpg]

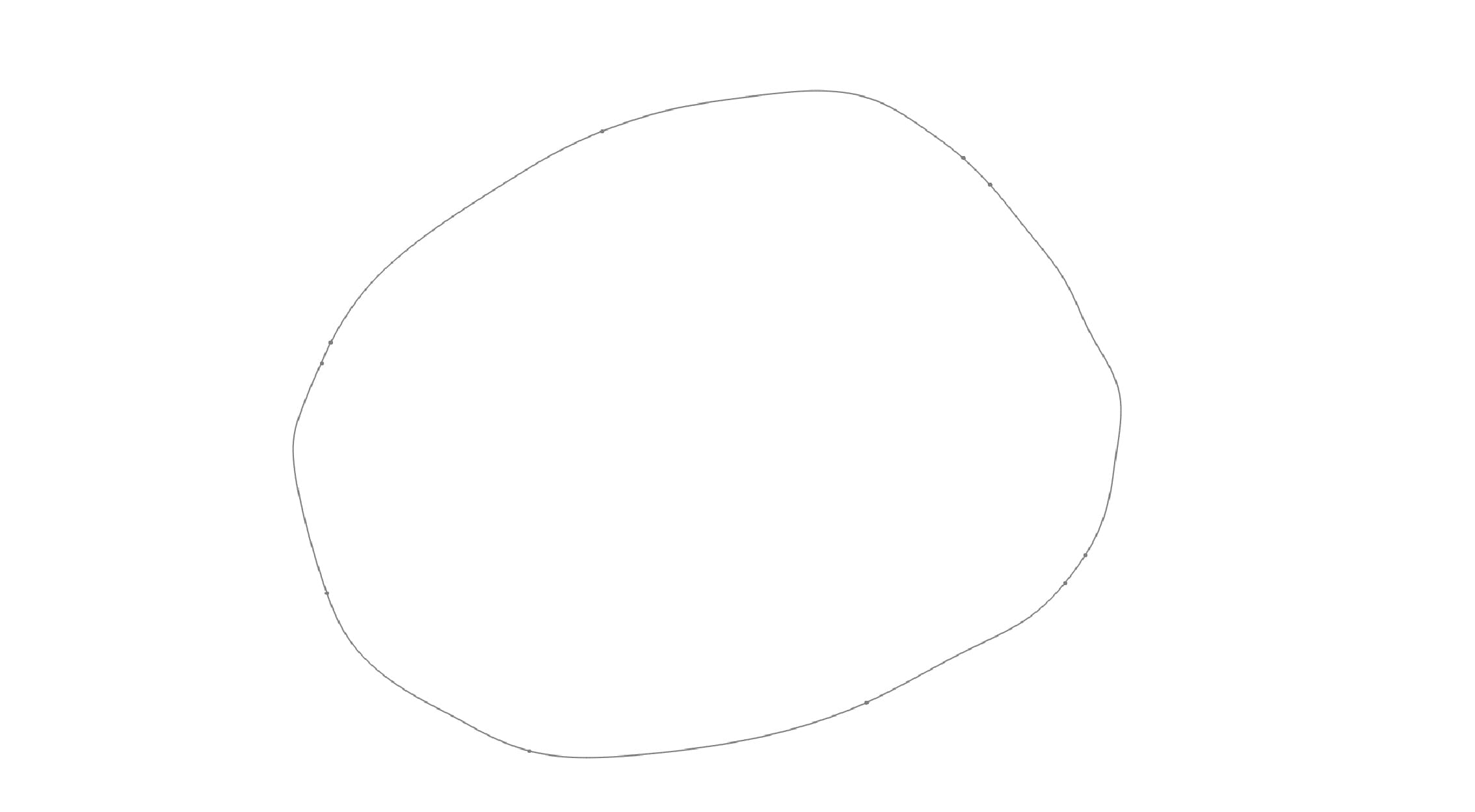

Supplement: Supplementary file 4 — Supporting Information [file ADVS-10-2203062-s013.zip › advs202203062-sup-0004-Supplementary-DataS3/Supplementary Data S3/45.jpg]

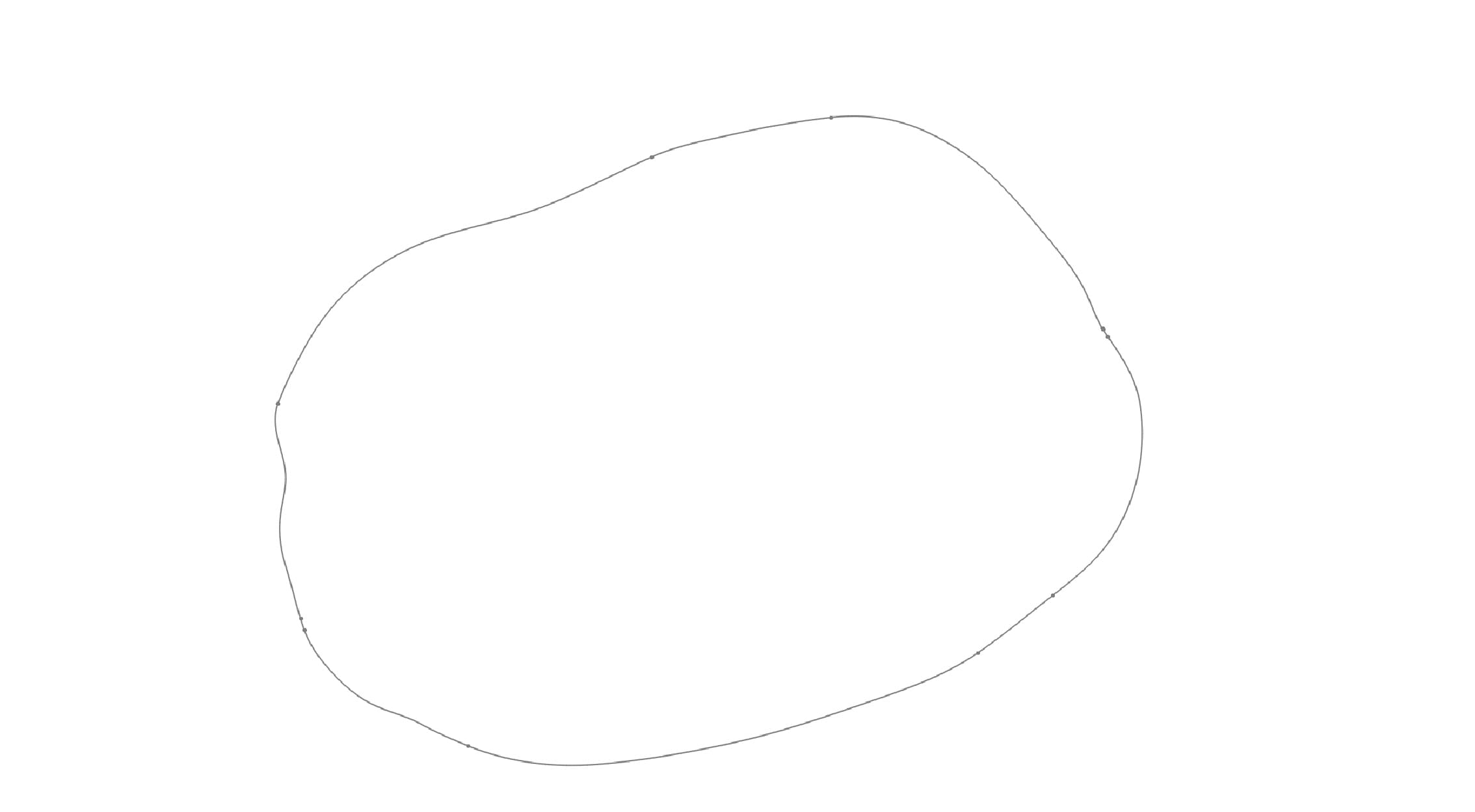

Supplement: Supplementary file 4 — Supporting Information [file ADVS-10-2203062-s013.zip › advs202203062-sup-0004-Supplementary-DataS3/Supplementary Data S3/46.jpg]

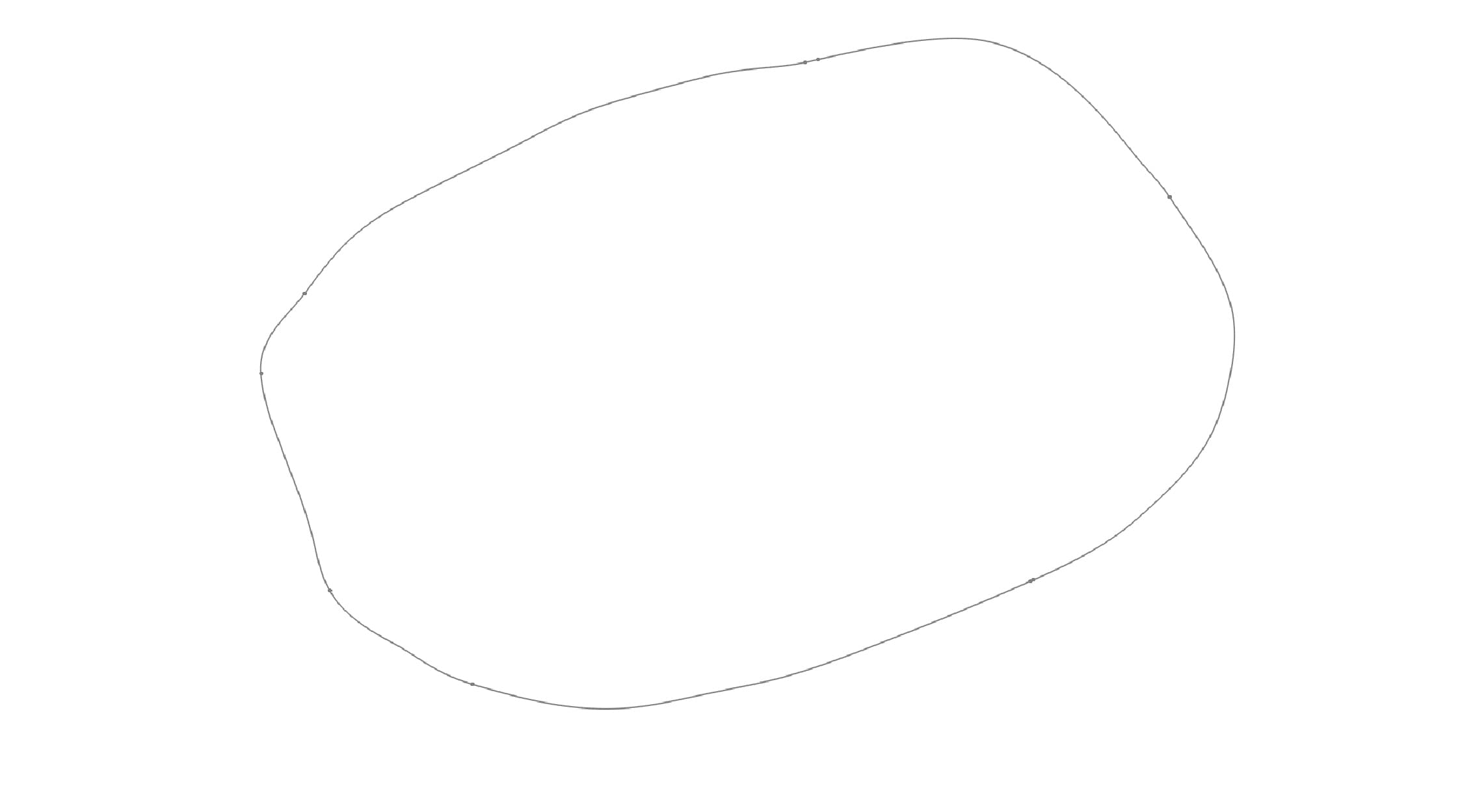

Supplement: Supplementary file 4 — Supporting Information [file ADVS-10-2203062-s013.zip › advs202203062-sup-0004-Supplementary-DataS3/Supplementary Data S3/47.jpg]

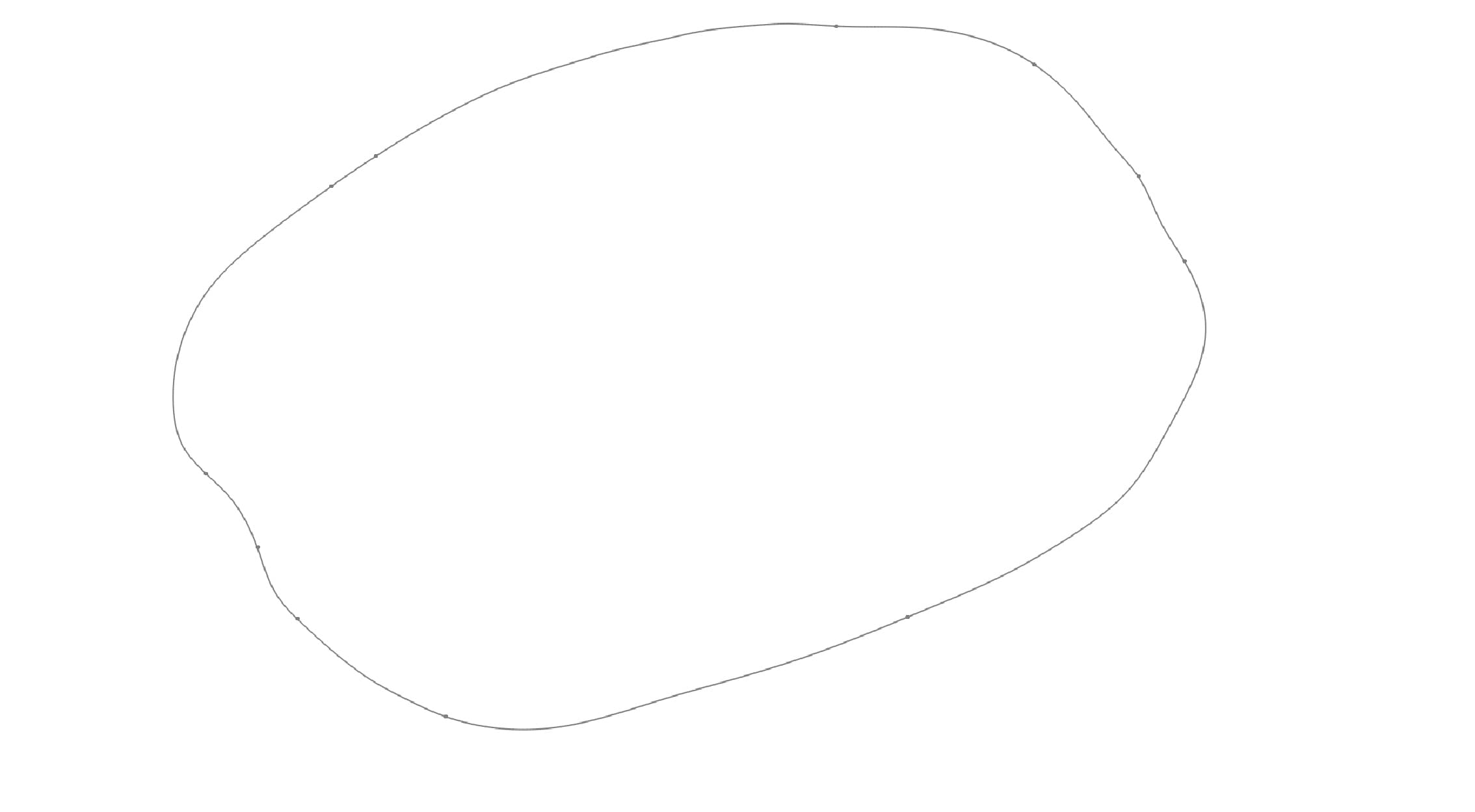

Supplement: Supplementary file 4 — Supporting Information [file ADVS-10-2203062-s013.zip › advs202203062-sup-0004-Supplementary-DataS3/Supplementary Data S3/48.jpg]

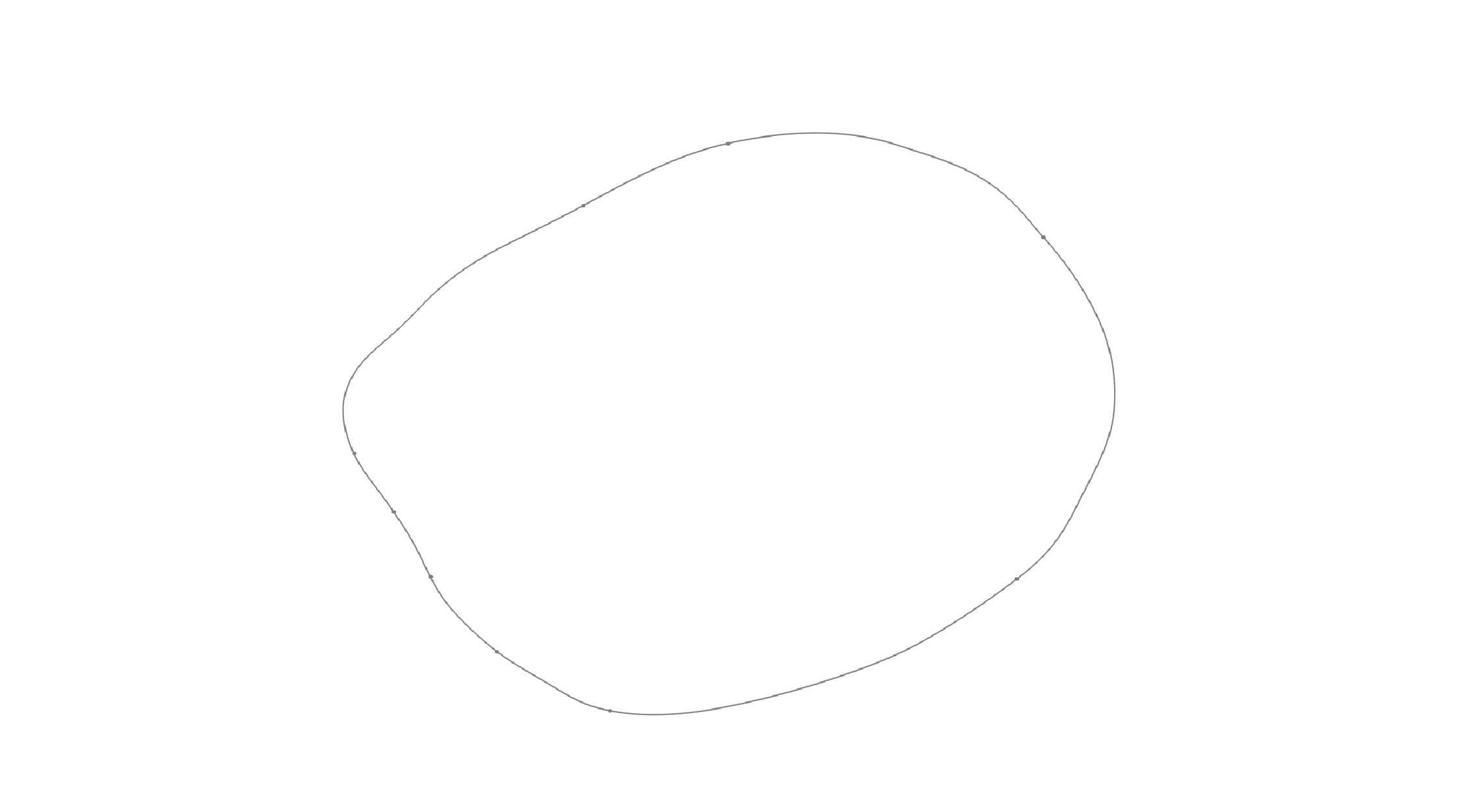

Supplement: Supplementary file 4 — Supporting Information [file ADVS-10-2203062-s013.zip › advs202203062-sup-0004-Supplementary-DataS3/Supplementary Data S3/49.jpg]

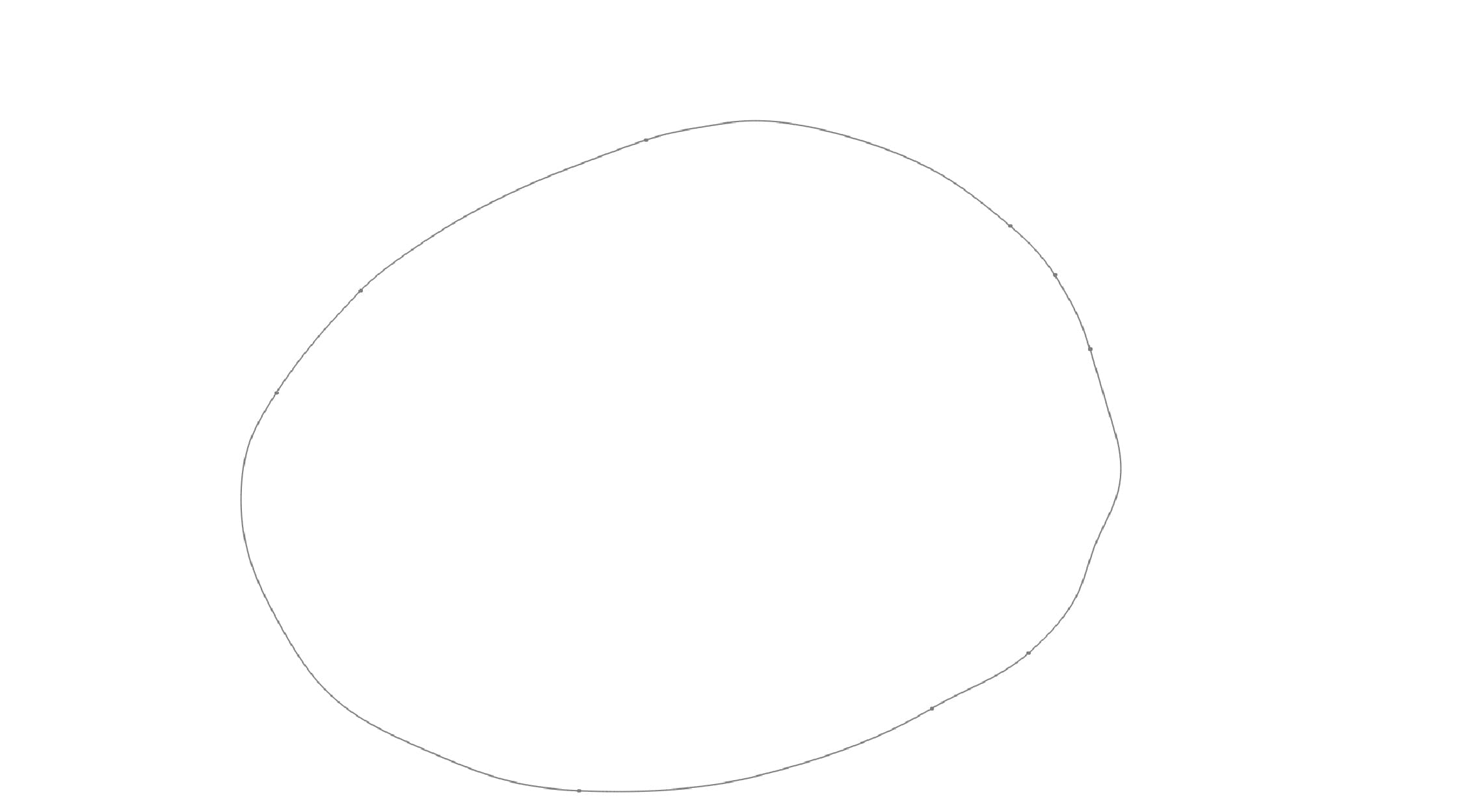

Supplement: Supplementary file 4 — Supporting Information [file ADVS-10-2203062-s013.zip › advs202203062-sup-0004-Supplementary-DataS3/Supplementary Data S3/5.jpg]

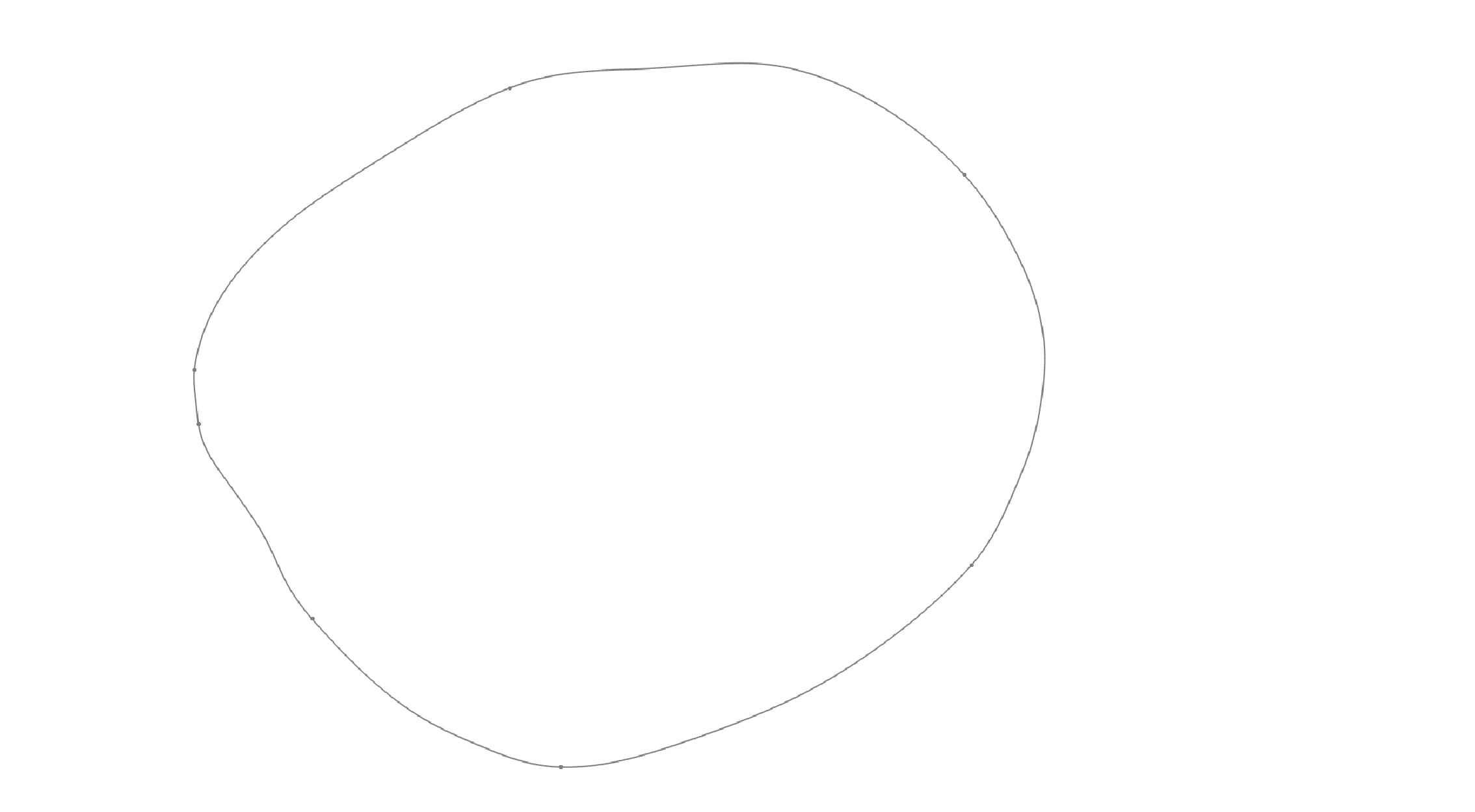

Supplement: Supplementary file 4 — Supporting Information [file ADVS-10-2203062-s013.zip › advs202203062-sup-0004-Supplementary-DataS3/Supplementary Data S3/50.jpg]

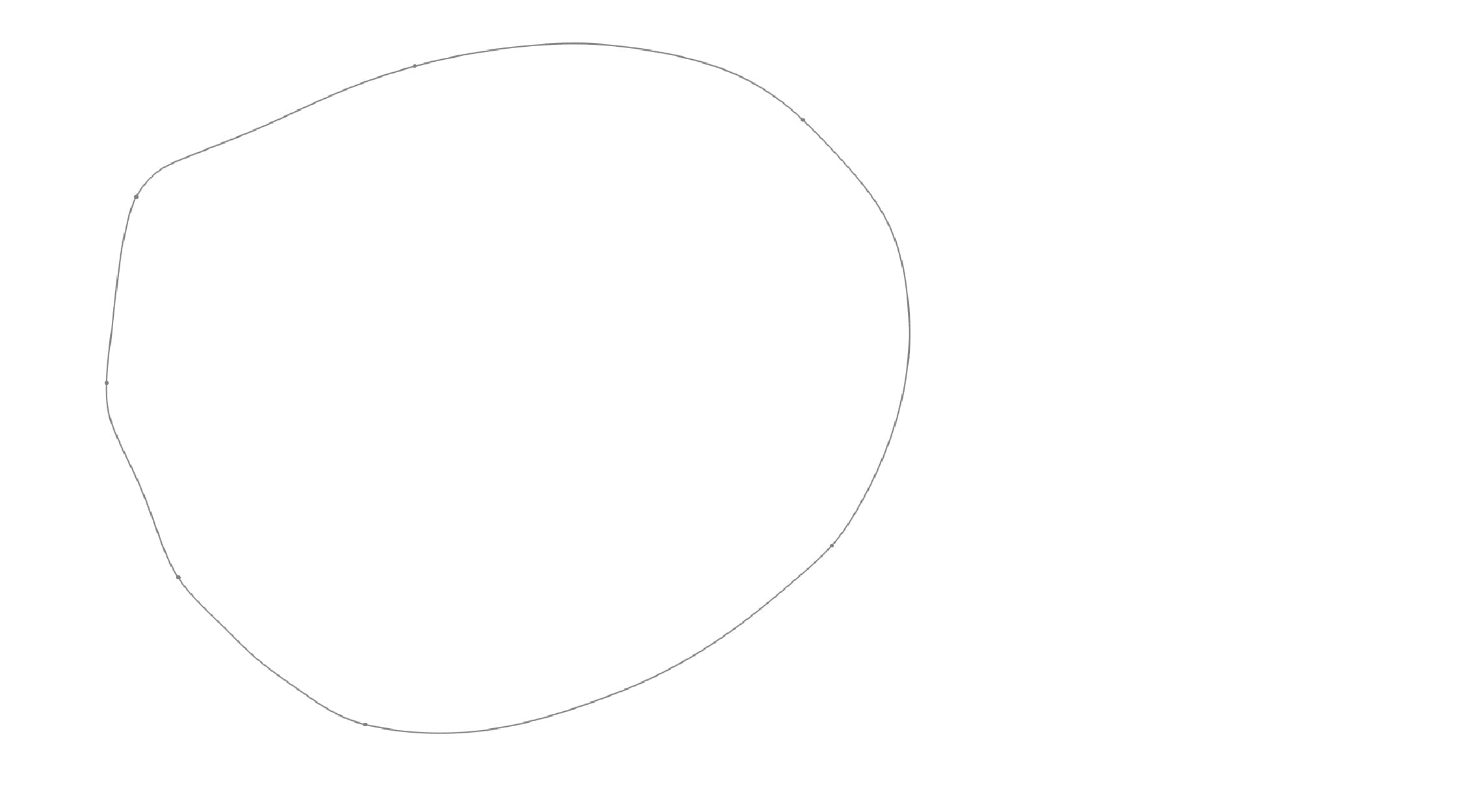

Supplement: Supplementary file 4 — Supporting Information [file ADVS-10-2203062-s013.zip › advs202203062-sup-0004-Supplementary-DataS3/Supplementary Data S3/51.jpg]

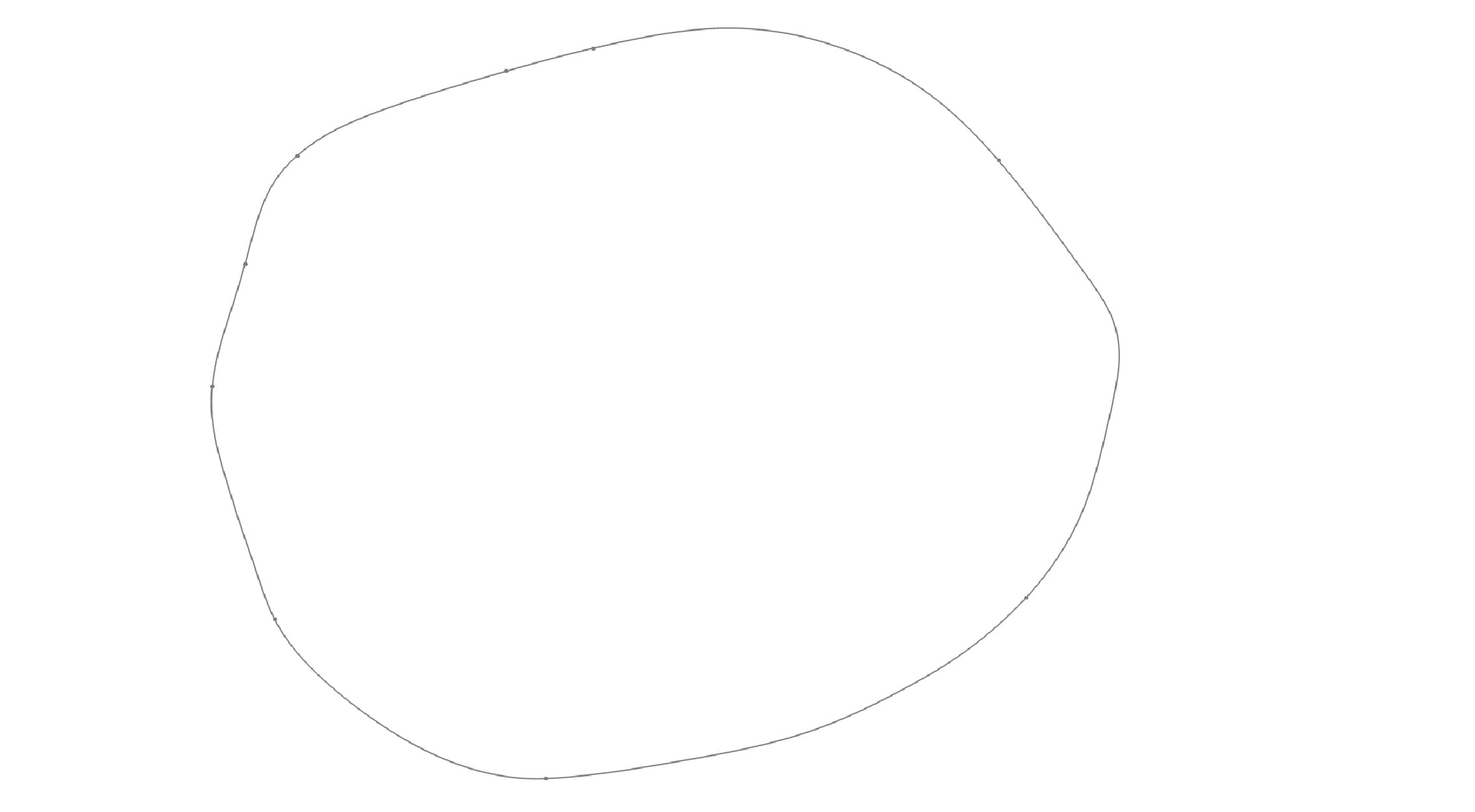

Supplement: Supplementary file 4 — Supporting Information [file ADVS-10-2203062-s013.zip › advs202203062-sup-0004-Supplementary-DataS3/Supplementary Data S3/52.jpg]

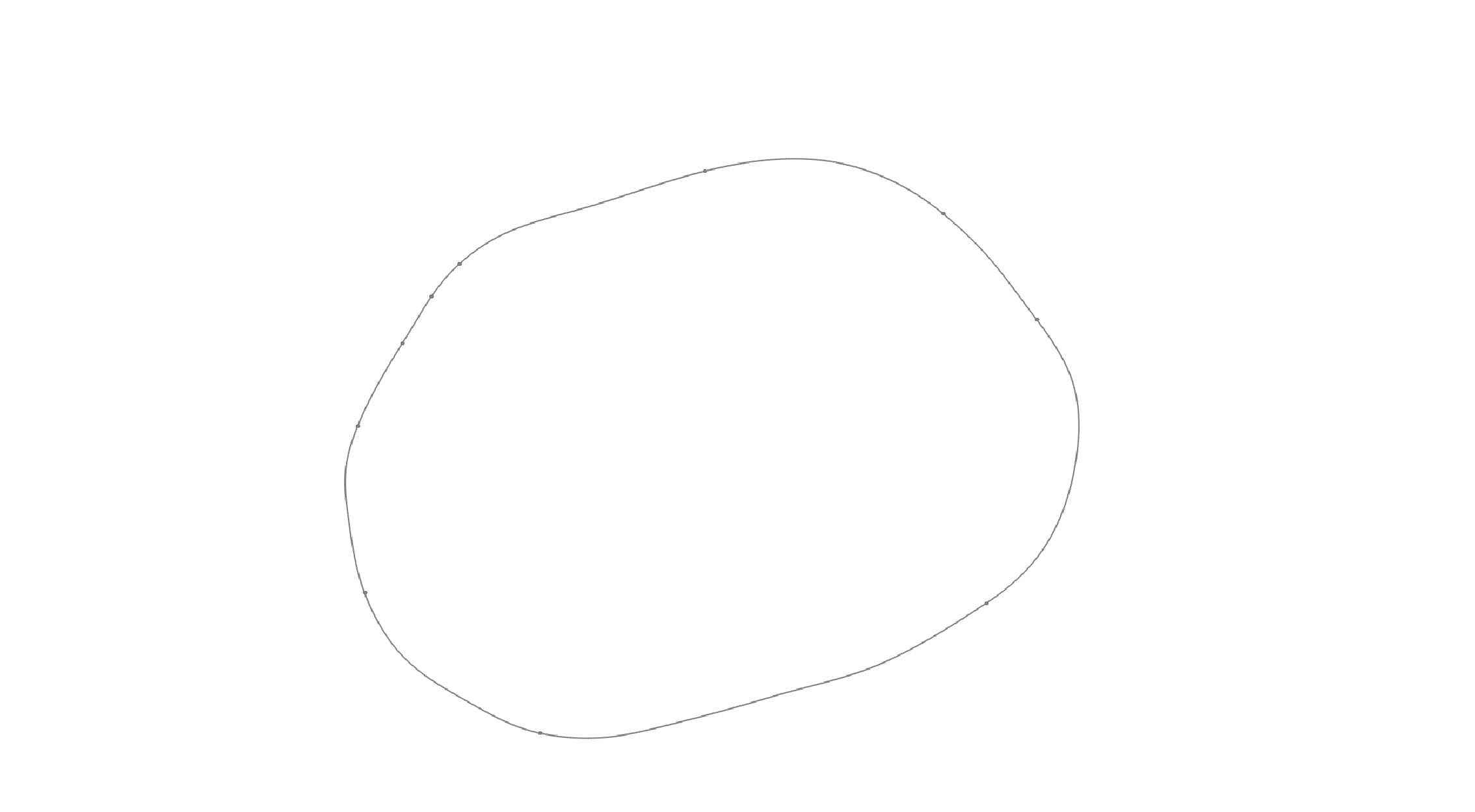

Supplement: Supplementary file 4 — Supporting Information [file ADVS-10-2203062-s013.zip › advs202203062-sup-0004-Supplementary-DataS3/Supplementary Data S3/53.jpg]

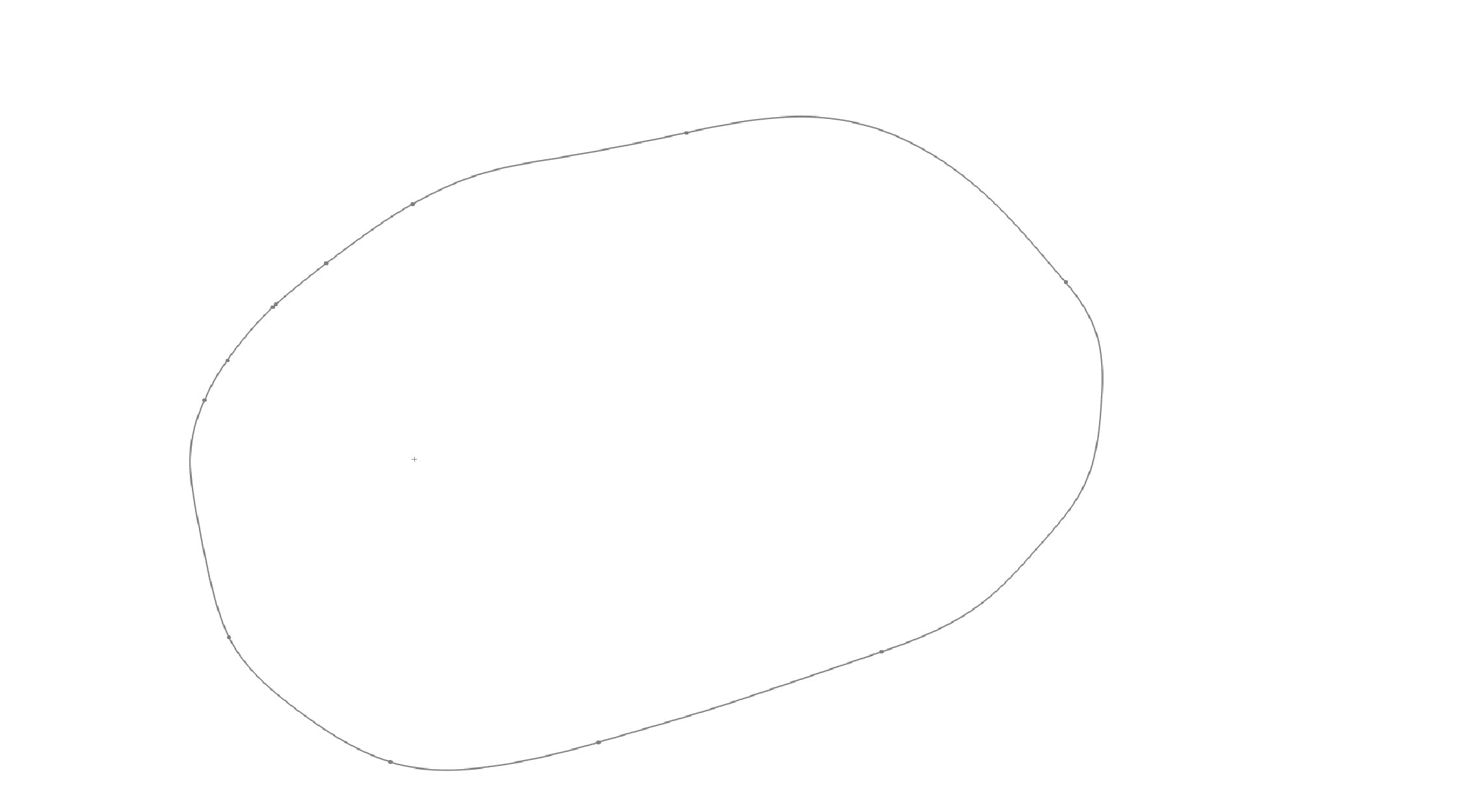

Supplement: Supplementary file 4 — Supporting Information [file ADVS-10-2203062-s013.zip › advs202203062-sup-0004-Supplementary-DataS3/Supplementary Data S3/54.jpg]

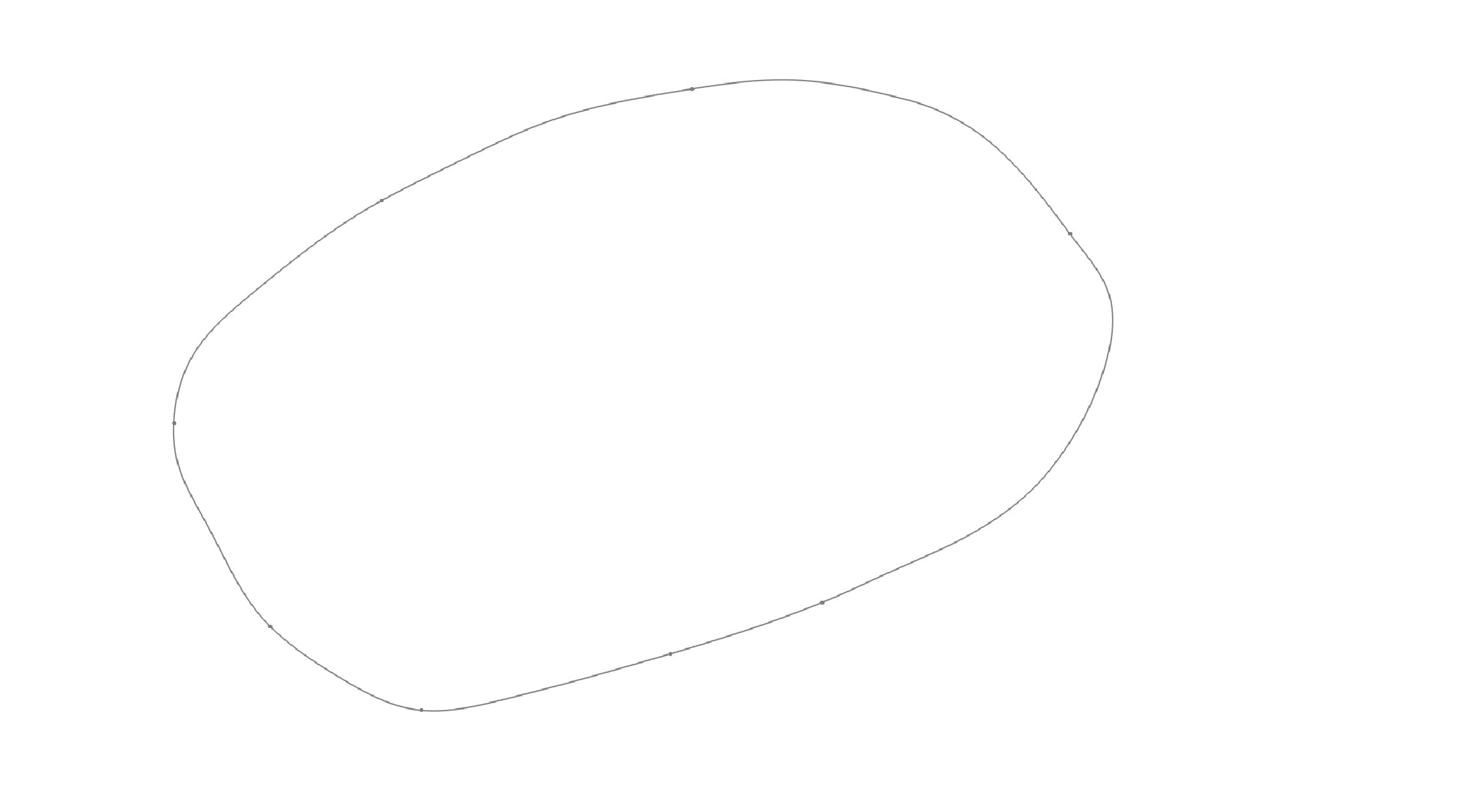

Supplement: Supplementary file 4 — Supporting Information [file ADVS-10-2203062-s013.zip › advs202203062-sup-0004-Supplementary-DataS3/Supplementary Data S3/55.jpg]

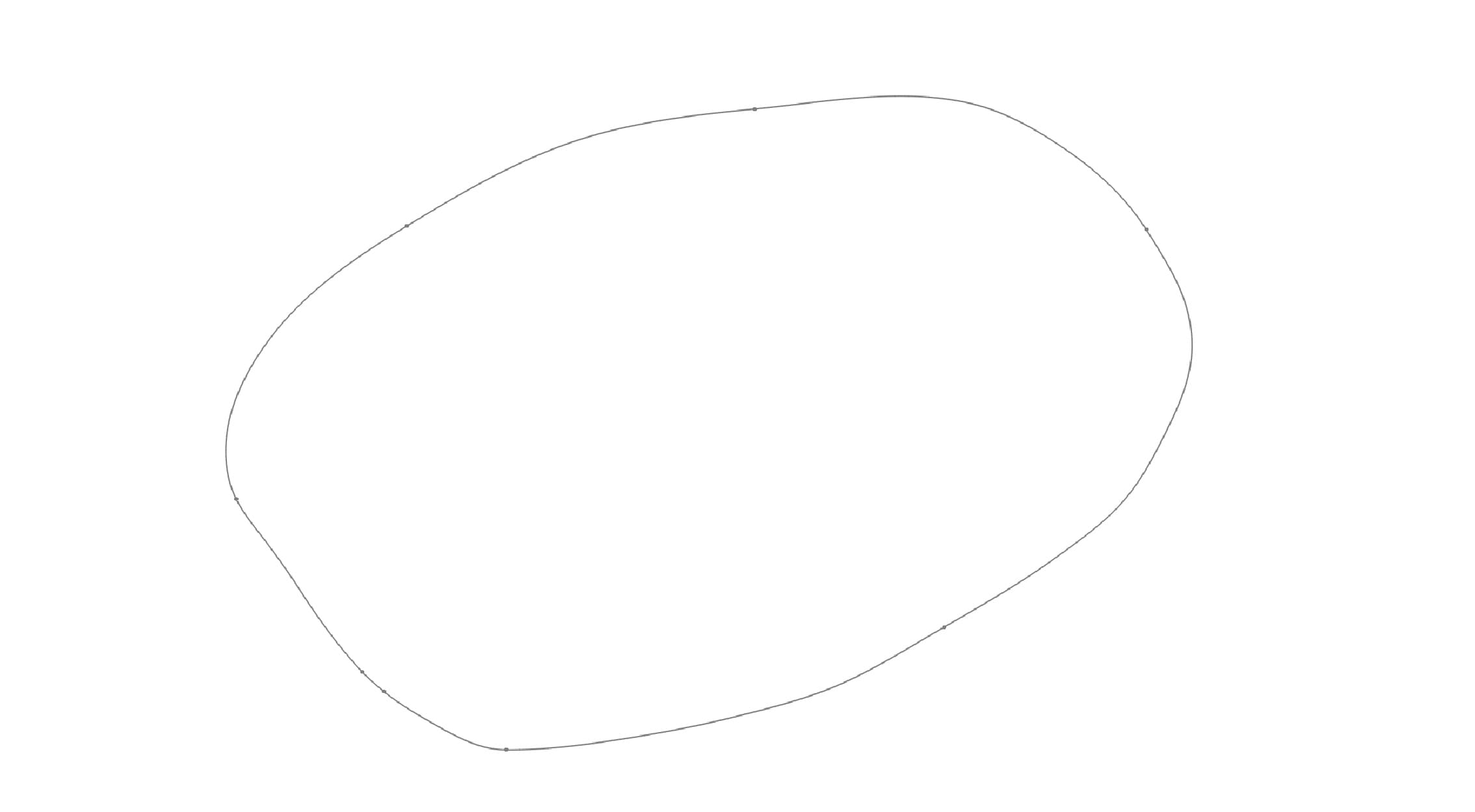

Supplement: Supplementary file 4 — Supporting Information [file ADVS-10-2203062-s013.zip › advs202203062-sup-0004-Supplementary-DataS3/Supplementary Data S3/56.jpg]

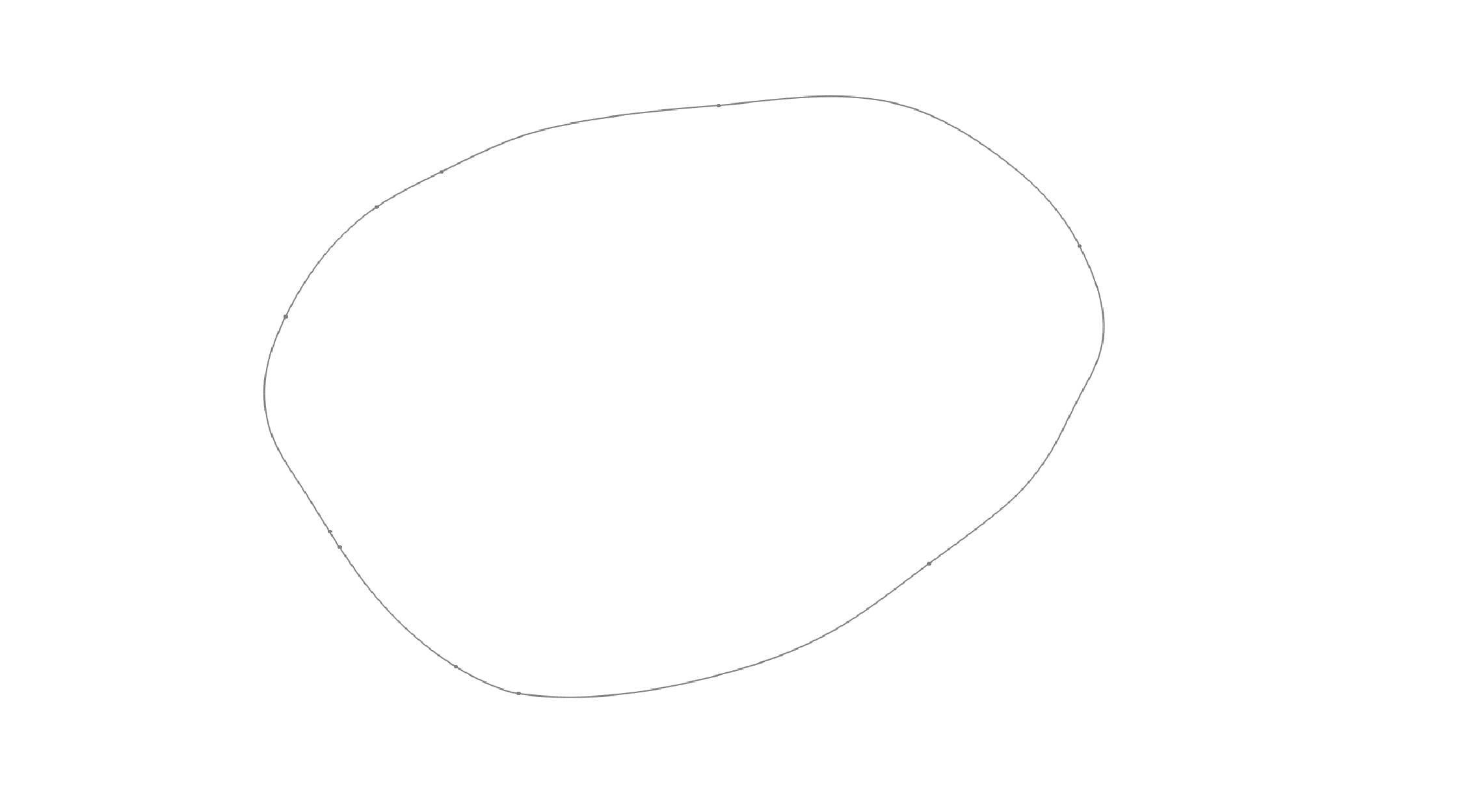

Supplement: Supplementary file 4 — Supporting Information [file ADVS-10-2203062-s013.zip › advs202203062-sup-0004-Supplementary-DataS3/Supplementary Data S3/57.jpg]

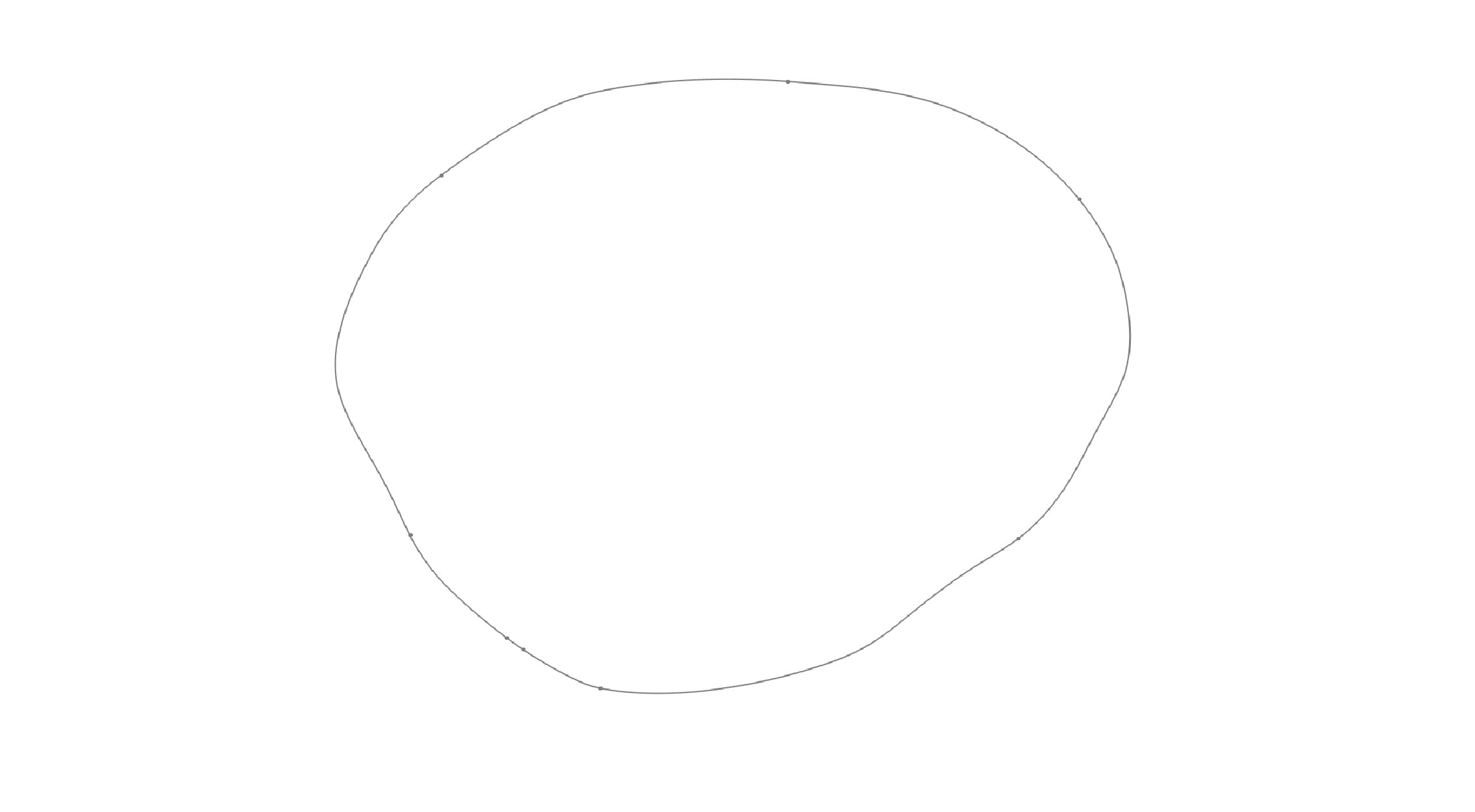

Supplement: Supplementary file 4 — Supporting Information [file ADVS-10-2203062-s013.zip › advs202203062-sup-0004-Supplementary-DataS3/Supplementary Data S3/58.jpg]

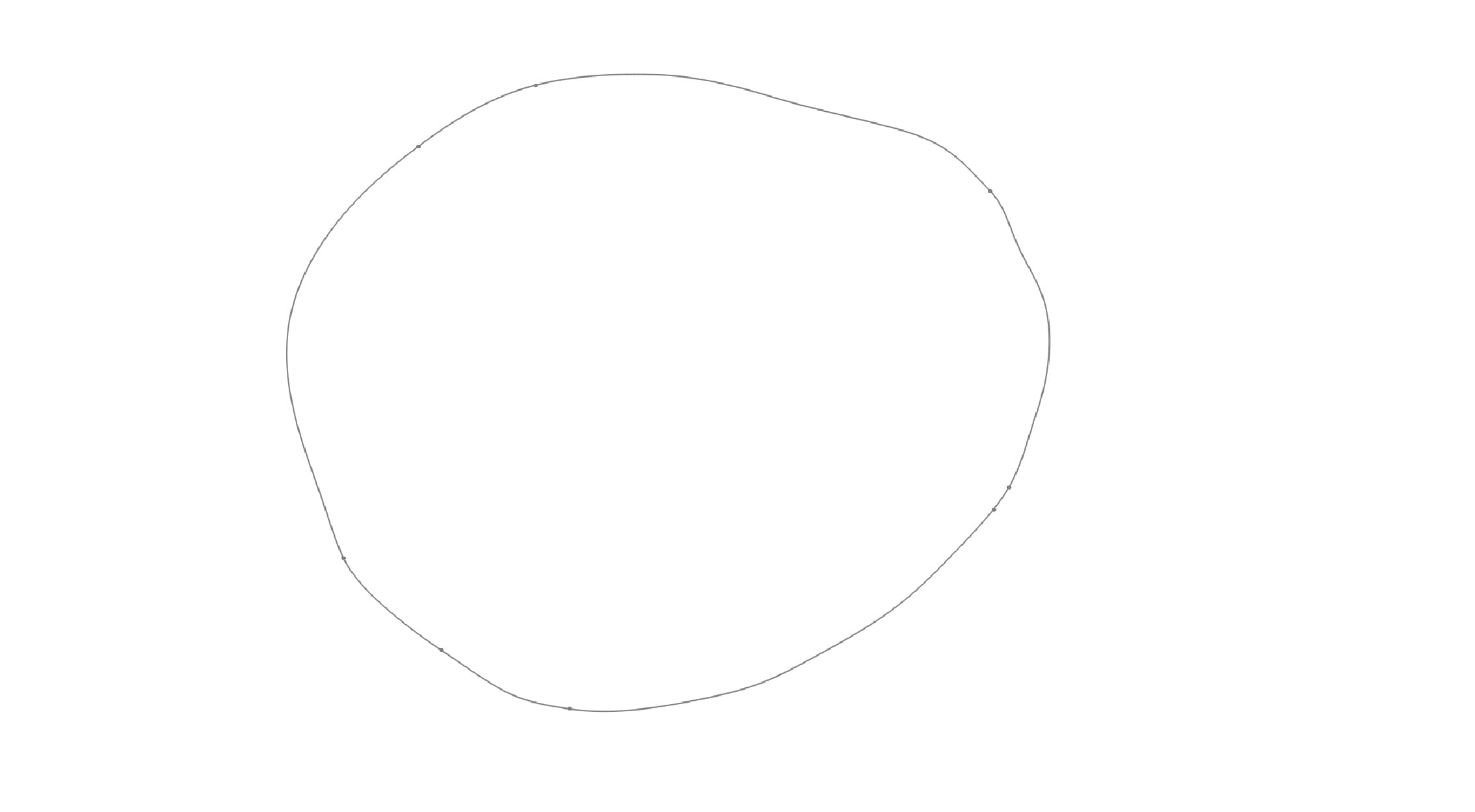

Supplement: Supplementary file 4 — Supporting Information [file ADVS-10-2203062-s013.zip › advs202203062-sup-0004-Supplementary-DataS3/Supplementary Data S3/59.jpg]

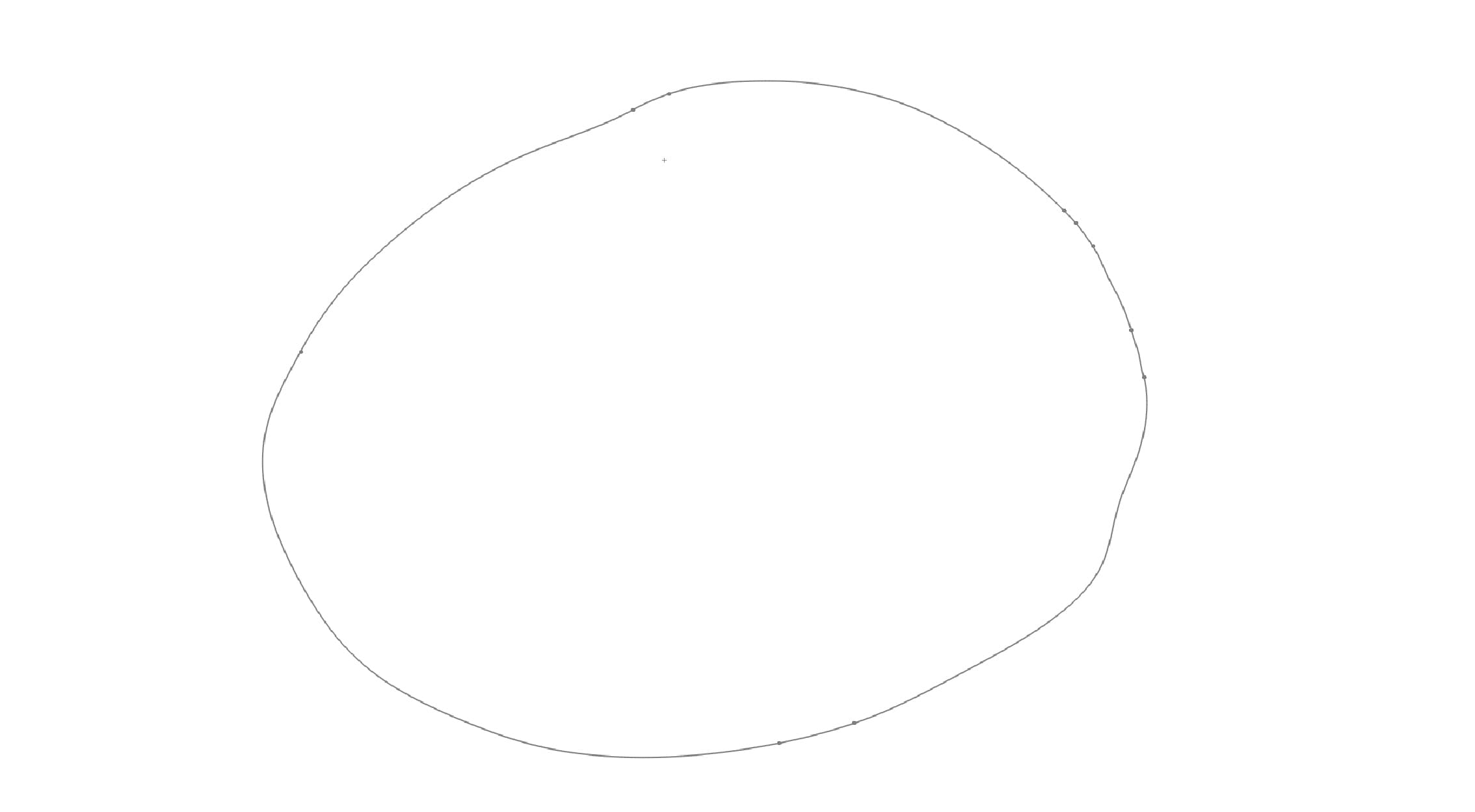

Supplement: Supplementary file 4 — Supporting Information [file ADVS-10-2203062-s013.zip › advs202203062-sup-0004-Supplementary-DataS3/Supplementary Data S3/6.jpg]

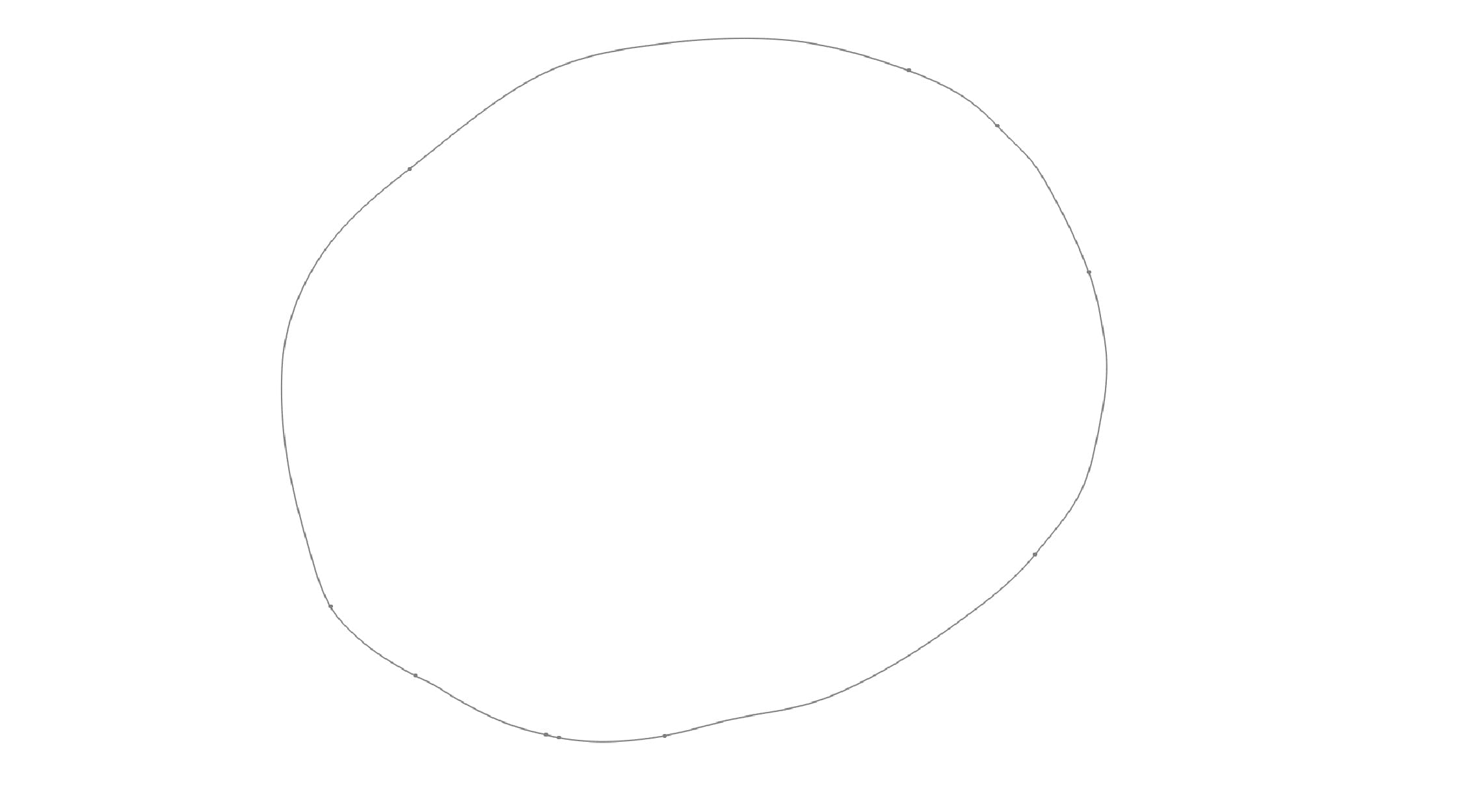

Supplement: Supplementary file 4 — Supporting Information [file ADVS-10-2203062-s013.zip › advs202203062-sup-0004-Supplementary-DataS3/Supplementary Data S3/60.jpg]

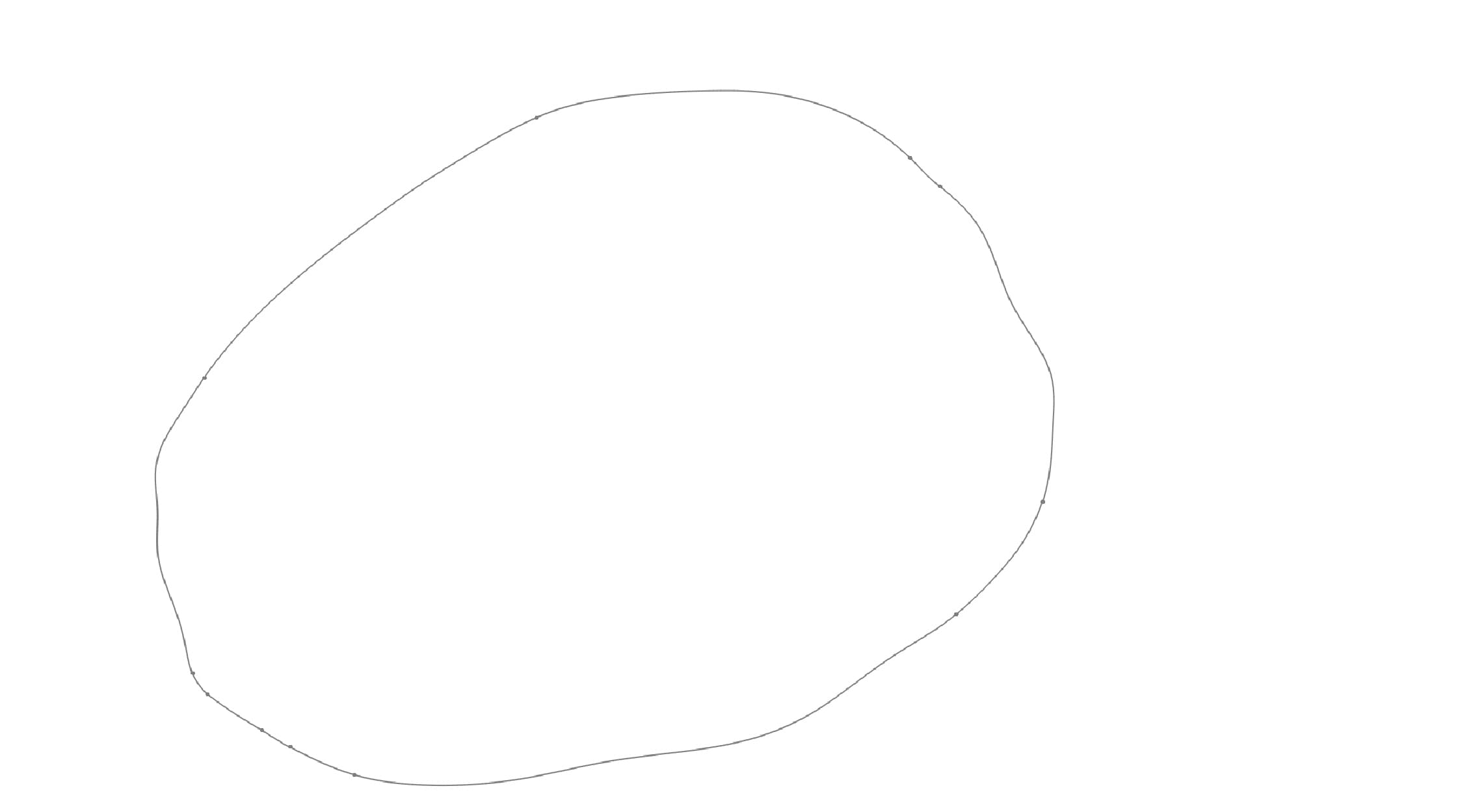

Supplement: Supplementary file 4 — Supporting Information [file ADVS-10-2203062-s013.zip › advs202203062-sup-0004-Supplementary-DataS3/Supplementary Data S3/61.jpg]

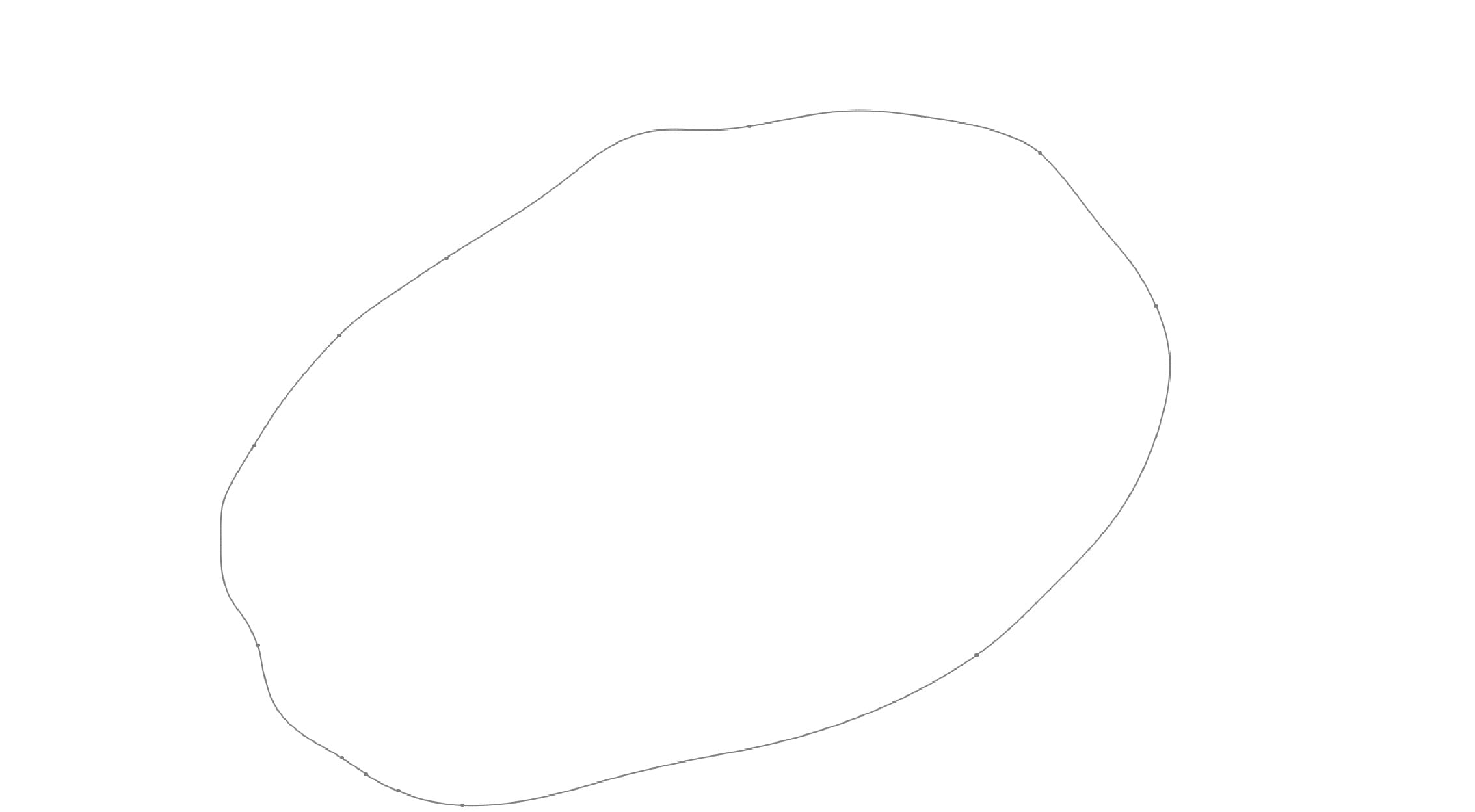

Supplement: Supplementary file 4 — Supporting Information [file ADVS-10-2203062-s013.zip › advs202203062-sup-0004-Supplementary-DataS3/Supplementary Data S3/62.jpg]

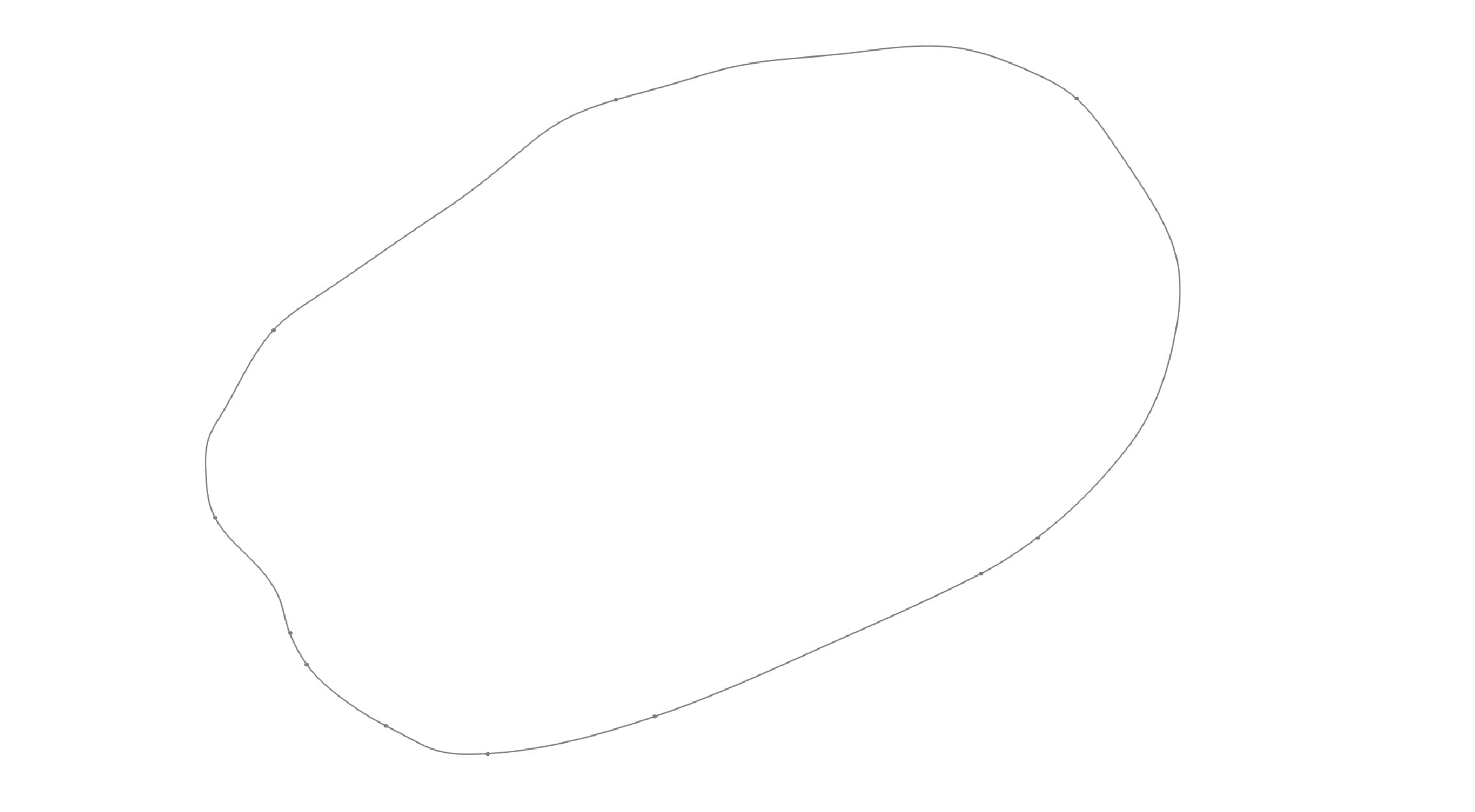

Supplement: Supplementary file 4 — Supporting Information [file ADVS-10-2203062-s013.zip › advs202203062-sup-0004-Supplementary-DataS3/Supplementary Data S3/63.jpg]

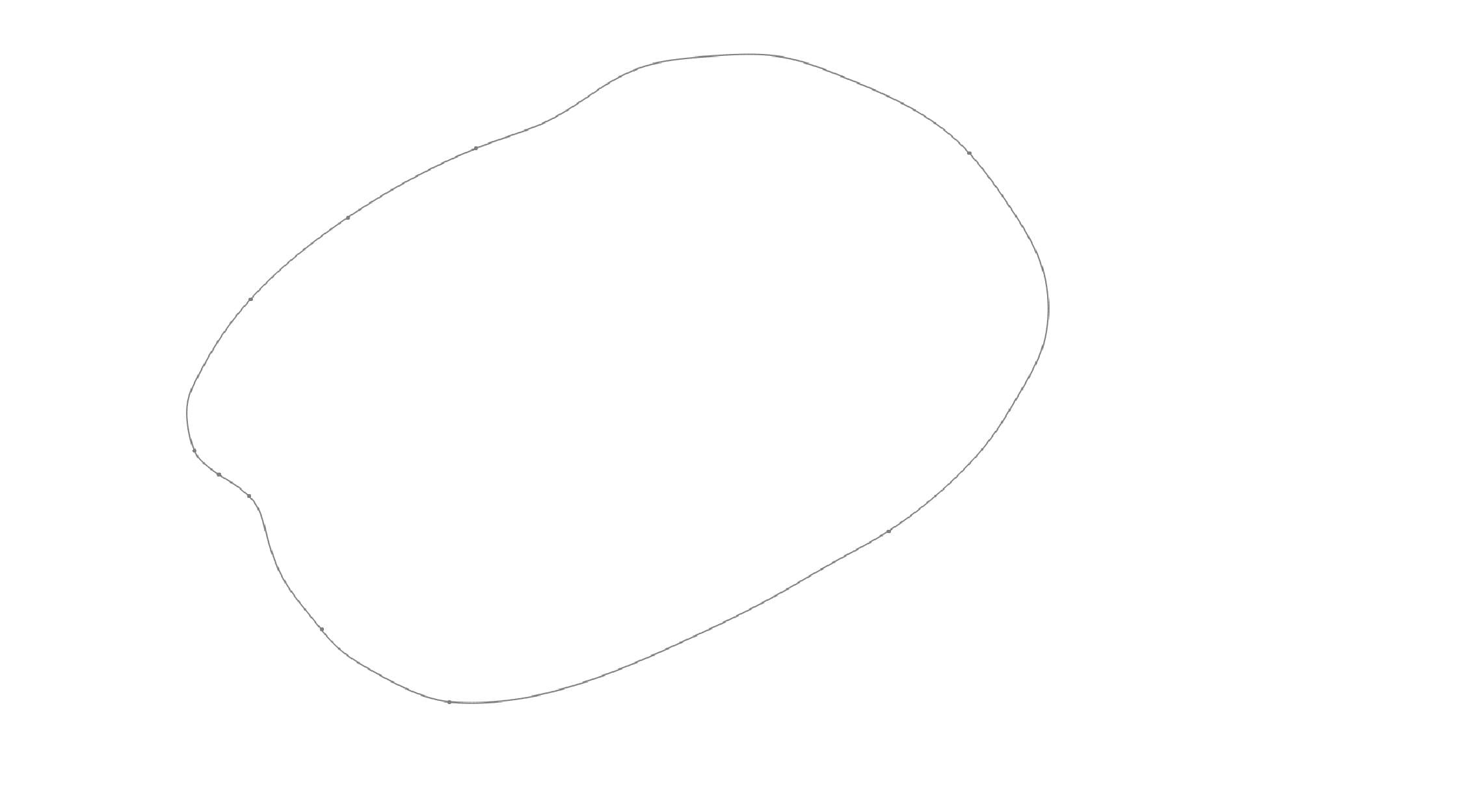

Supplement: Supplementary file 4 — Supporting Information [file ADVS-10-2203062-s013.zip › advs202203062-sup-0004-Supplementary-DataS3/Supplementary Data S3/64.jpg]

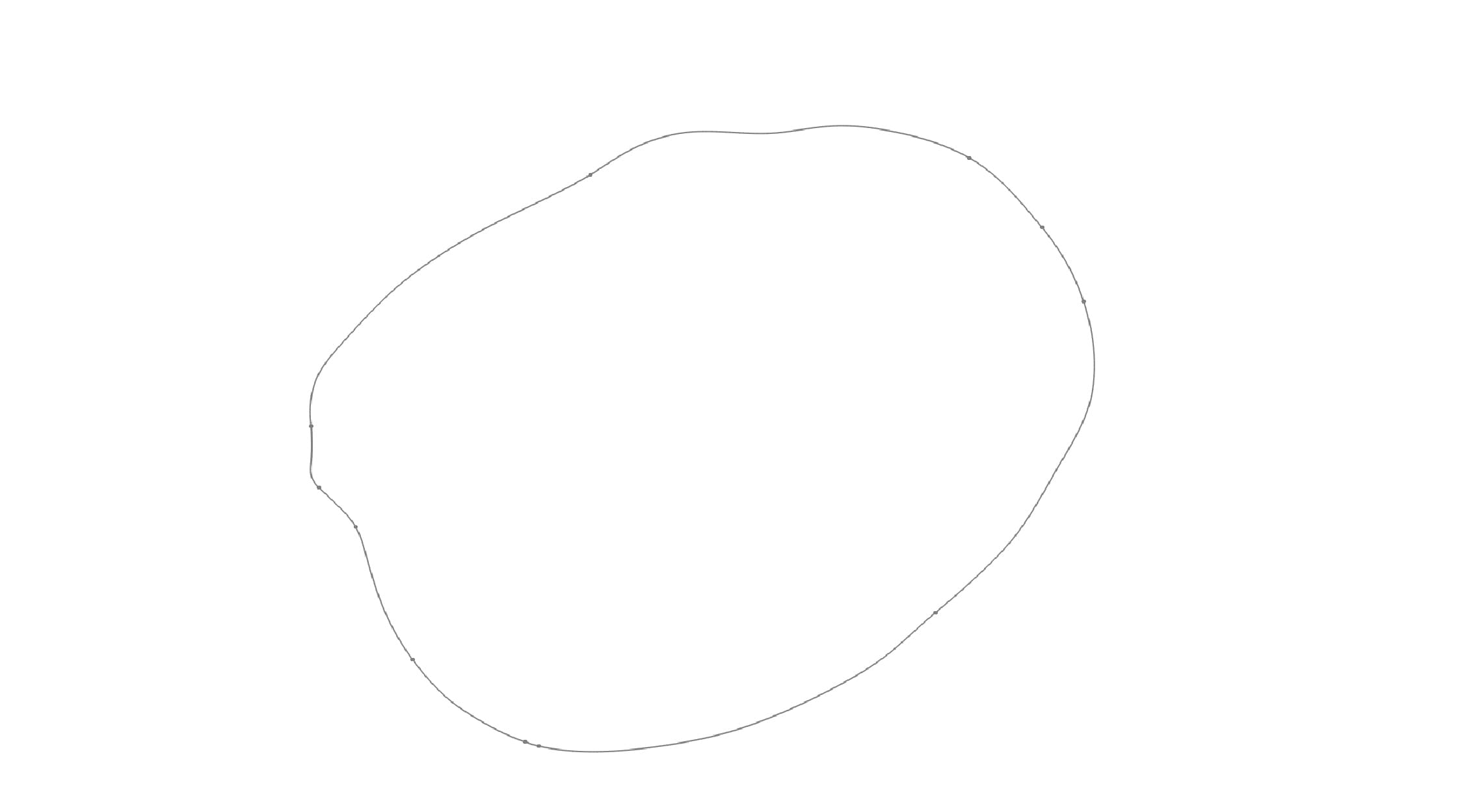

Supplement: Supplementary file 4 — Supporting Information [file ADVS-10-2203062-s013.zip › advs202203062-sup-0004-Supplementary-DataS3/Supplementary Data S3/65.jpg]

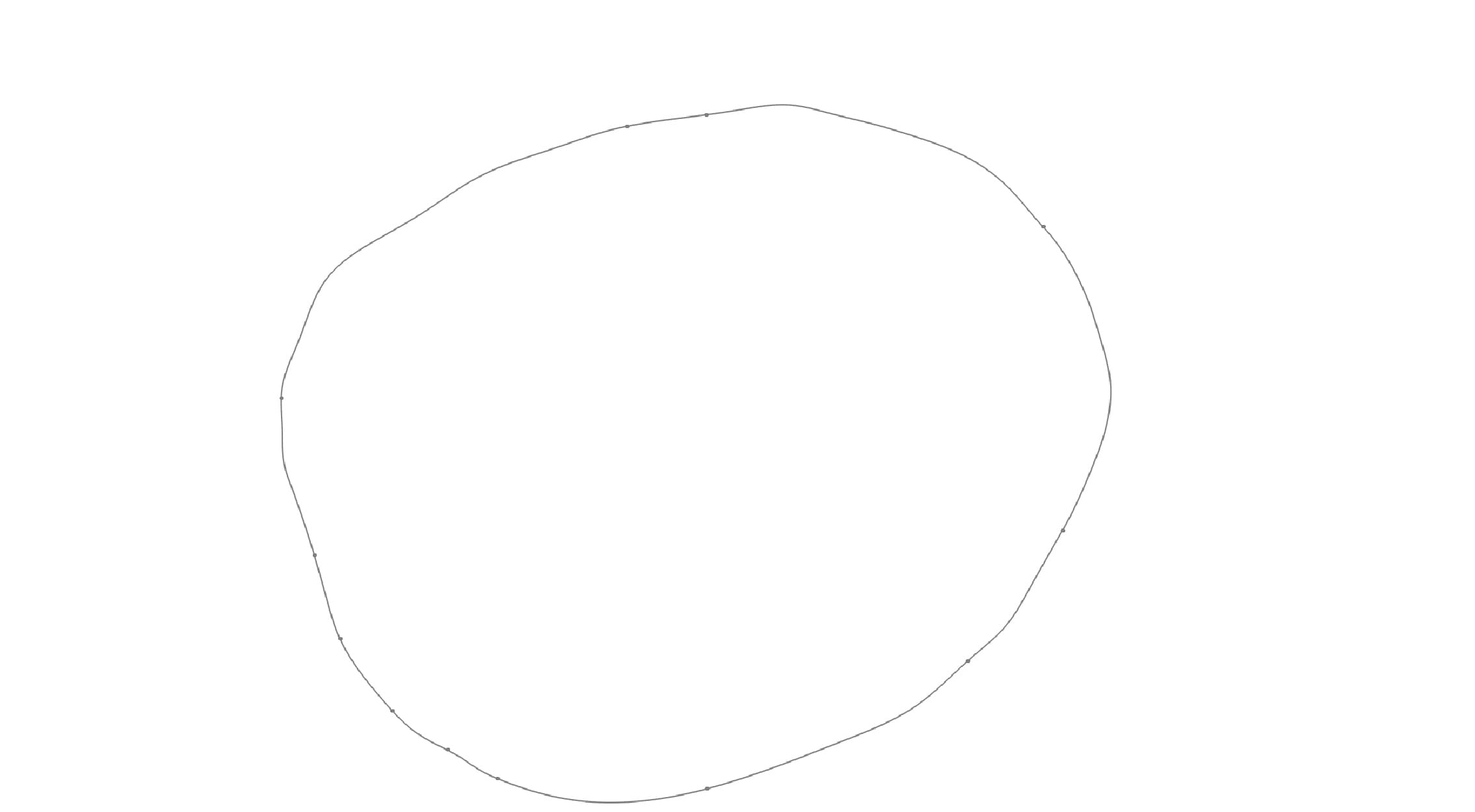

Supplement: Supplementary file 4 — Supporting Information [file ADVS-10-2203062-s013.zip › advs202203062-sup-0004-Supplementary-DataS3/Supplementary Data S3/66.jpg]

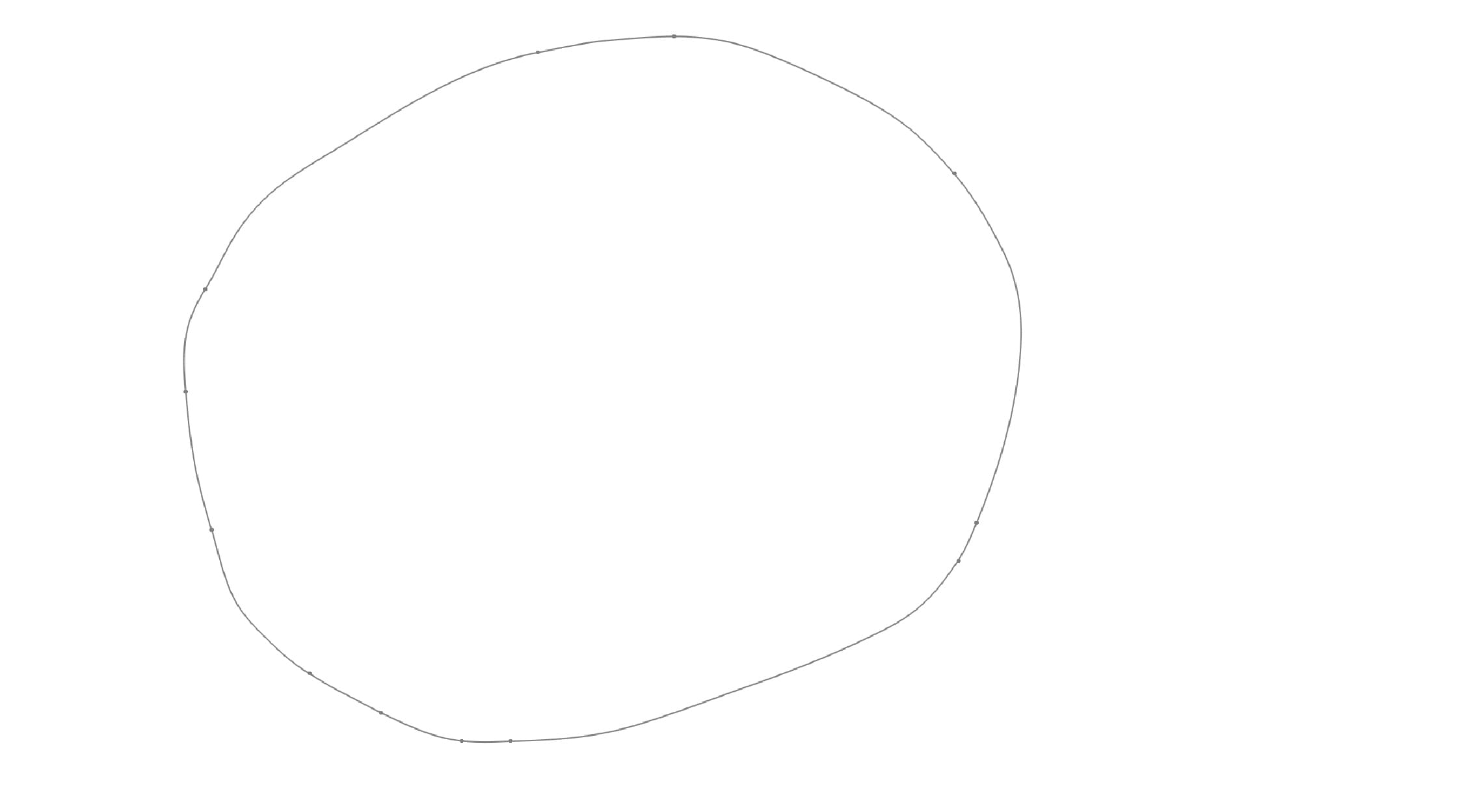

Supplement: Supplementary file 4 — Supporting Information [file ADVS-10-2203062-s013.zip › advs202203062-sup-0004-Supplementary-DataS3/Supplementary Data S3/67.jpg]

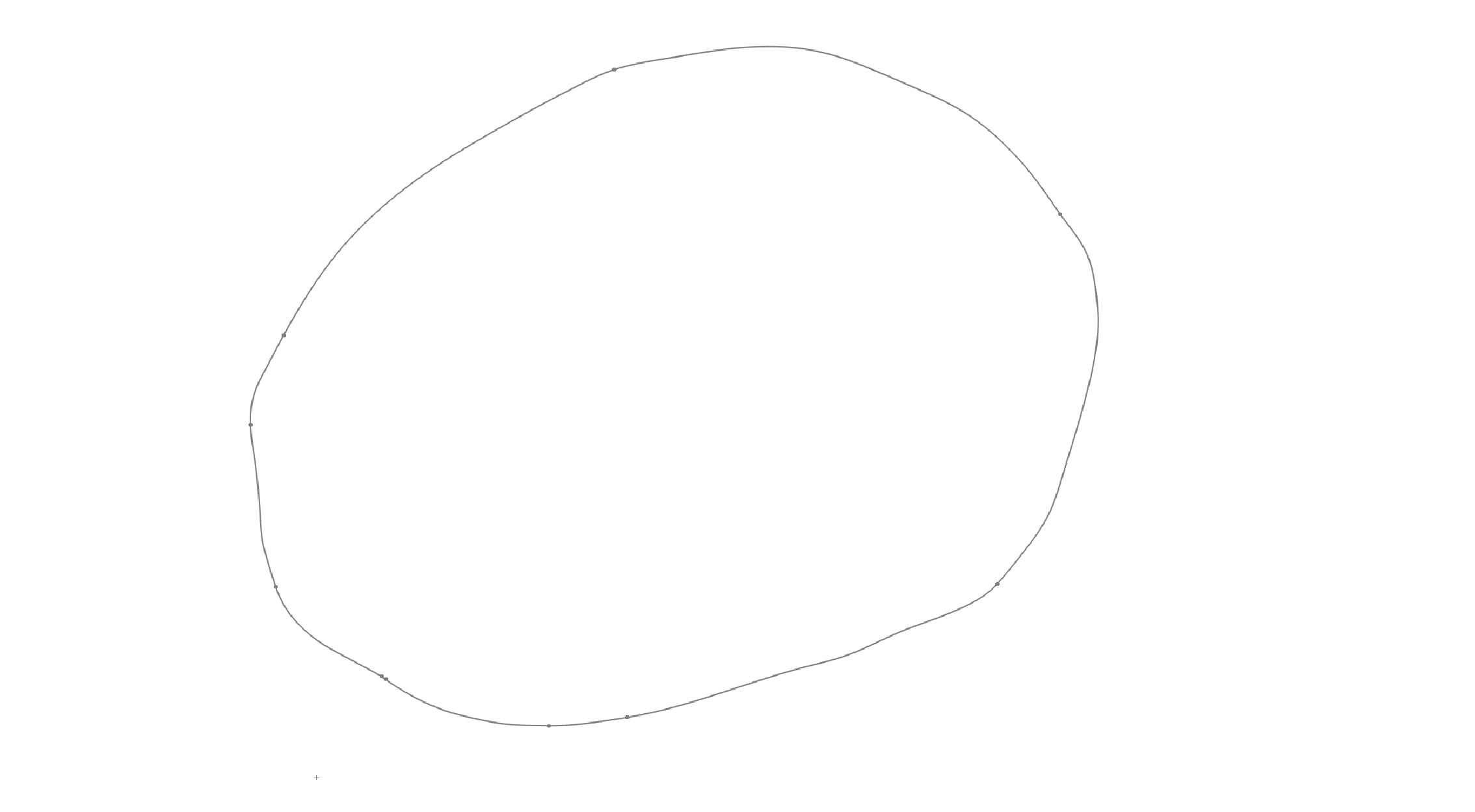

Supplement: Supplementary file 4 — Supporting Information [file ADVS-10-2203062-s013.zip › advs202203062-sup-0004-Supplementary-DataS3/Supplementary Data S3/68.jpg]

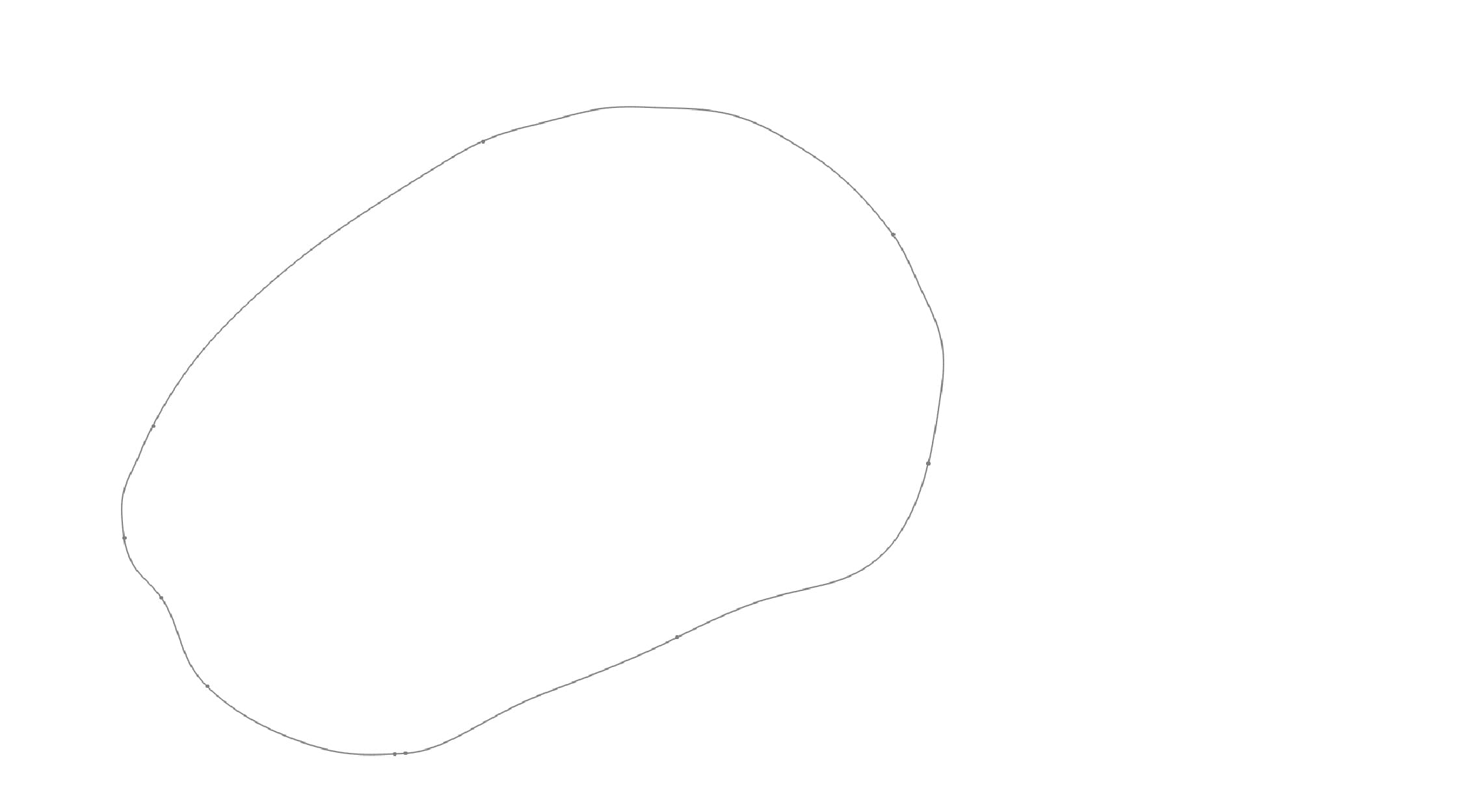

Supplement: Supplementary file 4 — Supporting Information [file ADVS-10-2203062-s013.zip › advs202203062-sup-0004-Supplementary-DataS3/Supplementary Data S3/69.jpg]

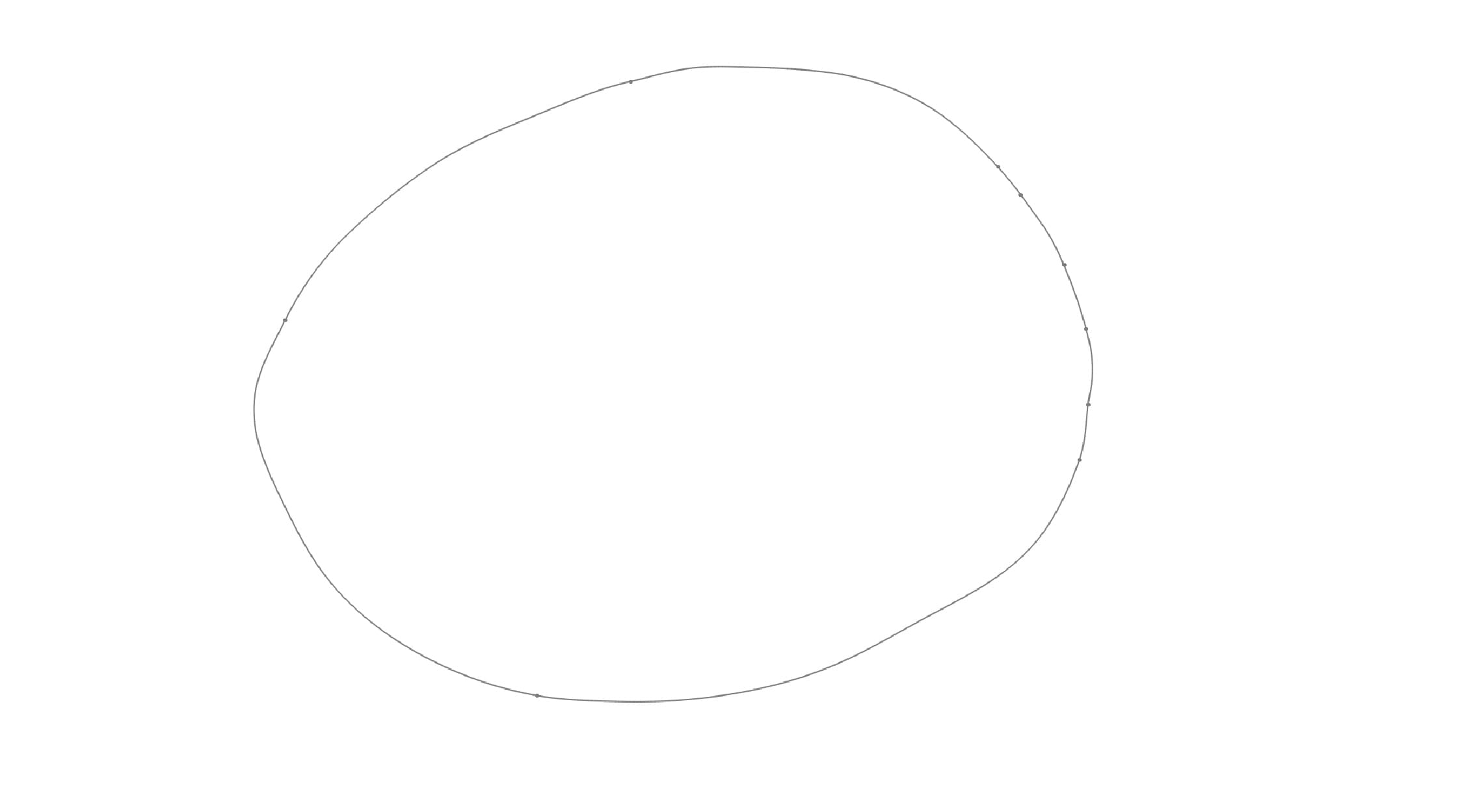

Supplement: Supplementary file 4 — Supporting Information [file ADVS-10-2203062-s013.zip › advs202203062-sup-0004-Supplementary-DataS3/Supplementary Data S3/7.jpg]

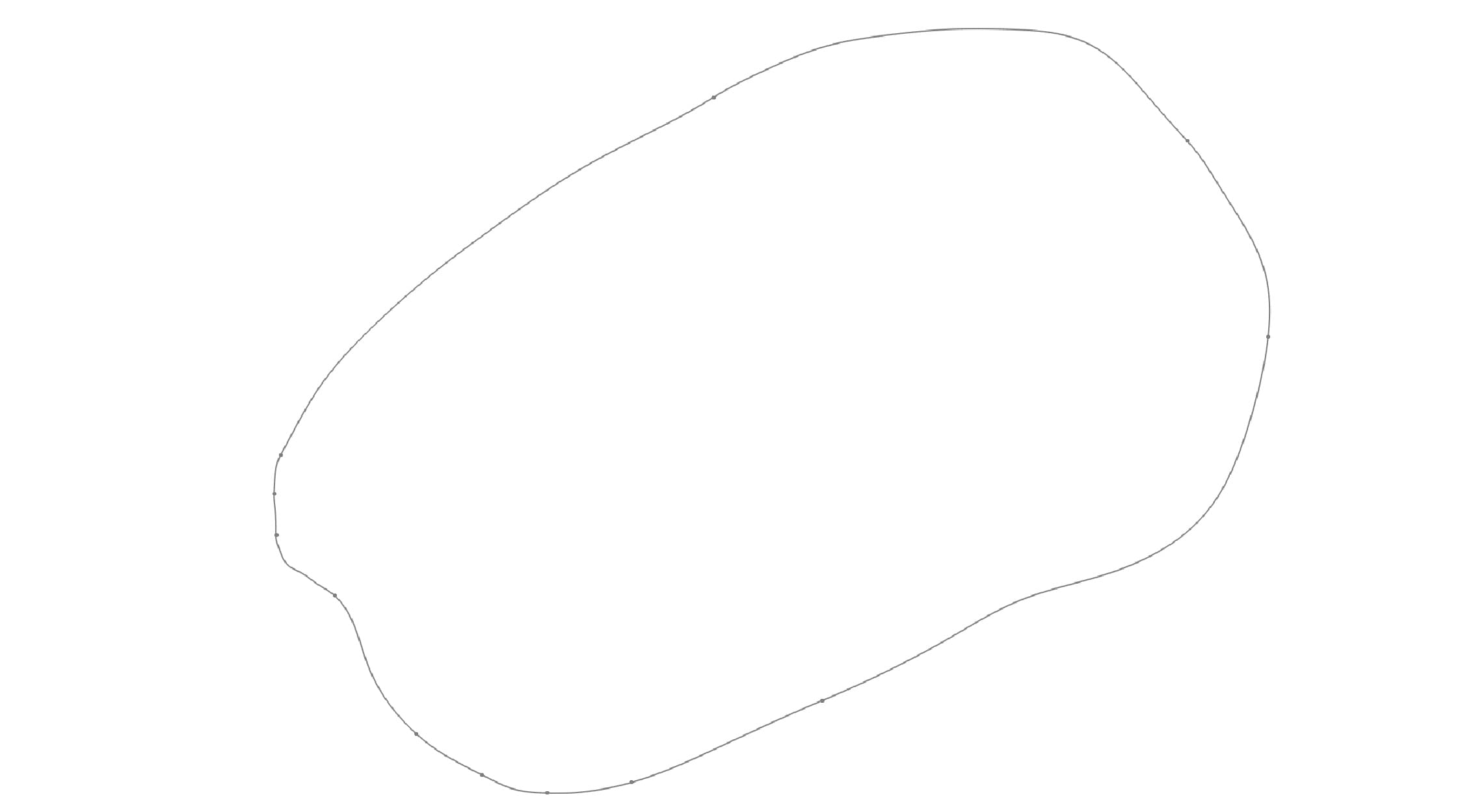

Supplement: Supplementary file 4 — Supporting Information [file ADVS-10-2203062-s013.zip › advs202203062-sup-0004-Supplementary-DataS3/Supplementary Data S3/70.jpg]

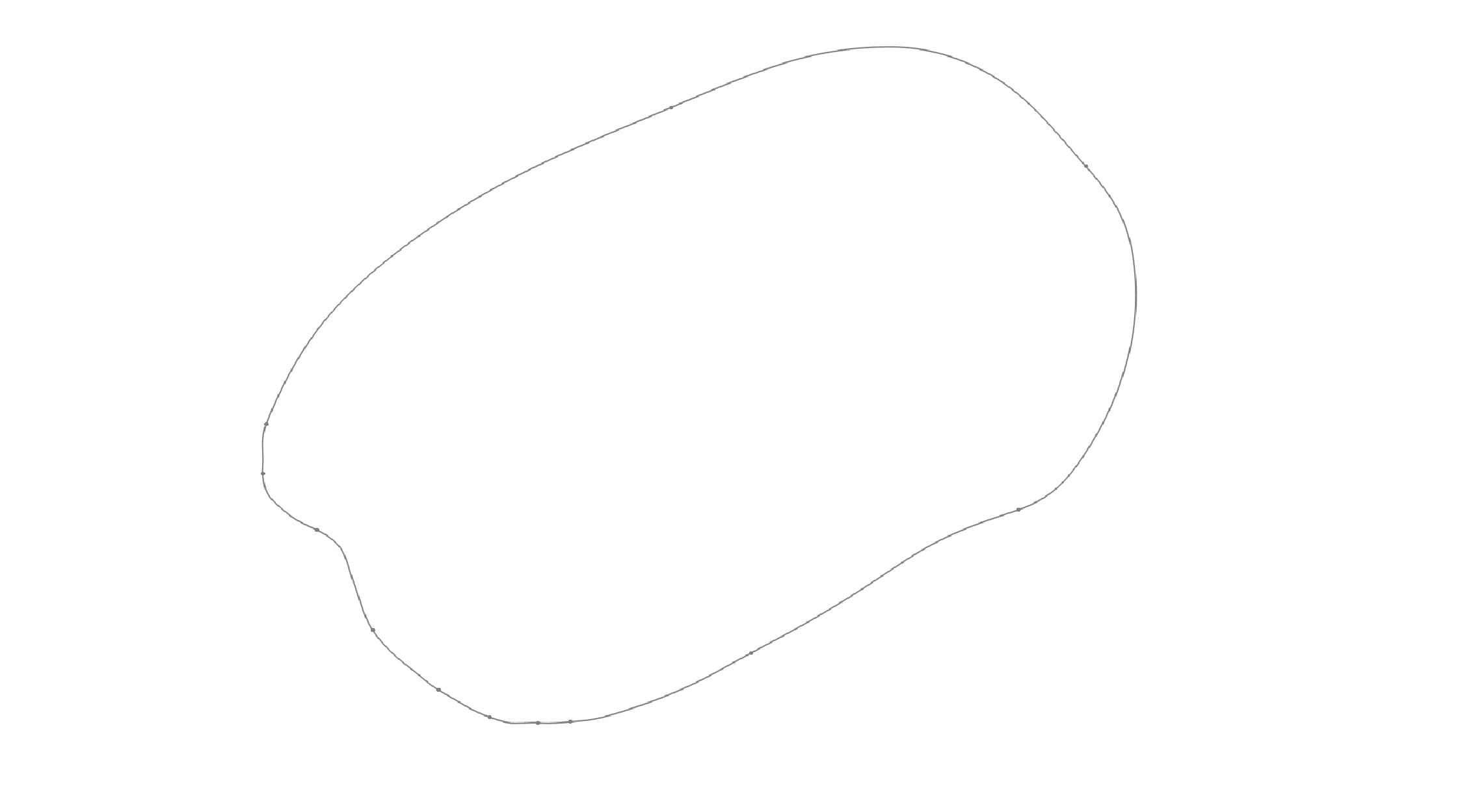

Supplement: Supplementary file 4 — Supporting Information [file ADVS-10-2203062-s013.zip › advs202203062-sup-0004-Supplementary-DataS3/Supplementary Data S3/71.jpg]

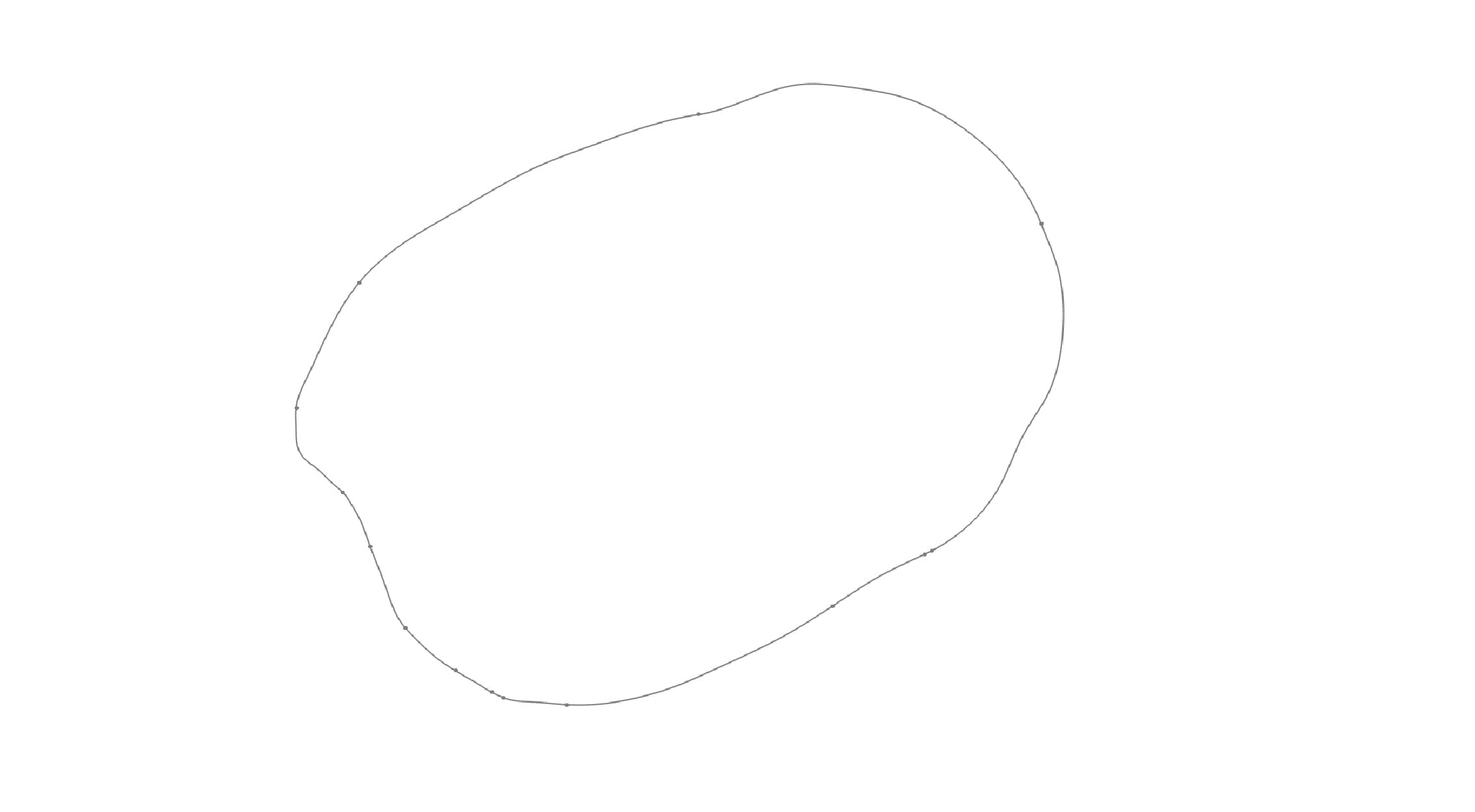

Supplement: Supplementary file 4 — Supporting Information [file ADVS-10-2203062-s013.zip › advs202203062-sup-0004-Supplementary-DataS3/Supplementary Data S3/72.jpg]

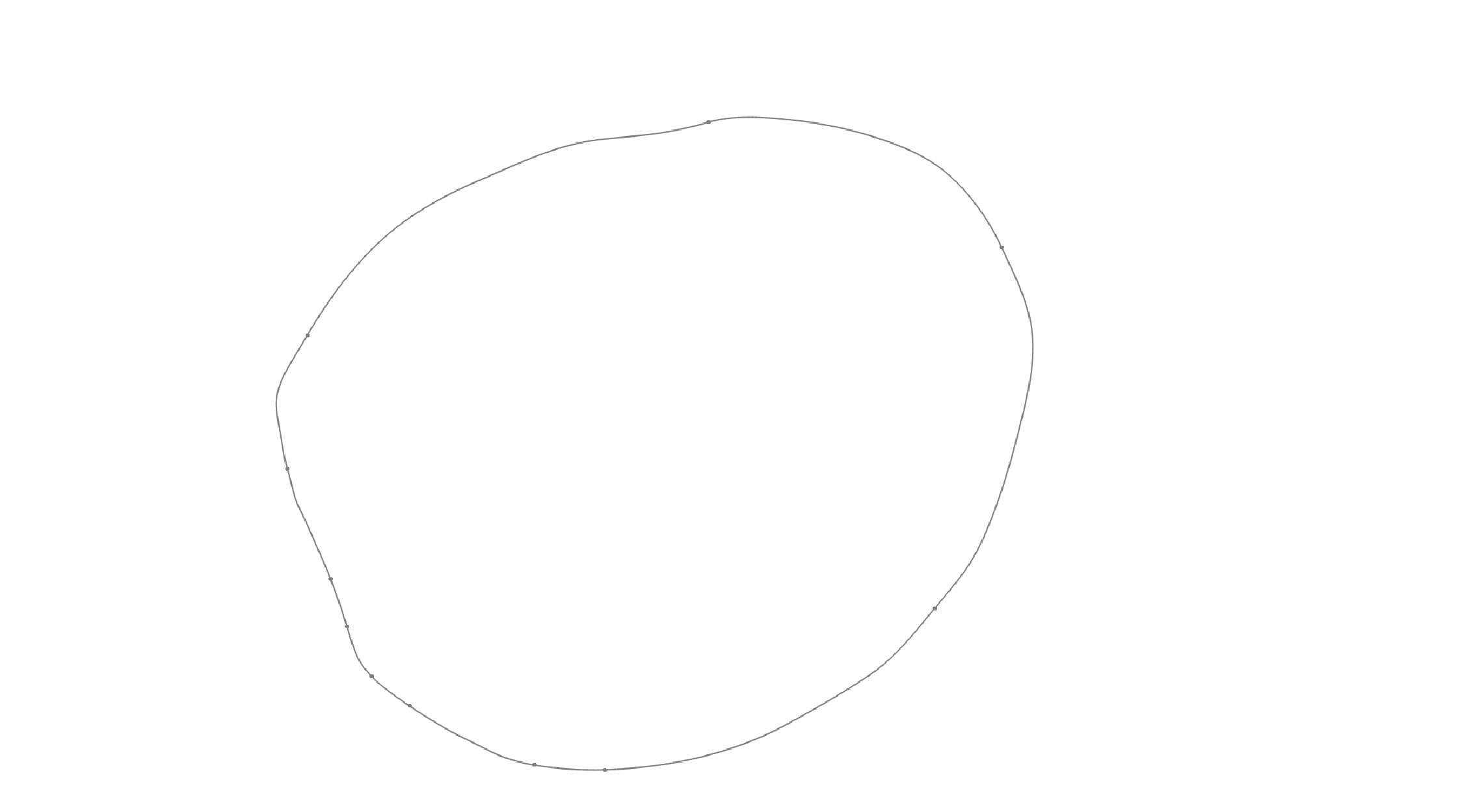

Supplement: Supplementary file 4 — Supporting Information [file ADVS-10-2203062-s013.zip › advs202203062-sup-0004-Supplementary-DataS3/Supplementary Data S3/73.jpg]

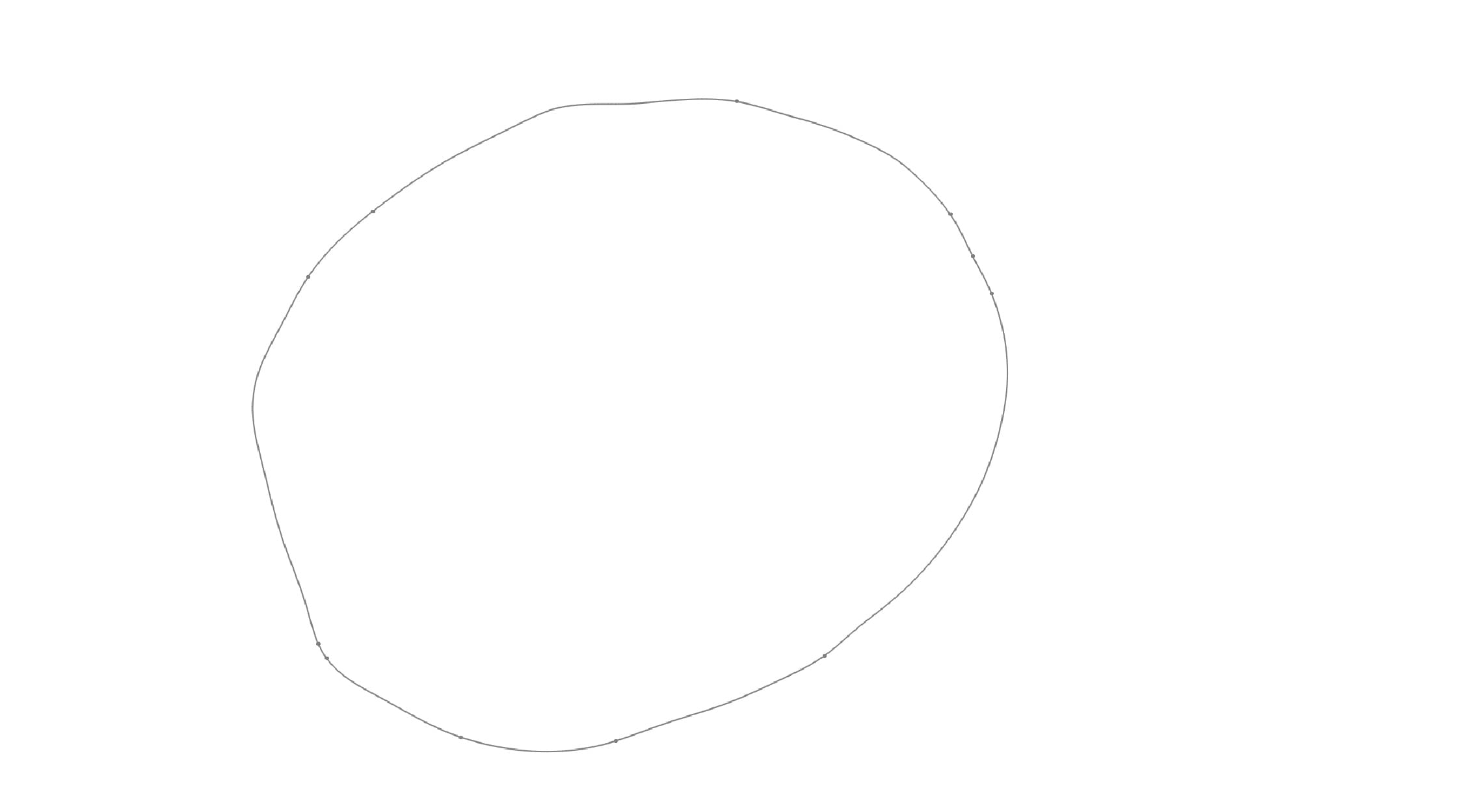

Supplement: Supplementary file 4 — Supporting Information [file ADVS-10-2203062-s013.zip › advs202203062-sup-0004-Supplementary-DataS3/Supplementary Data S3/74.jpg]

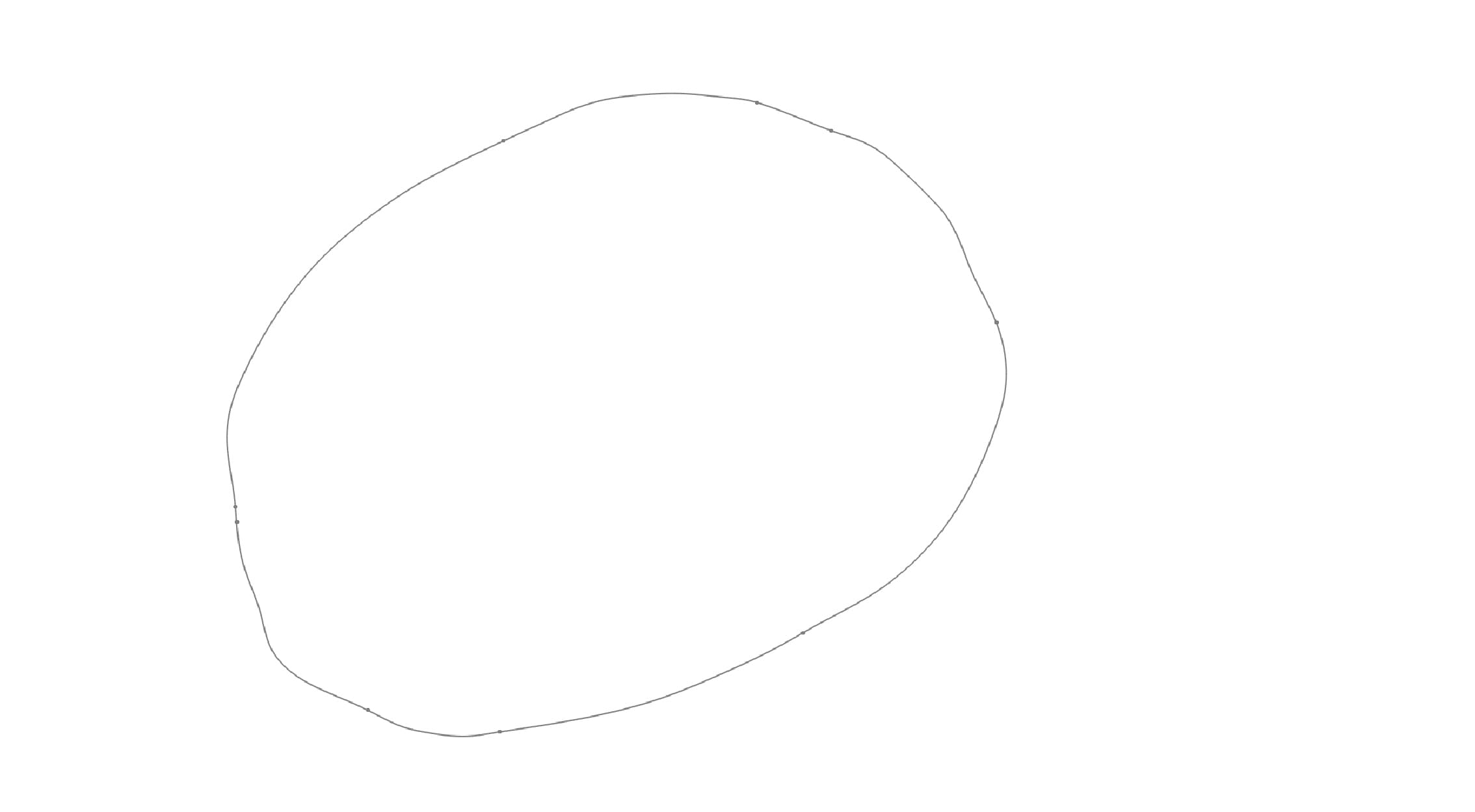

Supplement: Supplementary file 4 — Supporting Information [file ADVS-10-2203062-s013.zip › advs202203062-sup-0004-Supplementary-DataS3/Supplementary Data S3/75.jpg]

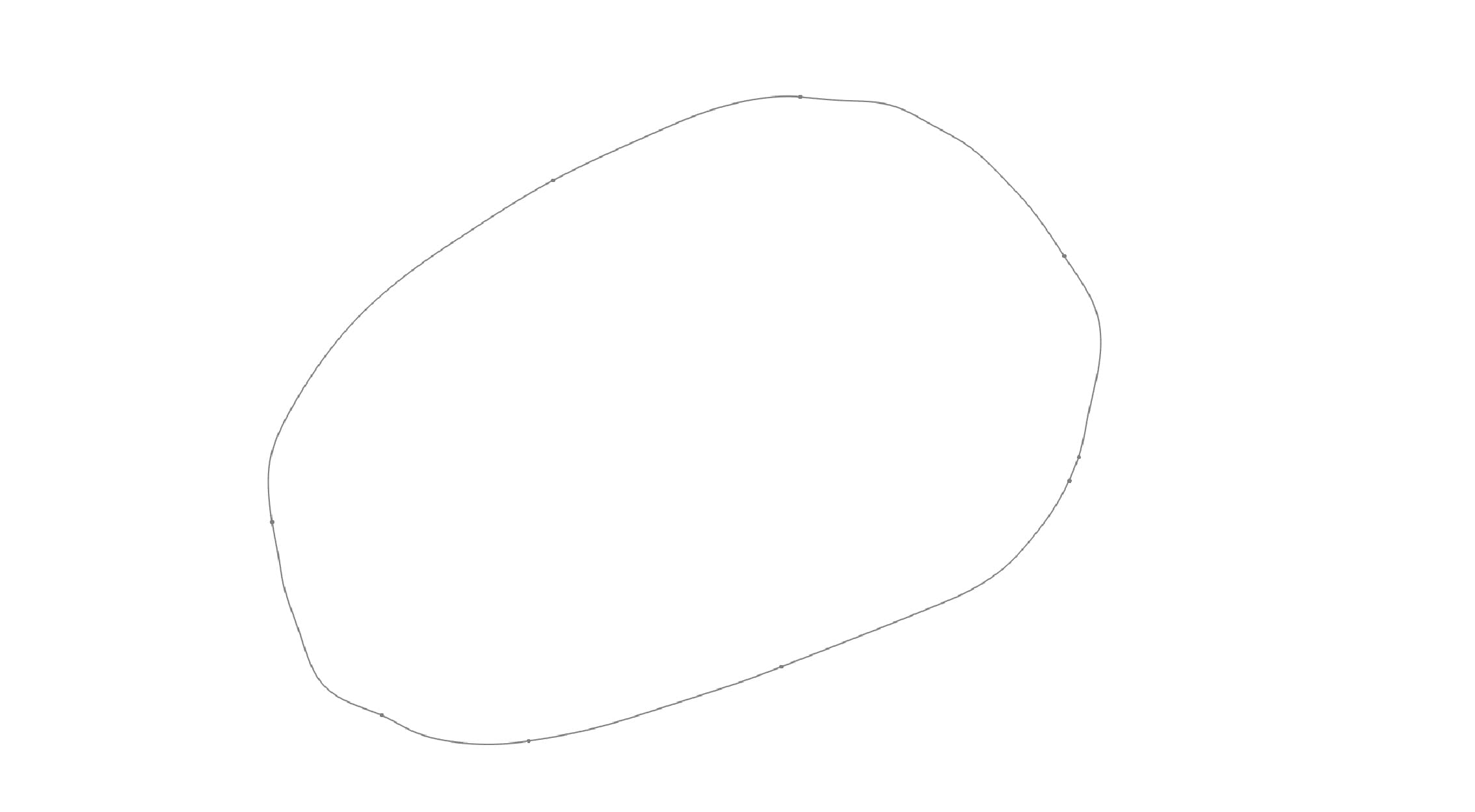

Supplement: Supplementary file 4 — Supporting Information [file ADVS-10-2203062-s013.zip › advs202203062-sup-0004-Supplementary-DataS3/Supplementary Data S3/76.jpg]

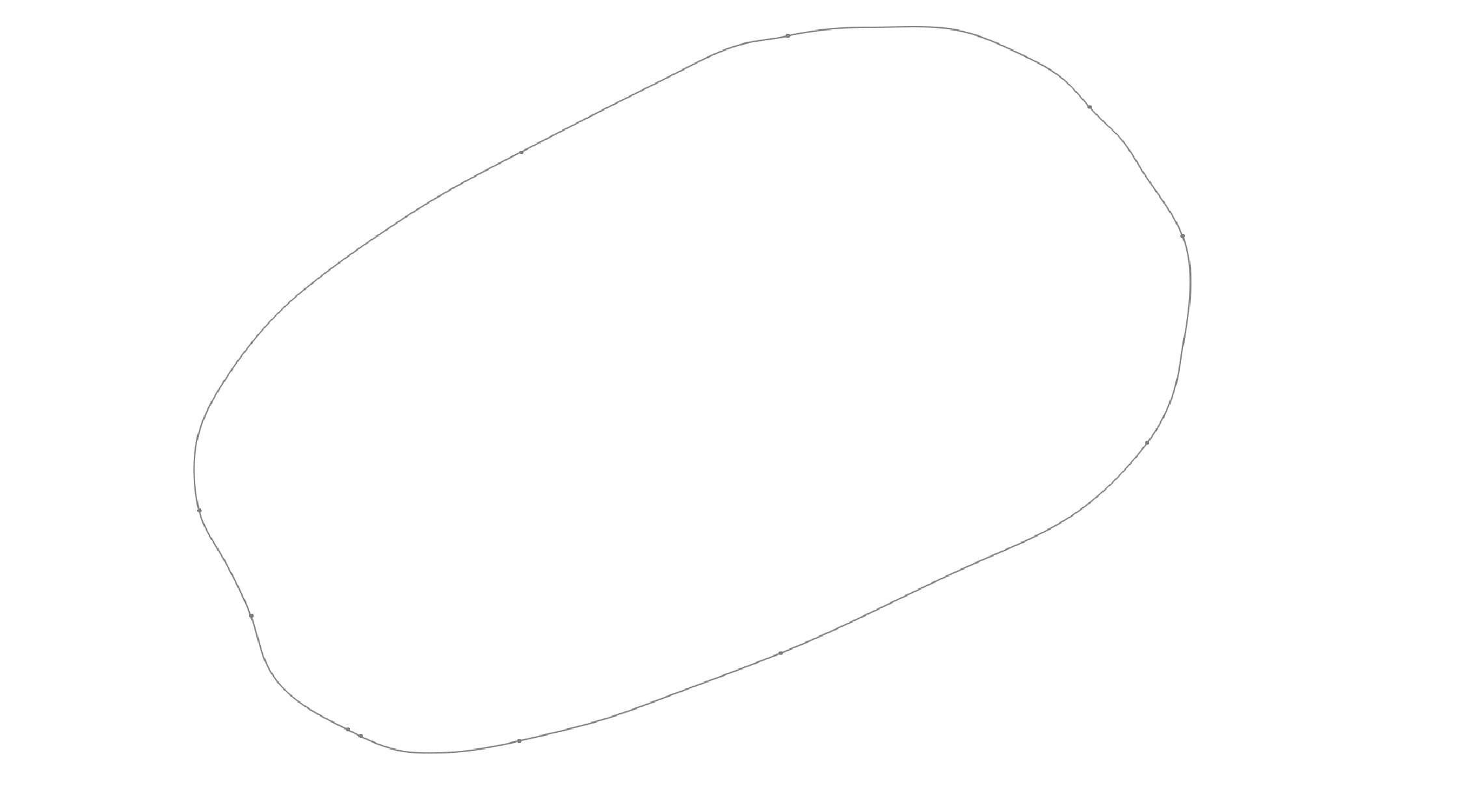

Supplement: Supplementary file 4 — Supporting Information [file ADVS-10-2203062-s013.zip › advs202203062-sup-0004-Supplementary-DataS3/Supplementary Data S3/77.jpg]

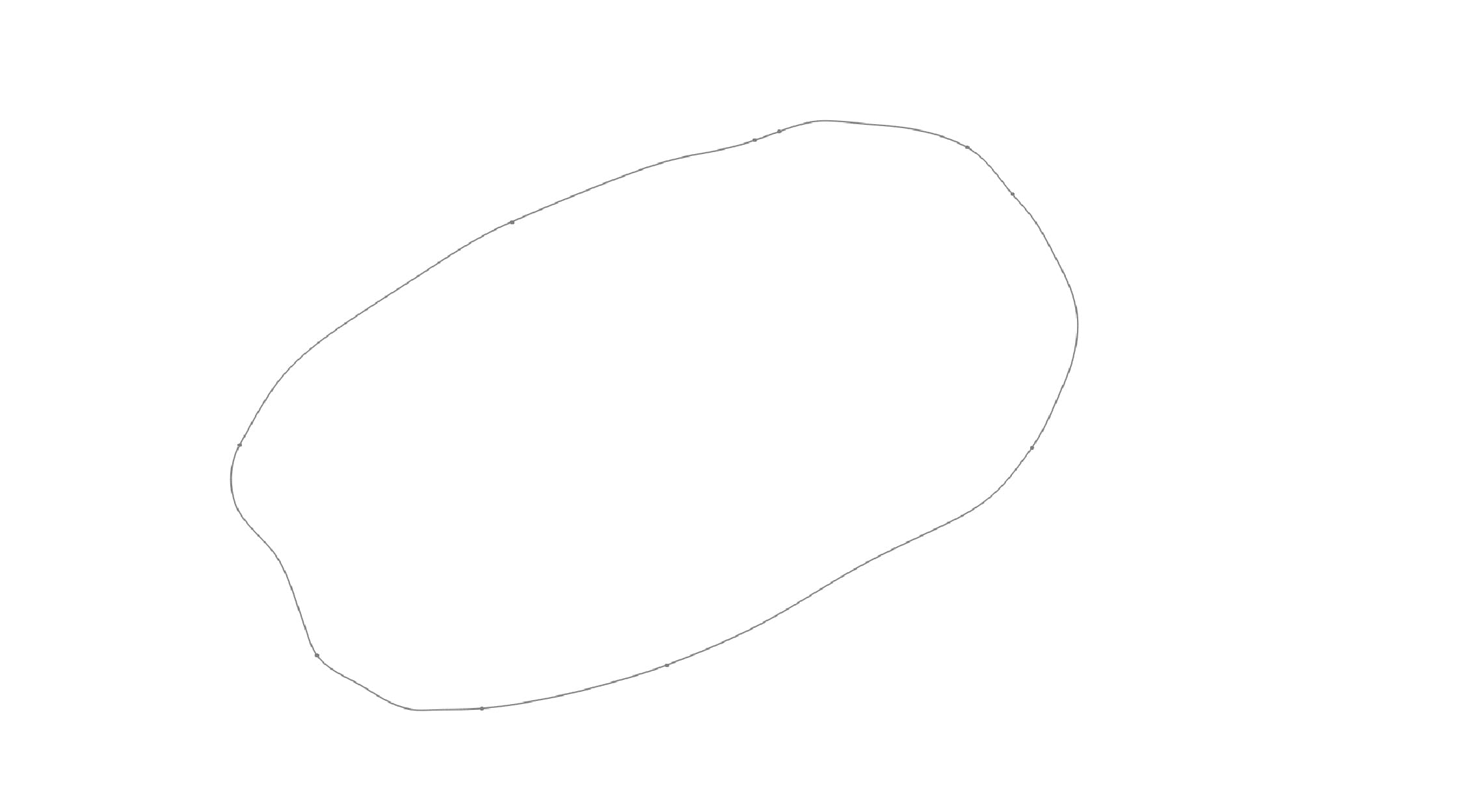

Supplement: Supplementary file 4 — Supporting Information [file ADVS-10-2203062-s013.zip › advs202203062-sup-0004-Supplementary-DataS3/Supplementary Data S3/78.jpg]

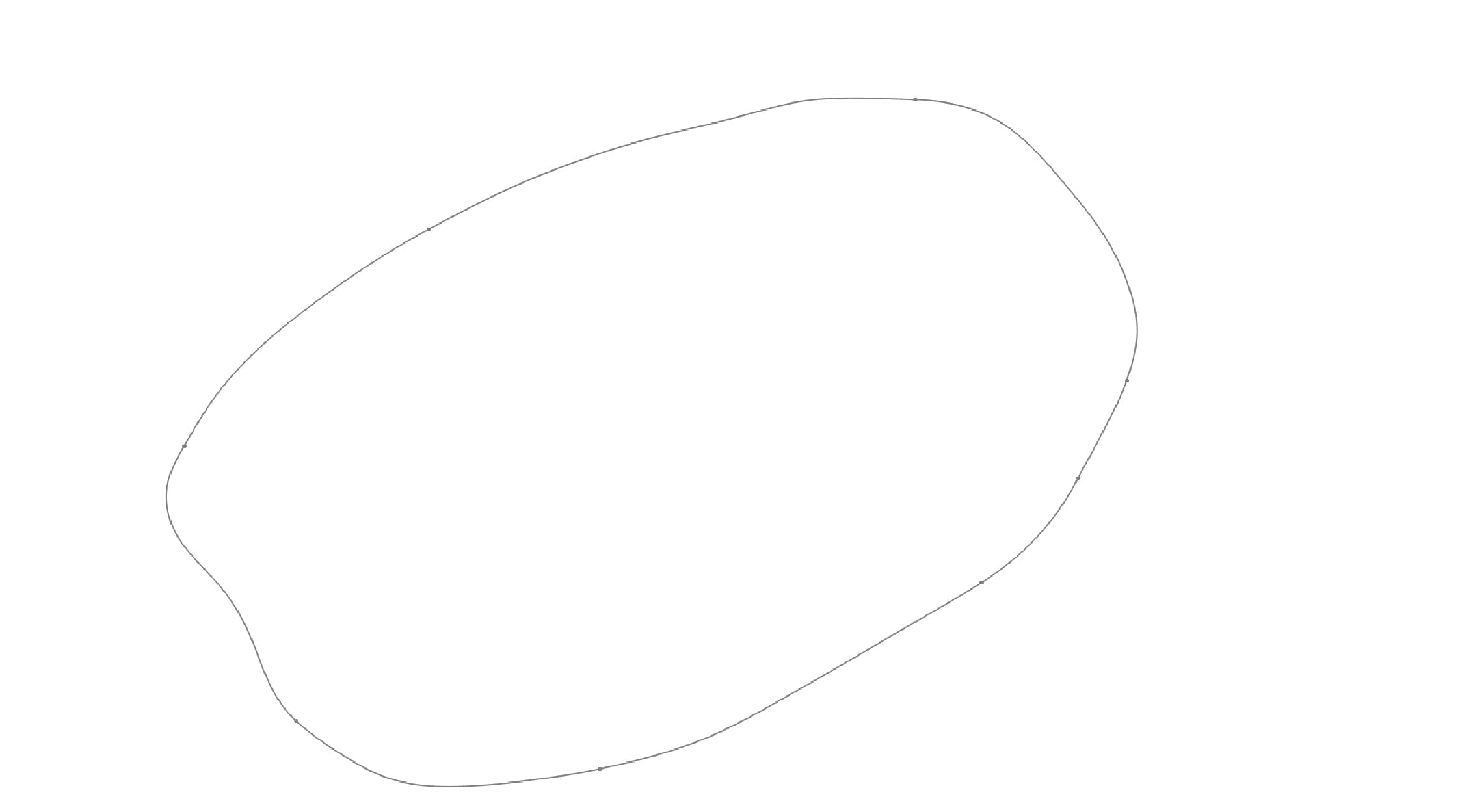

Supplement: Supplementary file 4 — Supporting Information [file ADVS-10-2203062-s013.zip › advs202203062-sup-0004-Supplementary-DataS3/Supplementary Data S3/79.jpg]

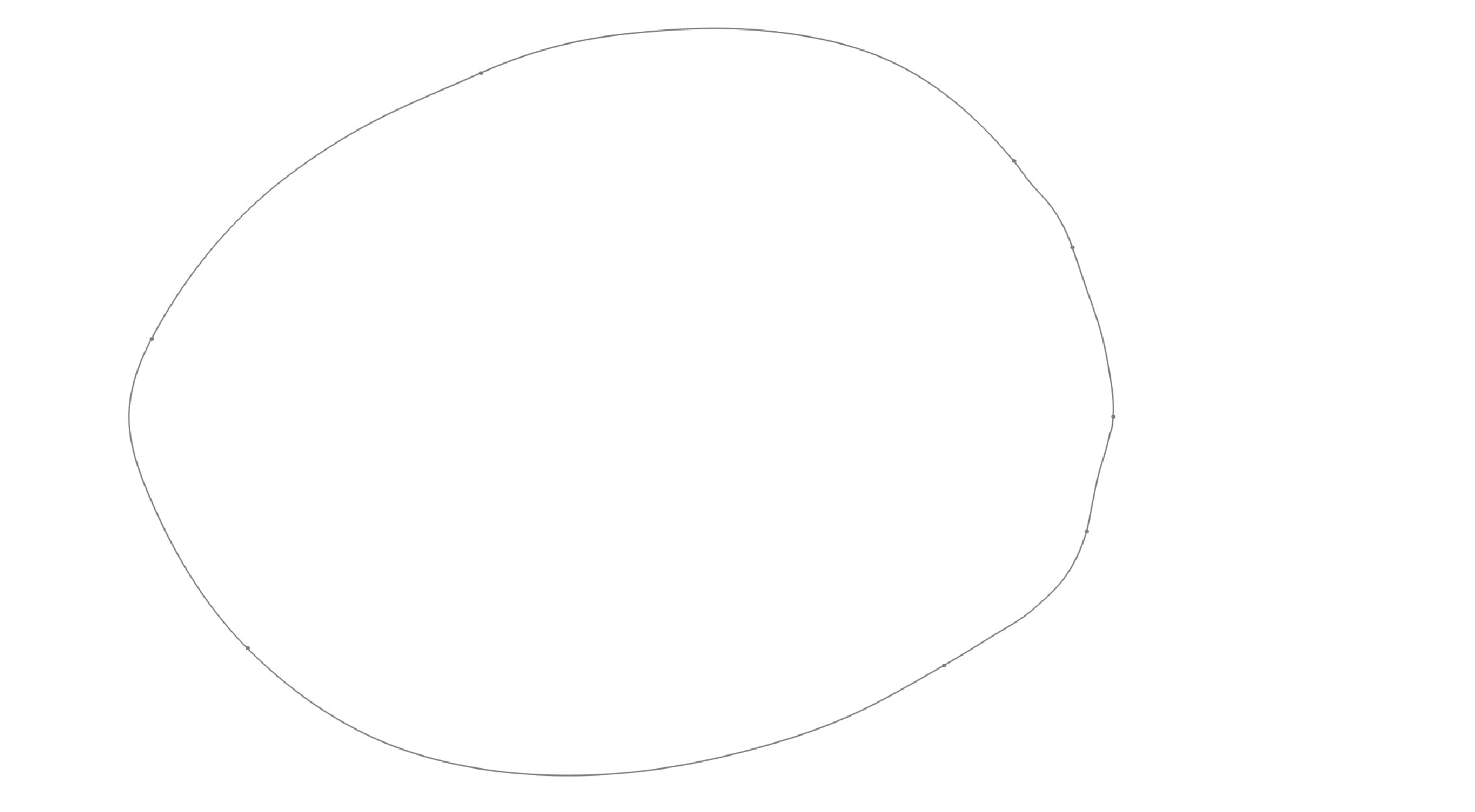

Supplement: Supplementary file 4 — Supporting Information [file ADVS-10-2203062-s013.zip › advs202203062-sup-0004-Supplementary-DataS3/Supplementary Data S3/8.jpg]

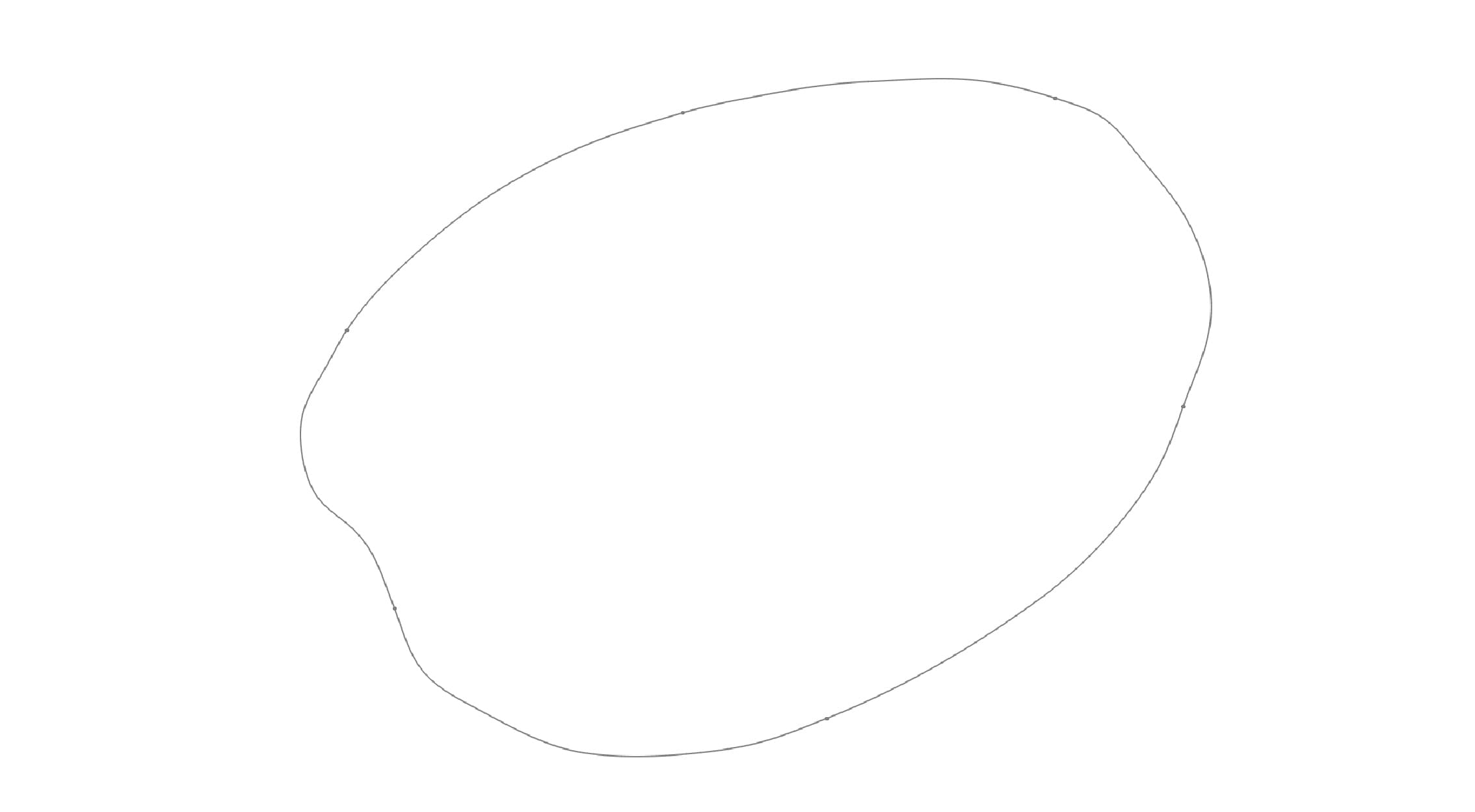

Supplement: Supplementary file 4 — Supporting Information [file ADVS-10-2203062-s013.zip › advs202203062-sup-0004-Supplementary-DataS3/Supplementary Data S3/80.jpg]

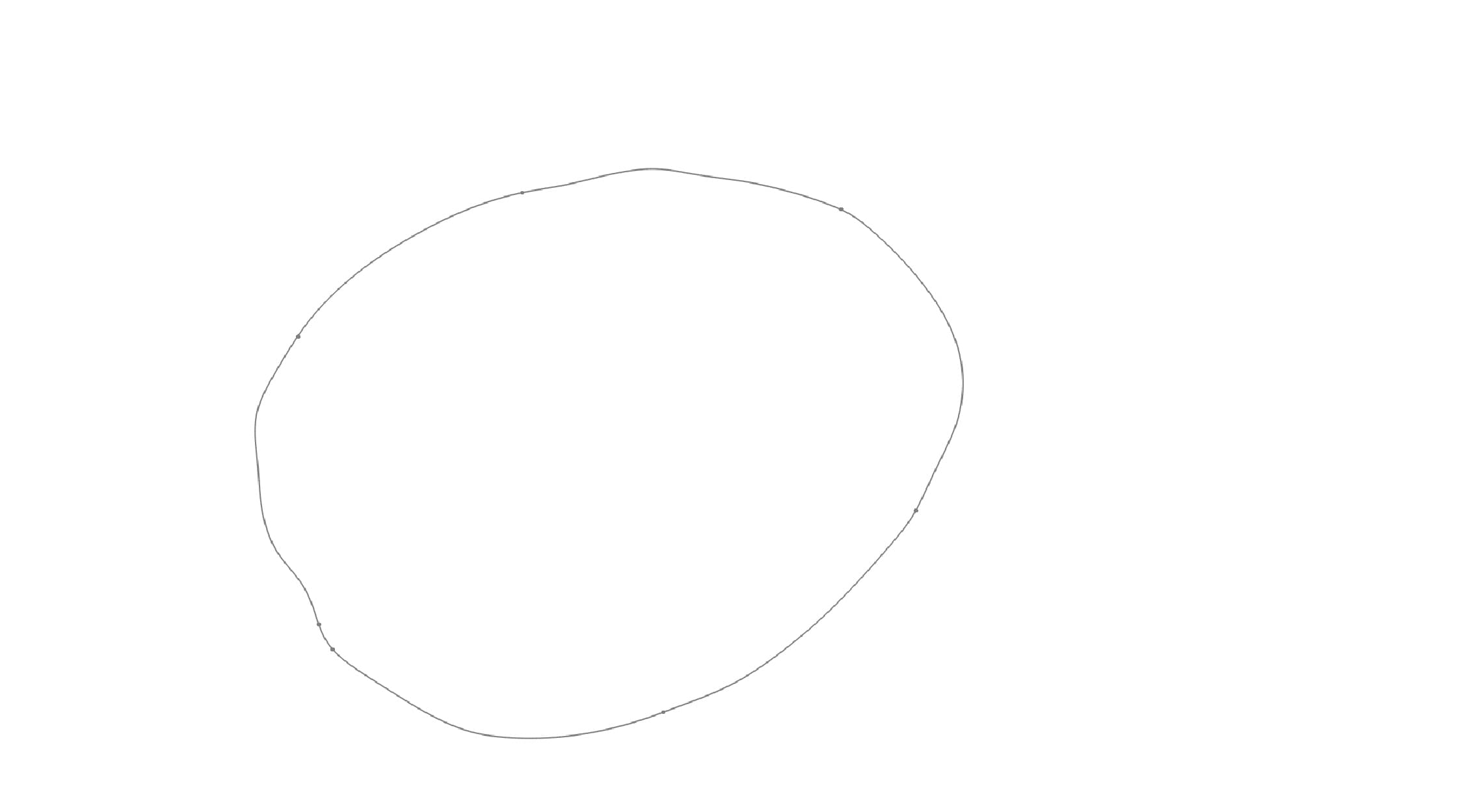

Supplement: Supplementary file 4 — Supporting Information [file ADVS-10-2203062-s013.zip › advs202203062-sup-0004-Supplementary-DataS3/Supplementary Data S3/81.jpg]

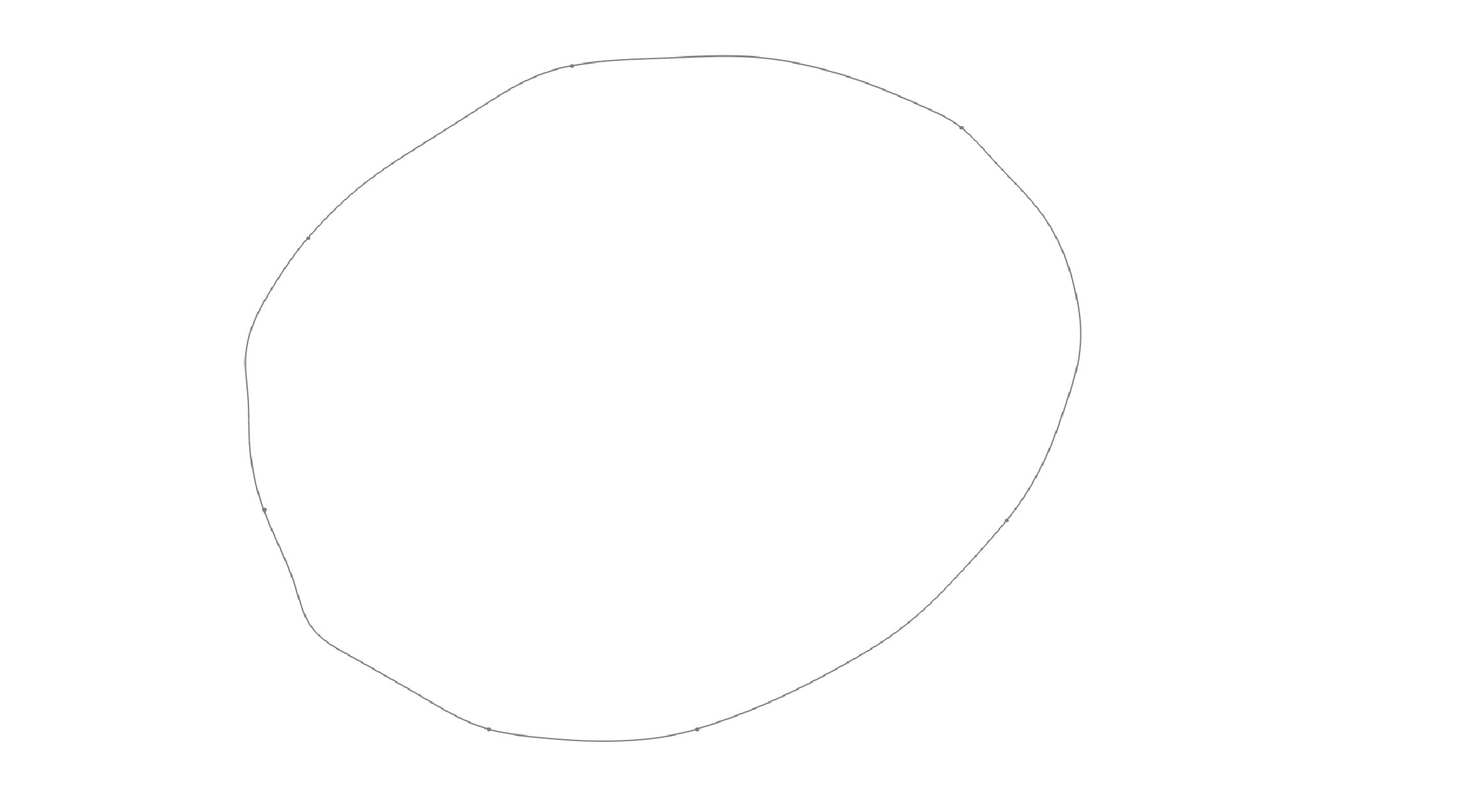

Supplement: Supplementary file 4 — Supporting Information [file ADVS-10-2203062-s013.zip › advs202203062-sup-0004-Supplementary-DataS3/Supplementary Data S3/82.jpg]

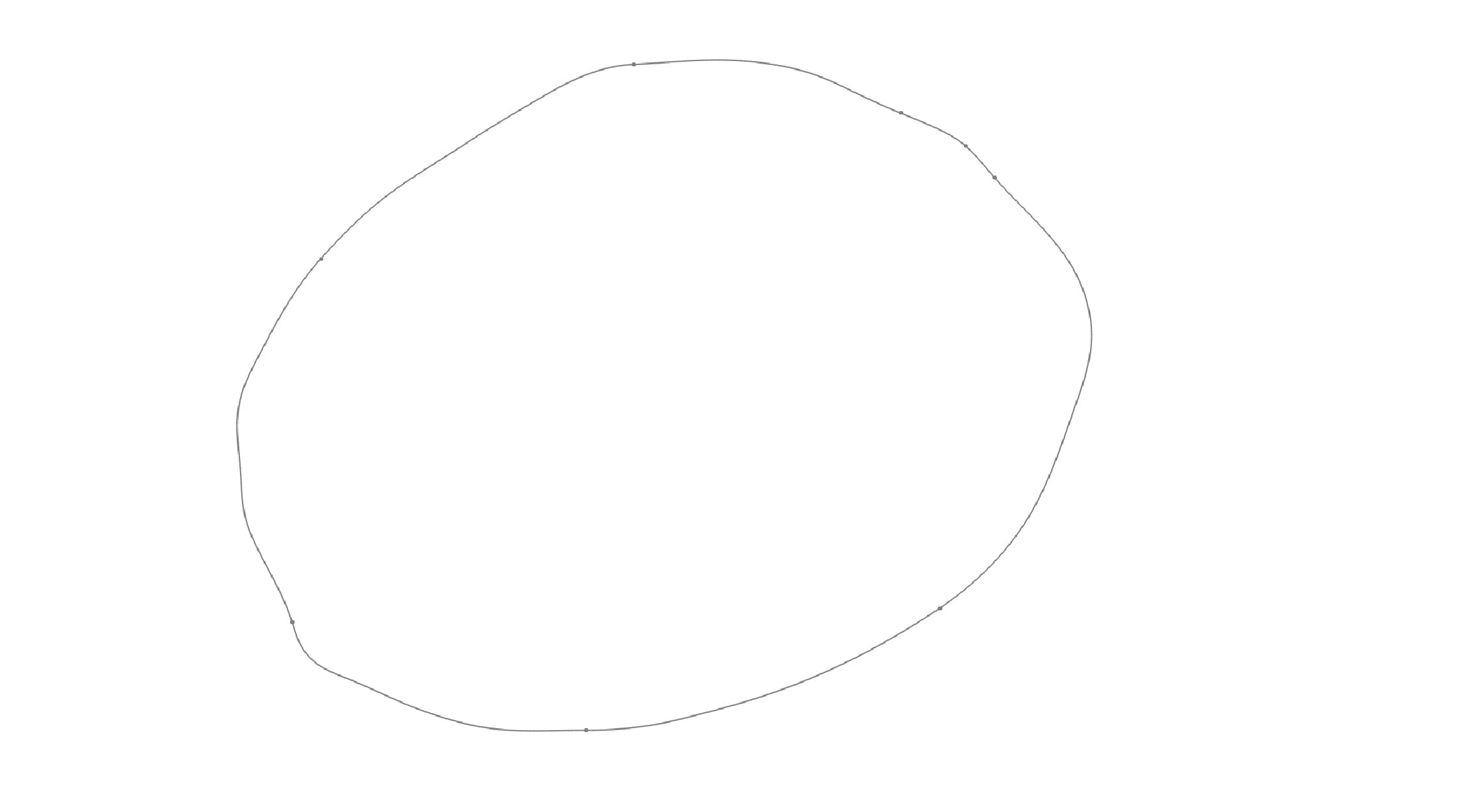

Supplement: Supplementary file 4 — Supporting Information [file ADVS-10-2203062-s013.zip › advs202203062-sup-0004-Supplementary-DataS3/Supplementary Data S3/83.jpg]

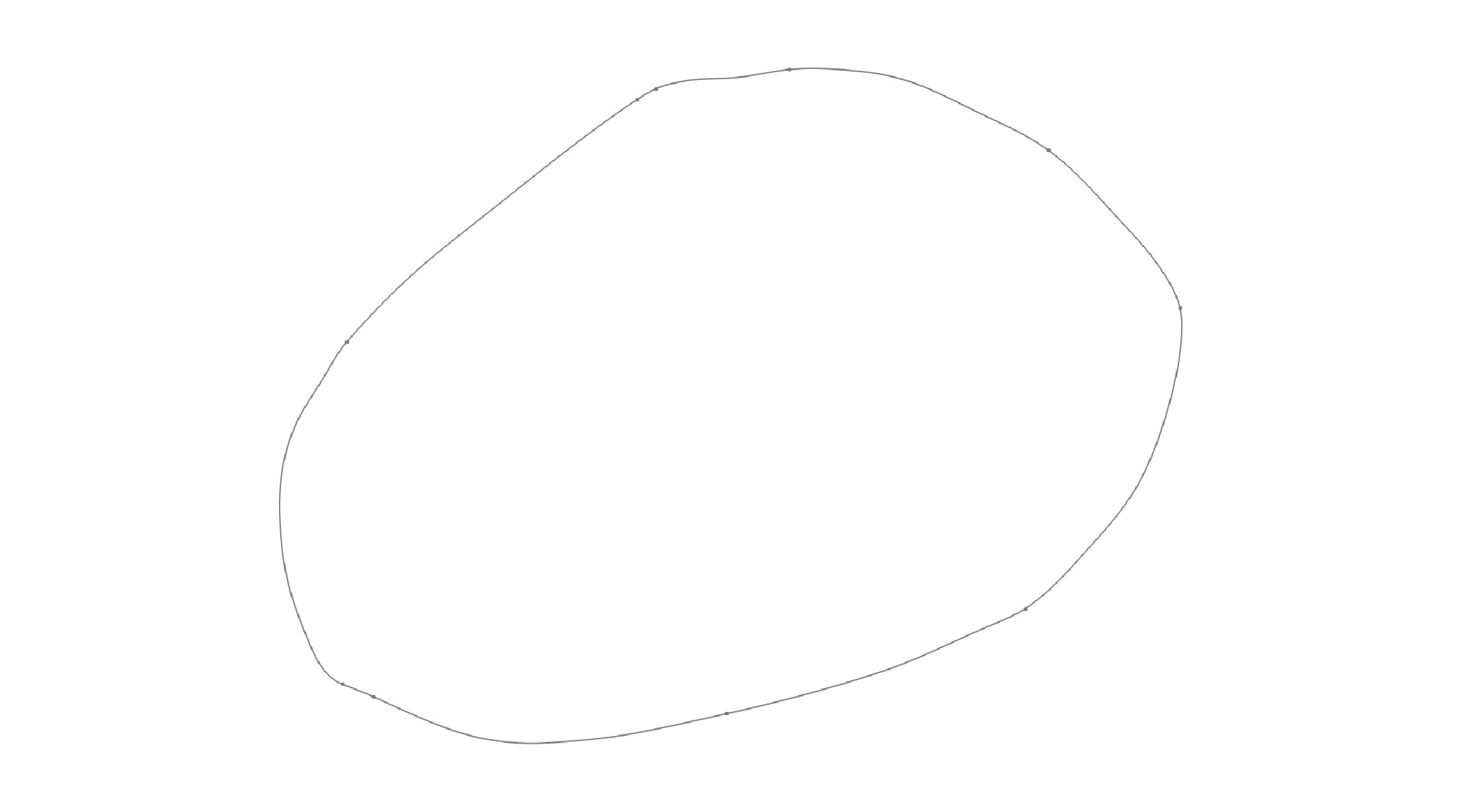

Supplement: Supplementary file 4 — Supporting Information [file ADVS-10-2203062-s013.zip › advs202203062-sup-0004-Supplementary-DataS3/Supplementary Data S3/84.jpg]

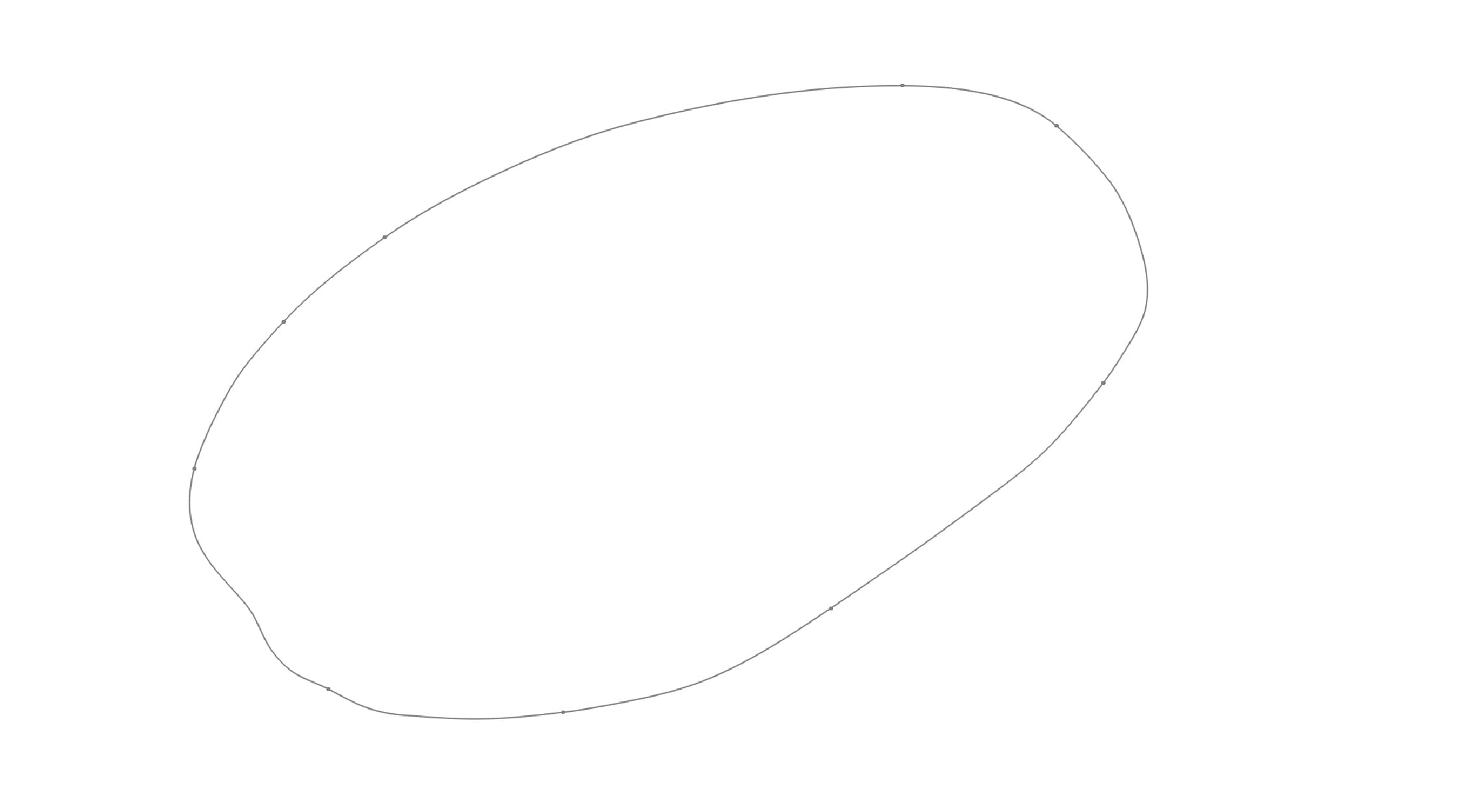

Supplement: Supplementary file 4 — Supporting Information [file ADVS-10-2203062-s013.zip › advs202203062-sup-0004-Supplementary-DataS3/Supplementary Data S3/85.jpg]

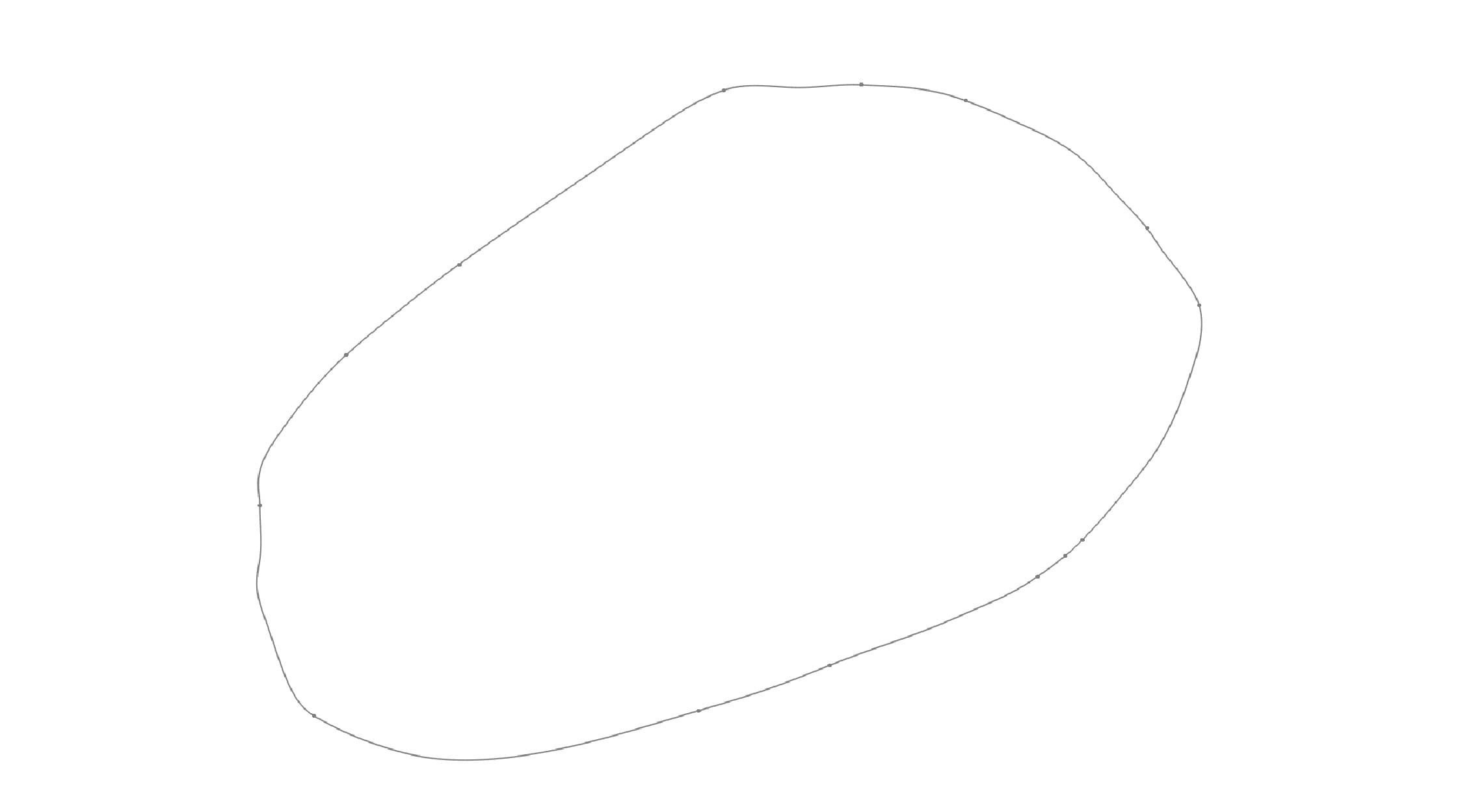

Supplement: Supplementary file 4 — Supporting Information [file ADVS-10-2203062-s013.zip › advs202203062-sup-0004-Supplementary-DataS3/Supplementary Data S3/86.jpg]

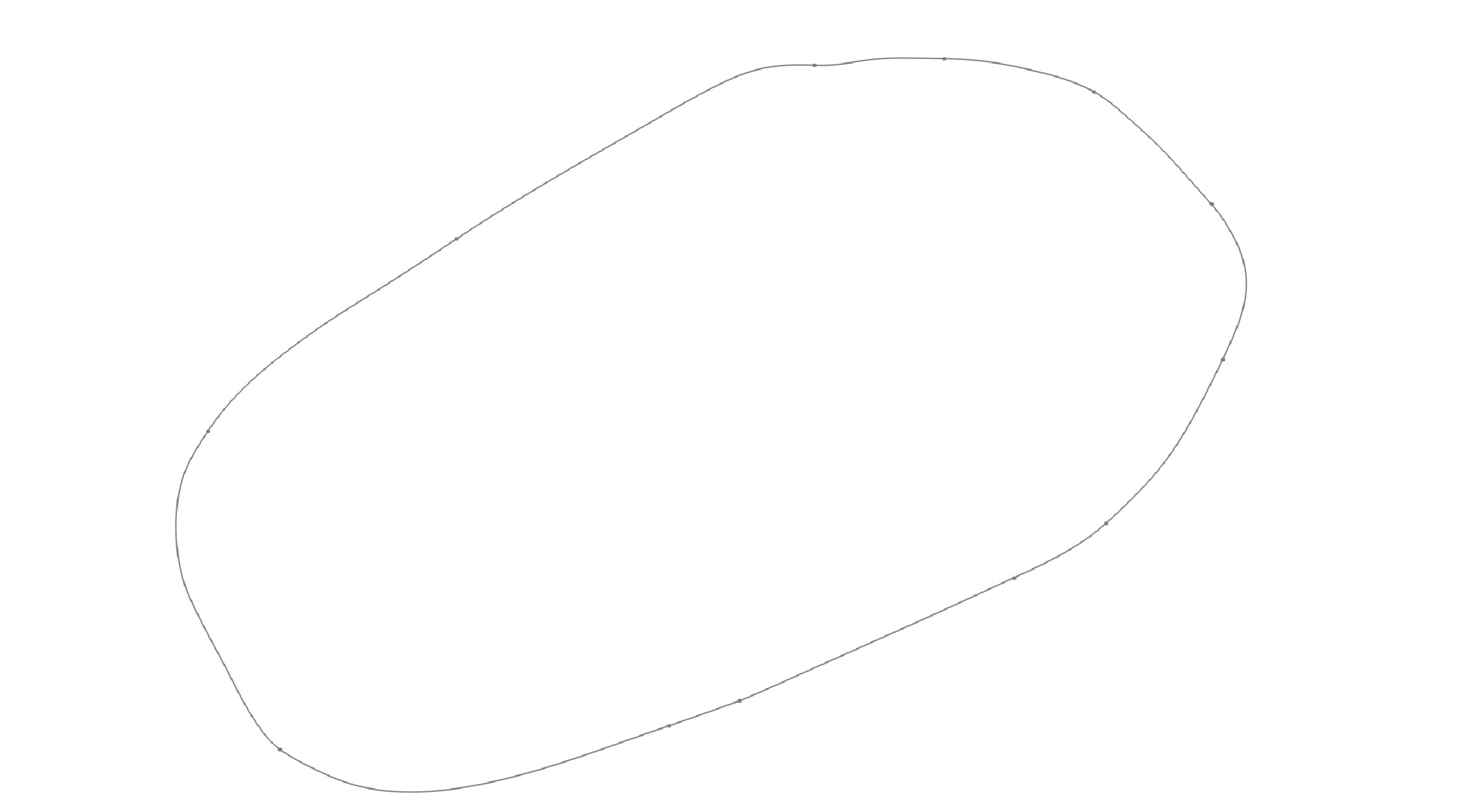

Supplement: Supplementary file 4 — Supporting Information [file ADVS-10-2203062-s013.zip › advs202203062-sup-0004-Supplementary-DataS3/Supplementary Data S3/87.jpg]

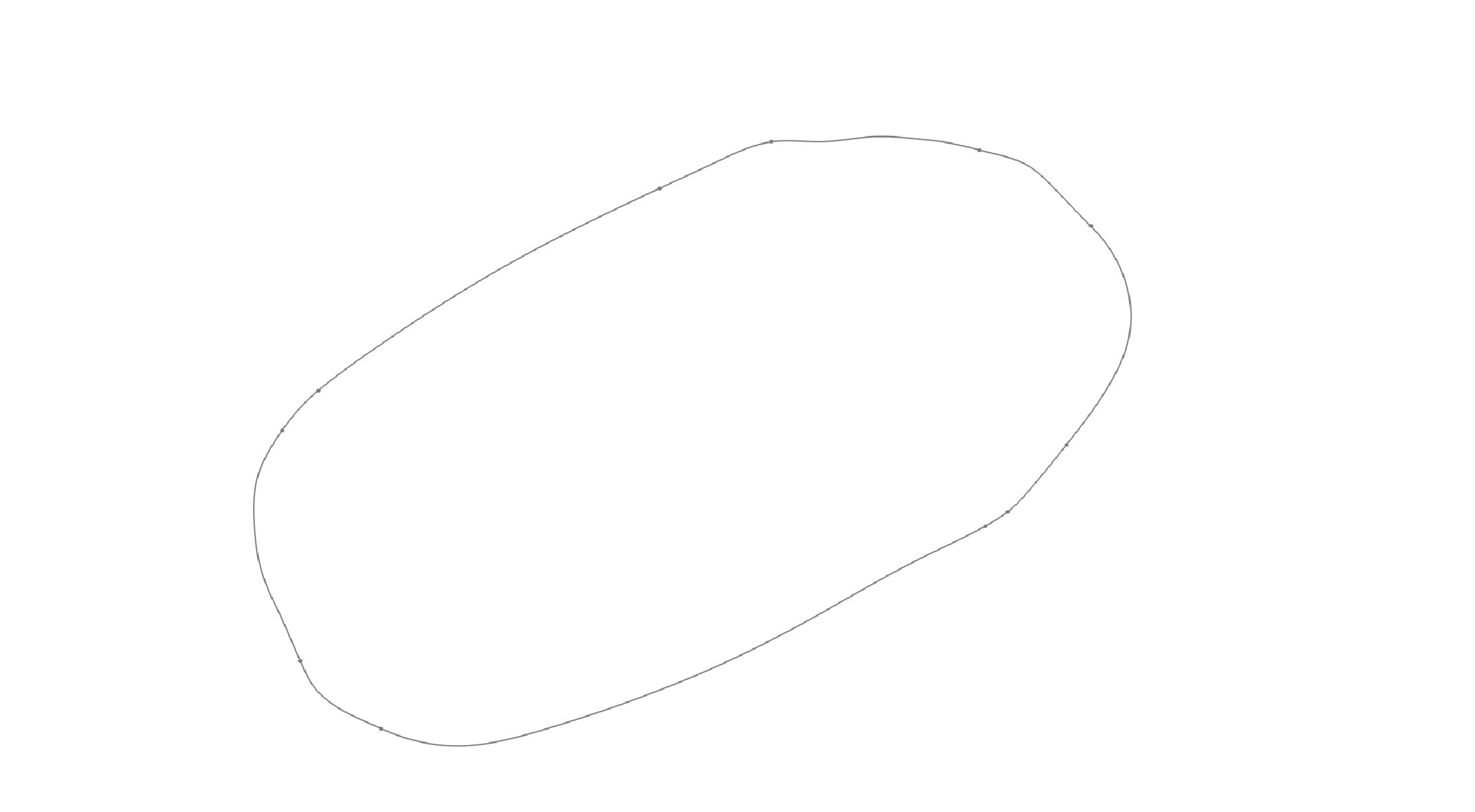

Supplement: Supplementary file 4 — Supporting Information [file ADVS-10-2203062-s013.zip › advs202203062-sup-0004-Supplementary-DataS3/Supplementary Data S3/88.jpg]

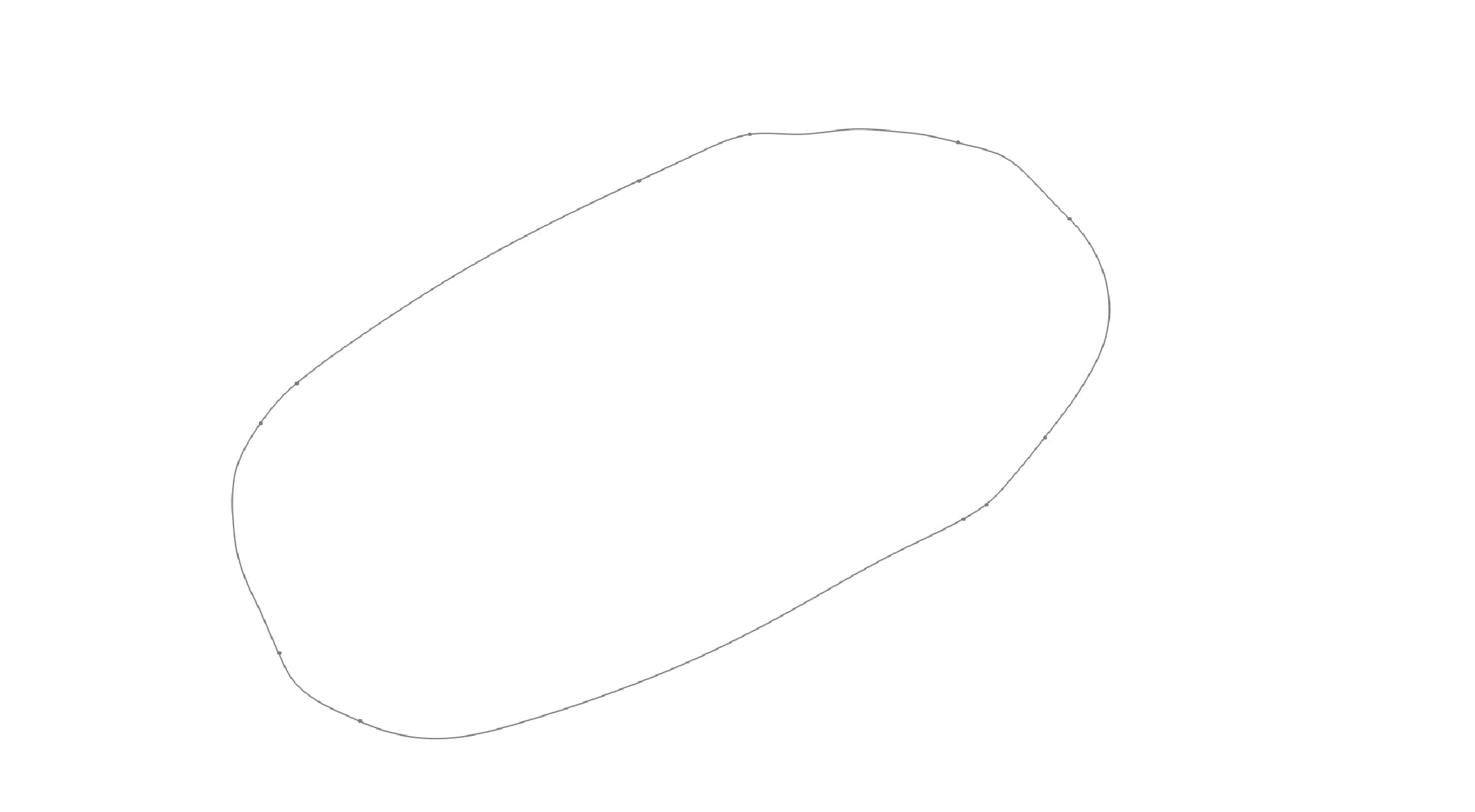

Supplement: Supplementary file 4 — Supporting Information [file ADVS-10-2203062-s013.zip › advs202203062-sup-0004-Supplementary-DataS3/Supplementary Data S3/89.jpg]

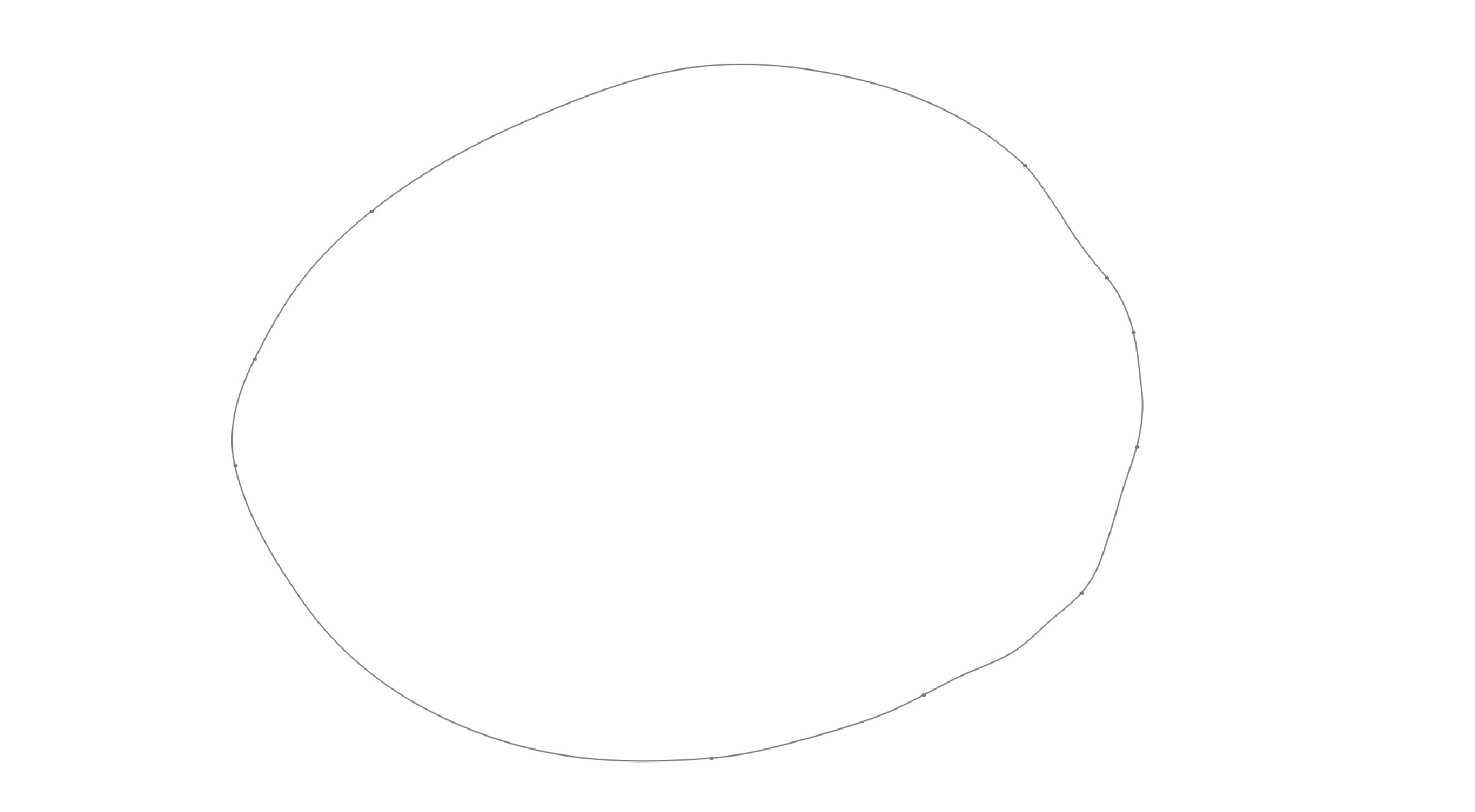

Supplement: Supplementary file 4 — Supporting Information [file ADVS-10-2203062-s013.zip › advs202203062-sup-0004-Supplementary-DataS3/Supplementary Data S3/9.jpg]

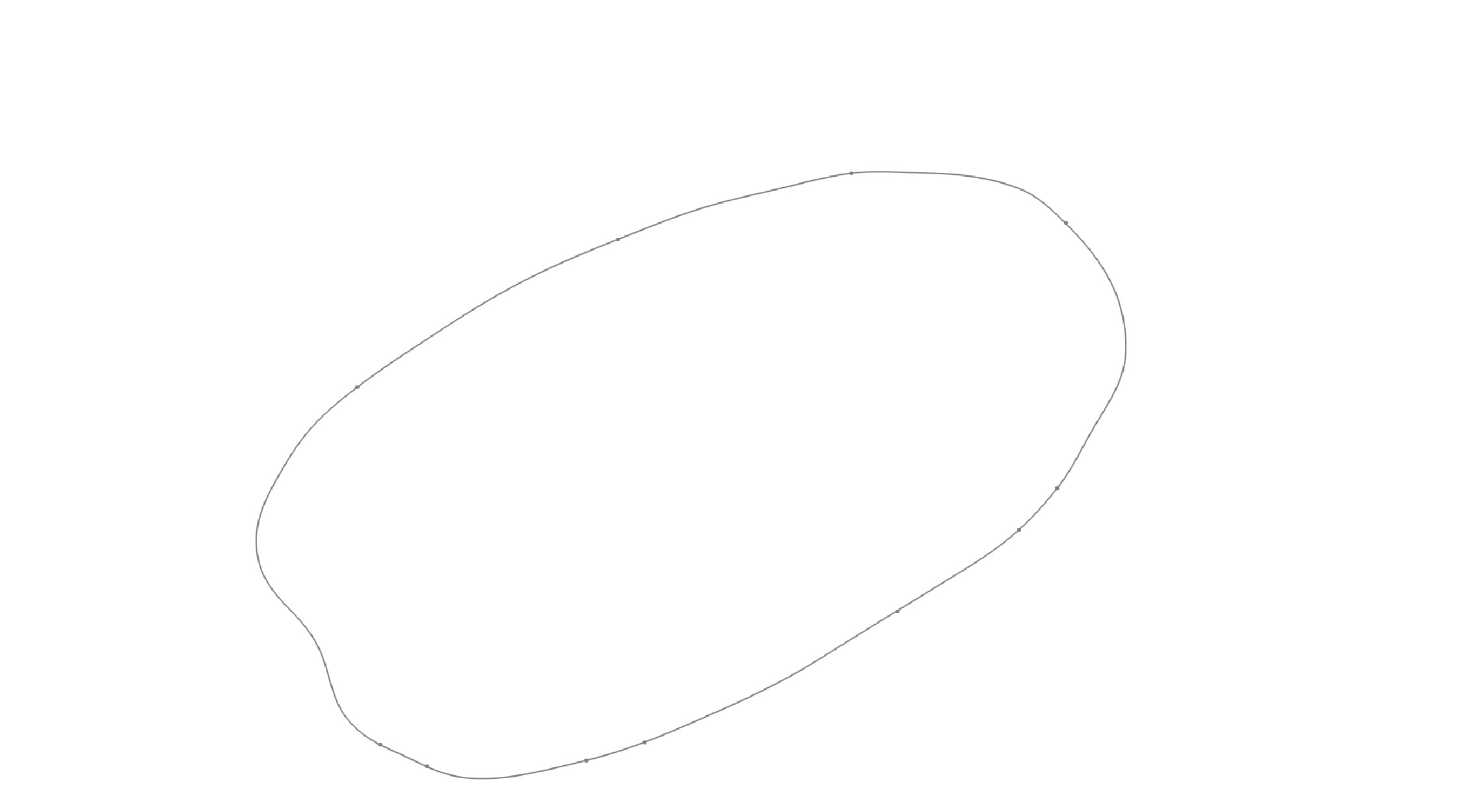

Supplement: Supplementary file 4 — Supporting Information [file ADVS-10-2203062-s013.zip › advs202203062-sup-0004-Supplementary-DataS3/Supplementary Data S3/90.jpg]

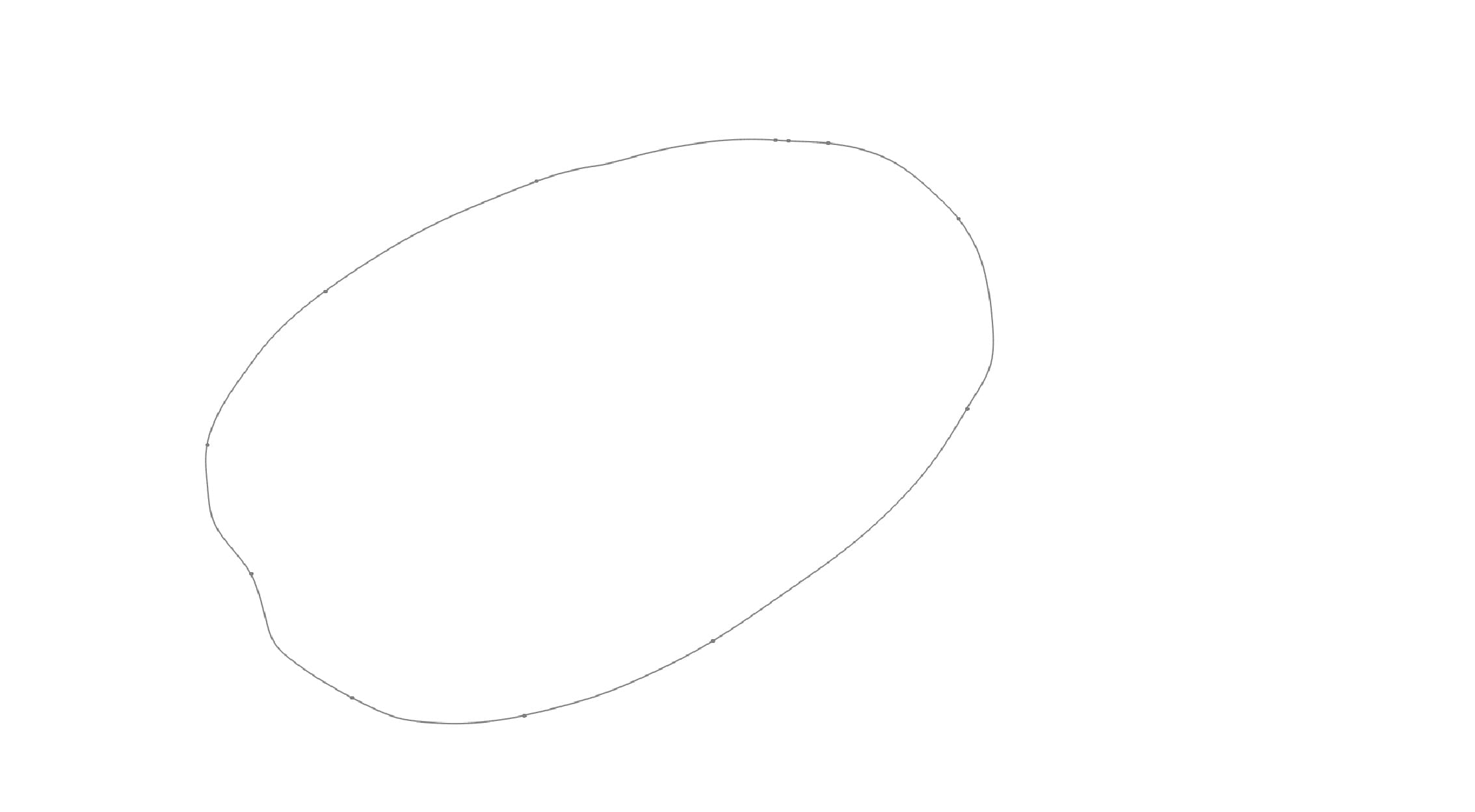

Supplement: Supplementary file 4 — Supporting Information [file ADVS-10-2203062-s013.zip › advs202203062-sup-0004-Supplementary-DataS3/Supplementary Data S3/91.jpg]

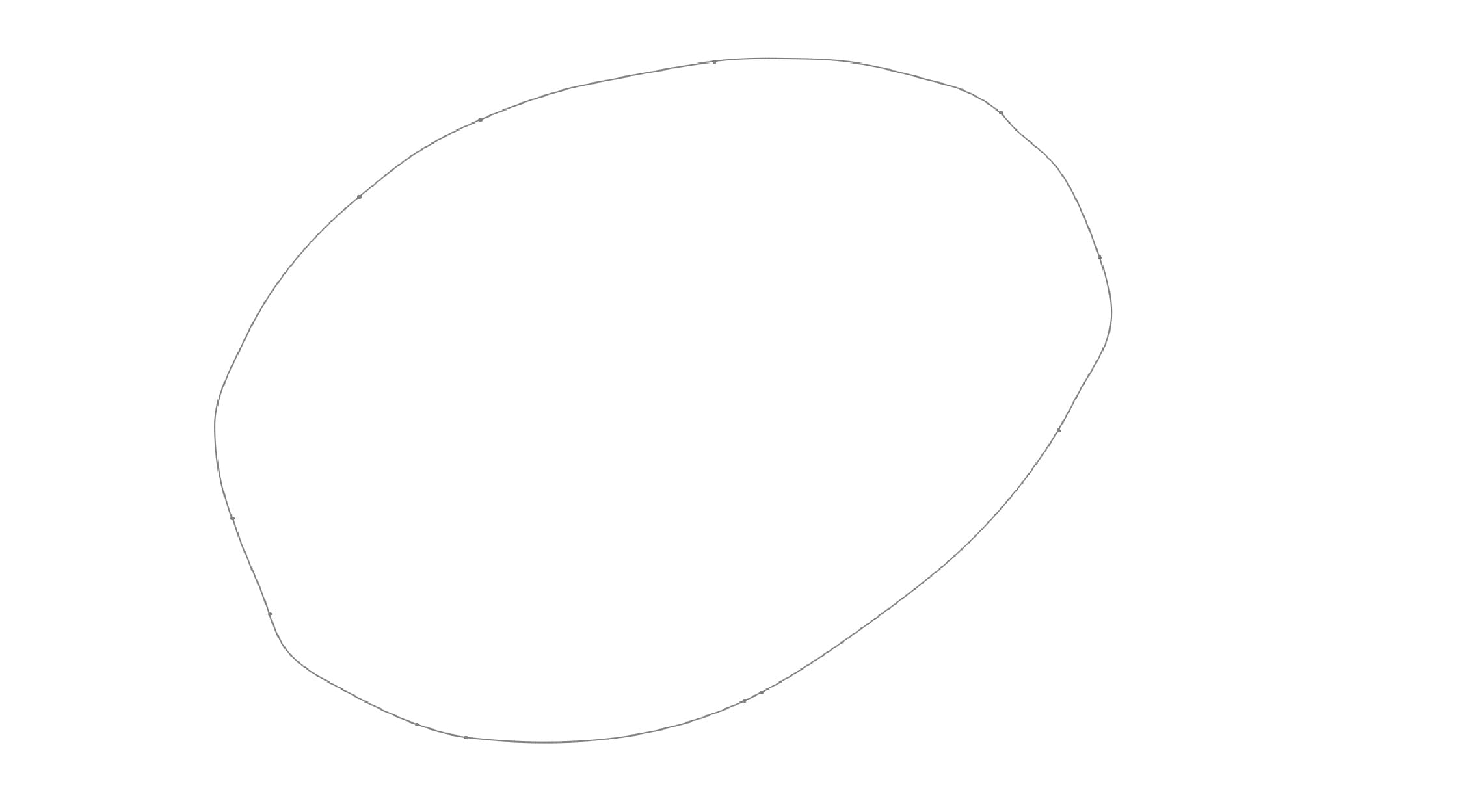

Supplement: Supplementary file 4 — Supporting Information [file ADVS-10-2203062-s013.zip › advs202203062-sup-0004-Supplementary-DataS3/Supplementary Data S3/92.jpg]

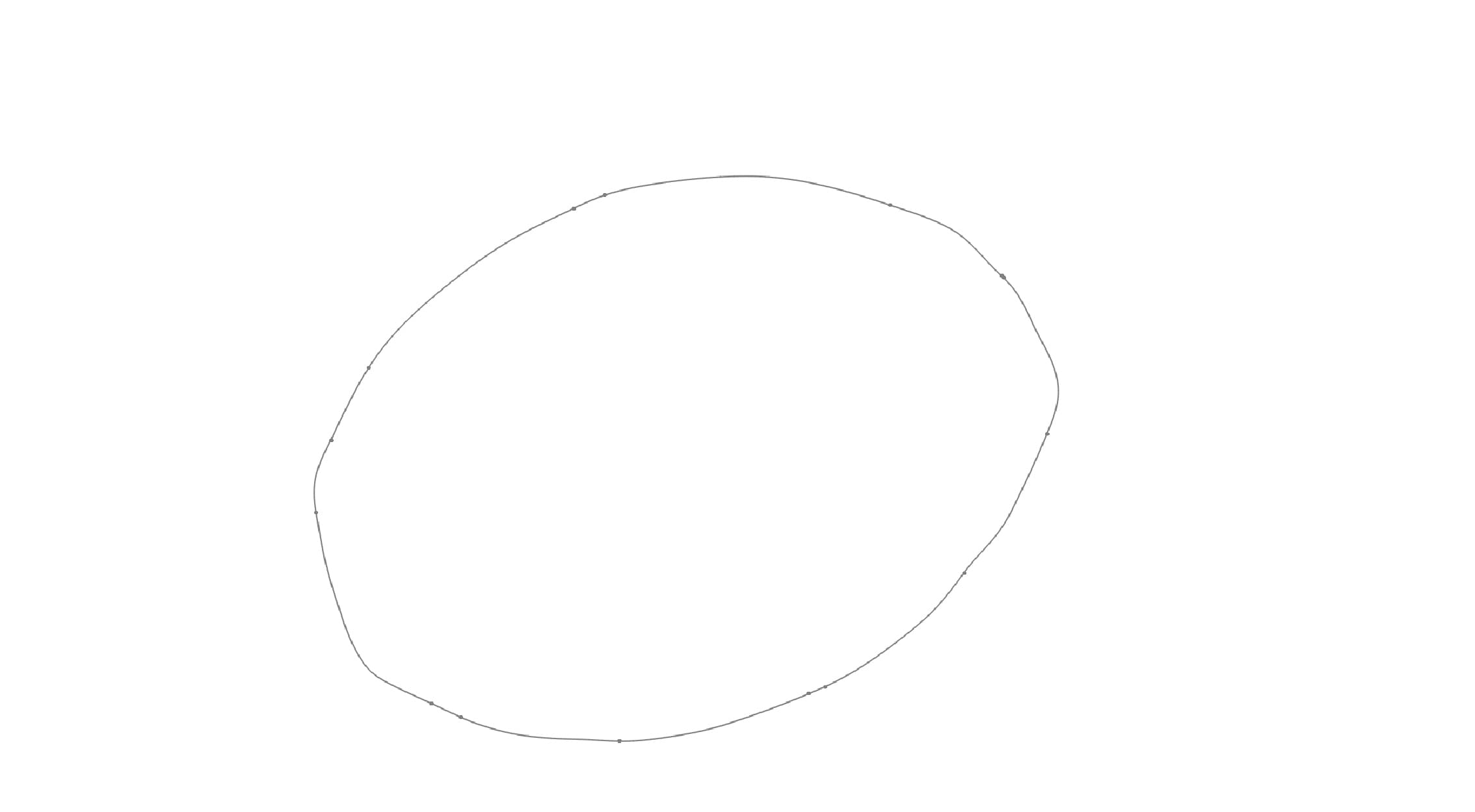

Supplement: Supplementary file 4 — Supporting Information [file ADVS-10-2203062-s013.zip › advs202203062-sup-0004-Supplementary-DataS3/Supplementary Data S3/93.jpg]

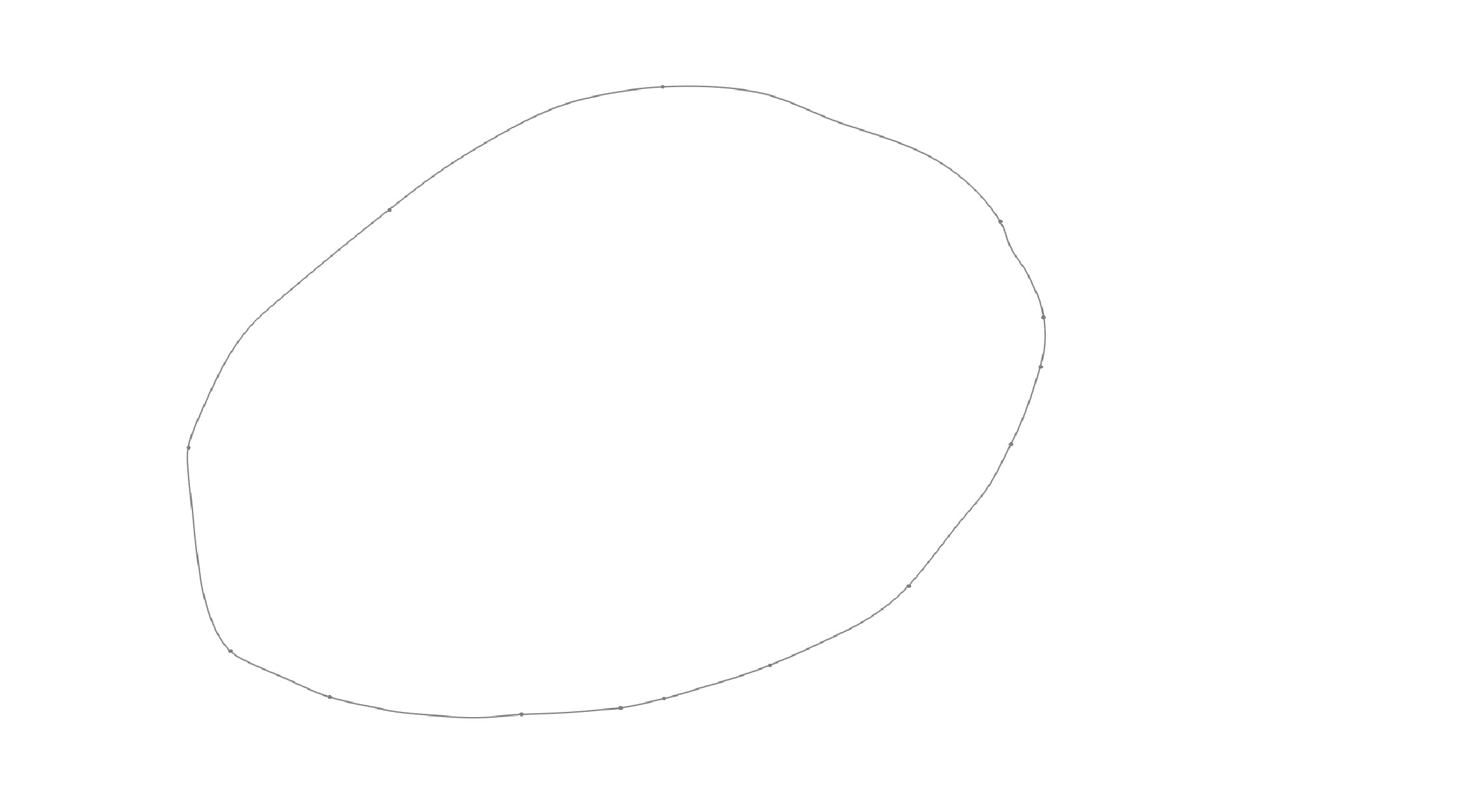

Supplement: Supplementary file 4 — Supporting Information [file ADVS-10-2203062-s013.zip › advs202203062-sup-0004-Supplementary-DataS3/Supplementary Data S3/94.jpg]

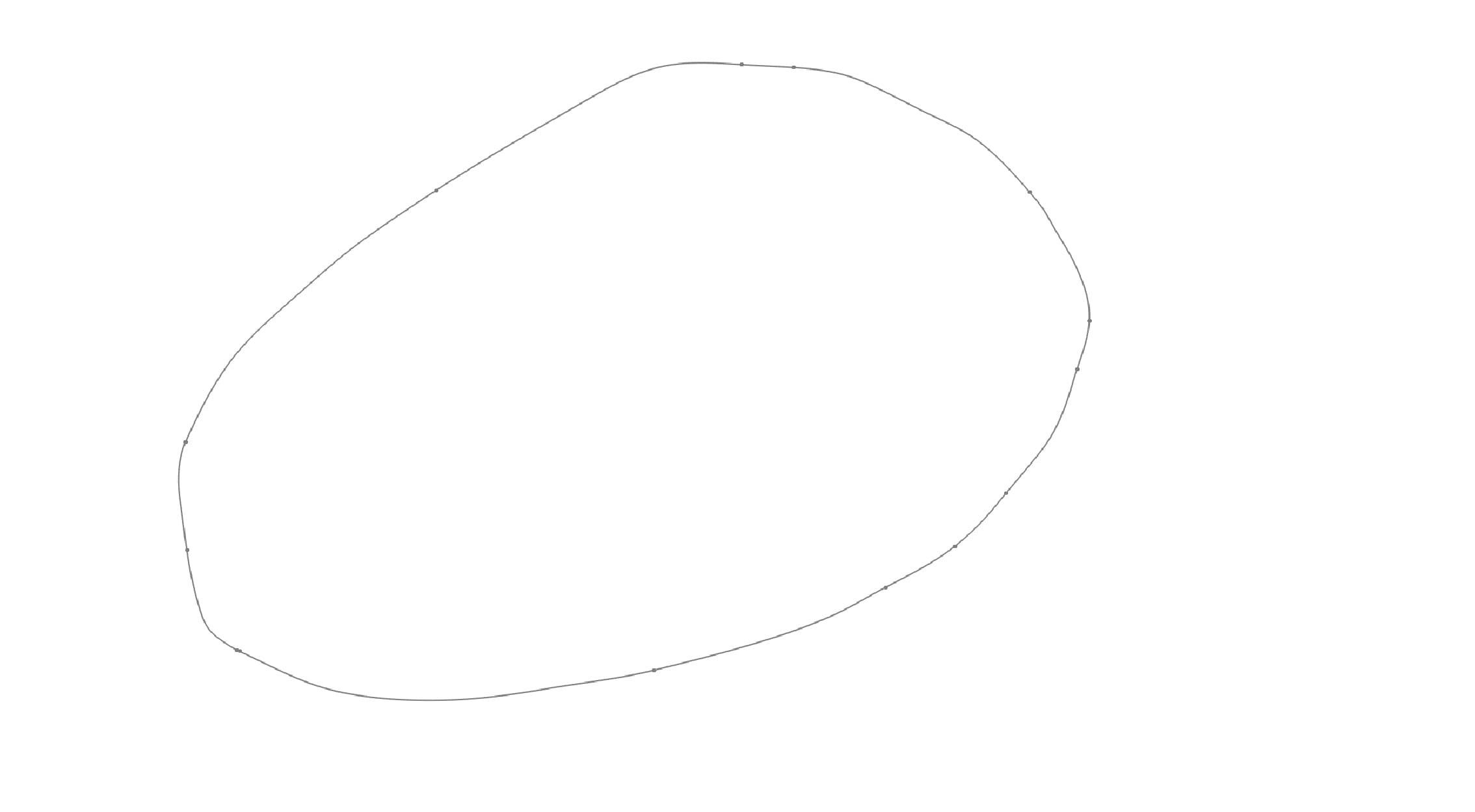

Supplement: Supplementary file 4 — Supporting Information [file ADVS-10-2203062-s013.zip › advs202203062-sup-0004-Supplementary-DataS3/Supplementary Data S3/95.jpg]

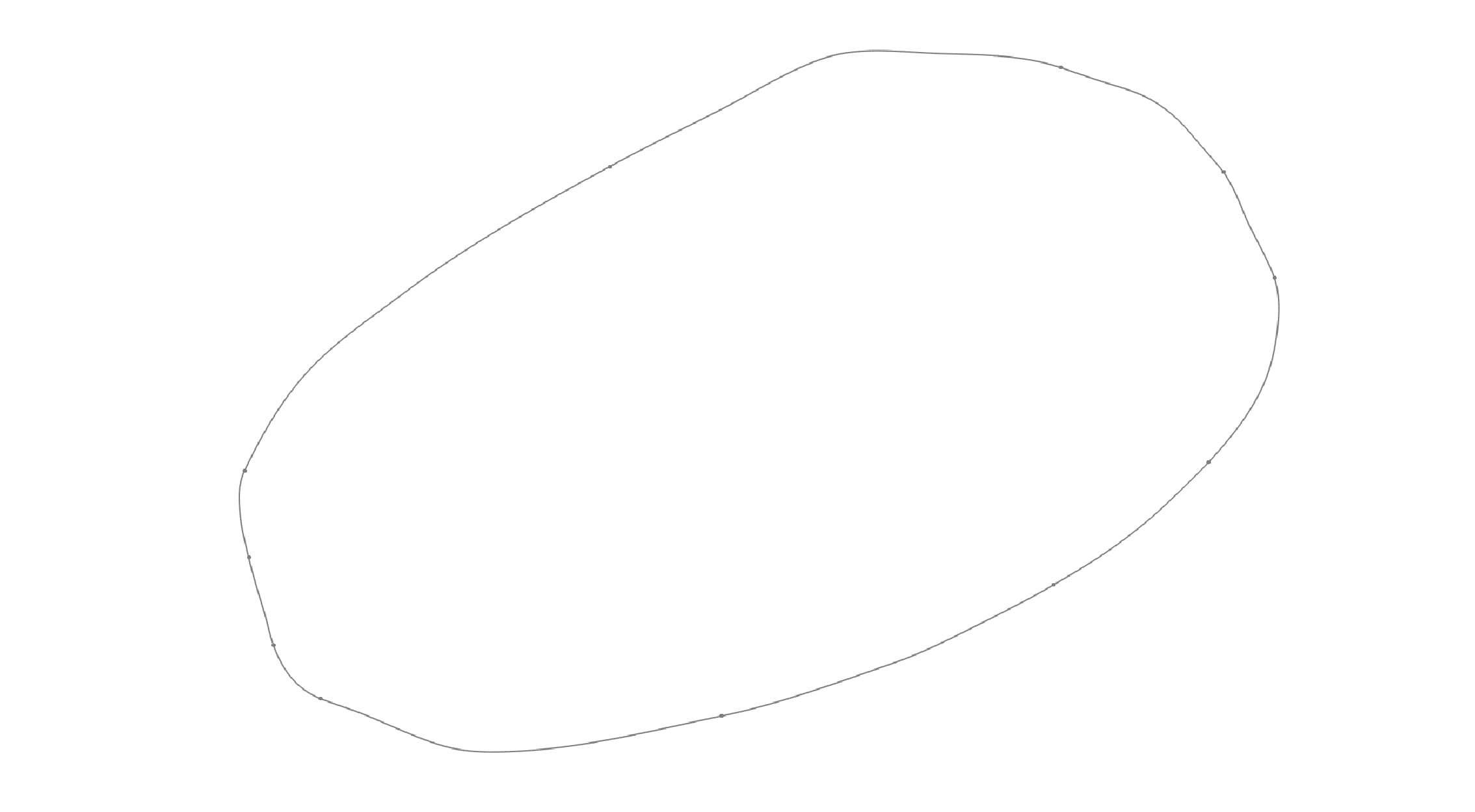

Supplement: Supplementary file 4 — Supporting Information [file ADVS-10-2203062-s013.zip › advs202203062-sup-0004-Supplementary-DataS3/Supplementary Data S3/96.jpg]

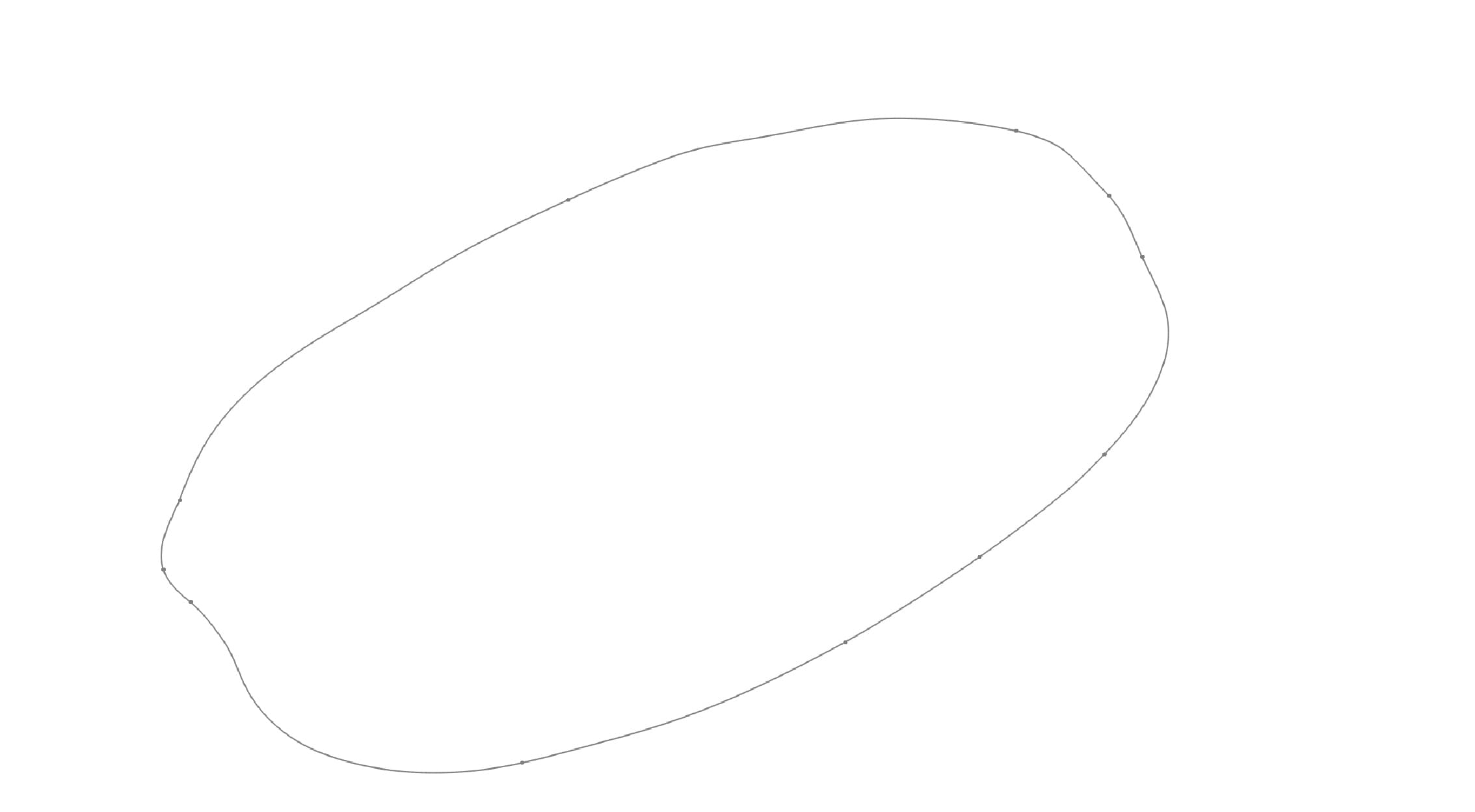

Supplement: Supplementary file 4 — Supporting Information [file ADVS-10-2203062-s013.zip › advs202203062-sup-0004-Supplementary-DataS3/Supplementary Data S3/97.jpg]

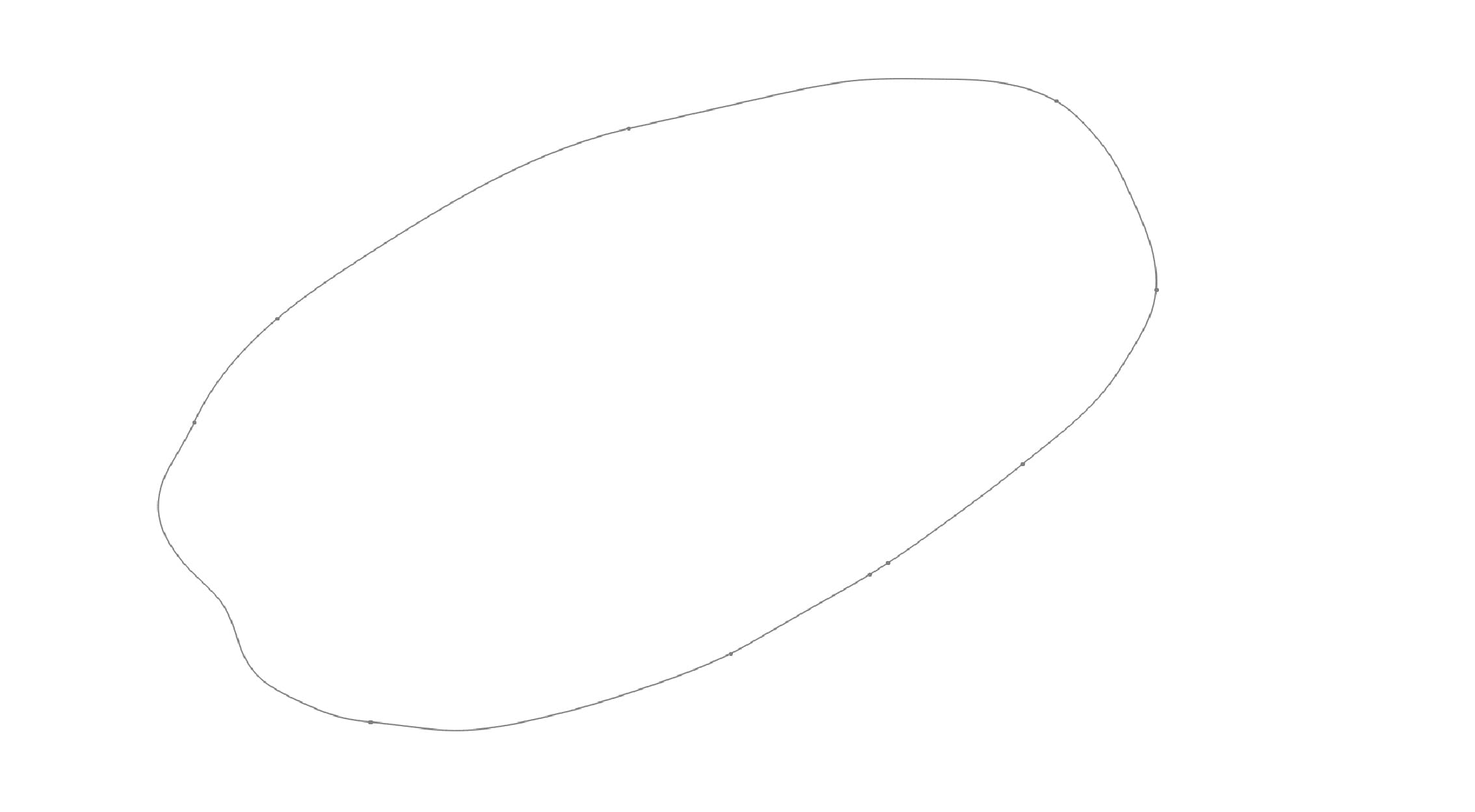

Supplement: Supplementary file 4 — Supporting Information [file ADVS-10-2203062-s013.zip › advs202203062-sup-0004-Supplementary-DataS3/Supplementary Data S3/98.jpg]

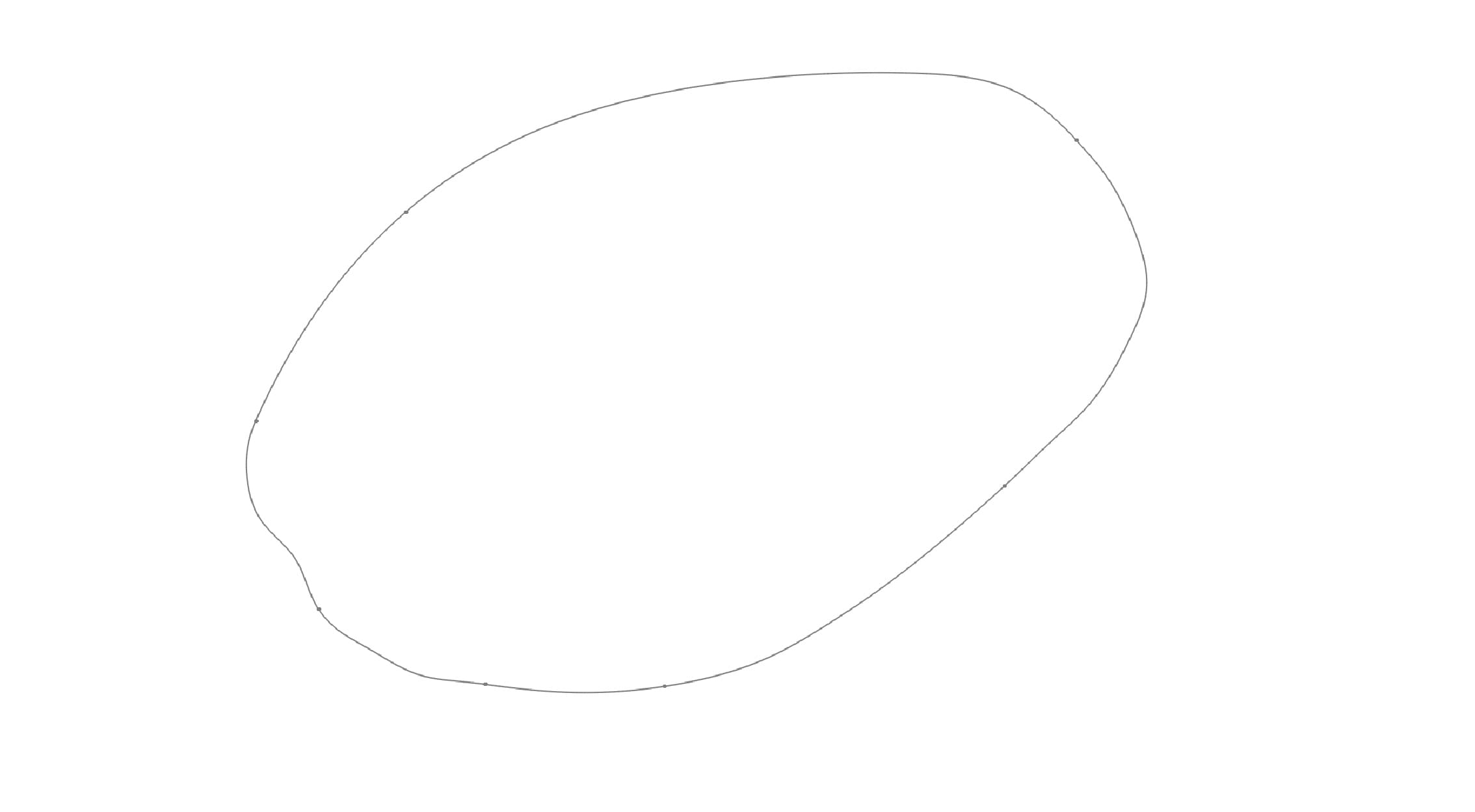

Supplement: Supplementary file 4 — Supporting Information [file ADVS-10-2203062-s013.zip › advs202203062-sup-0004-Supplementary-DataS3/Supplementary Data S3/99.jpg]
