# Supplementary figures and images for: Wavy Whiskers in Wakes: Explaining the Trail‐Tracking Capabilities of Whisker Arrays on Seal Muzzles (part 1 of 2)
Source: Adv Sci (Weinh). 2022 Nov 20;10(2):2203062. doi: 10.1002/advs.202203062 (PMC9839859; doi:10.1002/advs.202203062)

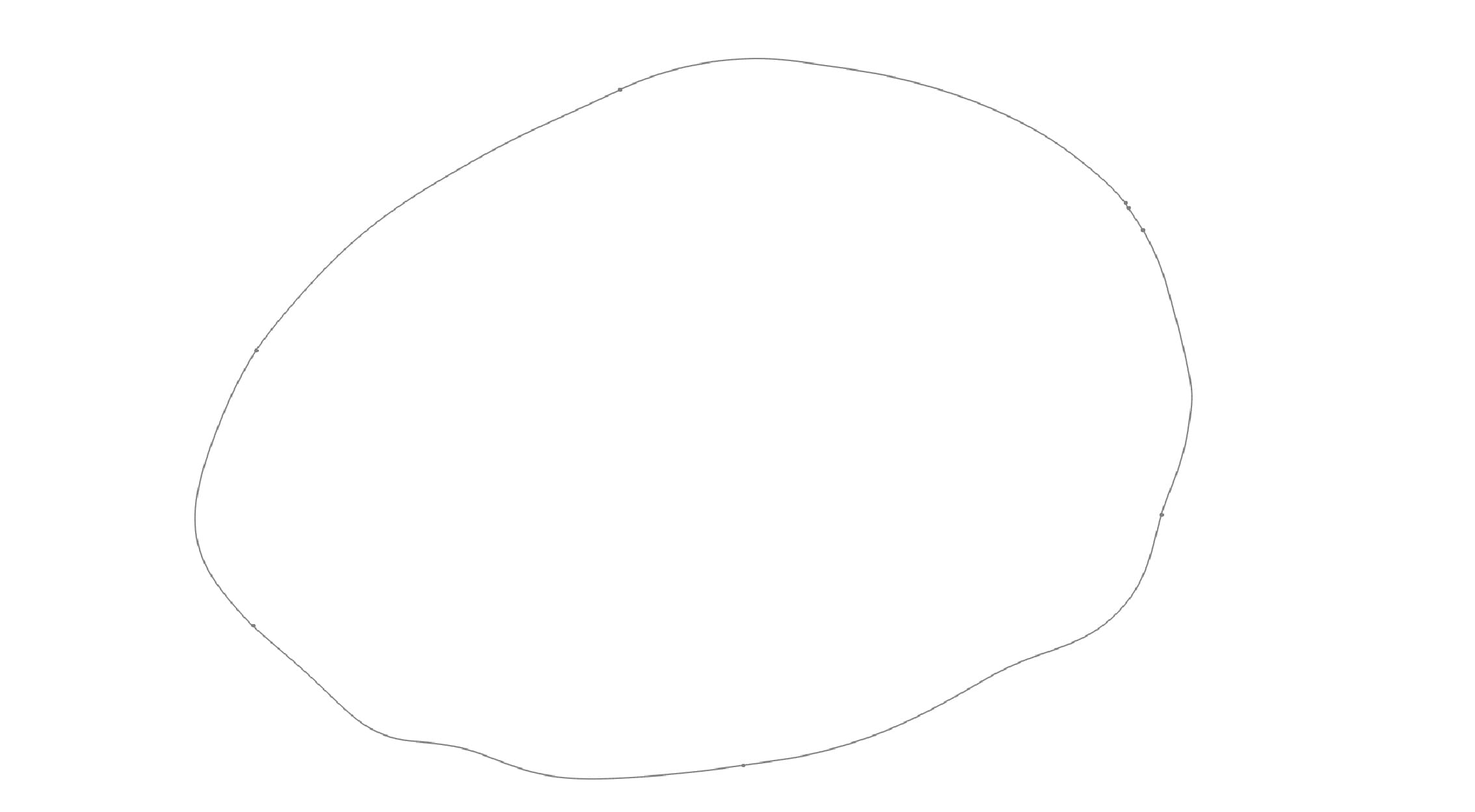

Supplement: Supplementary file 4 — Supporting Information [file ADVS-10-2203062-s013.zip › advs202203062-sup-0004-Supplementary-DataS3/Supplementary Data S3/1.jpg]

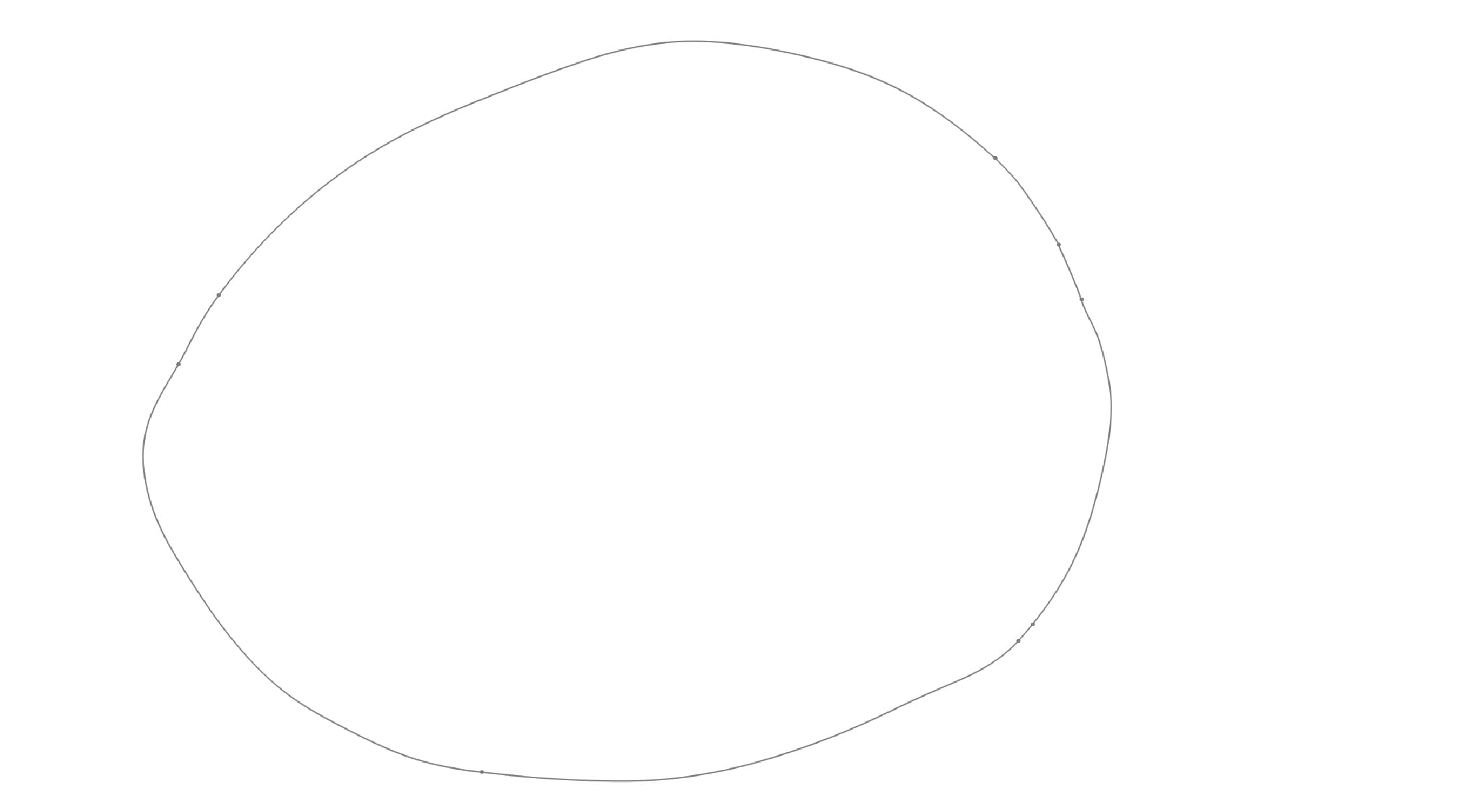

Supplement: Supplementary file 4 — Supporting Information [file ADVS-10-2203062-s013.zip › advs202203062-sup-0004-Supplementary-DataS3/Supplementary Data S3/10.jpg]

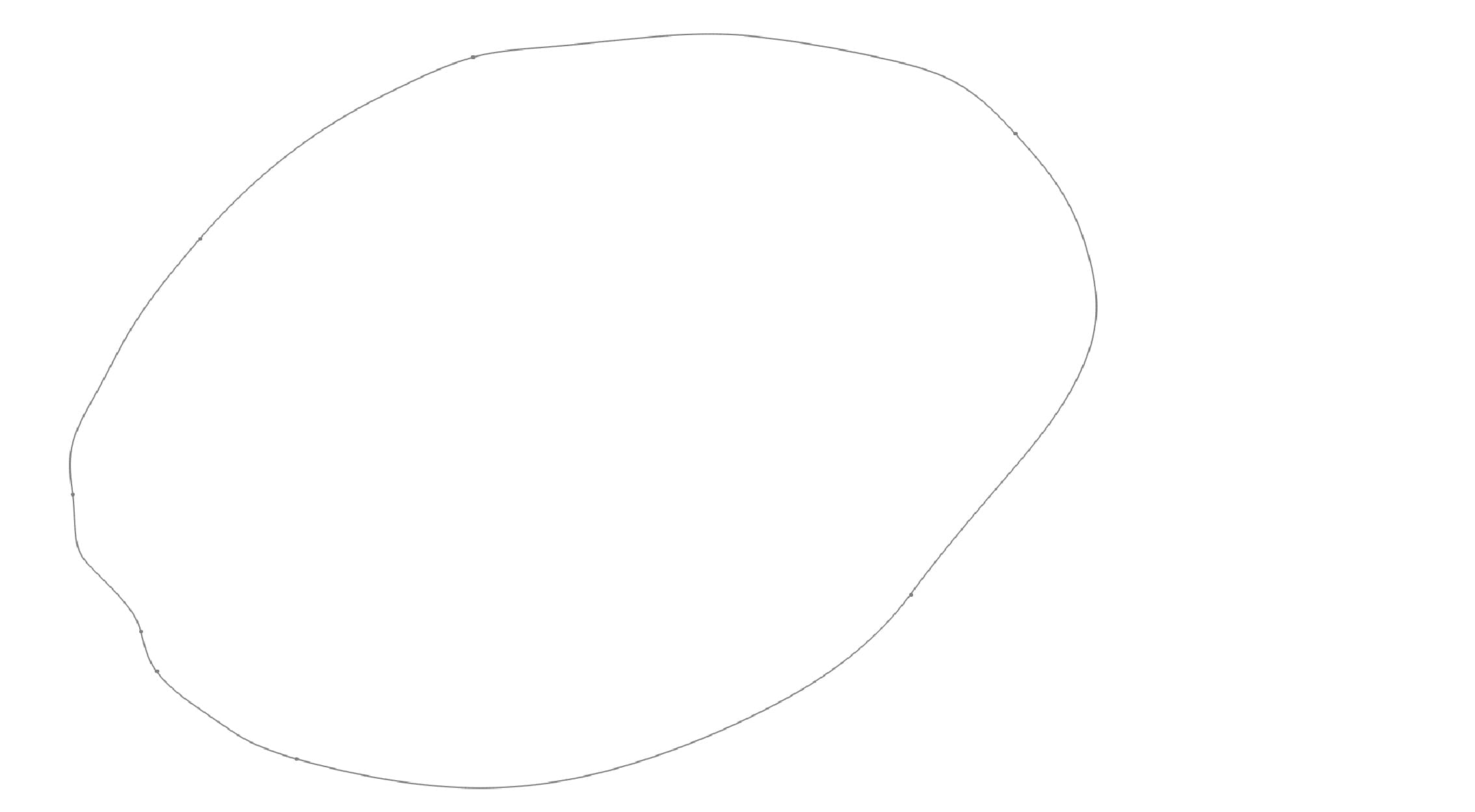

Supplement: Supplementary file 4 — Supporting Information [file ADVS-10-2203062-s013.zip › advs202203062-sup-0004-Supplementary-DataS3/Supplementary Data S3/100.jpg]

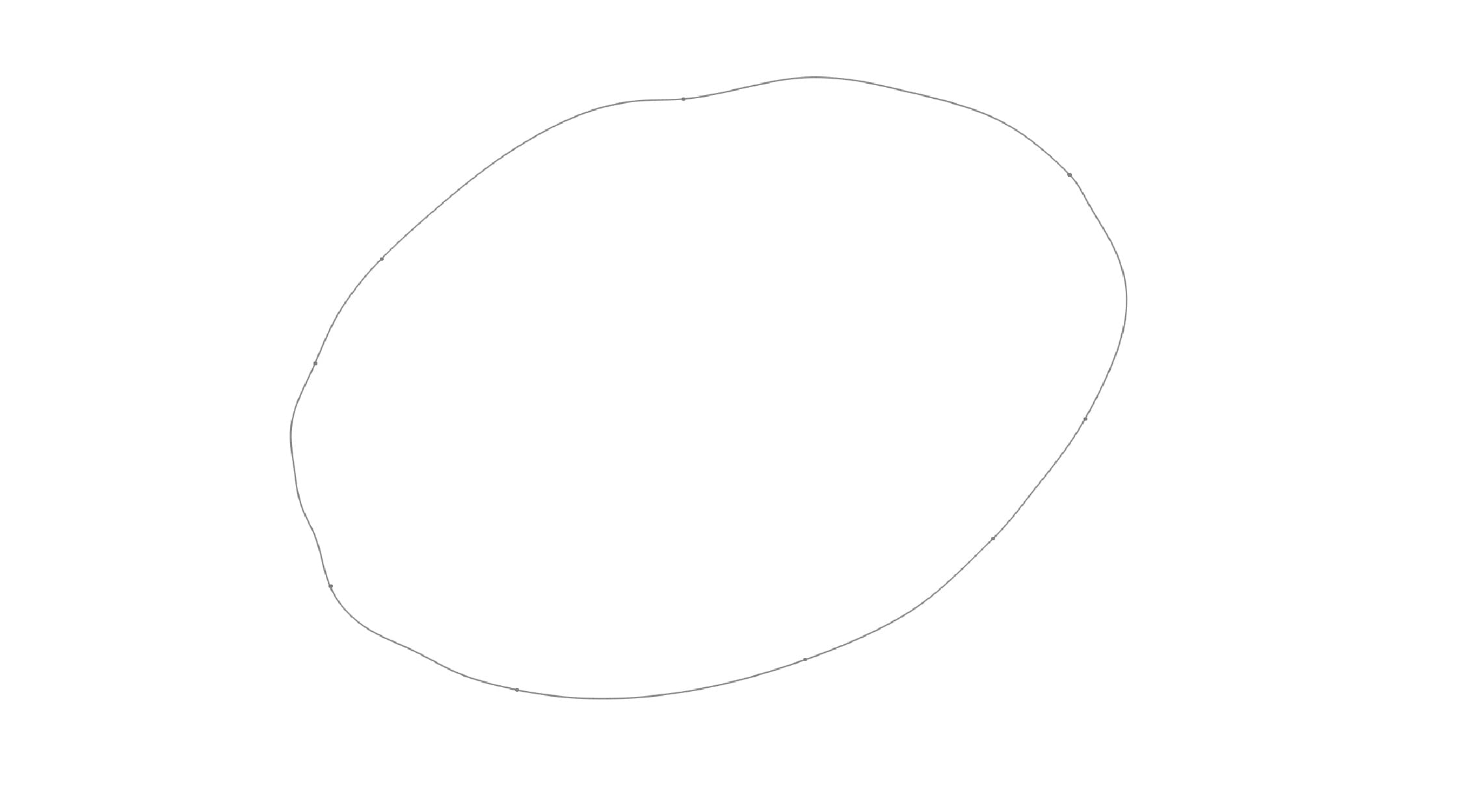

Supplement: Supplementary file 4 — Supporting Information [file ADVS-10-2203062-s013.zip › advs202203062-sup-0004-Supplementary-DataS3/Supplementary Data S3/101.jpg]

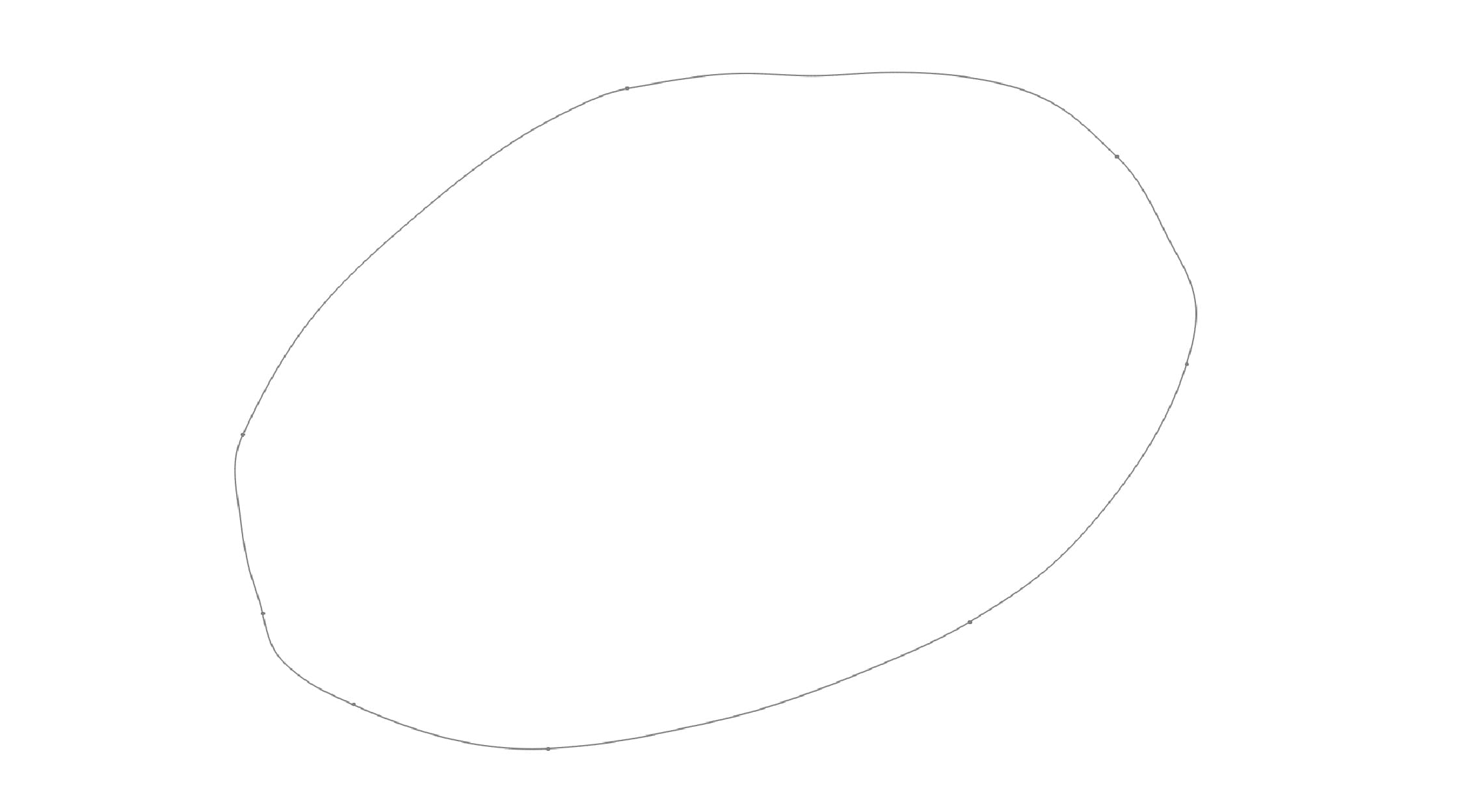

Supplement: Supplementary file 4 — Supporting Information [file ADVS-10-2203062-s013.zip › advs202203062-sup-0004-Supplementary-DataS3/Supplementary Data S3/102.jpg]

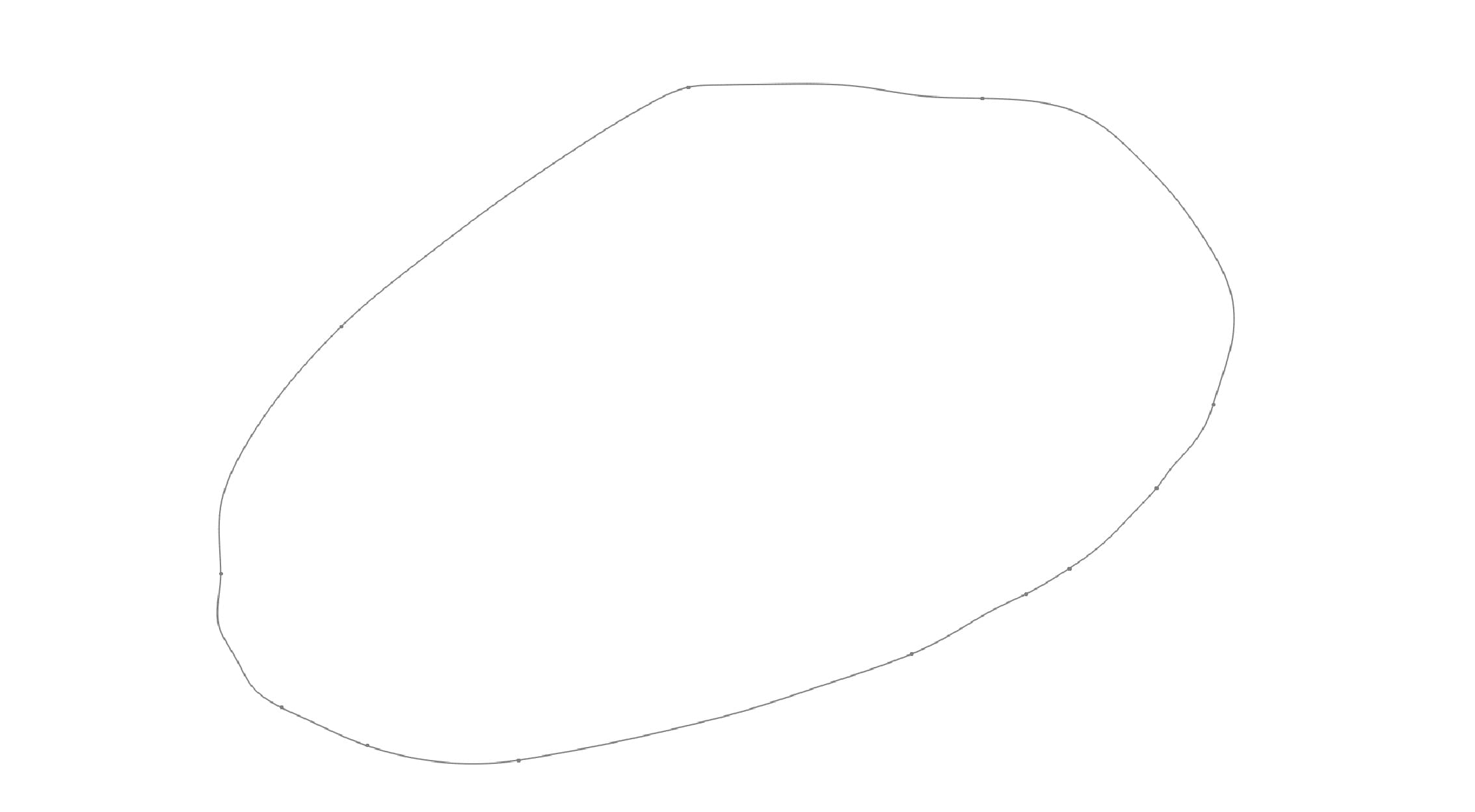

Supplement: Supplementary file 4 — Supporting Information [file ADVS-10-2203062-s013.zip › advs202203062-sup-0004-Supplementary-DataS3/Supplementary Data S3/103.jpg]

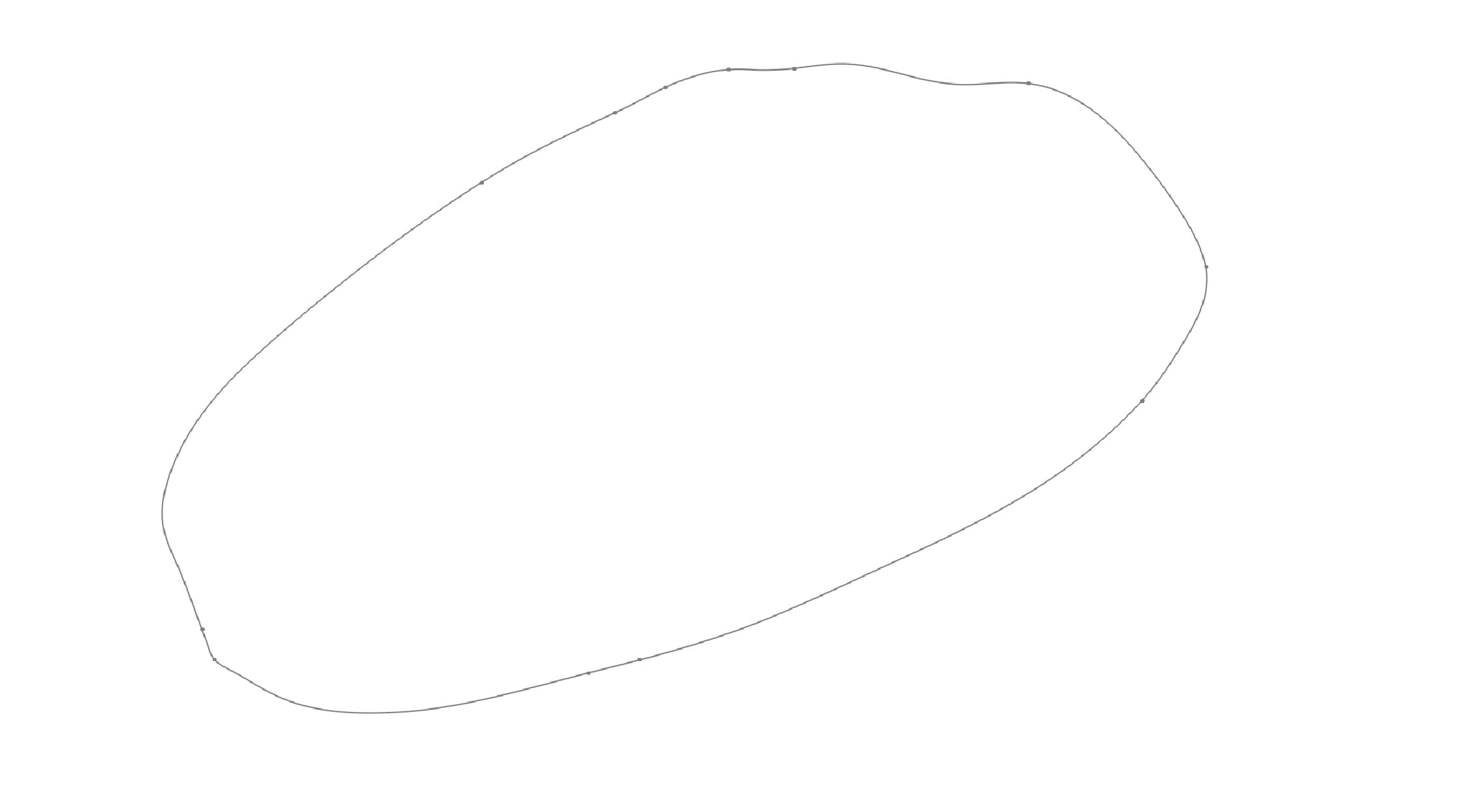

Supplement: Supplementary file 4 — Supporting Information [file ADVS-10-2203062-s013.zip › advs202203062-sup-0004-Supplementary-DataS3/Supplementary Data S3/104.jpg]

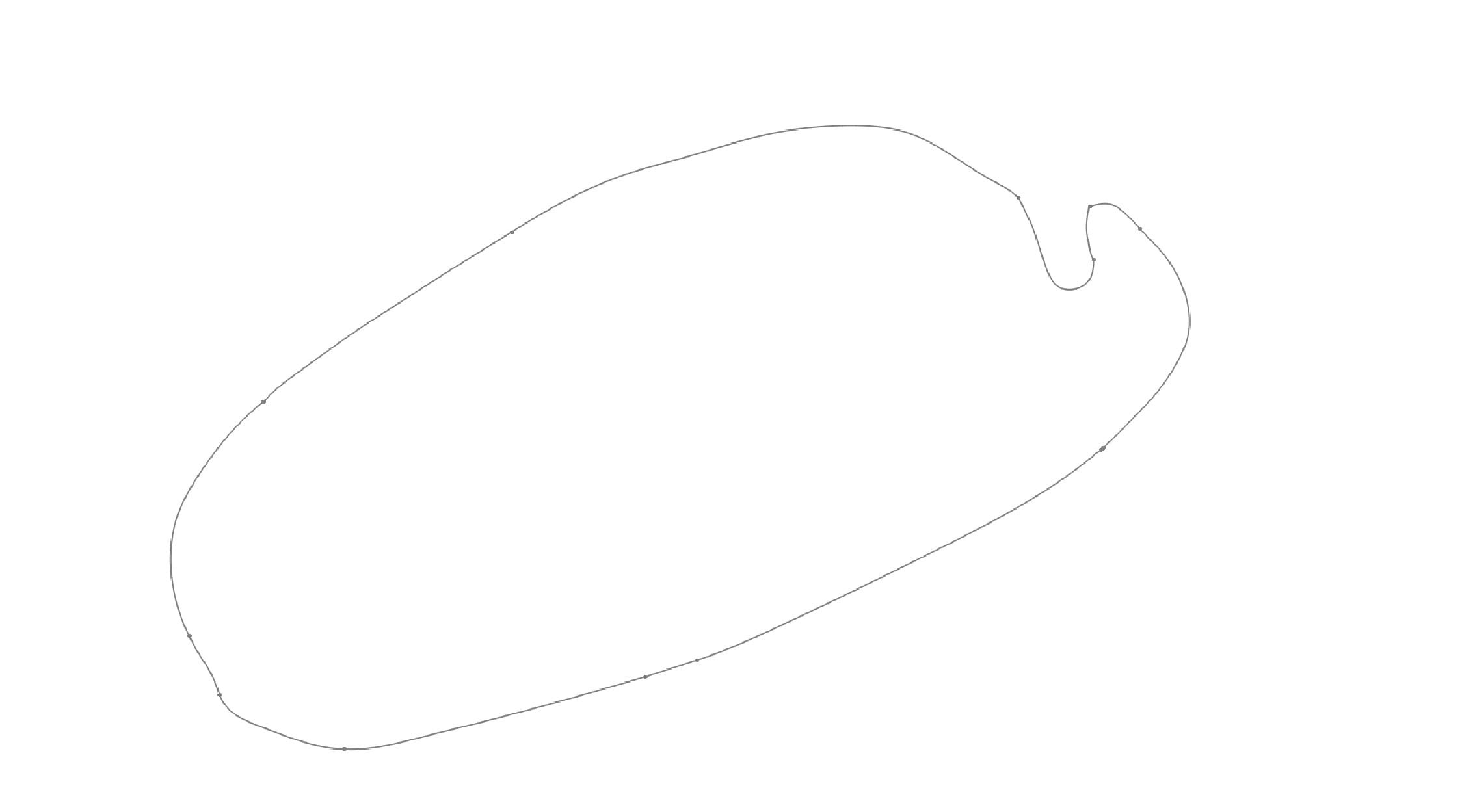

Supplement: Supplementary file 4 — Supporting Information [file ADVS-10-2203062-s013.zip › advs202203062-sup-0004-Supplementary-DataS3/Supplementary Data S3/105.jpg]

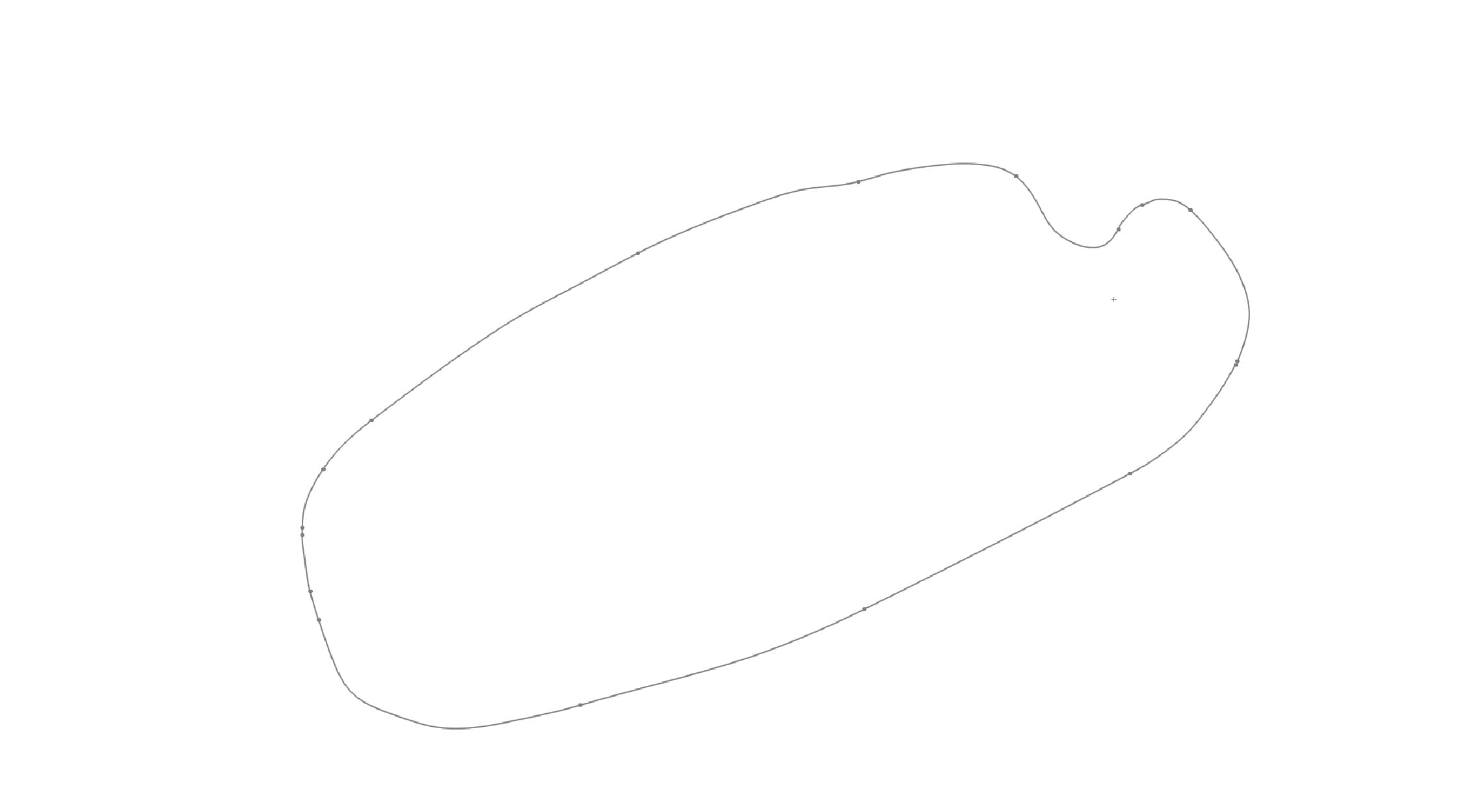

Supplement: Supplementary file 4 — Supporting Information [file ADVS-10-2203062-s013.zip › advs202203062-sup-0004-Supplementary-DataS3/Supplementary Data S3/106.jpg]

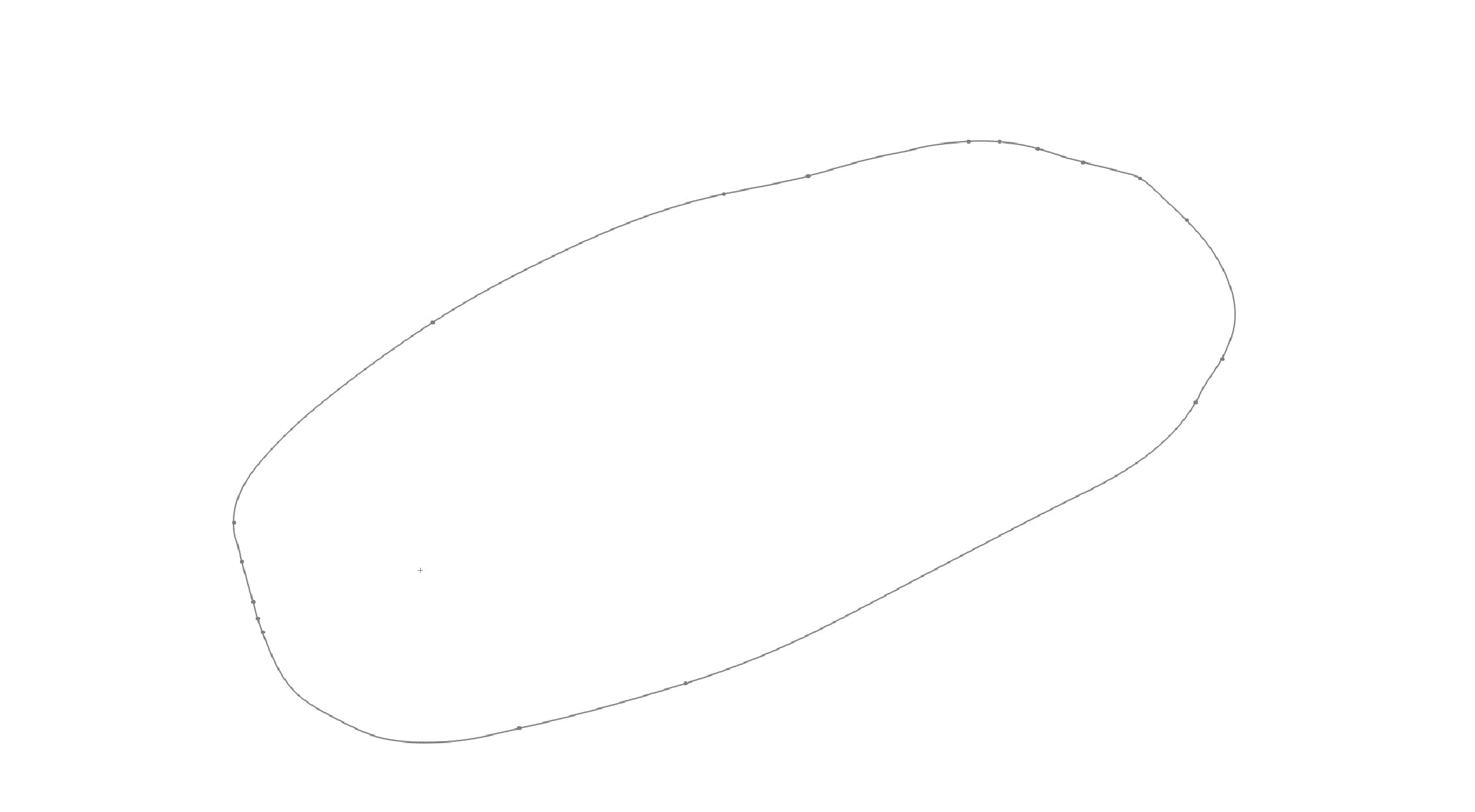

Supplement: Supplementary file 4 — Supporting Information [file ADVS-10-2203062-s013.zip › advs202203062-sup-0004-Supplementary-DataS3/Supplementary Data S3/107.jpg]

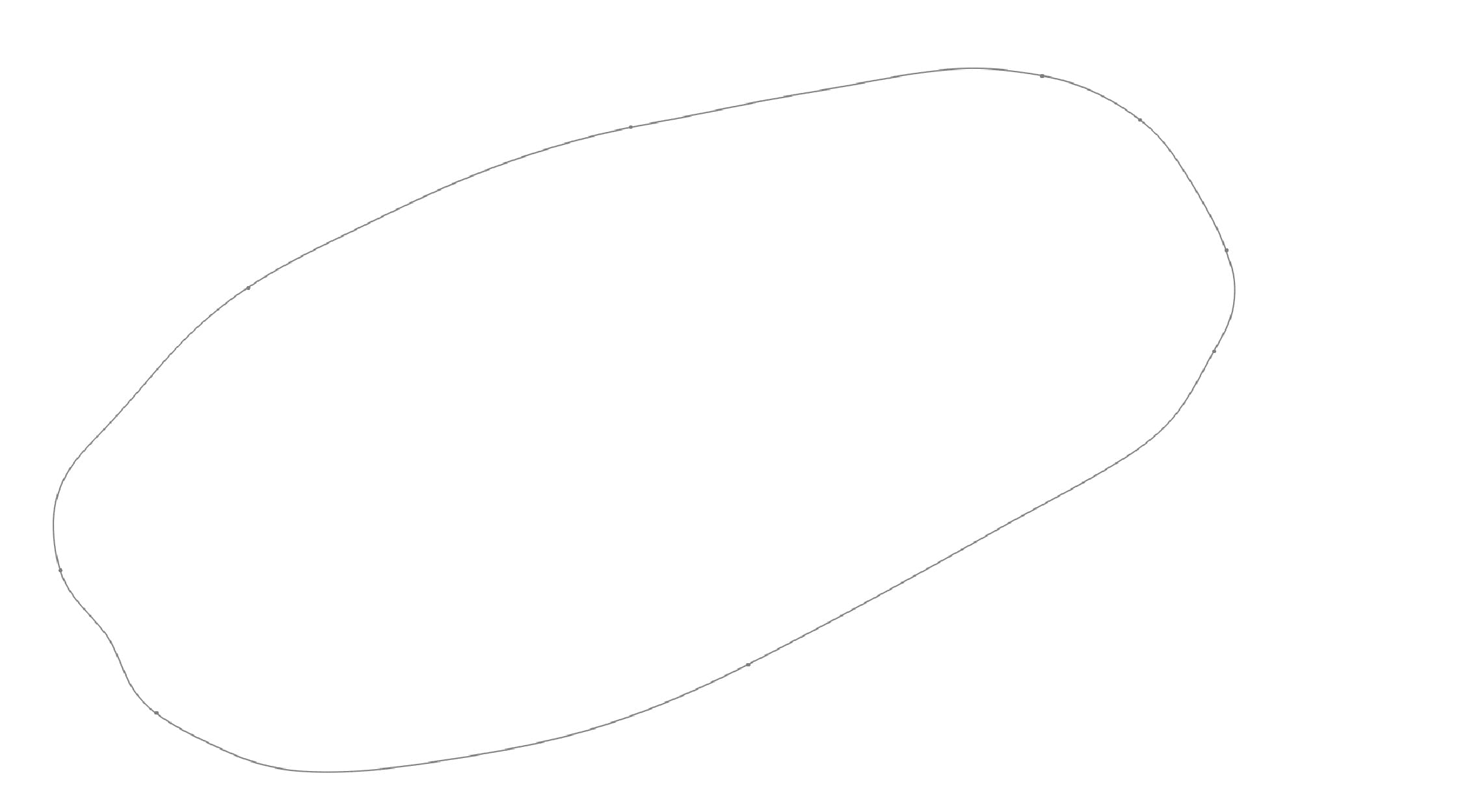

Supplement: Supplementary file 4 — Supporting Information [file ADVS-10-2203062-s013.zip › advs202203062-sup-0004-Supplementary-DataS3/Supplementary Data S3/108.jpg]

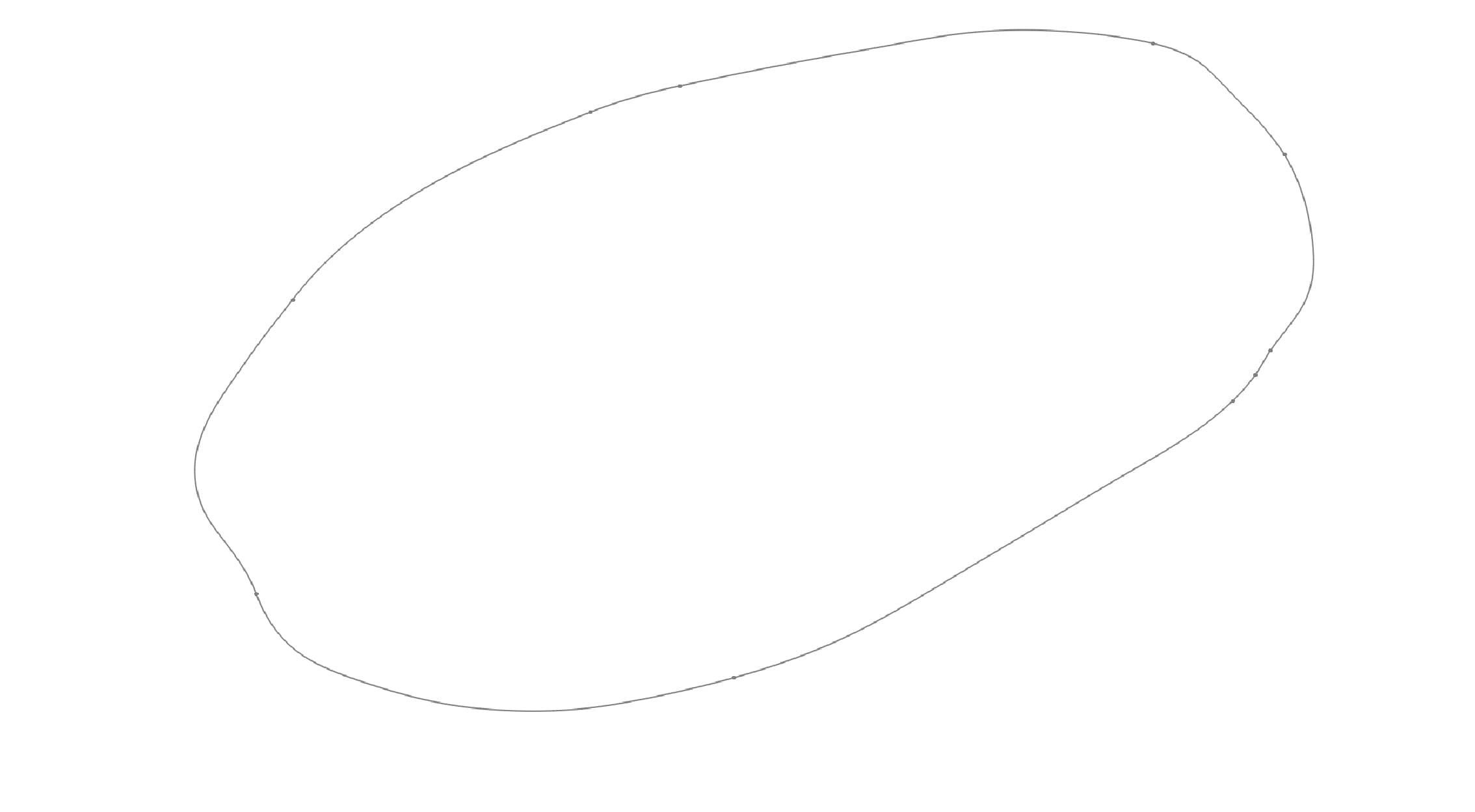

Supplement: Supplementary file 4 — Supporting Information [file ADVS-10-2203062-s013.zip › advs202203062-sup-0004-Supplementary-DataS3/Supplementary Data S3/109.jpg]

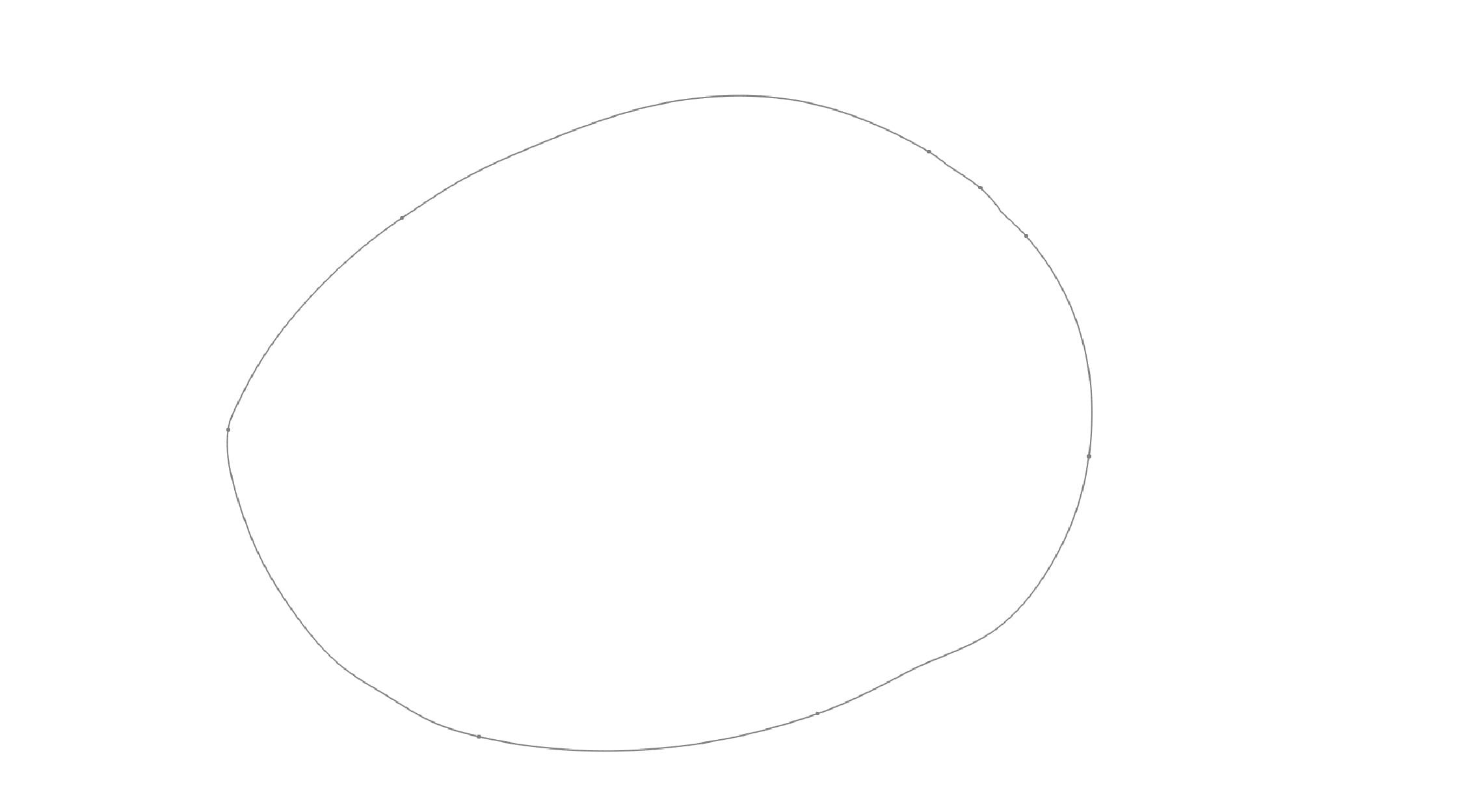

Supplement: Supplementary file 4 — Supporting Information [file ADVS-10-2203062-s013.zip › advs202203062-sup-0004-Supplementary-DataS3/Supplementary Data S3/11.jpg]

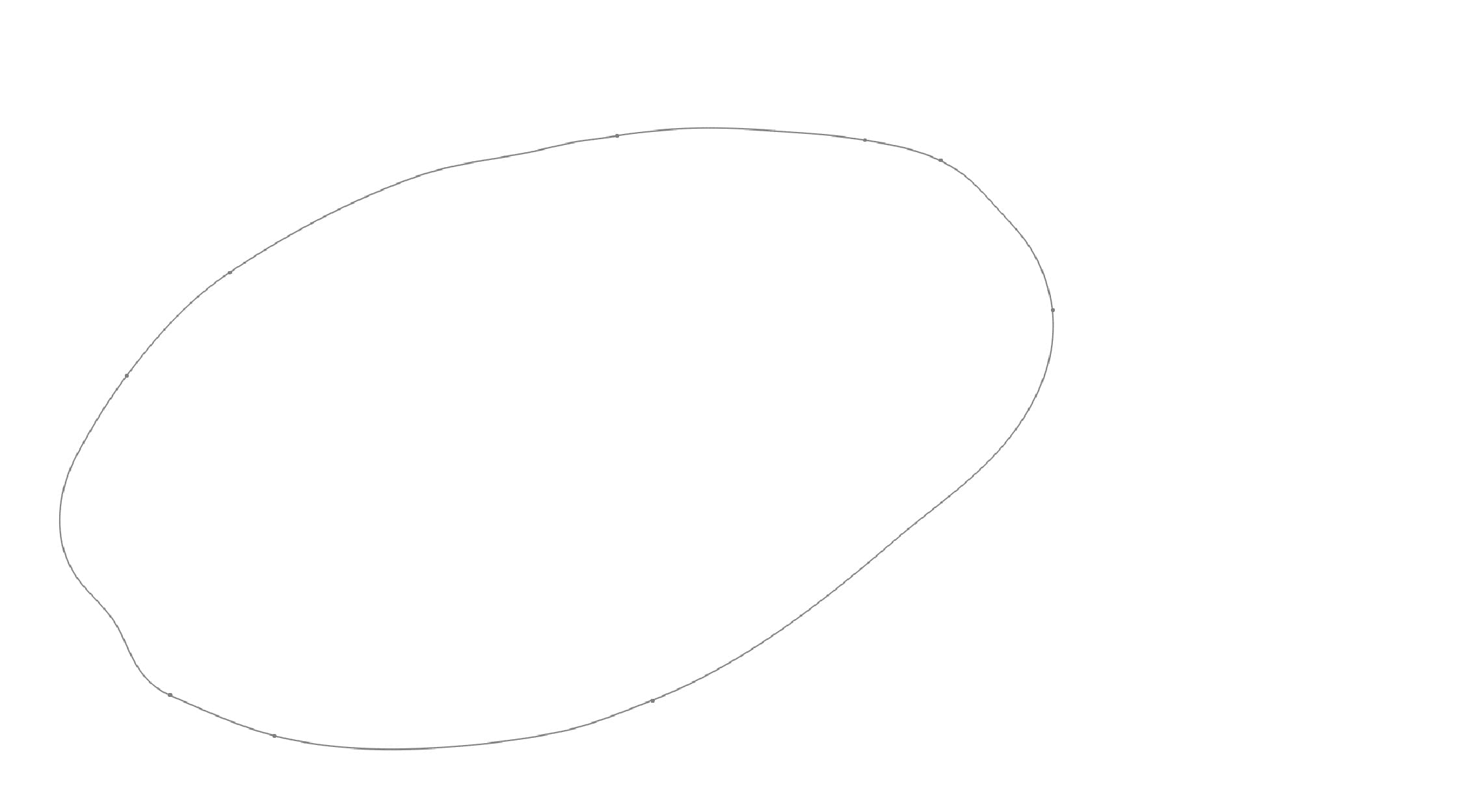

Supplement: Supplementary file 4 — Supporting Information [file ADVS-10-2203062-s013.zip › advs202203062-sup-0004-Supplementary-DataS3/Supplementary Data S3/110.jpg]

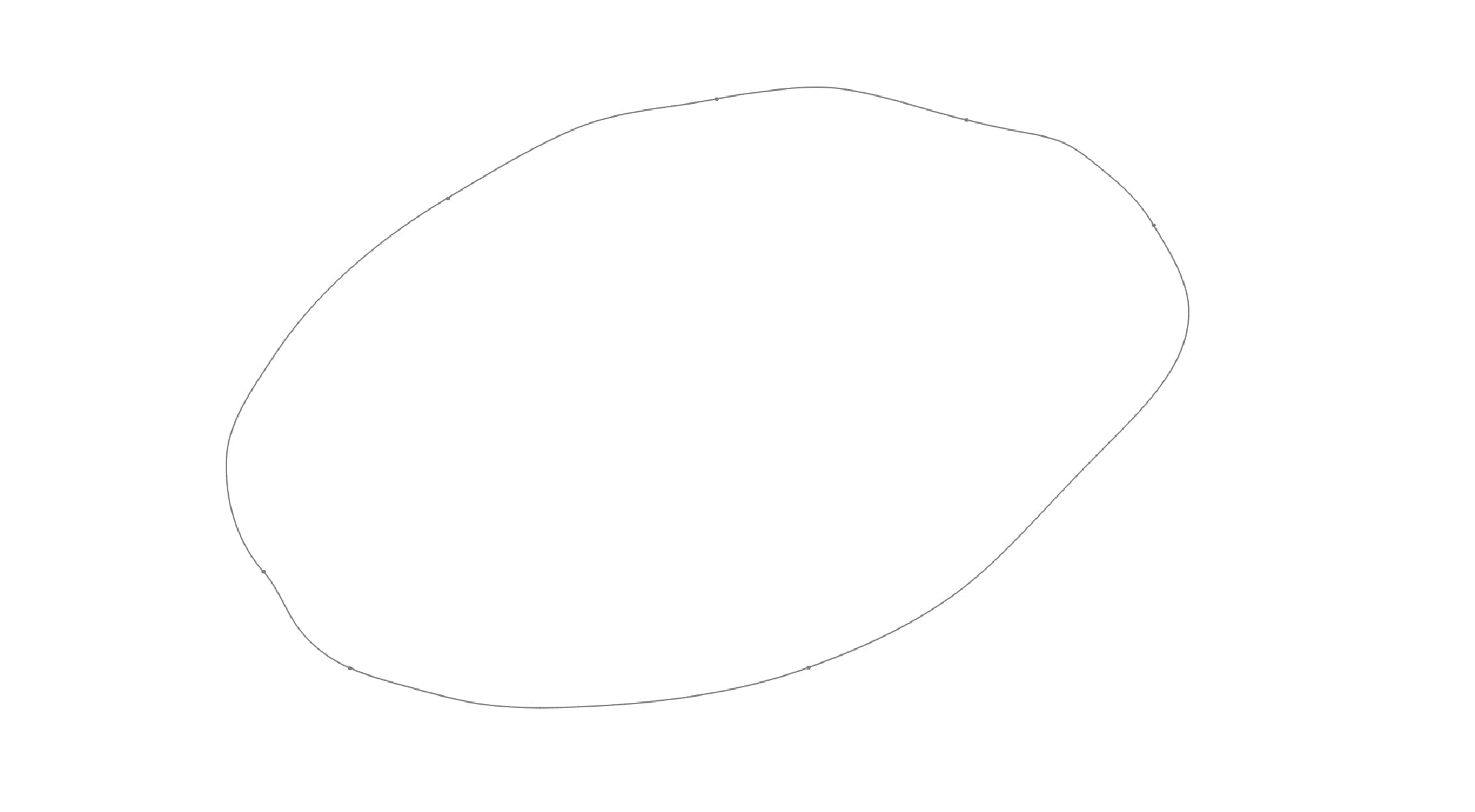

Supplement: Supplementary file 4 — Supporting Information [file ADVS-10-2203062-s013.zip › advs202203062-sup-0004-Supplementary-DataS3/Supplementary Data S3/111.jpg]

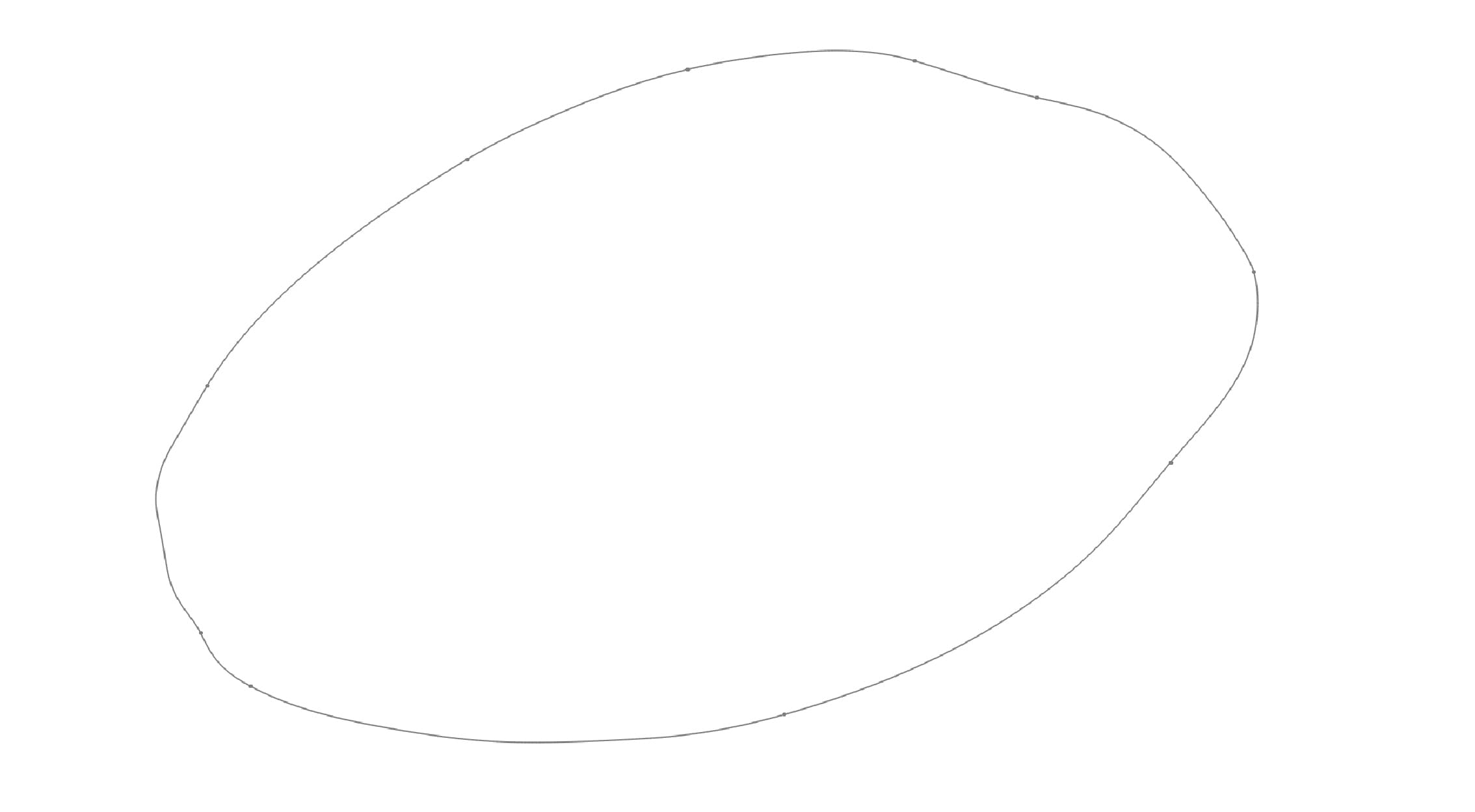

Supplement: Supplementary file 4 — Supporting Information [file ADVS-10-2203062-s013.zip › advs202203062-sup-0004-Supplementary-DataS3/Supplementary Data S3/112.jpg]

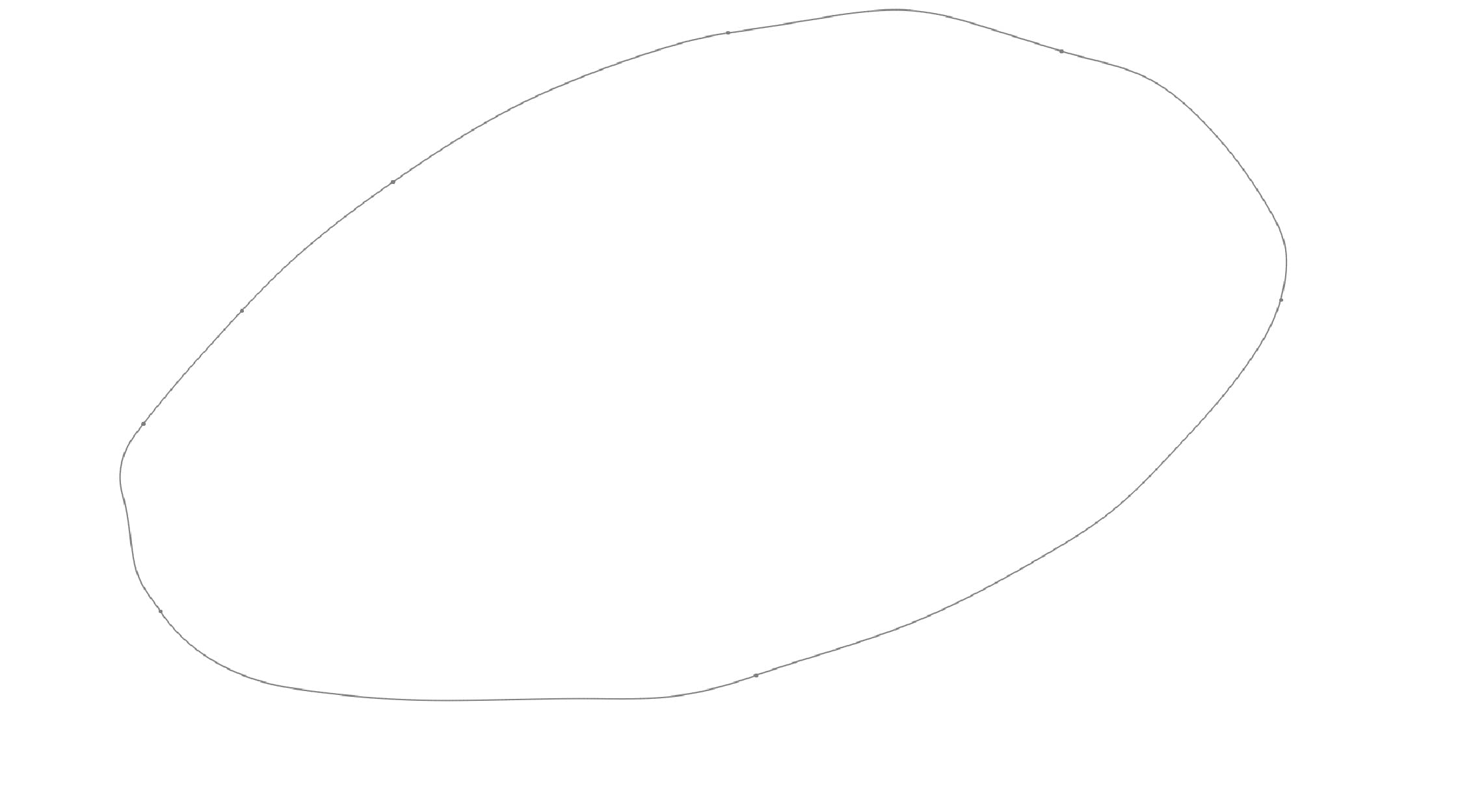

Supplement: Supplementary file 4 — Supporting Information [file ADVS-10-2203062-s013.zip › advs202203062-sup-0004-Supplementary-DataS3/Supplementary Data S3/113.jpg]

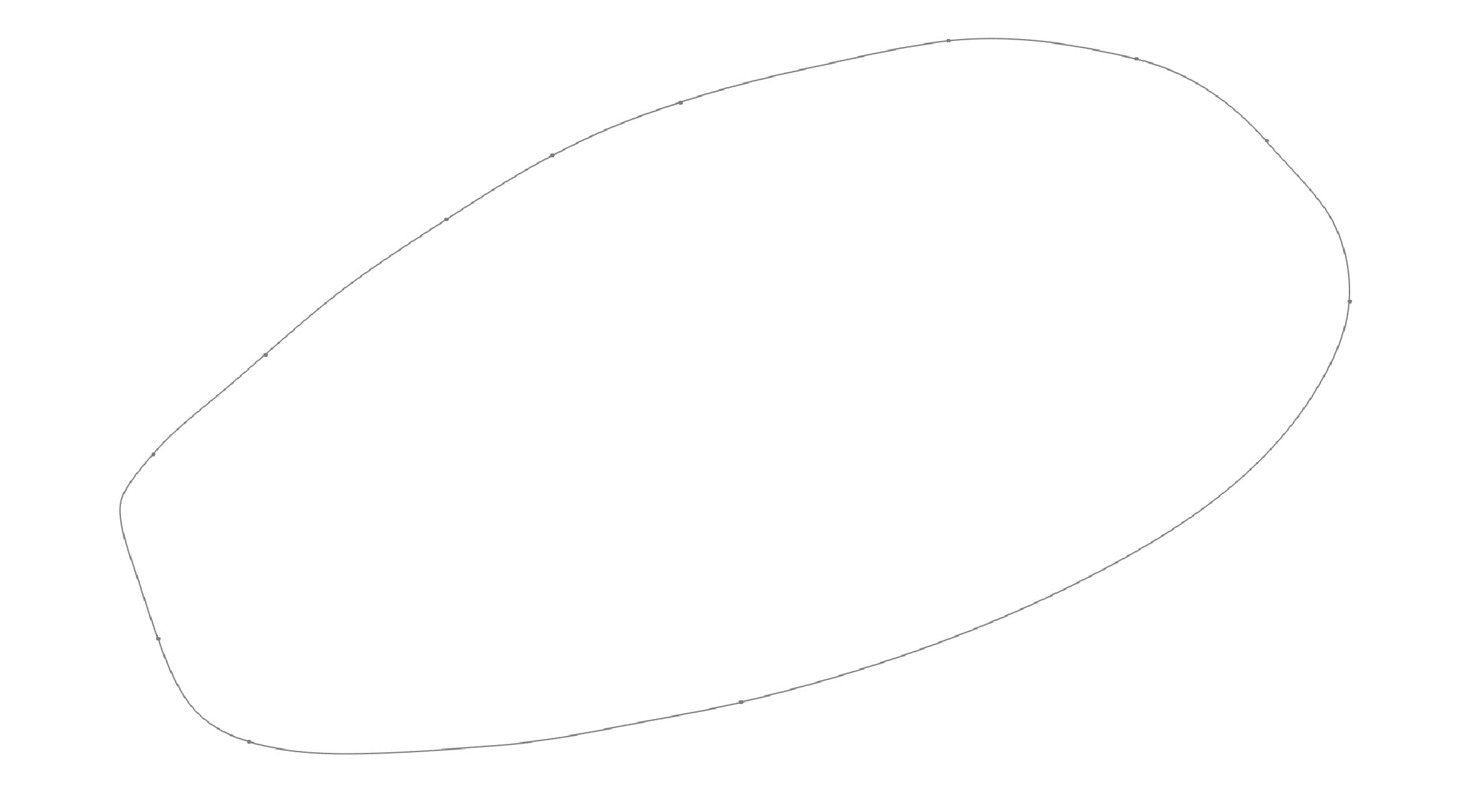

Supplement: Supplementary file 4 — Supporting Information [file ADVS-10-2203062-s013.zip › advs202203062-sup-0004-Supplementary-DataS3/Supplementary Data S3/114.jpg]

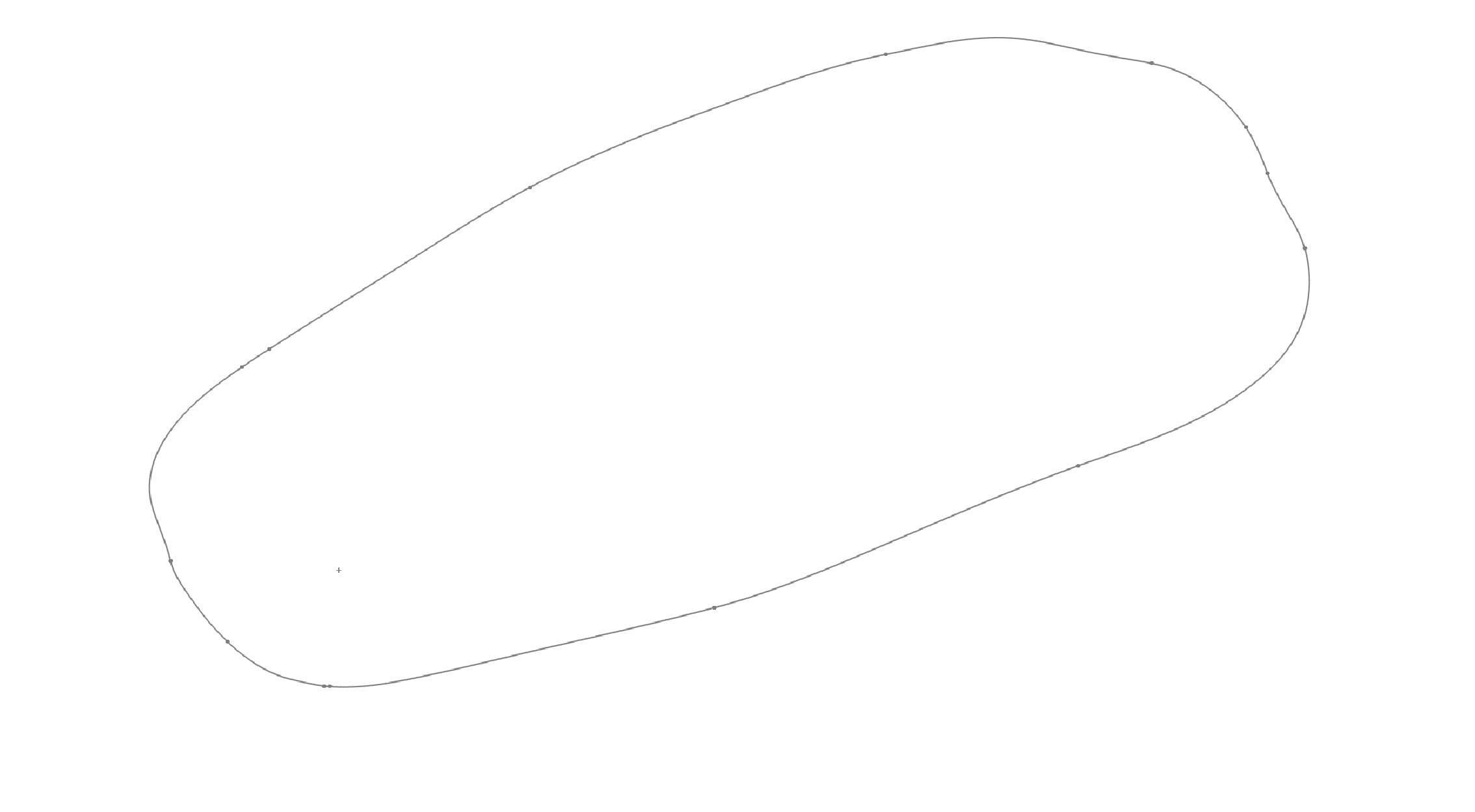

Supplement: Supplementary file 4 — Supporting Information [file ADVS-10-2203062-s013.zip › advs202203062-sup-0004-Supplementary-DataS3/Supplementary Data S3/115.jpg]

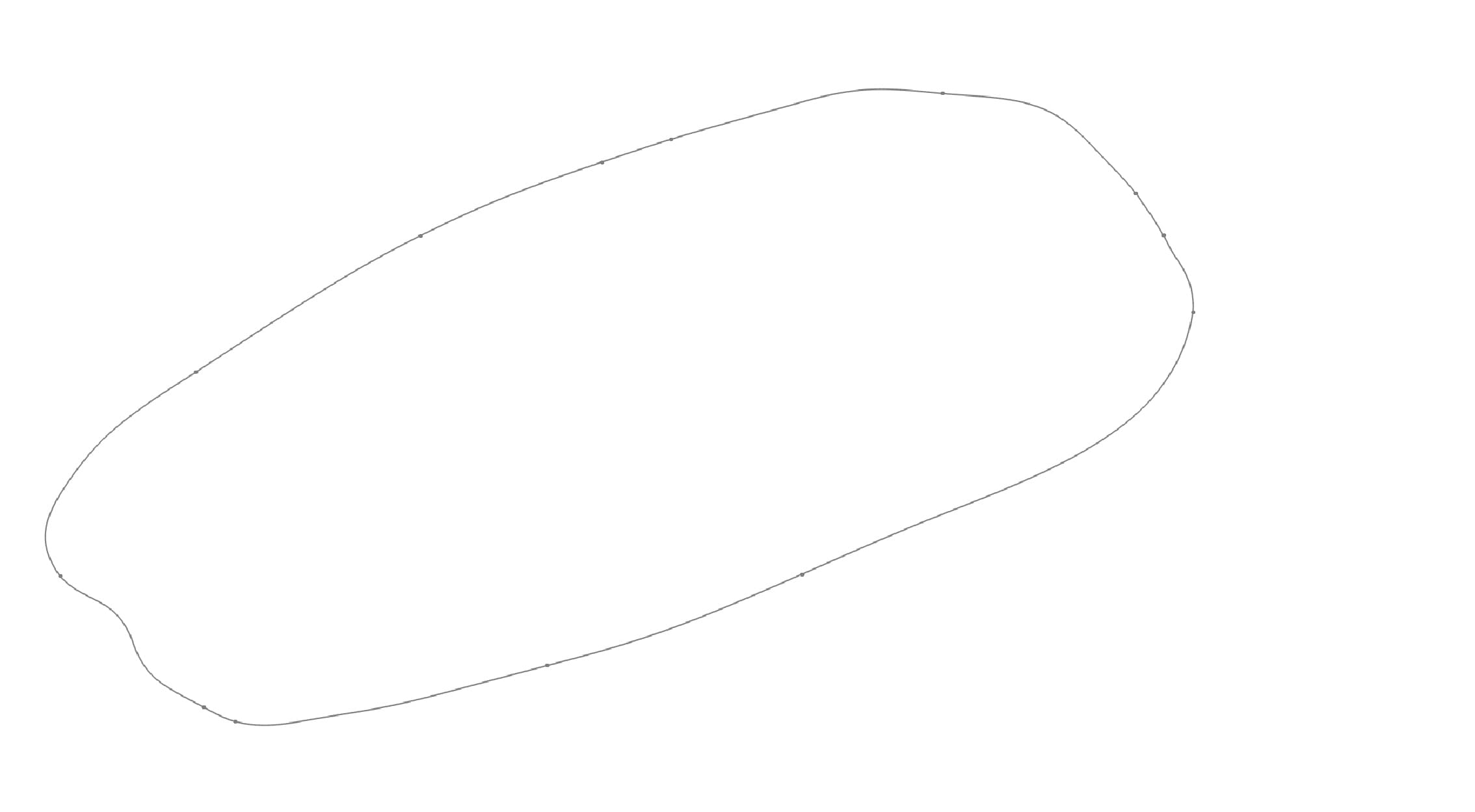

Supplement: Supplementary file 4 — Supporting Information [file ADVS-10-2203062-s013.zip › advs202203062-sup-0004-Supplementary-DataS3/Supplementary Data S3/116.jpg]

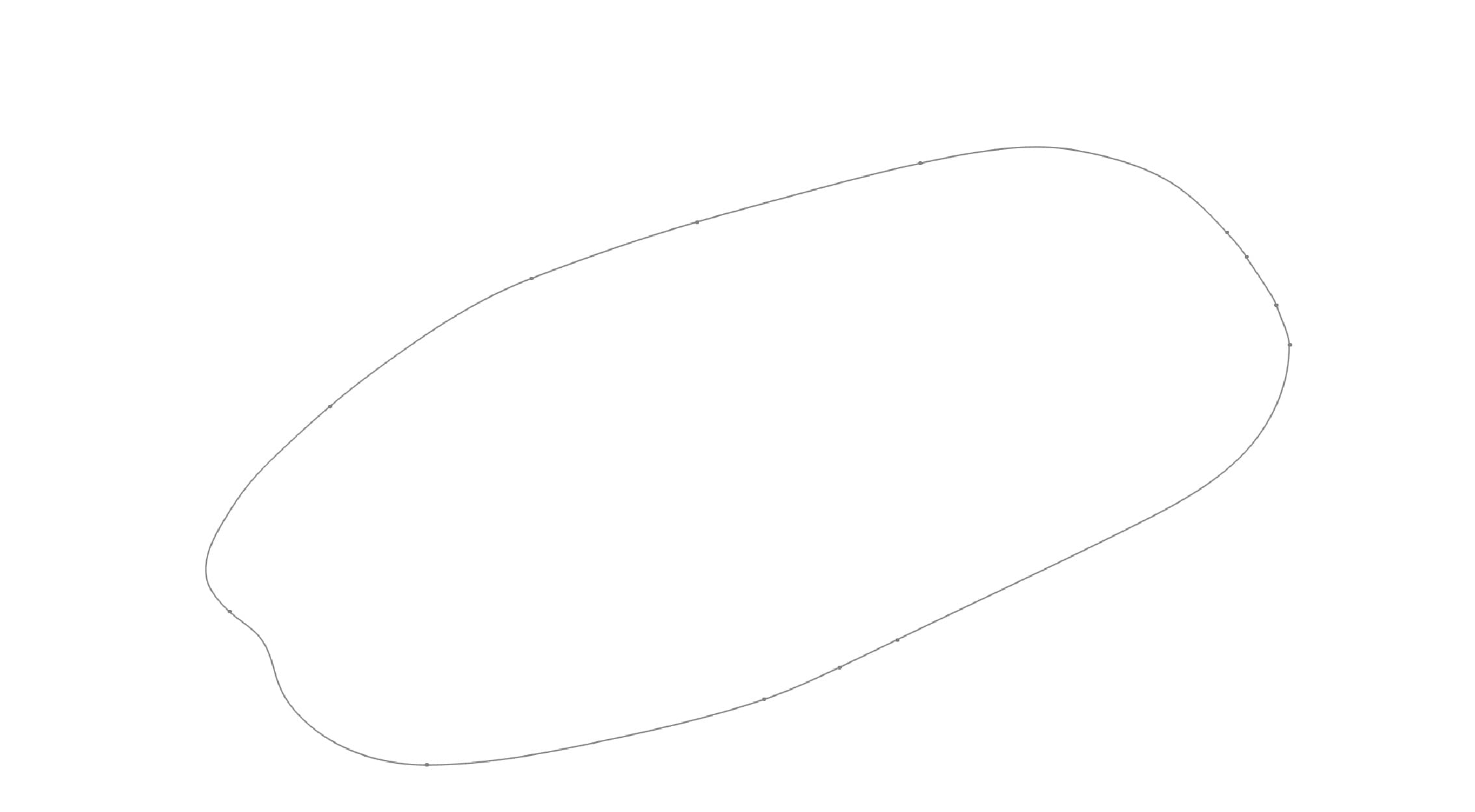

Supplement: Supplementary file 4 — Supporting Information [file ADVS-10-2203062-s013.zip › advs202203062-sup-0004-Supplementary-DataS3/Supplementary Data S3/117.jpg]

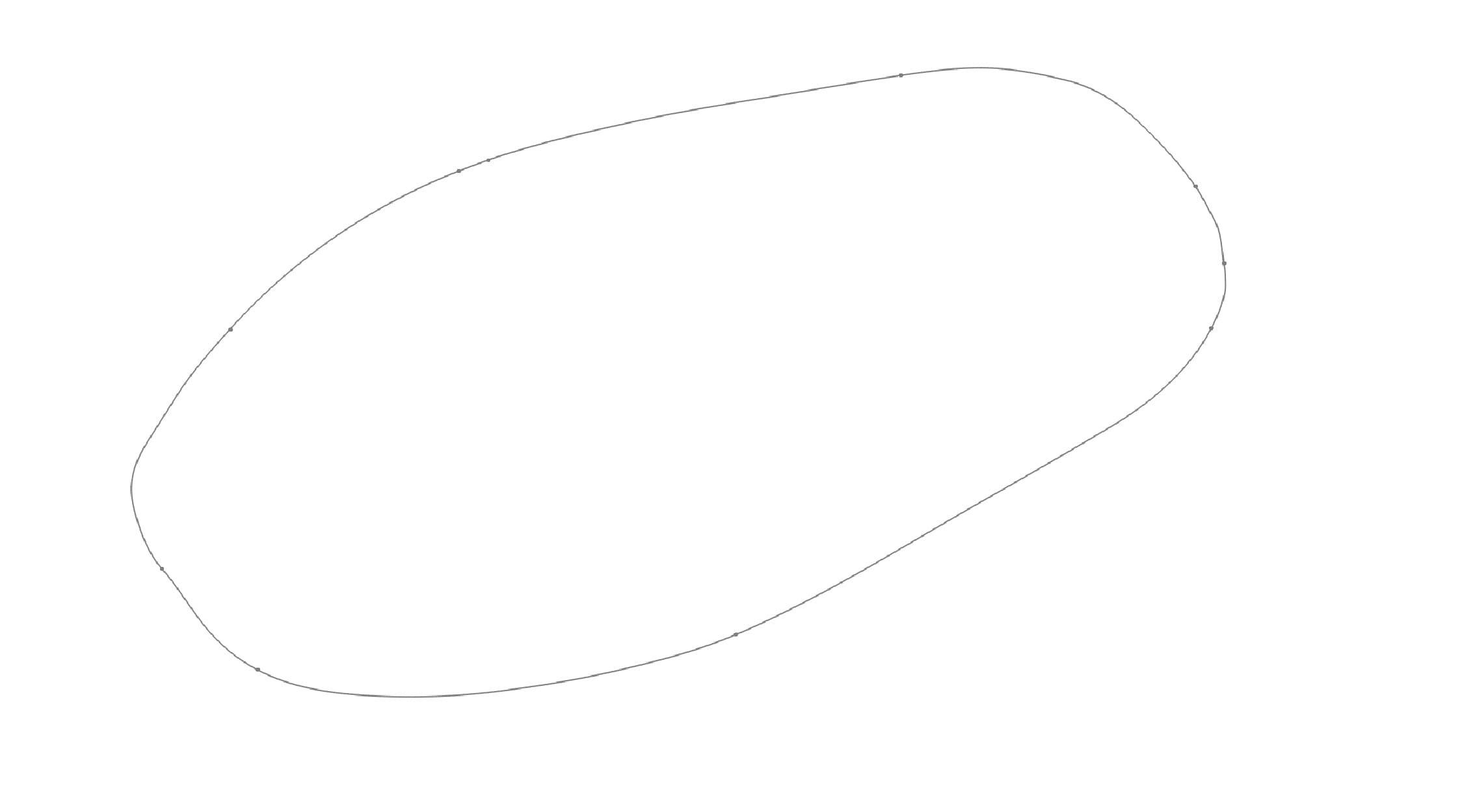

Supplement: Supplementary file 4 — Supporting Information [file ADVS-10-2203062-s013.zip › advs202203062-sup-0004-Supplementary-DataS3/Supplementary Data S3/118.jpg]

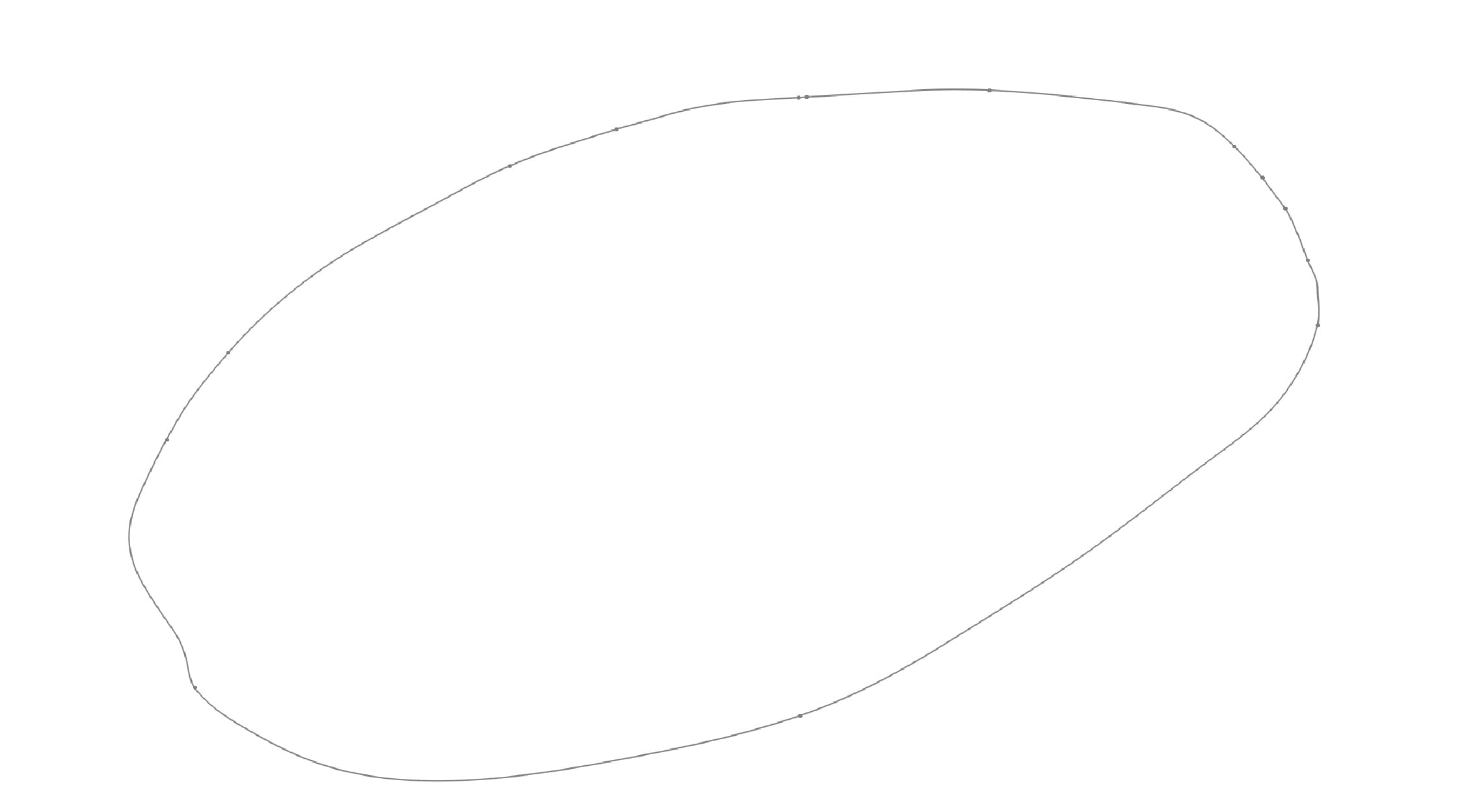

Supplement: Supplementary file 4 — Supporting Information [file ADVS-10-2203062-s013.zip › advs202203062-sup-0004-Supplementary-DataS3/Supplementary Data S3/119.jpg]

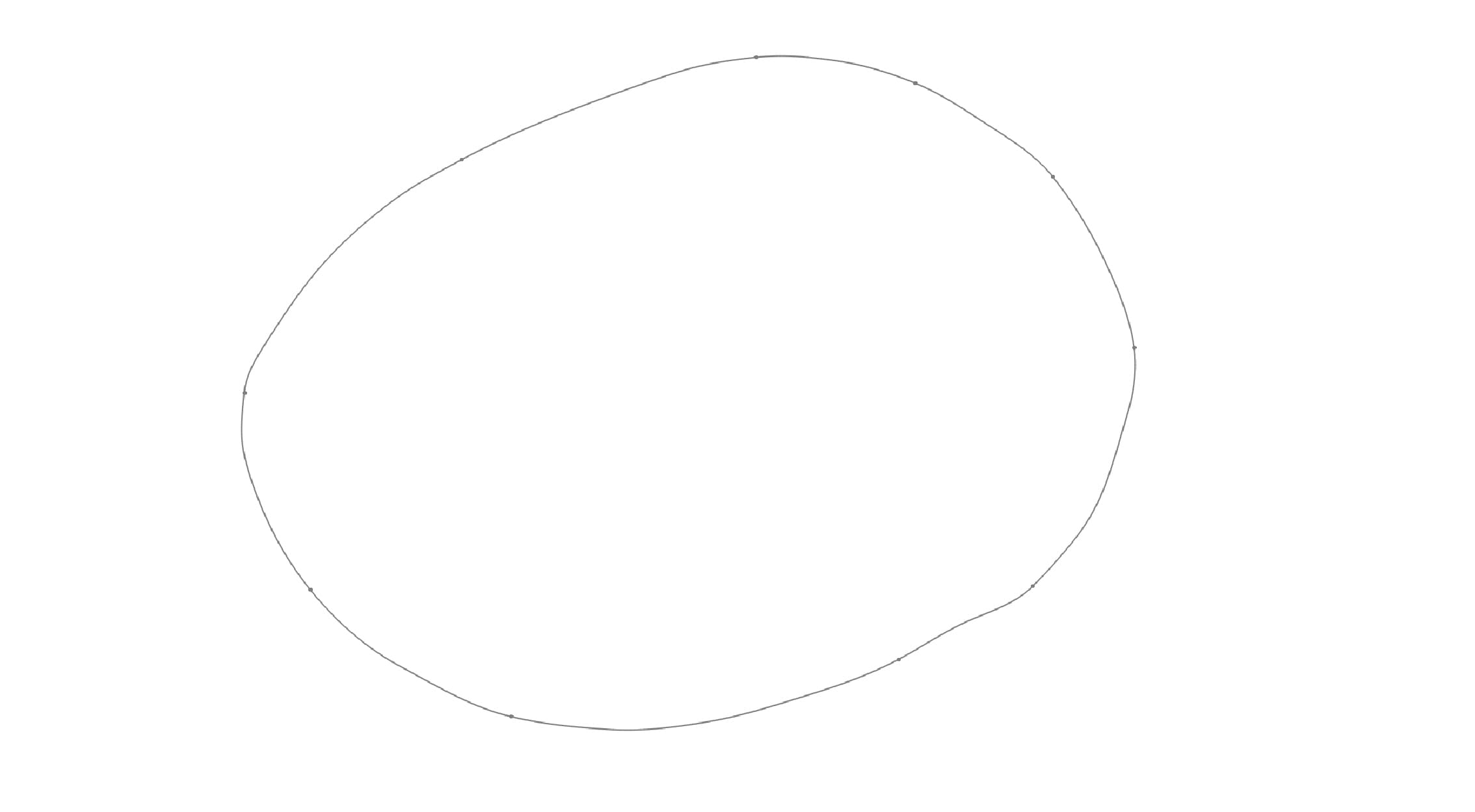

Supplement: Supplementary file 4 — Supporting Information [file ADVS-10-2203062-s013.zip › advs202203062-sup-0004-Supplementary-DataS3/Supplementary Data S3/12.jpg]

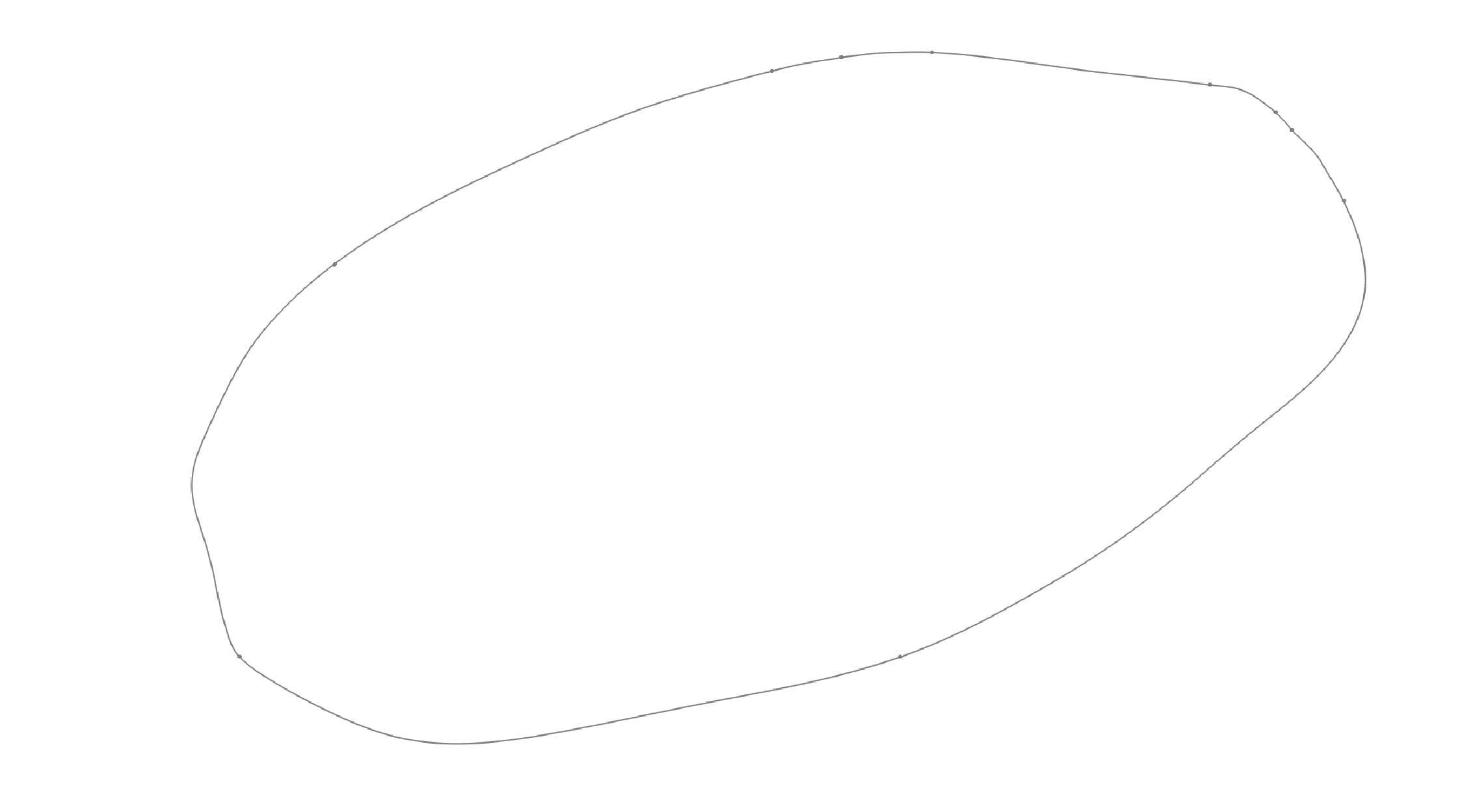

Supplement: Supplementary file 4 — Supporting Information [file ADVS-10-2203062-s013.zip › advs202203062-sup-0004-Supplementary-DataS3/Supplementary Data S3/120.jpg]

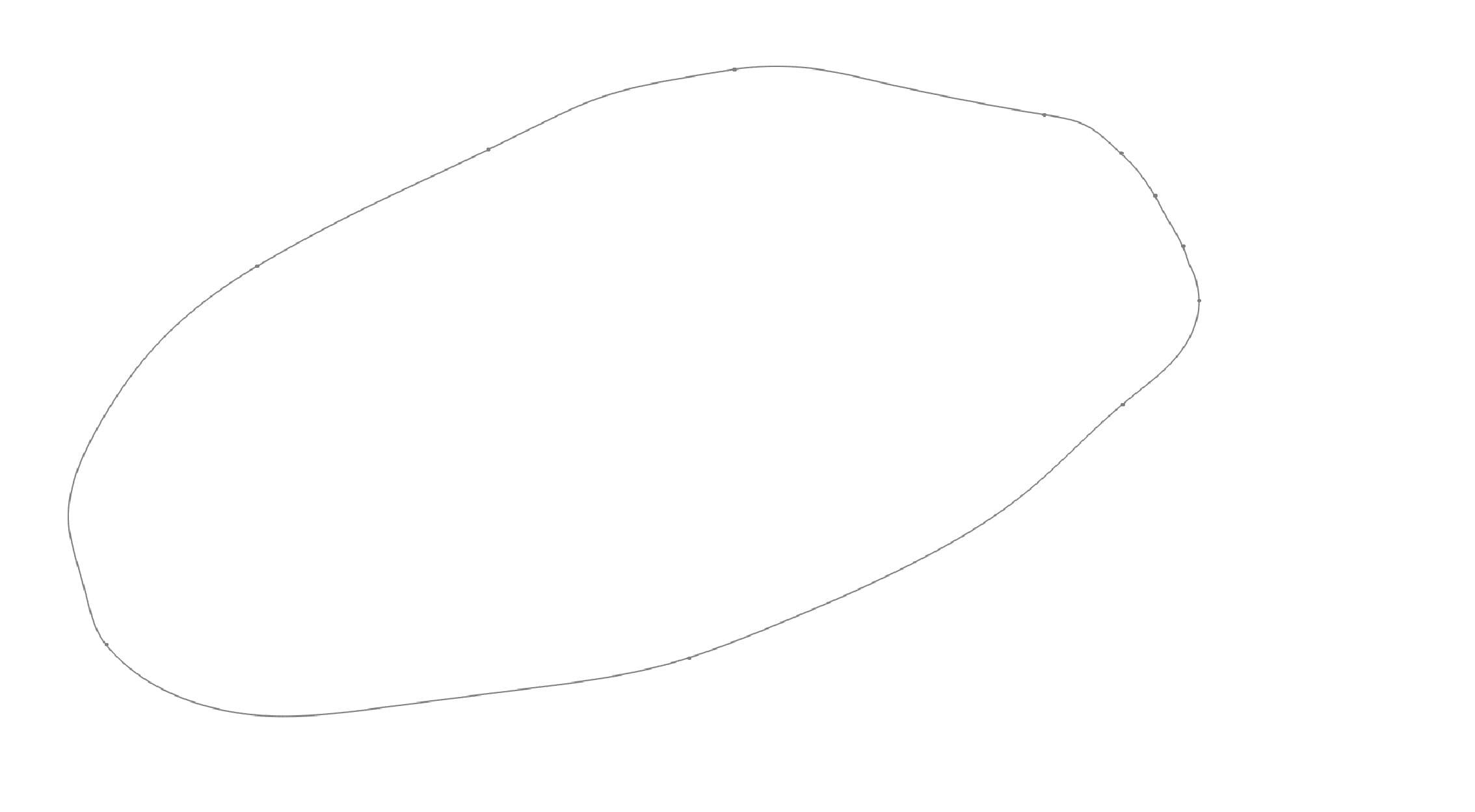

Supplement: Supplementary file 4 — Supporting Information [file ADVS-10-2203062-s013.zip › advs202203062-sup-0004-Supplementary-DataS3/Supplementary Data S3/121.jpg]

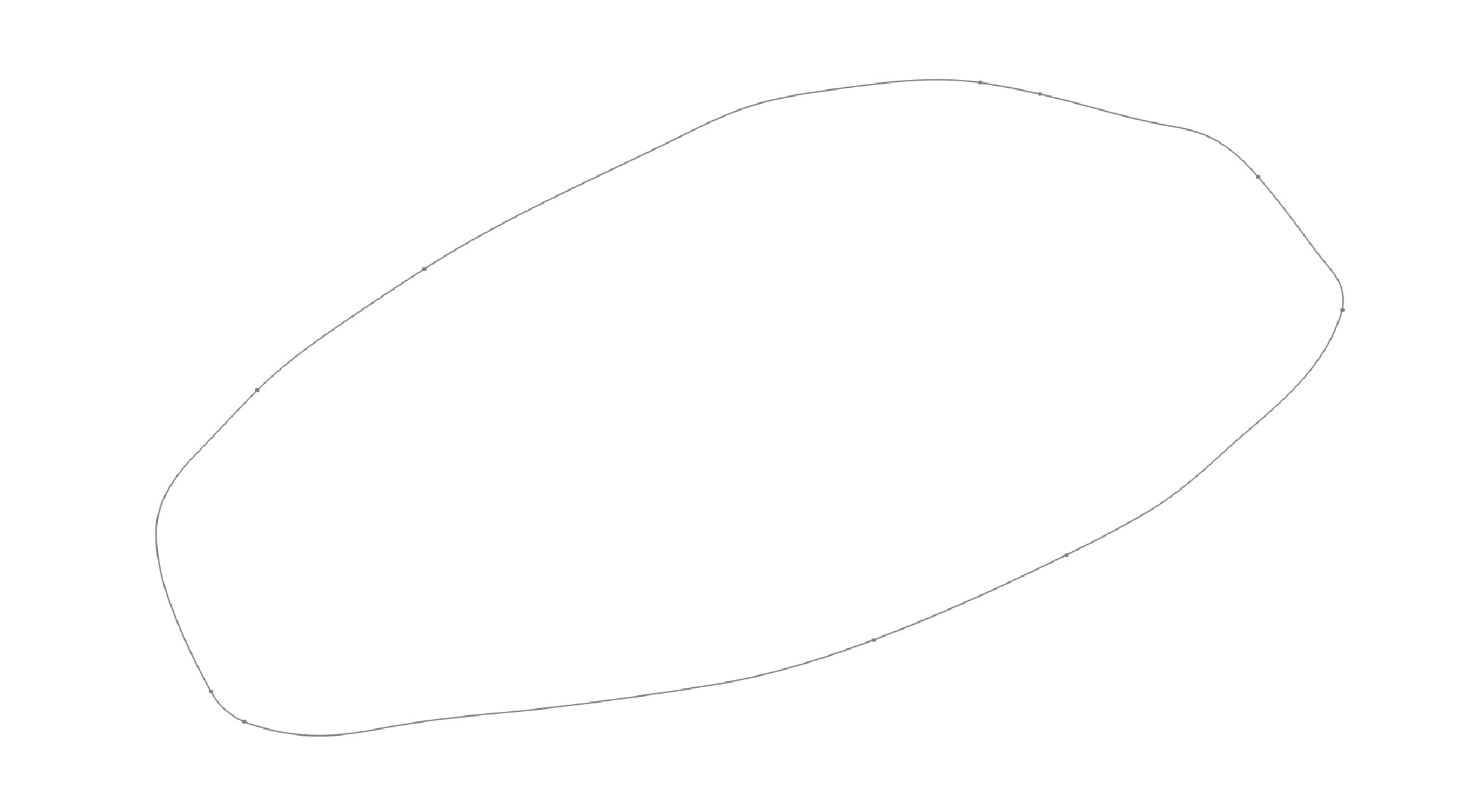

Supplement: Supplementary file 4 — Supporting Information [file ADVS-10-2203062-s013.zip › advs202203062-sup-0004-Supplementary-DataS3/Supplementary Data S3/122.jpg]

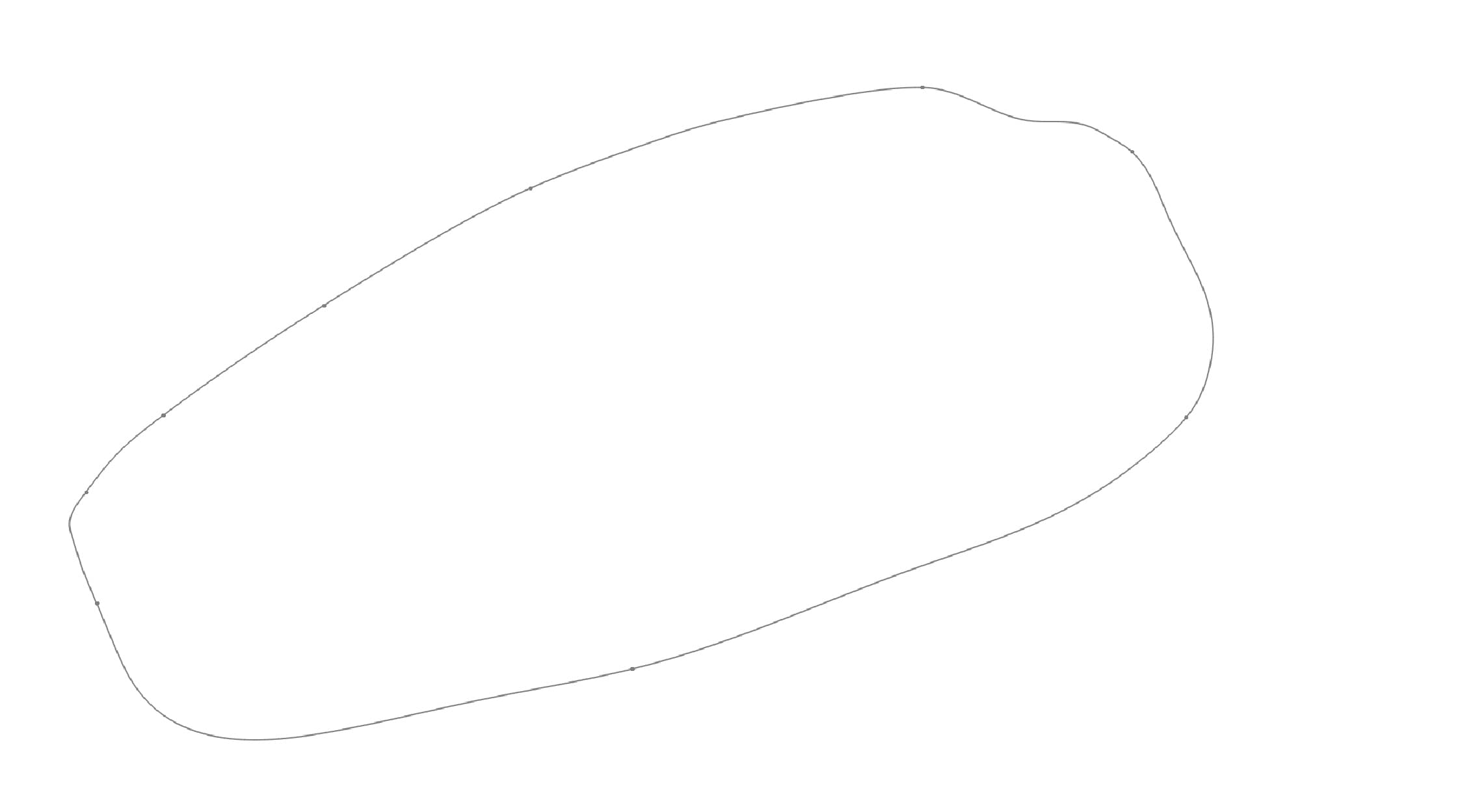

Supplement: Supplementary file 4 — Supporting Information [file ADVS-10-2203062-s013.zip › advs202203062-sup-0004-Supplementary-DataS3/Supplementary Data S3/123.jpg]

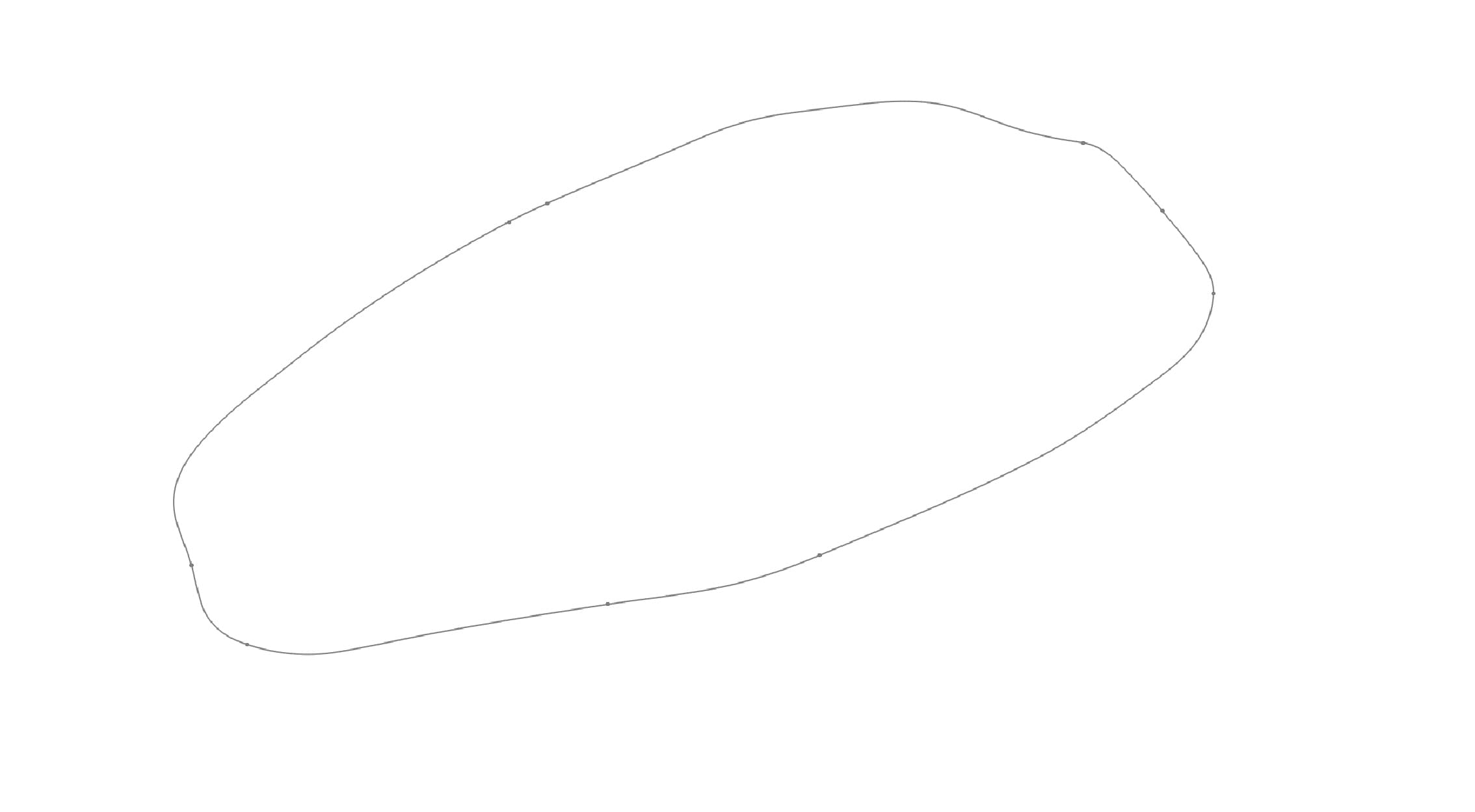

Supplement: Supplementary file 4 — Supporting Information [file ADVS-10-2203062-s013.zip › advs202203062-sup-0004-Supplementary-DataS3/Supplementary Data S3/124.jpg]

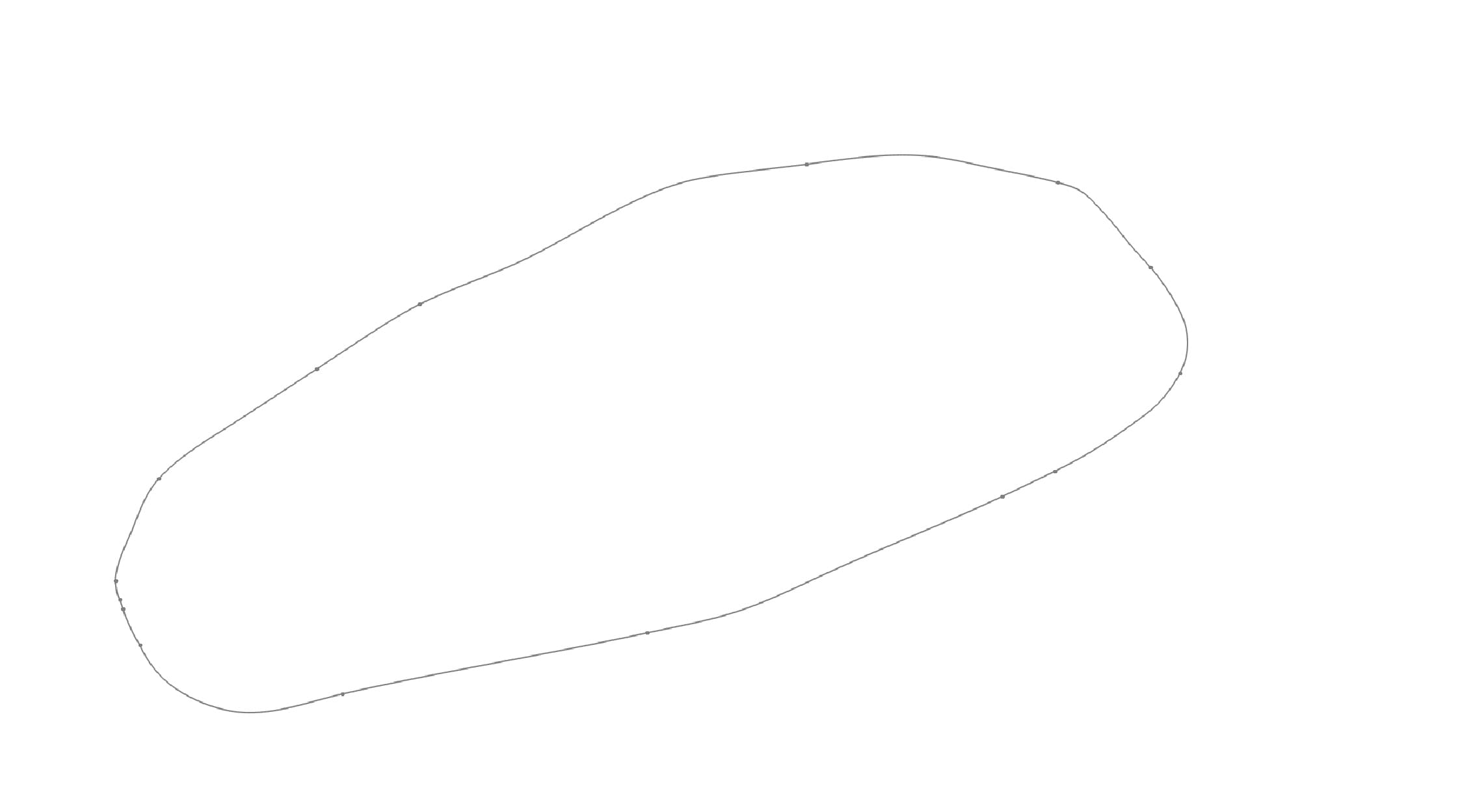

Supplement: Supplementary file 4 — Supporting Information [file ADVS-10-2203062-s013.zip › advs202203062-sup-0004-Supplementary-DataS3/Supplementary Data S3/125.jpg]

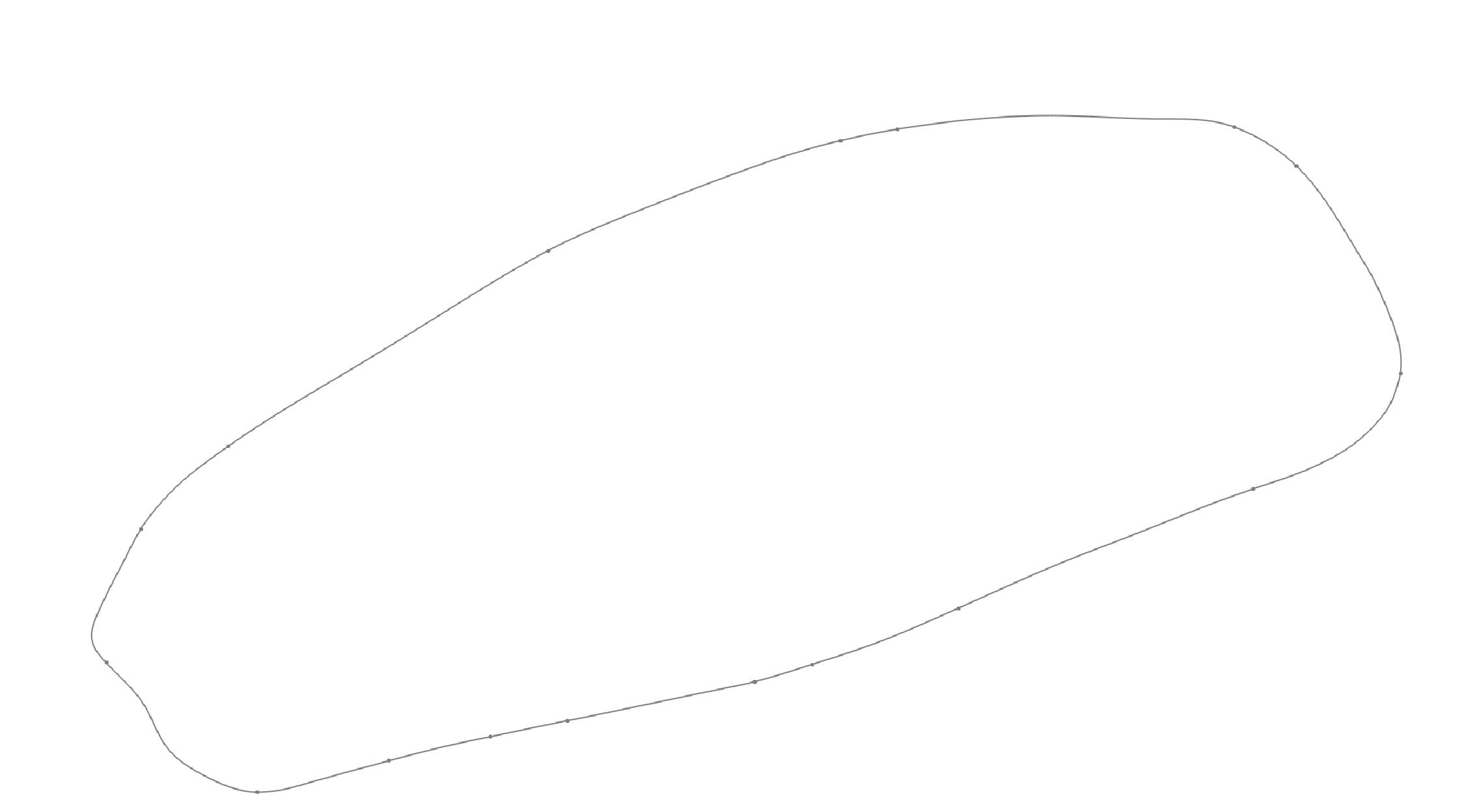

Supplement: Supplementary file 4 — Supporting Information [file ADVS-10-2203062-s013.zip › advs202203062-sup-0004-Supplementary-DataS3/Supplementary Data S3/126.jpg]

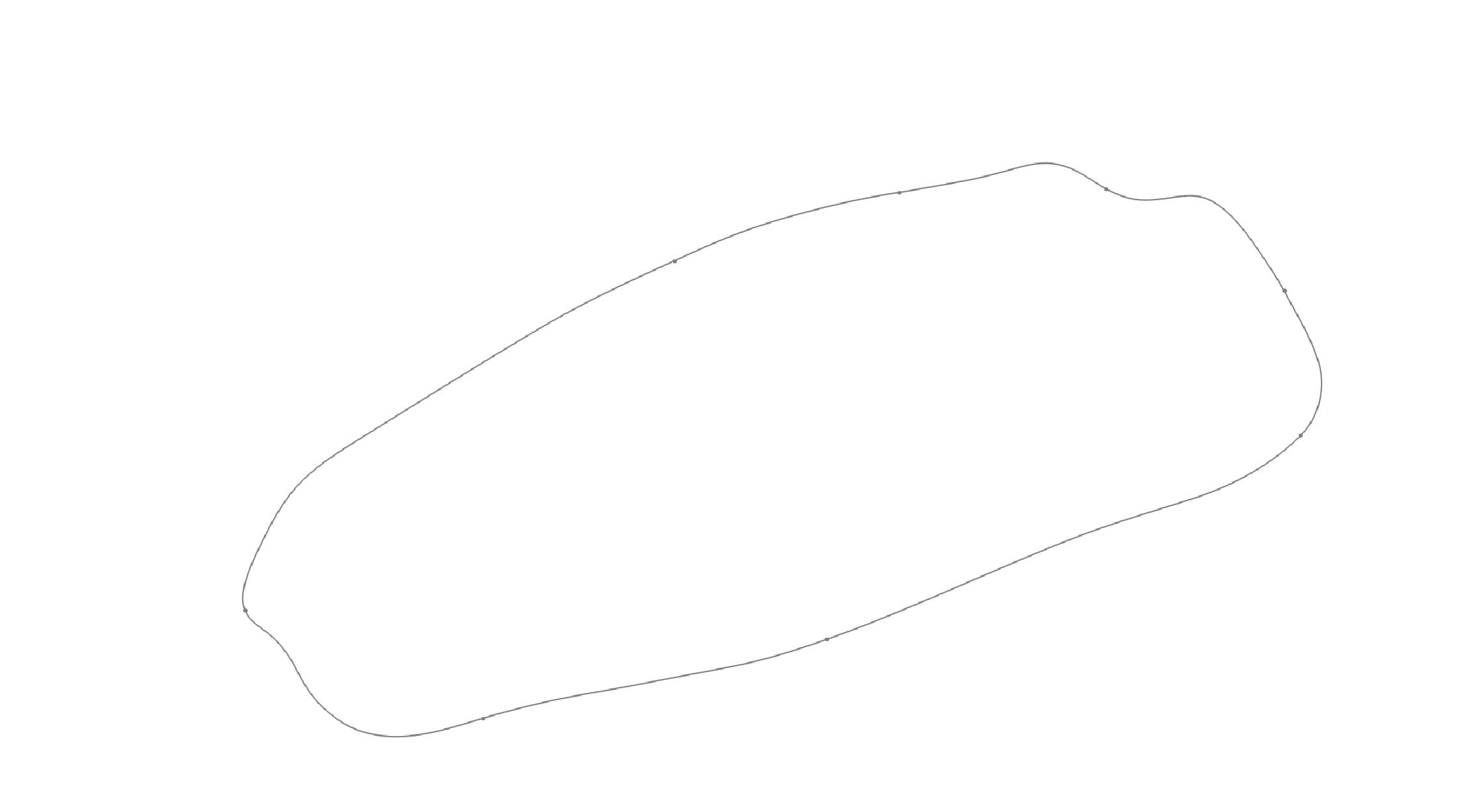

Supplement: Supplementary file 4 — Supporting Information [file ADVS-10-2203062-s013.zip › advs202203062-sup-0004-Supplementary-DataS3/Supplementary Data S3/127.jpg]

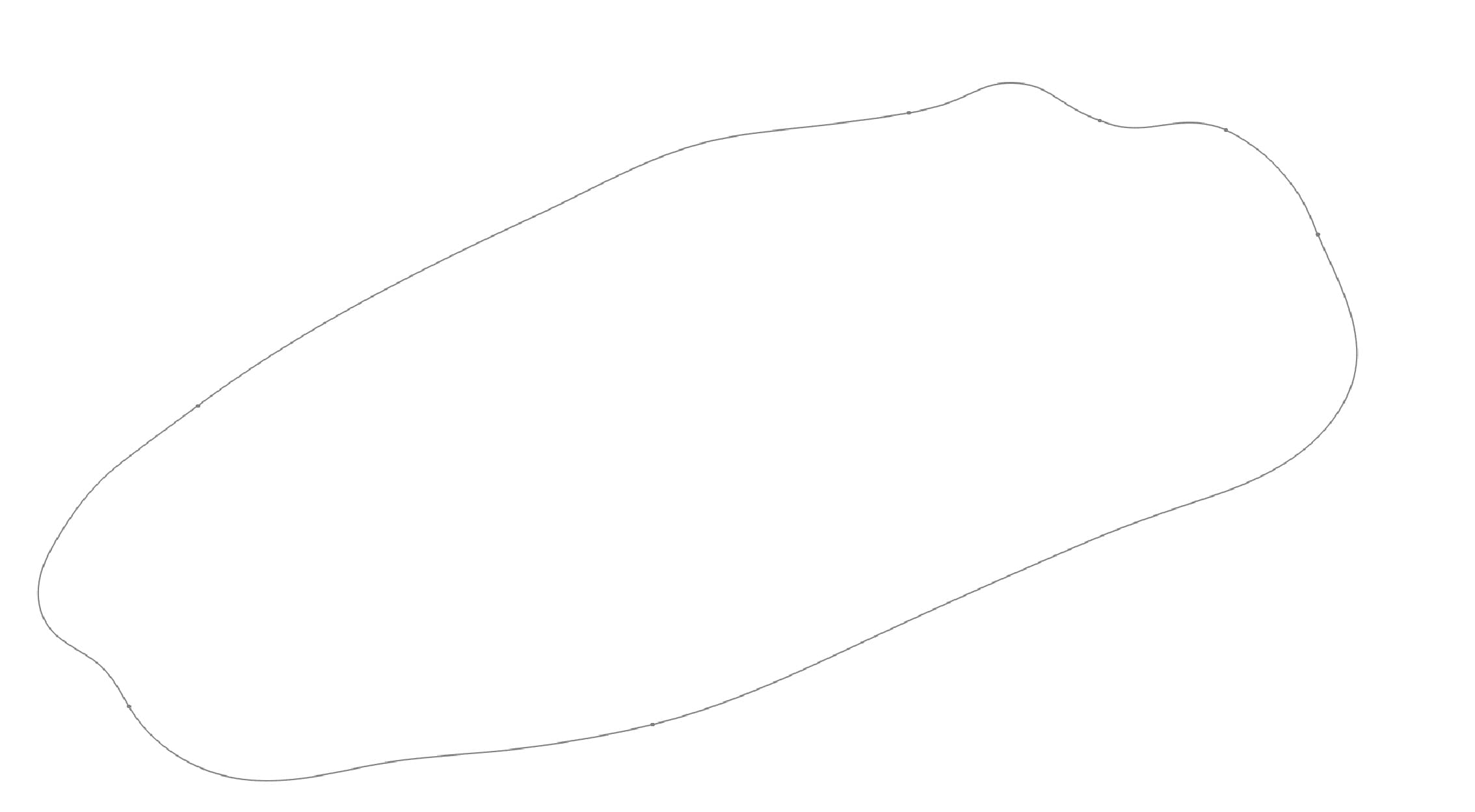

Supplement: Supplementary file 4 — Supporting Information [file ADVS-10-2203062-s013.zip › advs202203062-sup-0004-Supplementary-DataS3/Supplementary Data S3/128.jpg]

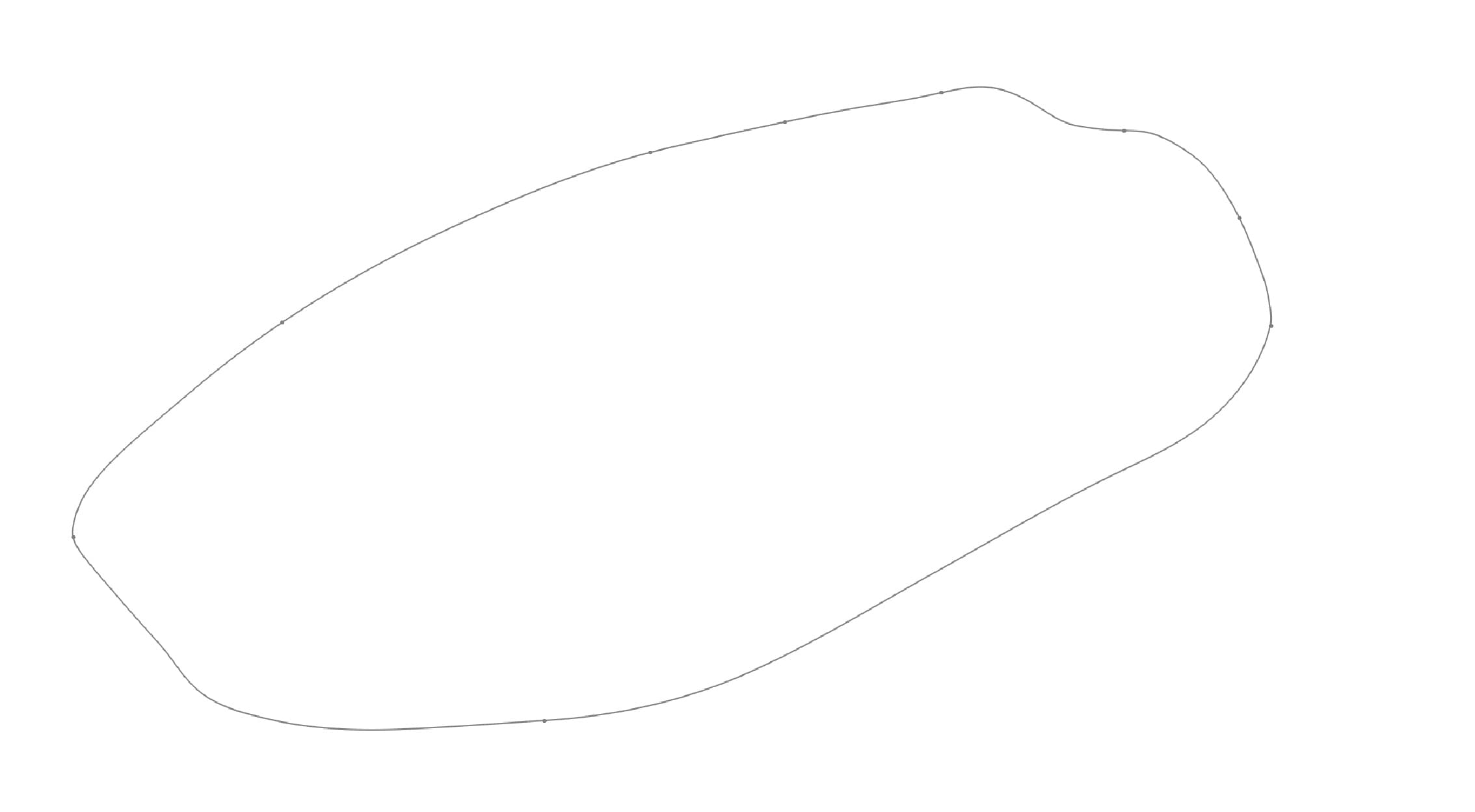

Supplement: Supplementary file 4 — Supporting Information [file ADVS-10-2203062-s013.zip › advs202203062-sup-0004-Supplementary-DataS3/Supplementary Data S3/129.jpg]

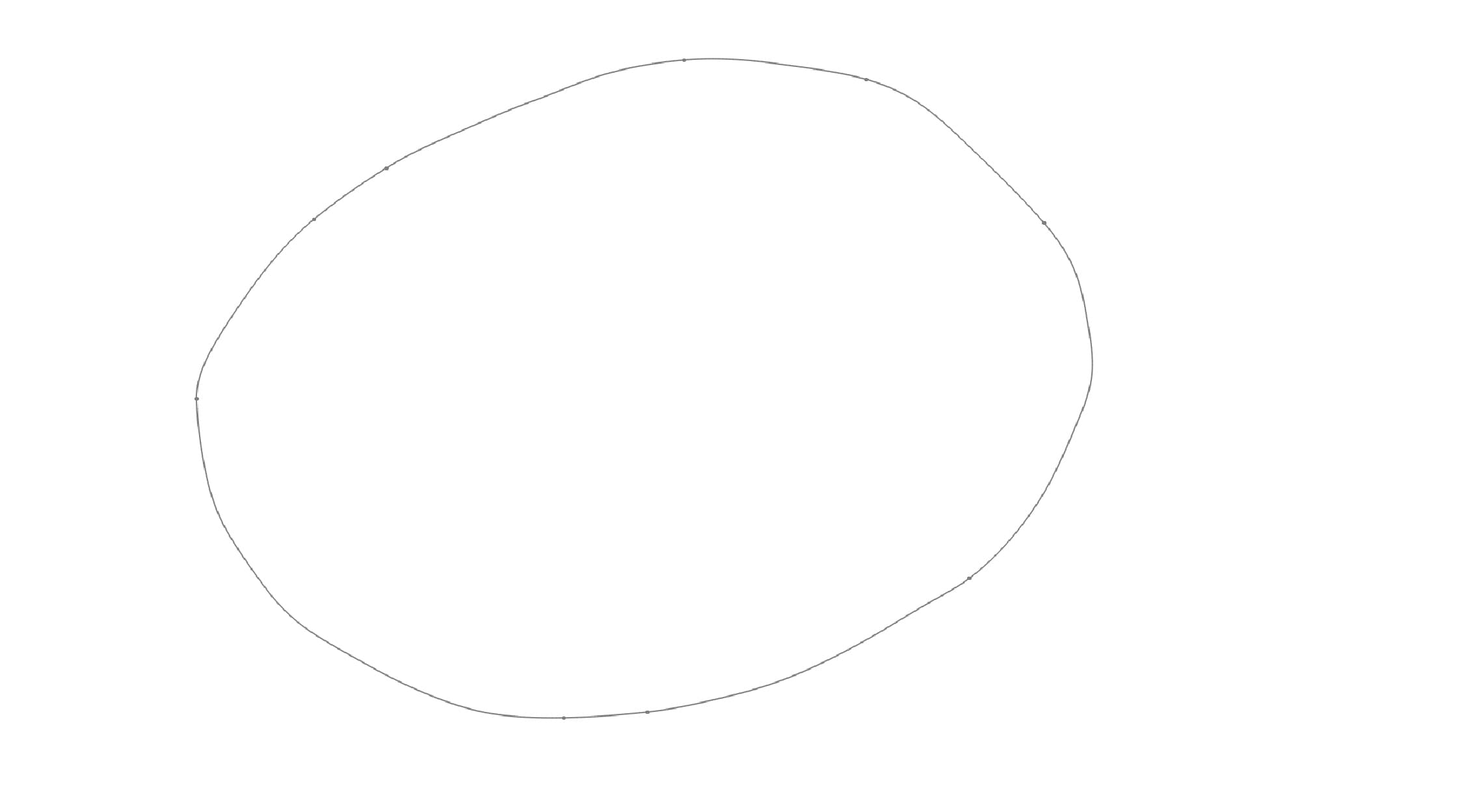

Supplement: Supplementary file 4 — Supporting Information [file ADVS-10-2203062-s013.zip › advs202203062-sup-0004-Supplementary-DataS3/Supplementary Data S3/13.jpg]

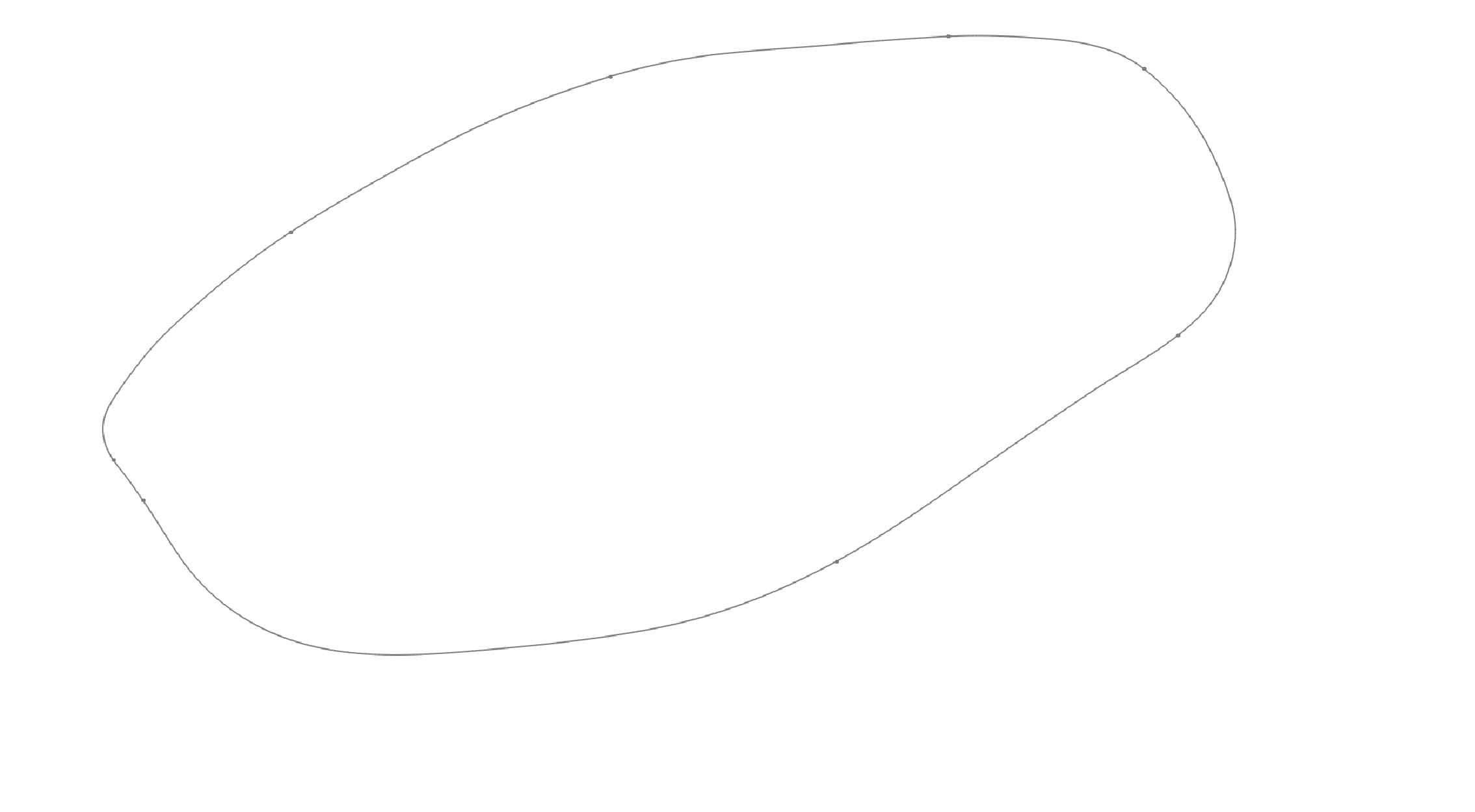

Supplement: Supplementary file 4 — Supporting Information [file ADVS-10-2203062-s013.zip › advs202203062-sup-0004-Supplementary-DataS3/Supplementary Data S3/130.jpg]

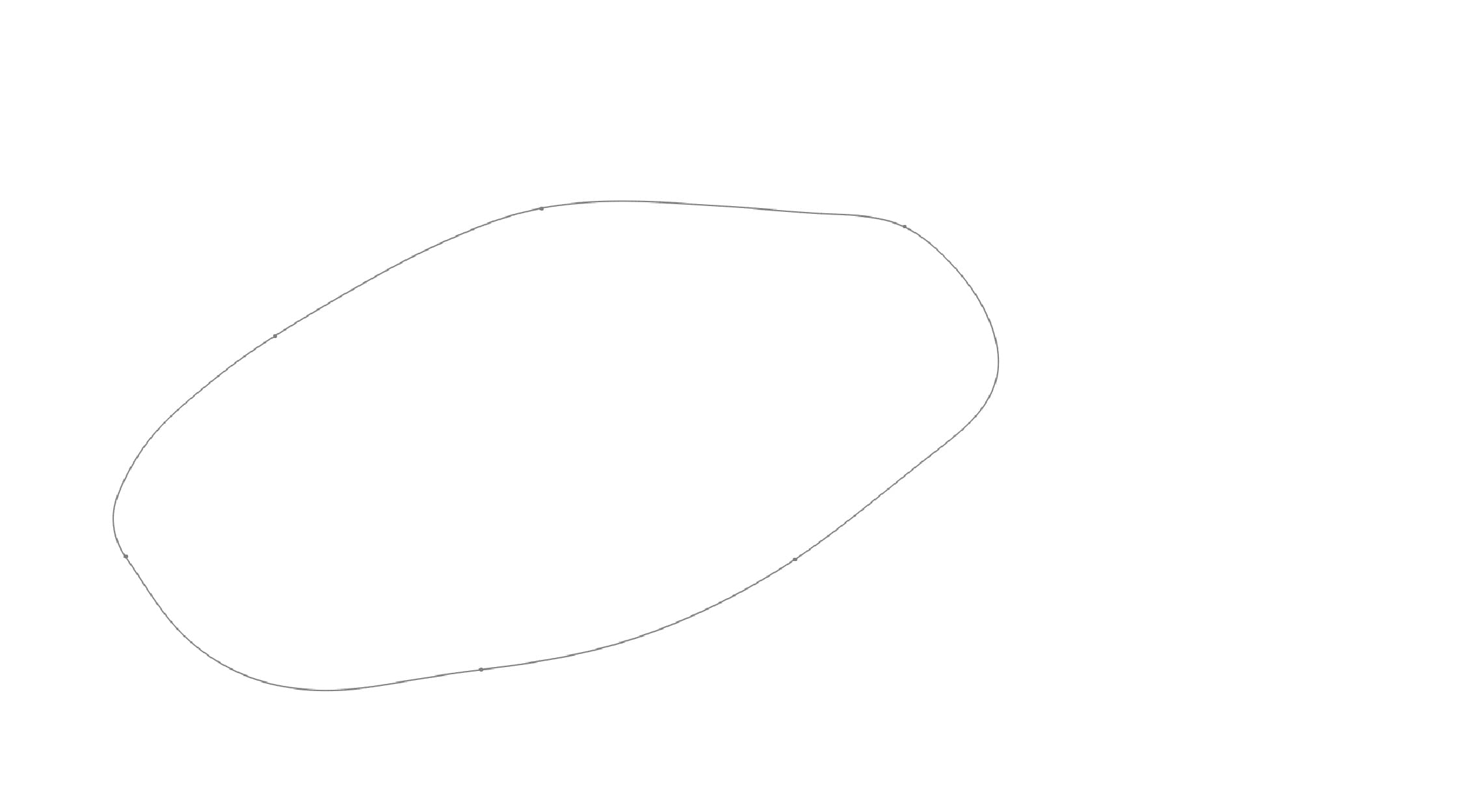

Supplement: Supplementary file 4 — Supporting Information [file ADVS-10-2203062-s013.zip › advs202203062-sup-0004-Supplementary-DataS3/Supplementary Data S3/131.jpg]

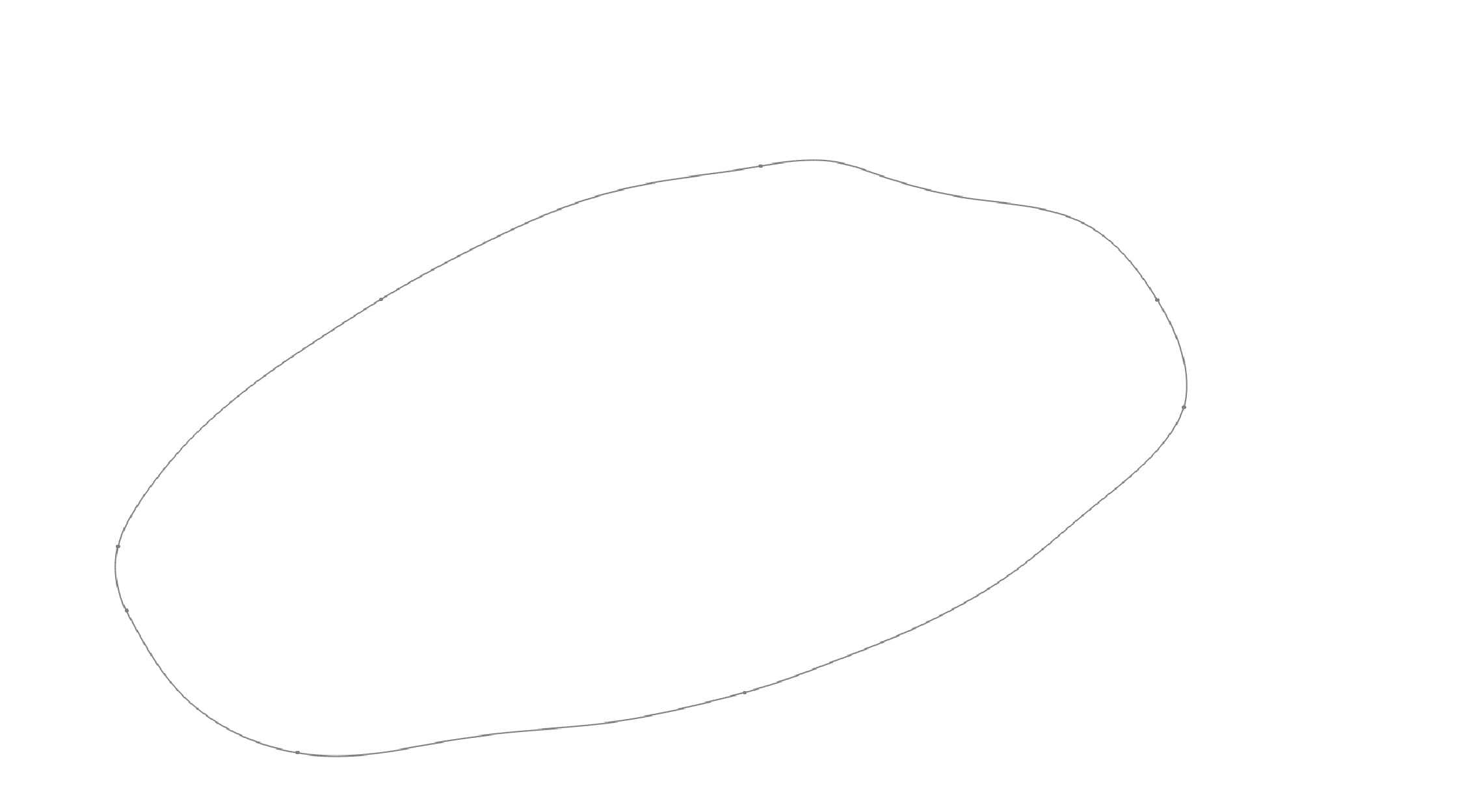

Supplement: Supplementary file 4 — Supporting Information [file ADVS-10-2203062-s013.zip › advs202203062-sup-0004-Supplementary-DataS3/Supplementary Data S3/132.jpg]

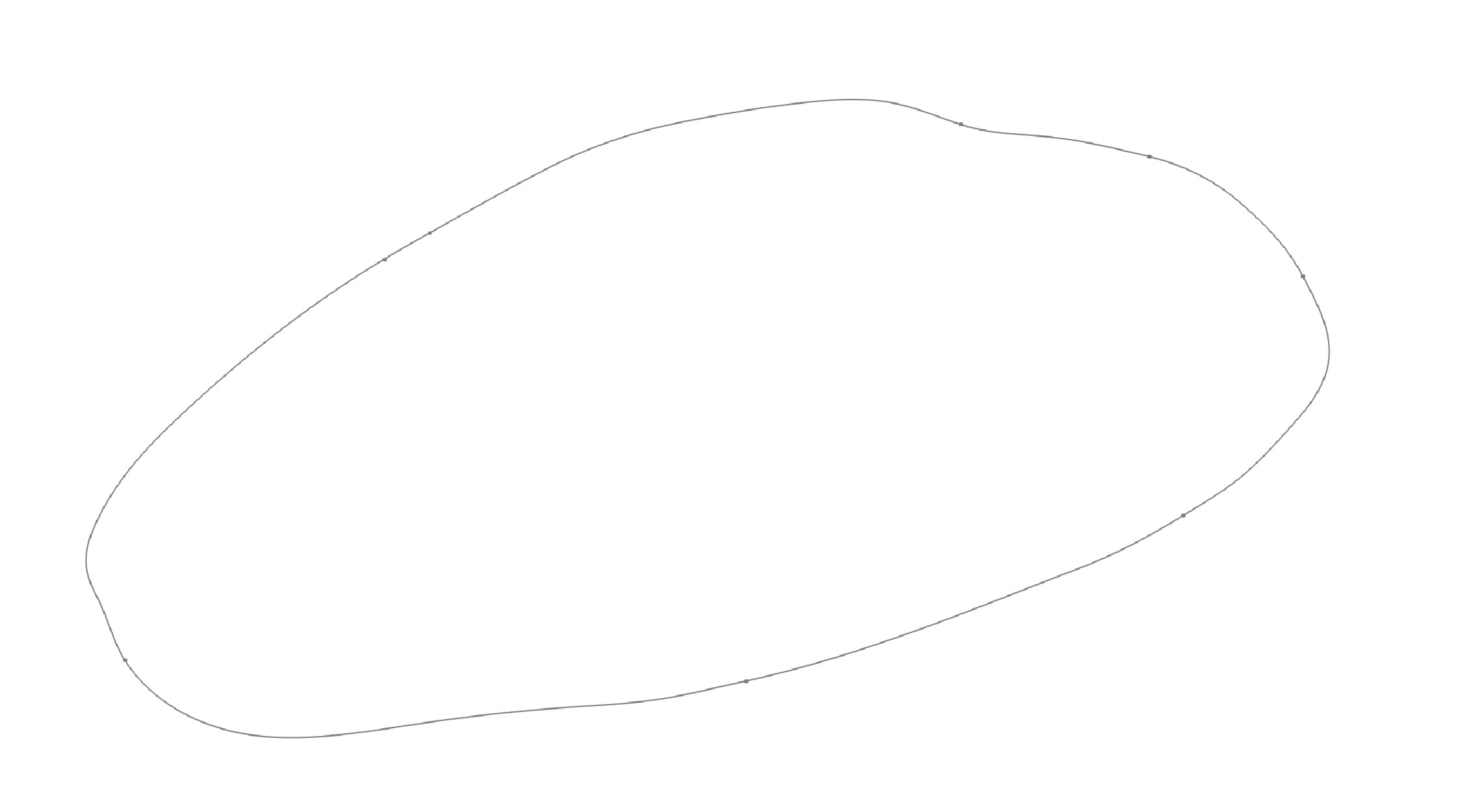

Supplement: Supplementary file 4 — Supporting Information [file ADVS-10-2203062-s013.zip › advs202203062-sup-0004-Supplementary-DataS3/Supplementary Data S3/133.jpg]

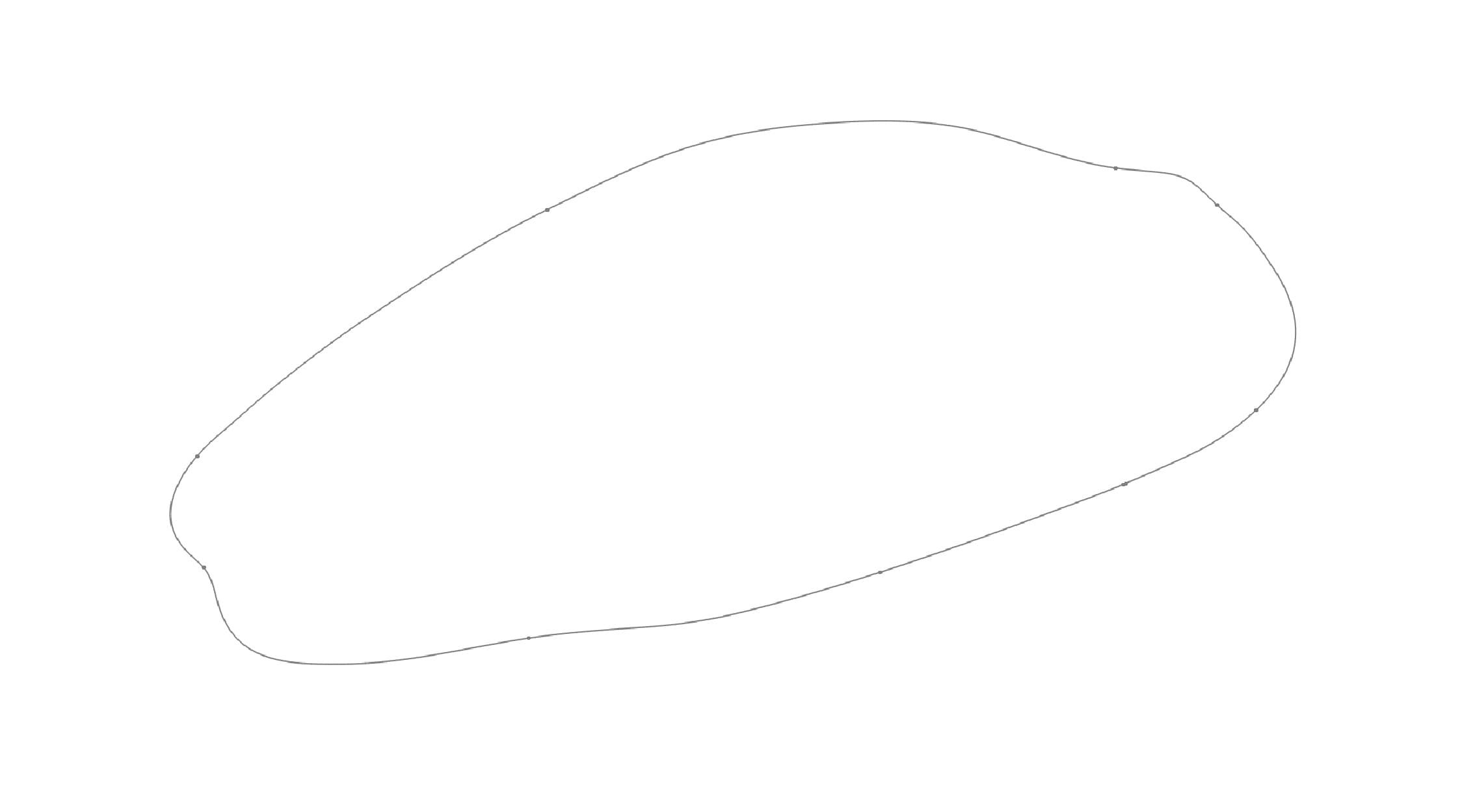

Supplement: Supplementary file 4 — Supporting Information [file ADVS-10-2203062-s013.zip › advs202203062-sup-0004-Supplementary-DataS3/Supplementary Data S3/134.jpg]

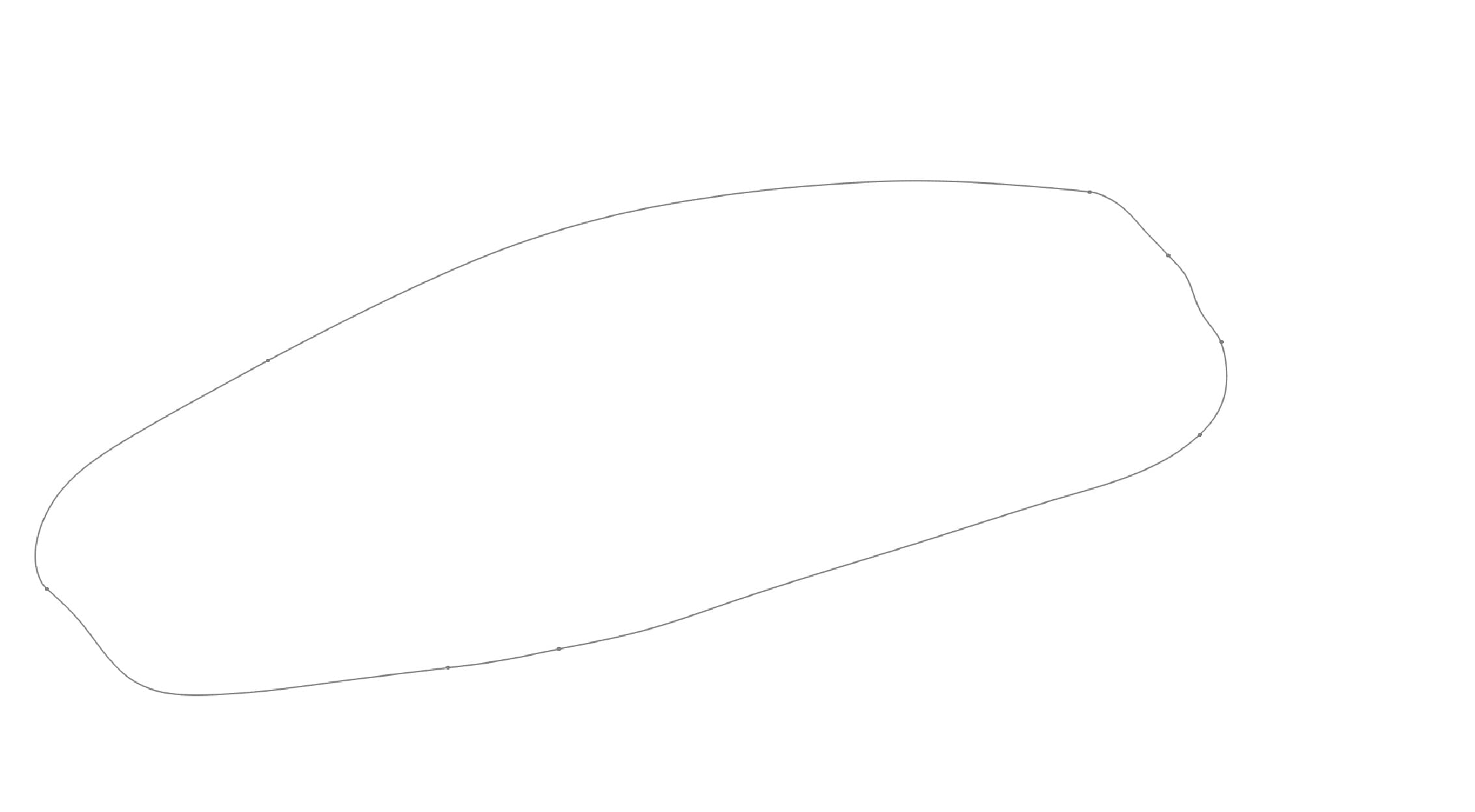

Supplement: Supplementary file 4 — Supporting Information [file ADVS-10-2203062-s013.zip › advs202203062-sup-0004-Supplementary-DataS3/Supplementary Data S3/135.jpg]

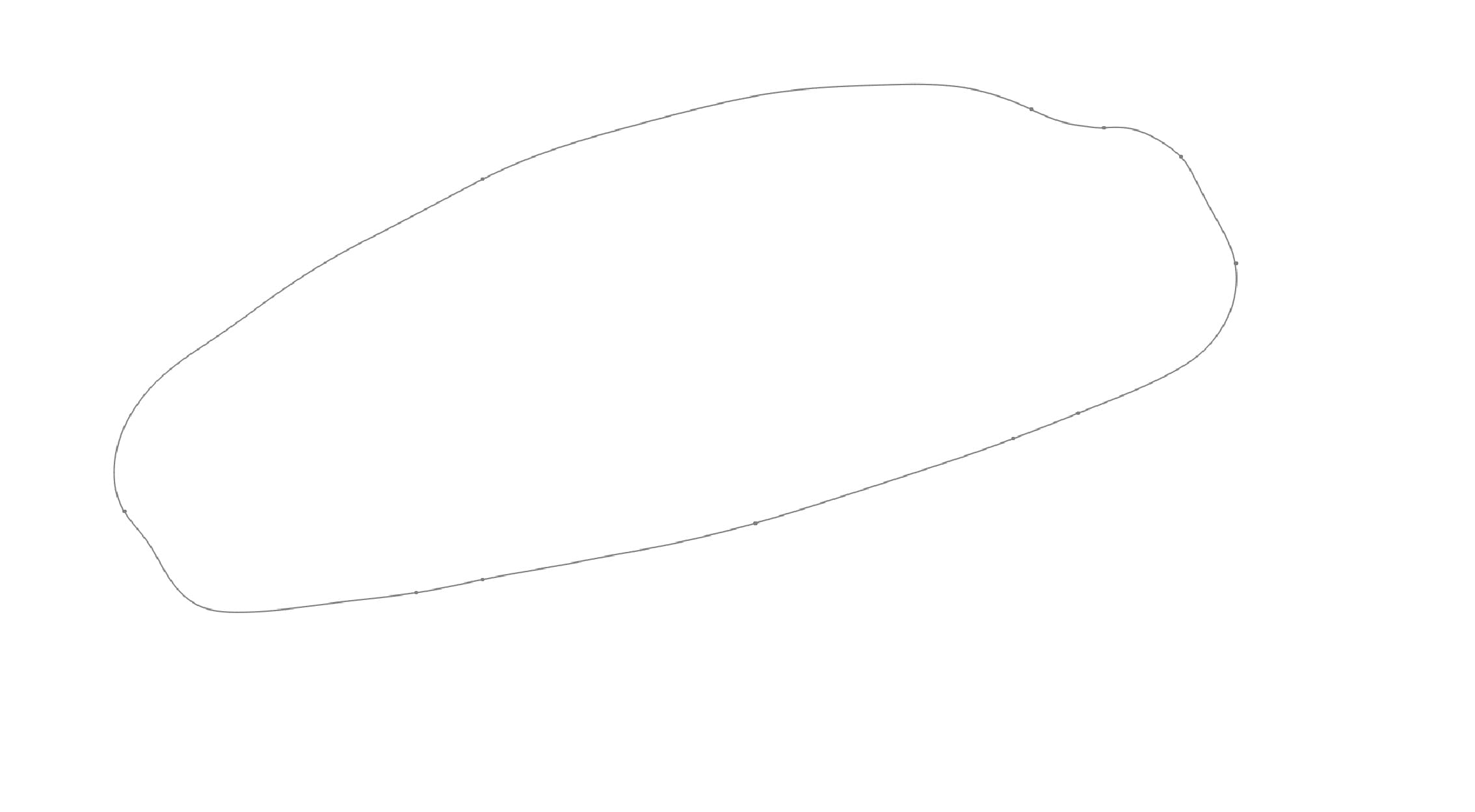

Supplement: Supplementary file 4 — Supporting Information [file ADVS-10-2203062-s013.zip › advs202203062-sup-0004-Supplementary-DataS3/Supplementary Data S3/136.jpg]

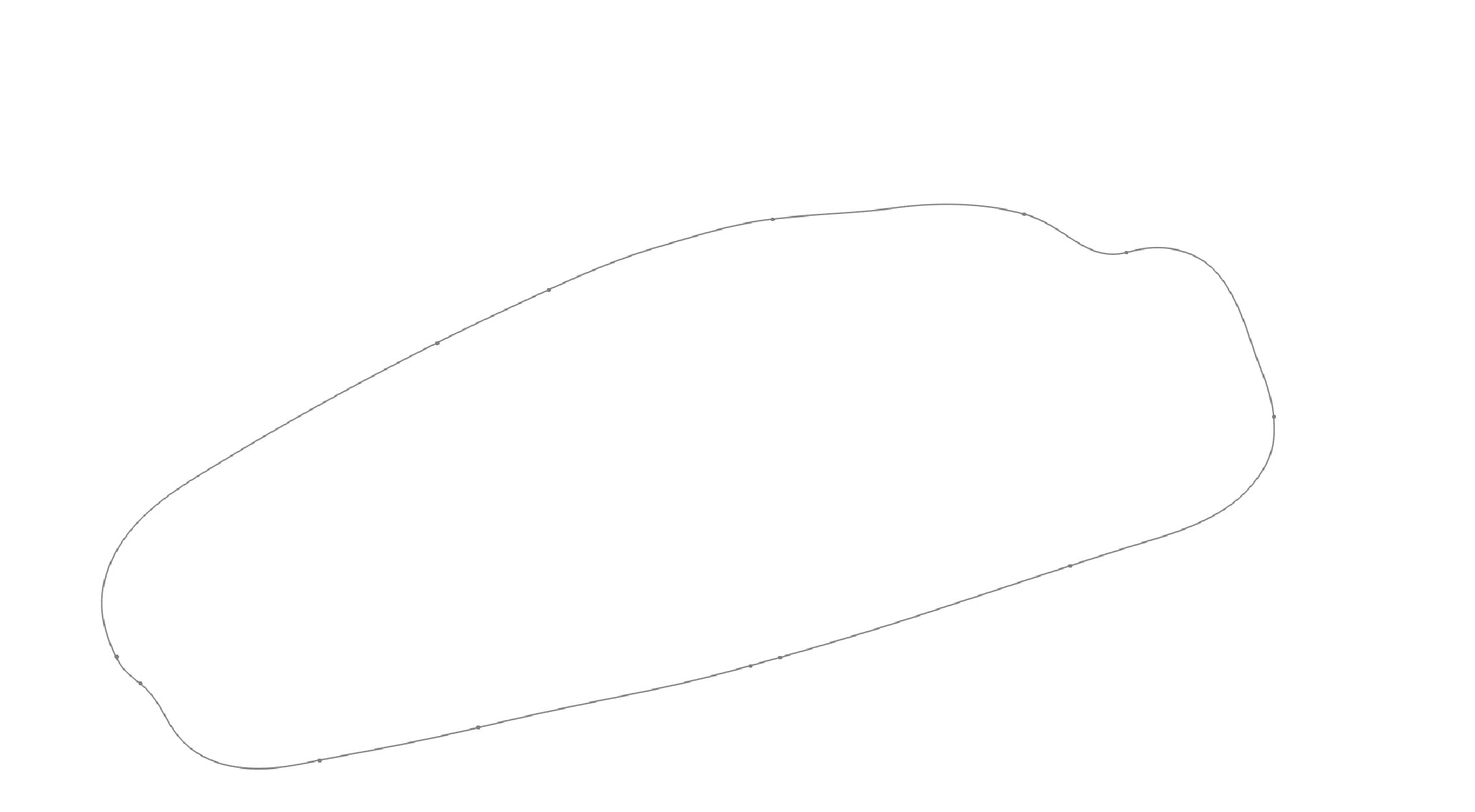

Supplement: Supplementary file 4 — Supporting Information [file ADVS-10-2203062-s013.zip › advs202203062-sup-0004-Supplementary-DataS3/Supplementary Data S3/137.jpg]

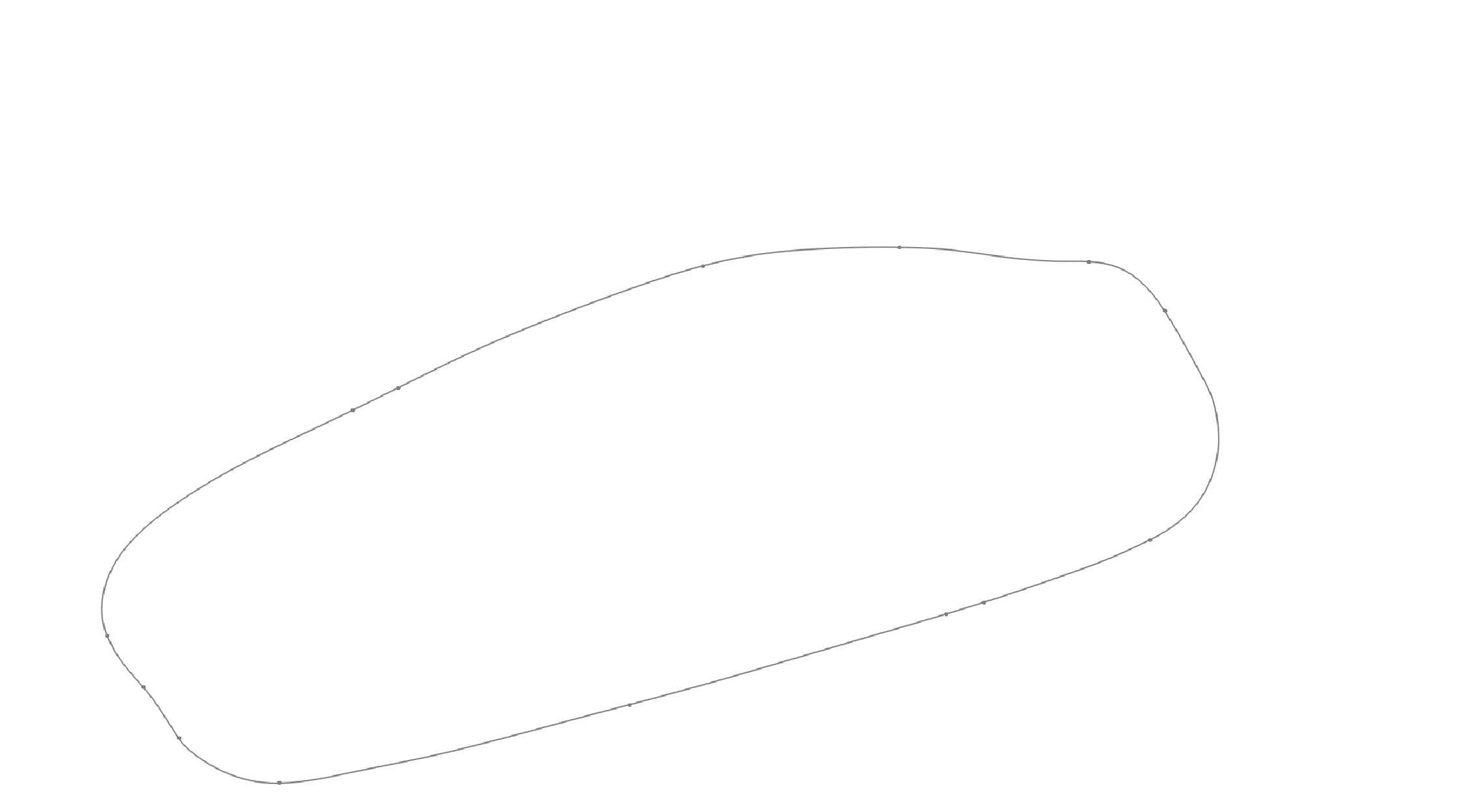

Supplement: Supplementary file 4 — Supporting Information [file ADVS-10-2203062-s013.zip › advs202203062-sup-0004-Supplementary-DataS3/Supplementary Data S3/138.jpg]

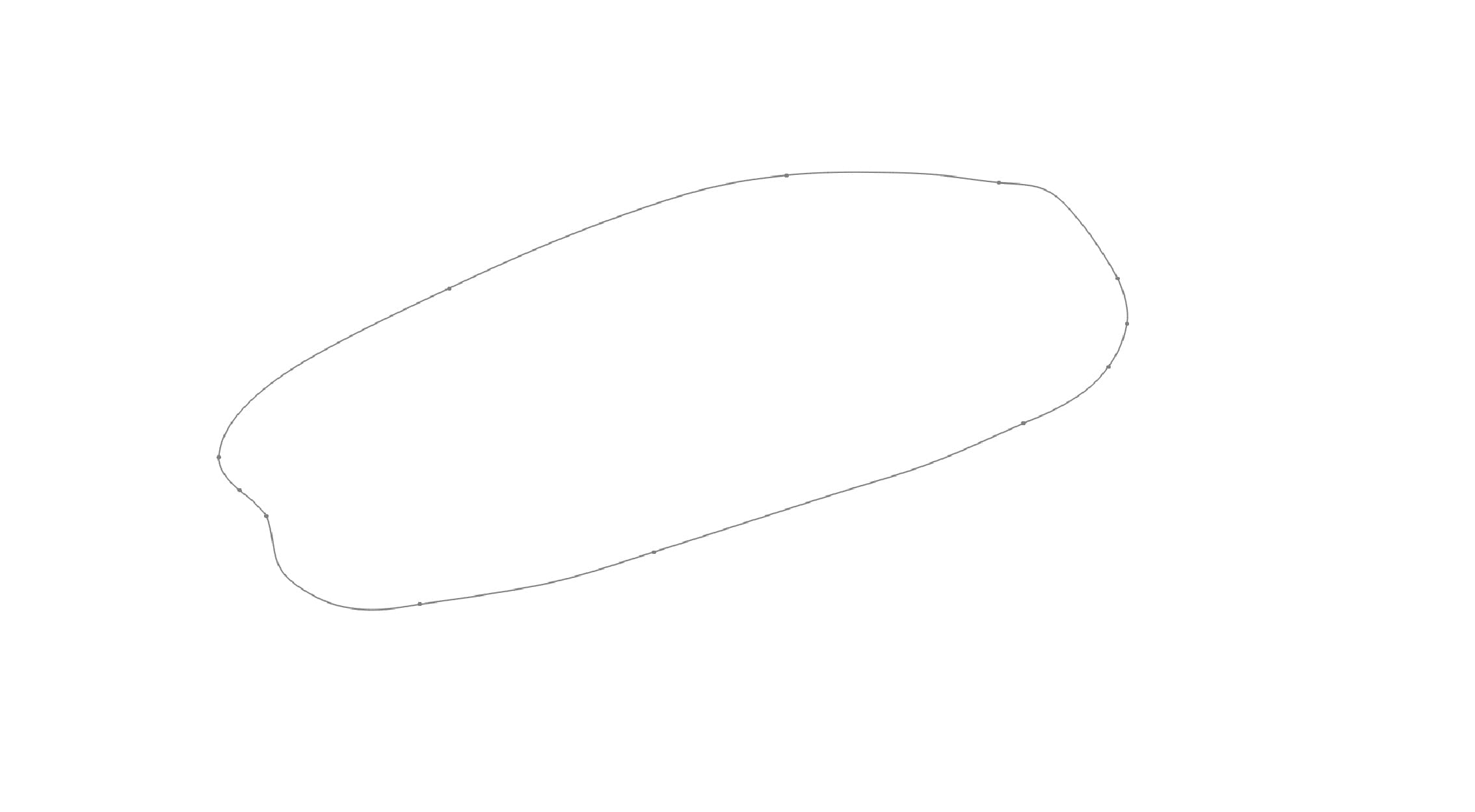

Supplement: Supplementary file 4 — Supporting Information [file ADVS-10-2203062-s013.zip › advs202203062-sup-0004-Supplementary-DataS3/Supplementary Data S3/139.jpg]

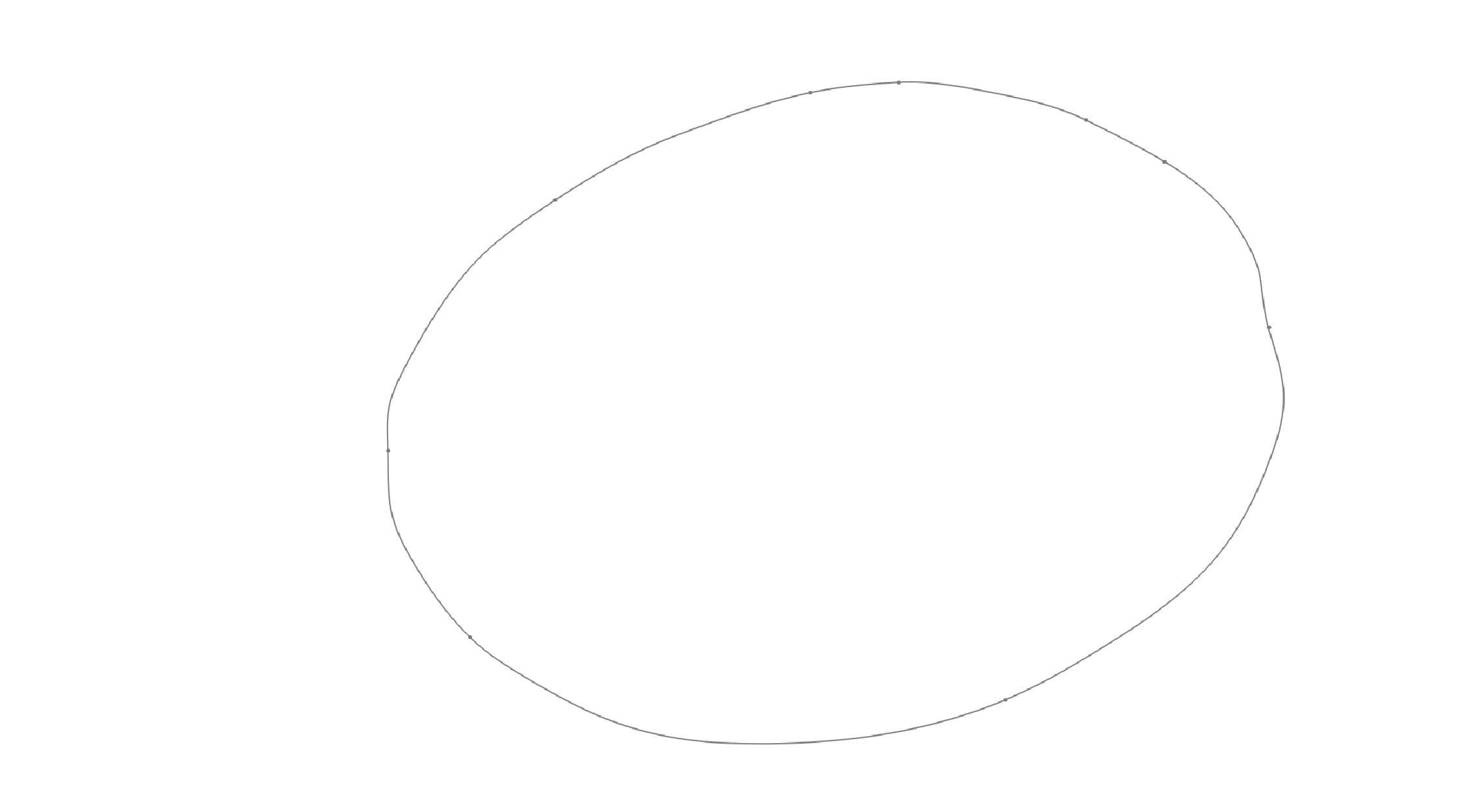

Supplement: Supplementary file 4 — Supporting Information [file ADVS-10-2203062-s013.zip › advs202203062-sup-0004-Supplementary-DataS3/Supplementary Data S3/14.jpg]

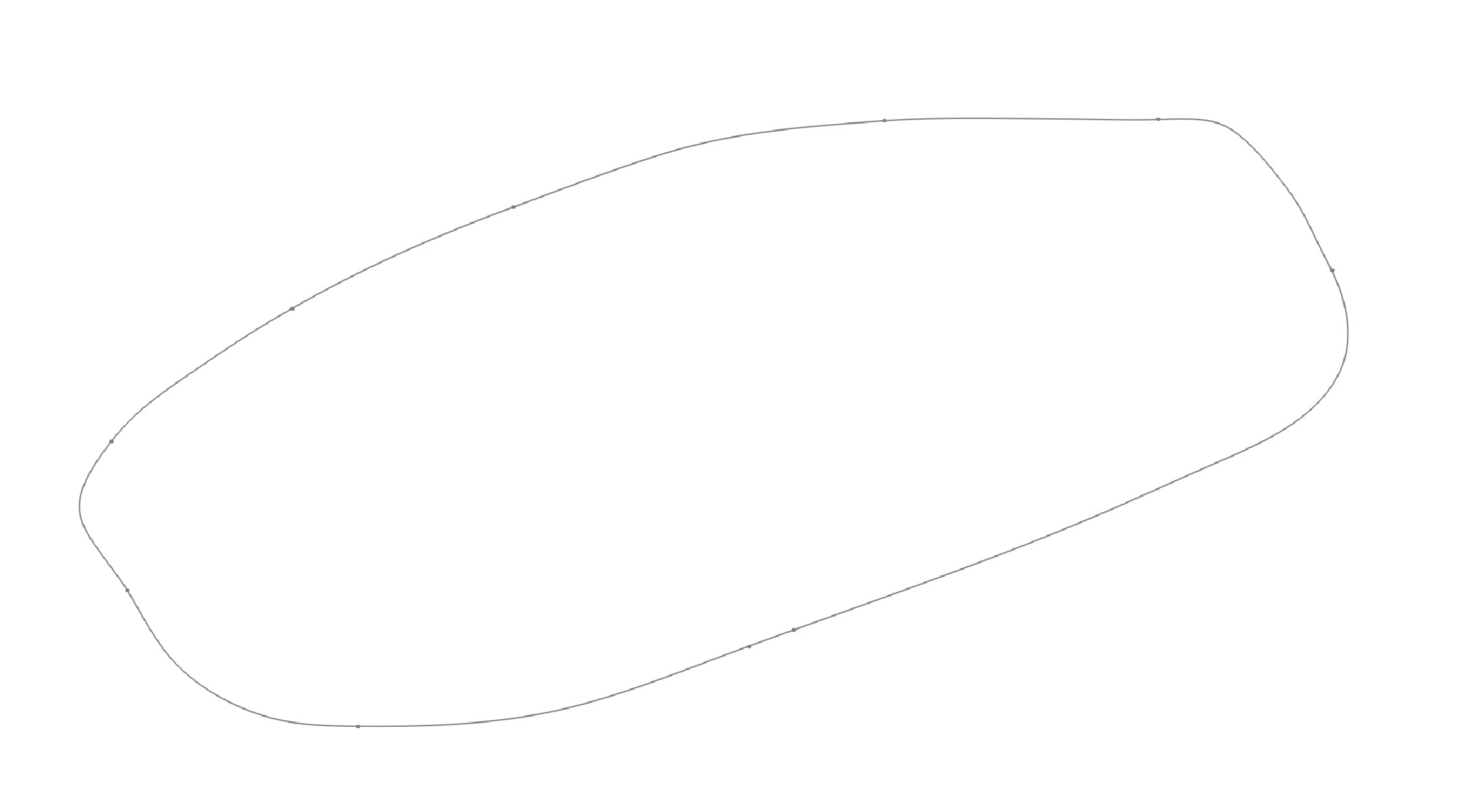

Supplement: Supplementary file 4 — Supporting Information [file ADVS-10-2203062-s013.zip › advs202203062-sup-0004-Supplementary-DataS3/Supplementary Data S3/140.jpg]

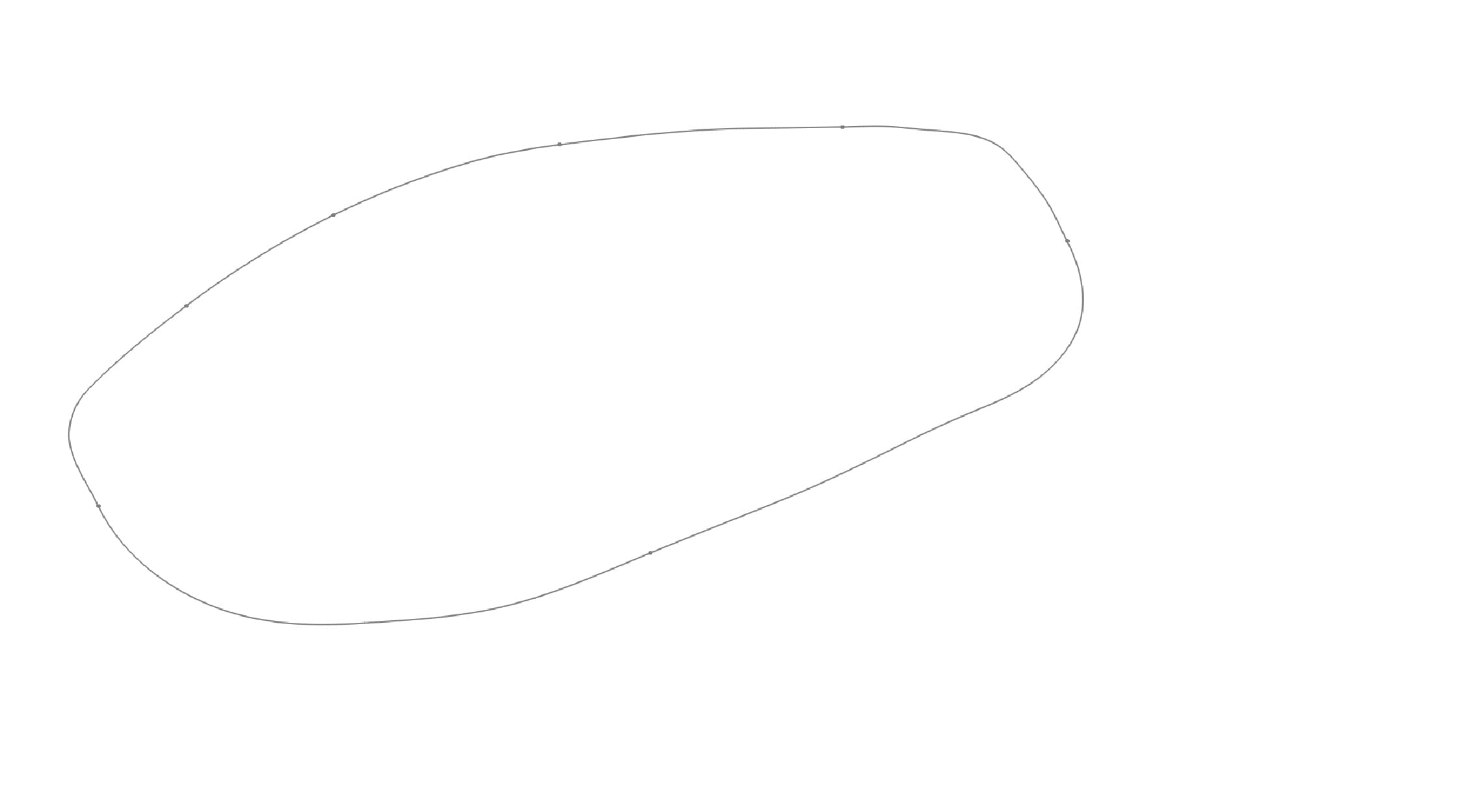

Supplement: Supplementary file 4 — Supporting Information [file ADVS-10-2203062-s013.zip › advs202203062-sup-0004-Supplementary-DataS3/Supplementary Data S3/141.jpg]

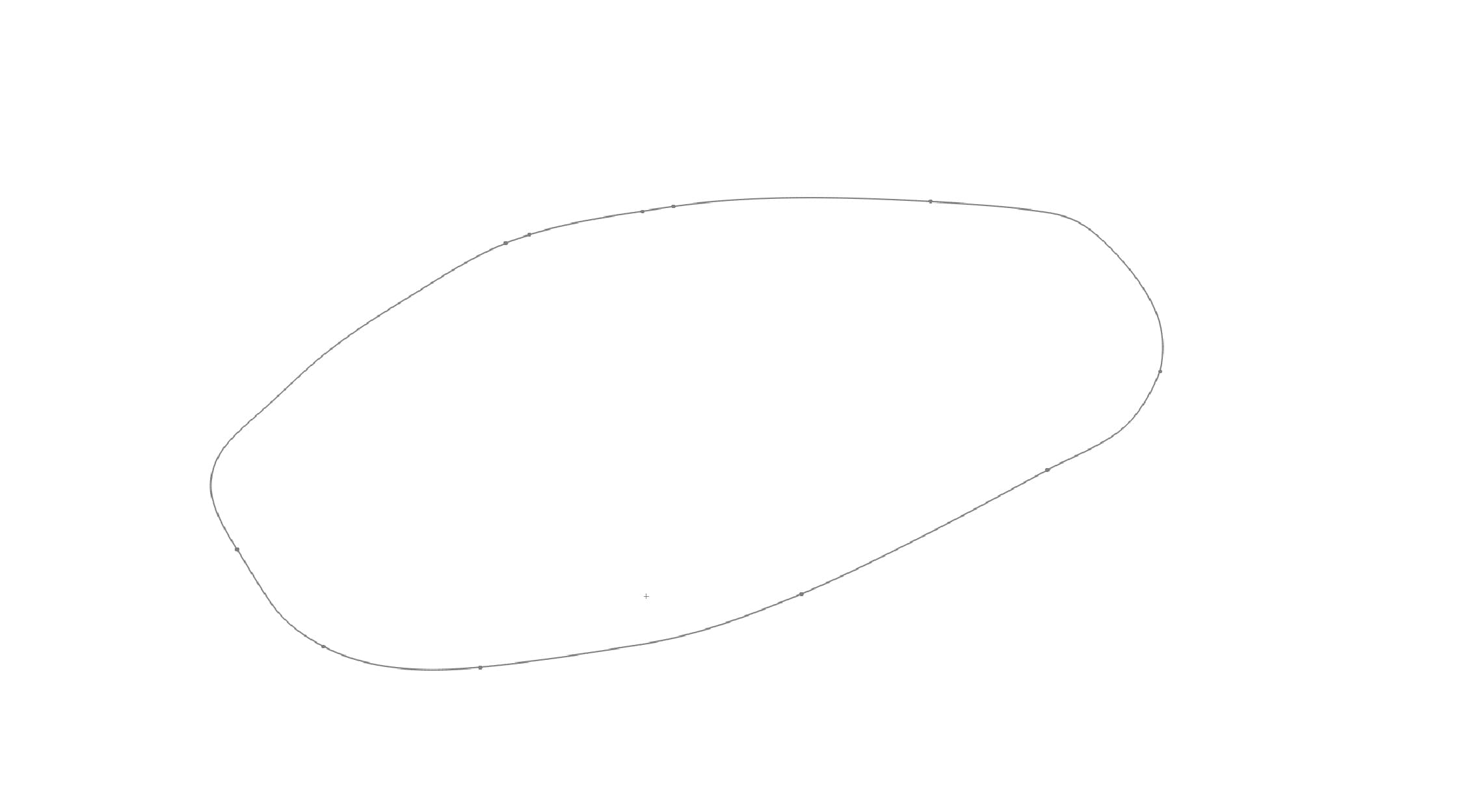

Supplement: Supplementary file 4 — Supporting Information [file ADVS-10-2203062-s013.zip › advs202203062-sup-0004-Supplementary-DataS3/Supplementary Data S3/142.jpg]

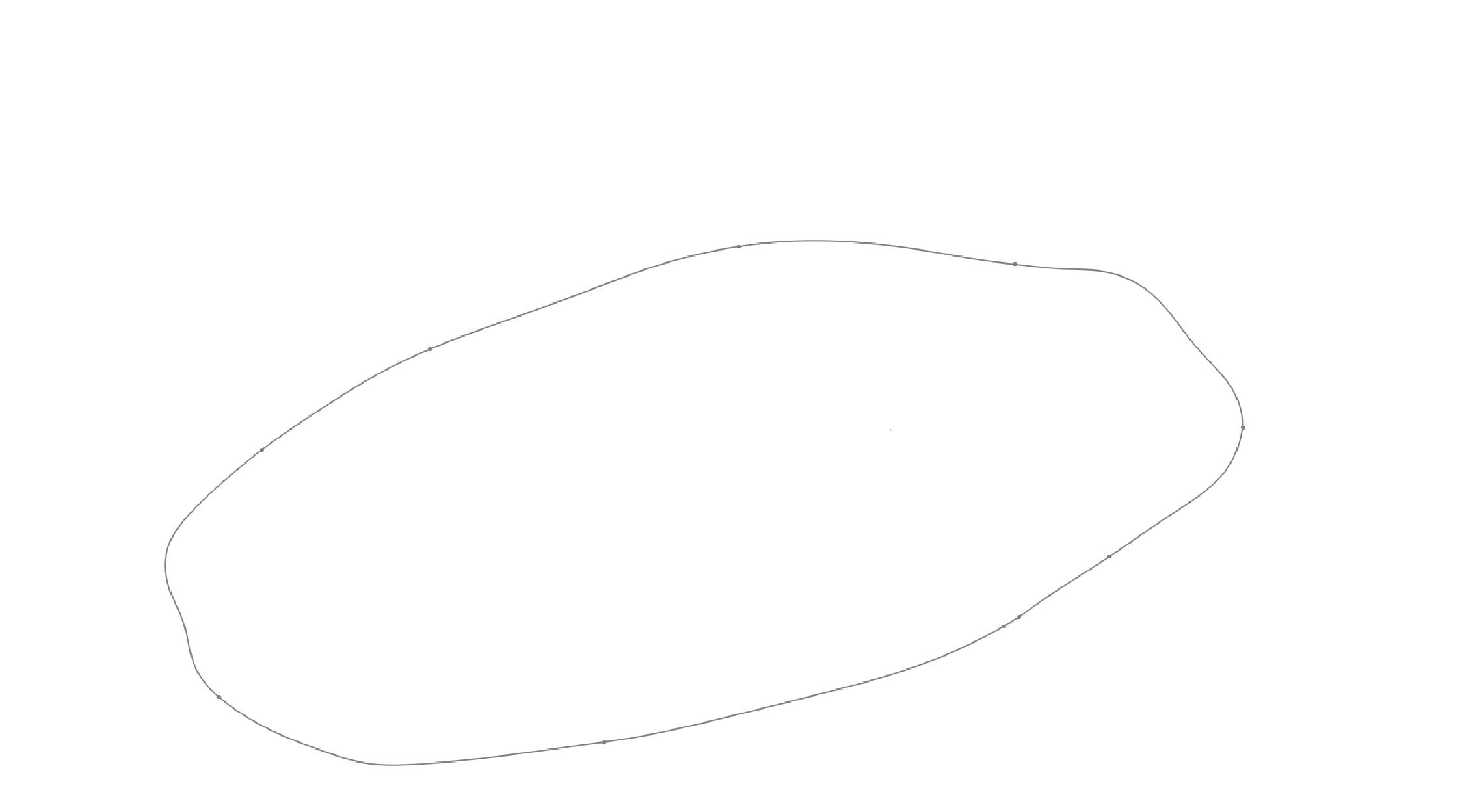

Supplement: Supplementary file 4 — Supporting Information [file ADVS-10-2203062-s013.zip › advs202203062-sup-0004-Supplementary-DataS3/Supplementary Data S3/143.jpg]

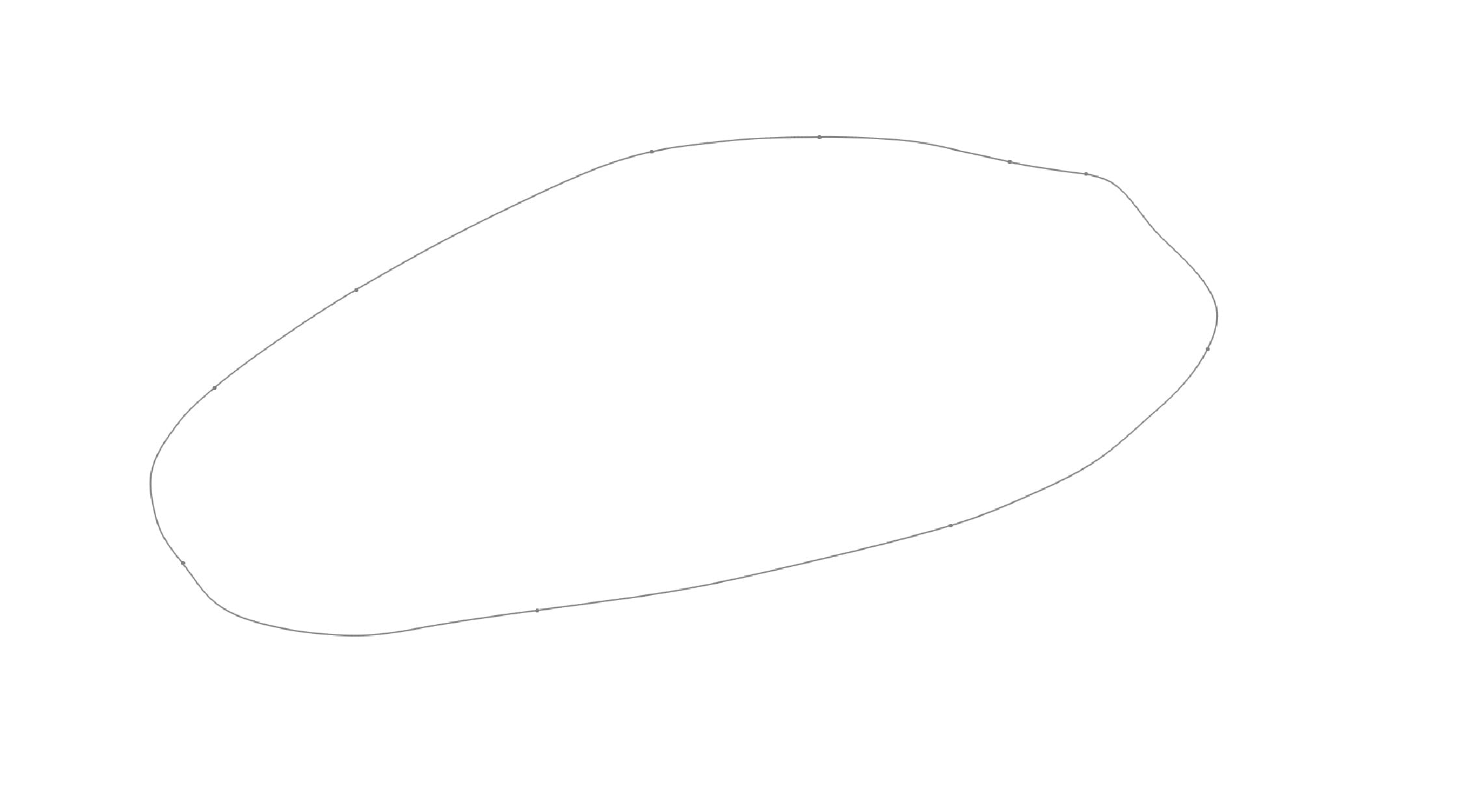

Supplement: Supplementary file 4 — Supporting Information [file ADVS-10-2203062-s013.zip › advs202203062-sup-0004-Supplementary-DataS3/Supplementary Data S3/144.jpg]

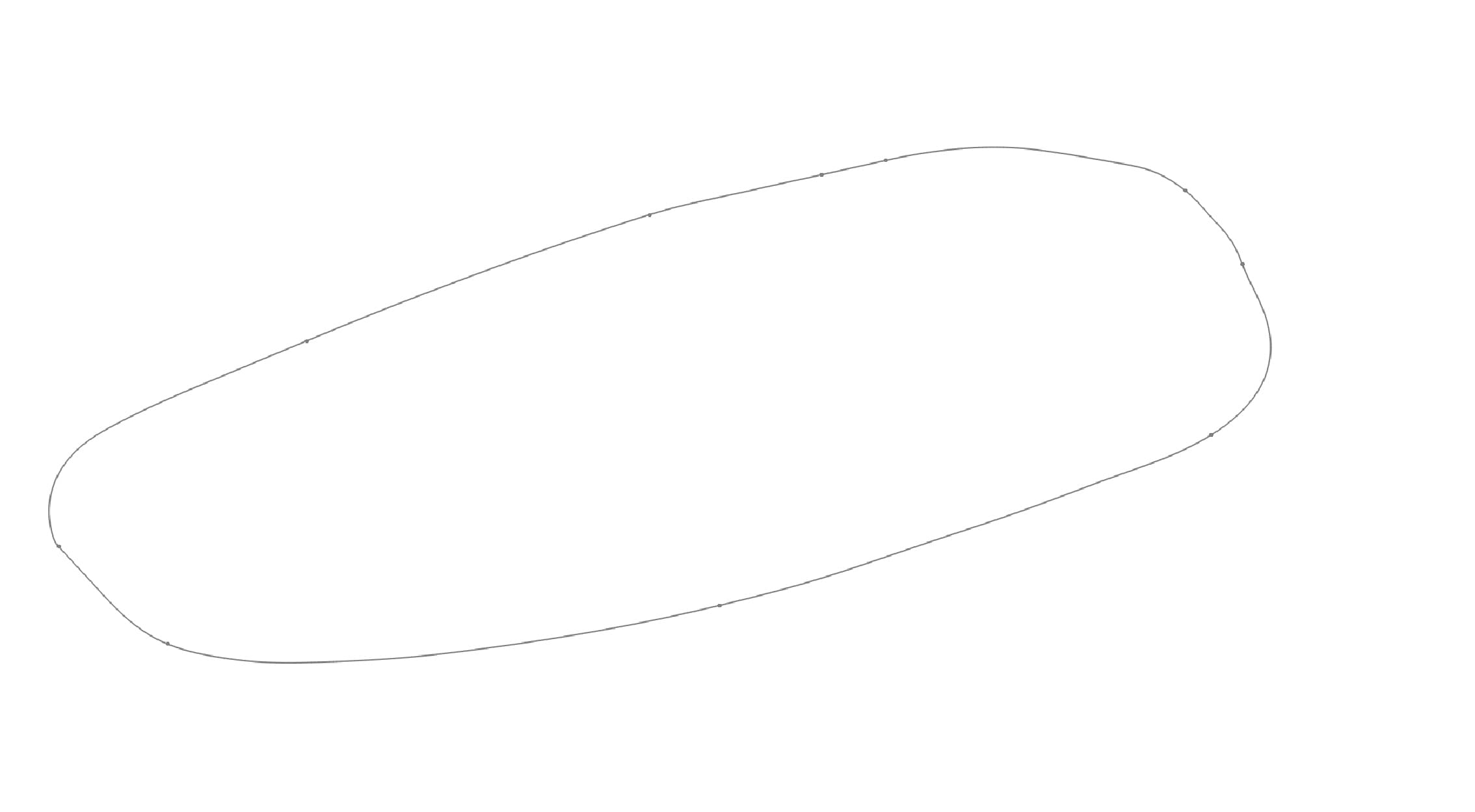

Supplement: Supplementary file 4 — Supporting Information [file ADVS-10-2203062-s013.zip › advs202203062-sup-0004-Supplementary-DataS3/Supplementary Data S3/145.jpg]

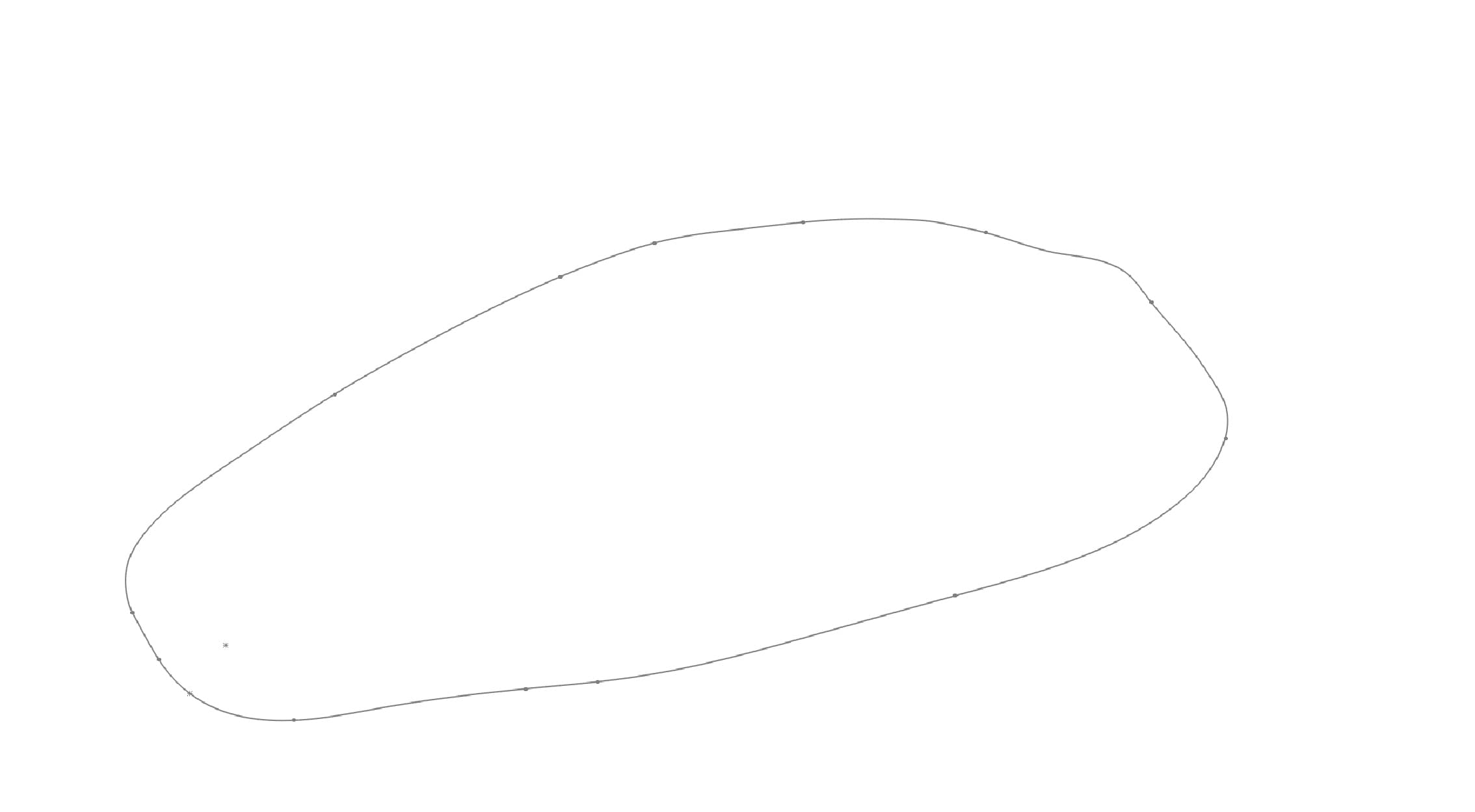

Supplement: Supplementary file 4 — Supporting Information [file ADVS-10-2203062-s013.zip › advs202203062-sup-0004-Supplementary-DataS3/Supplementary Data S3/146.jpg]

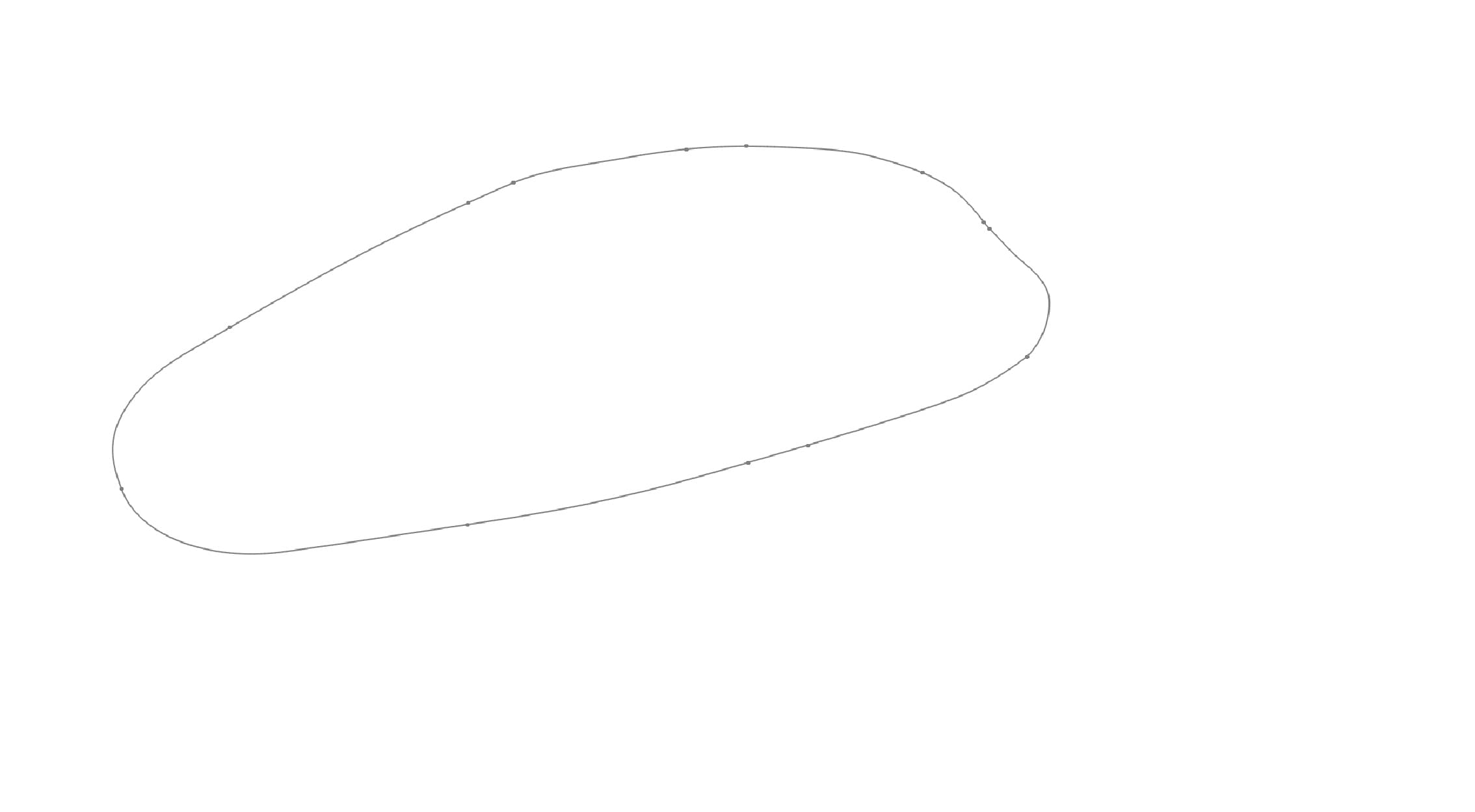

Supplement: Supplementary file 4 — Supporting Information [file ADVS-10-2203062-s013.zip › advs202203062-sup-0004-Supplementary-DataS3/Supplementary Data S3/147.jpg]

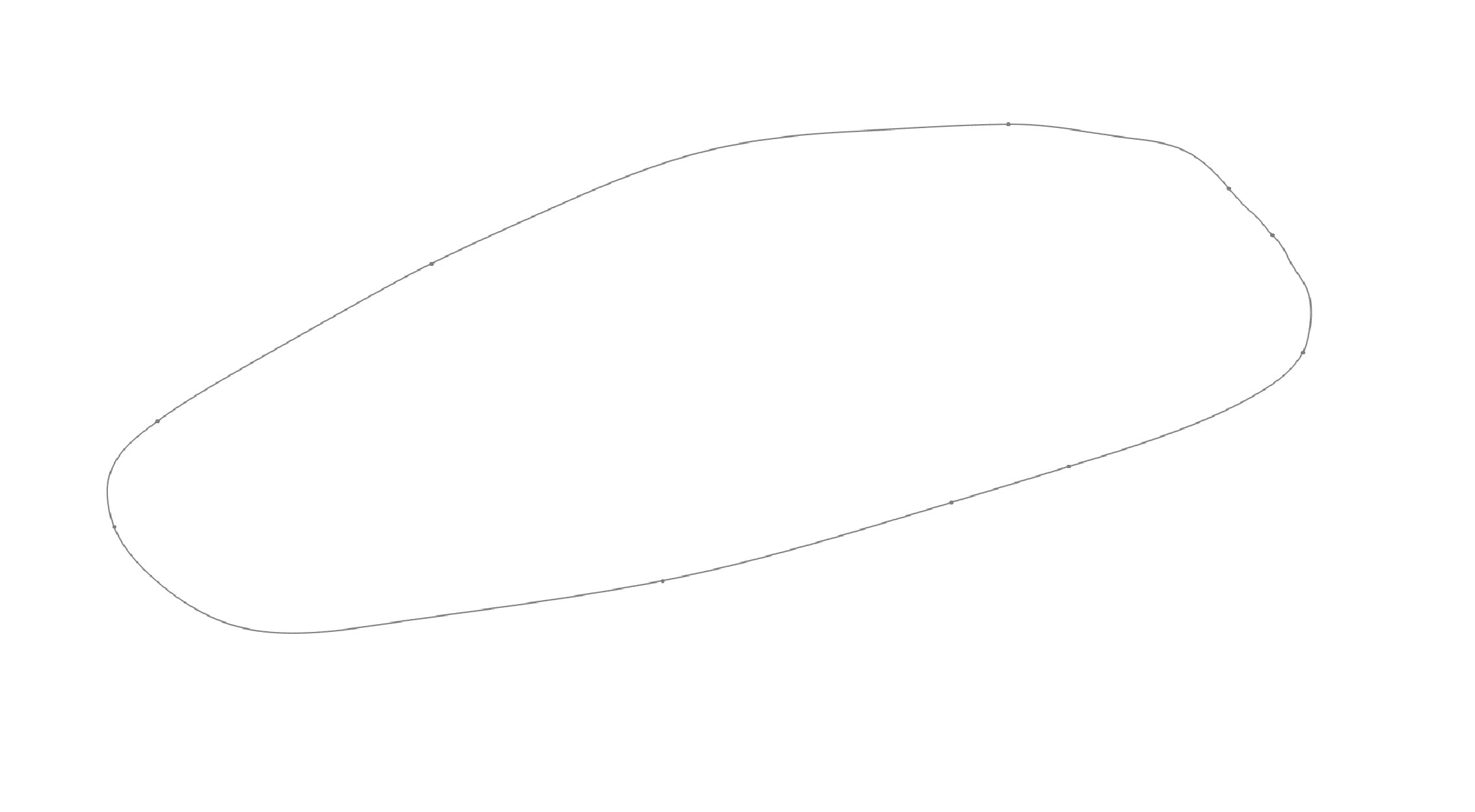

Supplement: Supplementary file 4 — Supporting Information [file ADVS-10-2203062-s013.zip › advs202203062-sup-0004-Supplementary-DataS3/Supplementary Data S3/148.jpg]

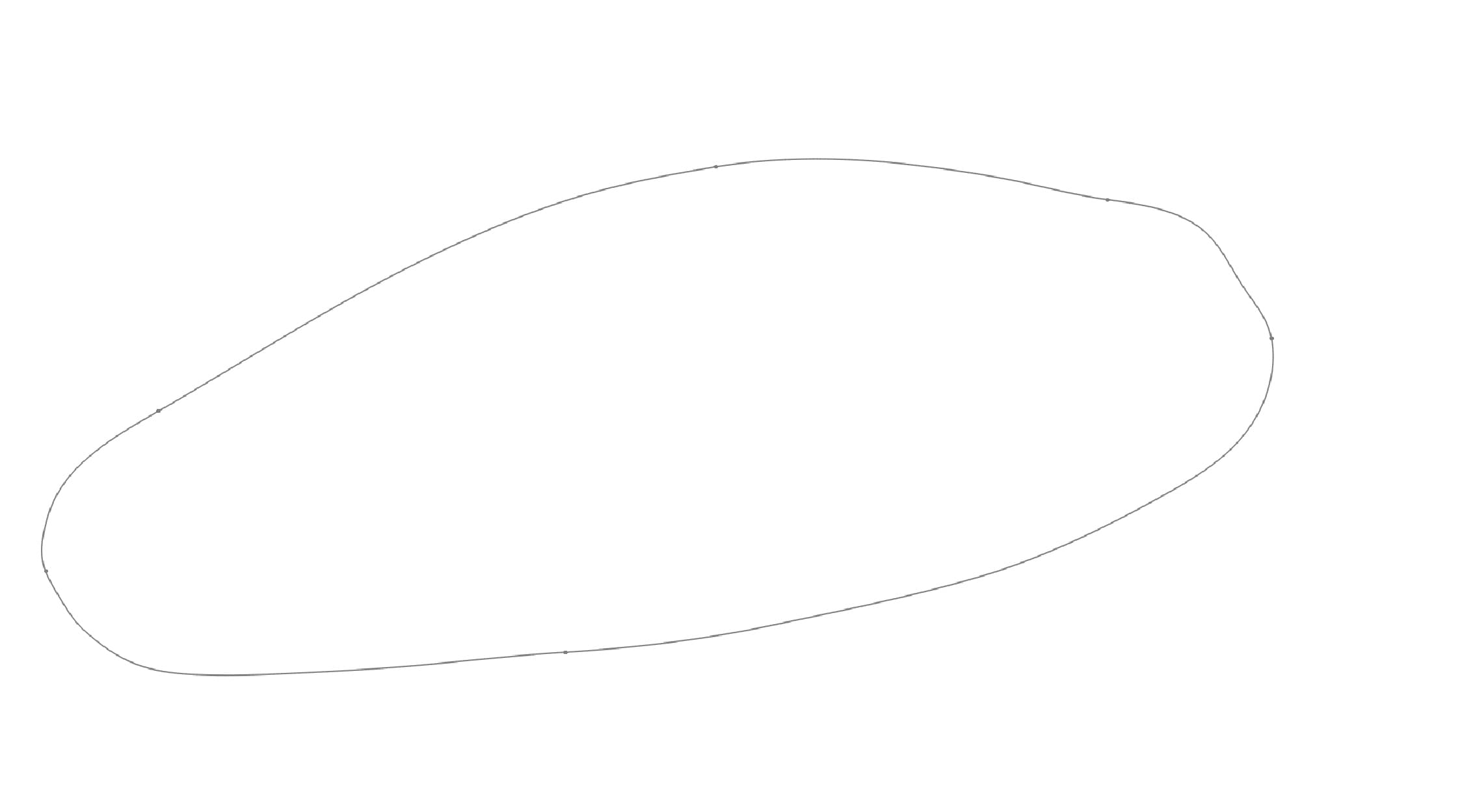

Supplement: Supplementary file 4 — Supporting Information [file ADVS-10-2203062-s013.zip › advs202203062-sup-0004-Supplementary-DataS3/Supplementary Data S3/149.jpg]

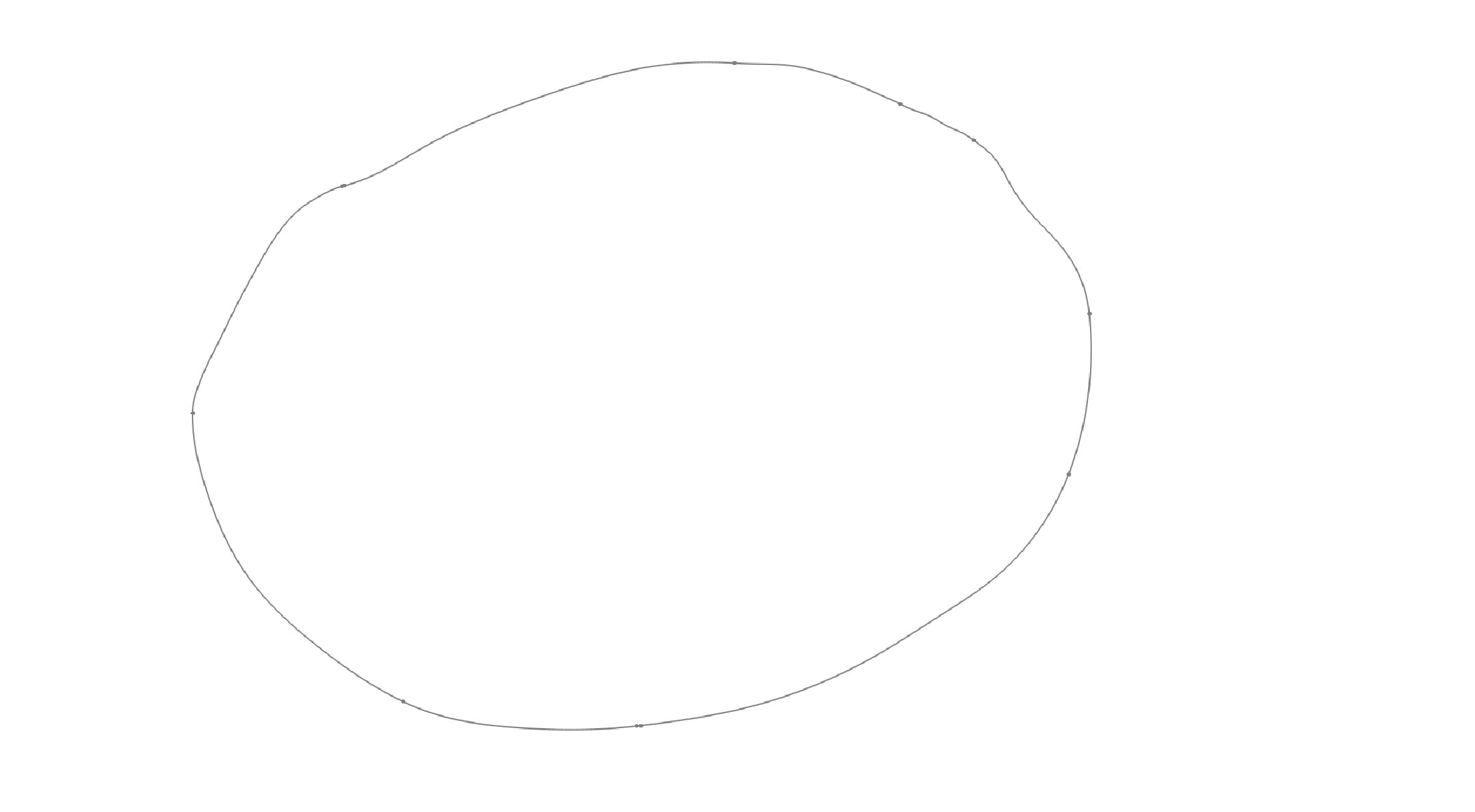

Supplement: Supplementary file 4 — Supporting Information [file ADVS-10-2203062-s013.zip › advs202203062-sup-0004-Supplementary-DataS3/Supplementary Data S3/15.jpg]

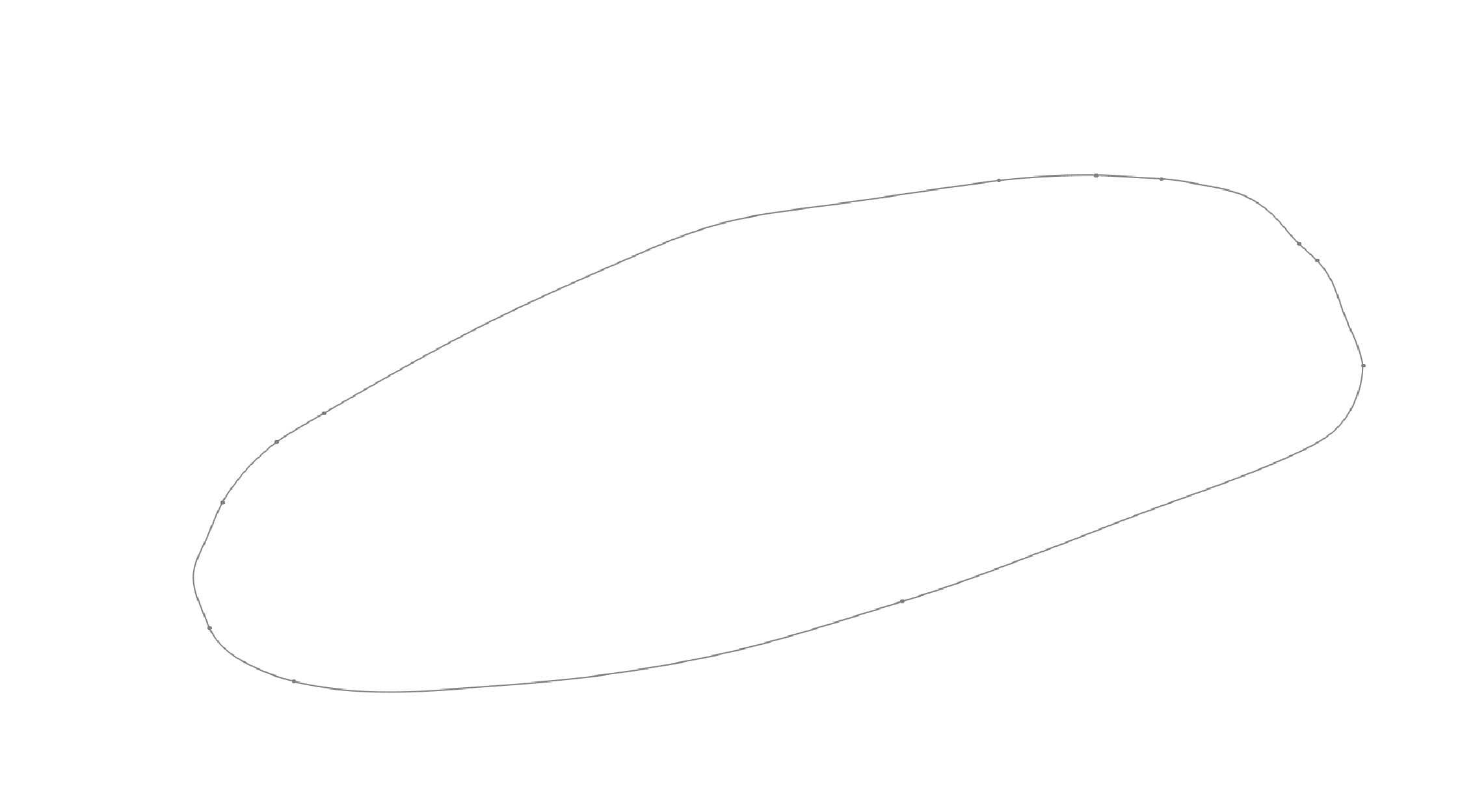

Supplement: Supplementary file 4 — Supporting Information [file ADVS-10-2203062-s013.zip › advs202203062-sup-0004-Supplementary-DataS3/Supplementary Data S3/150.jpg]

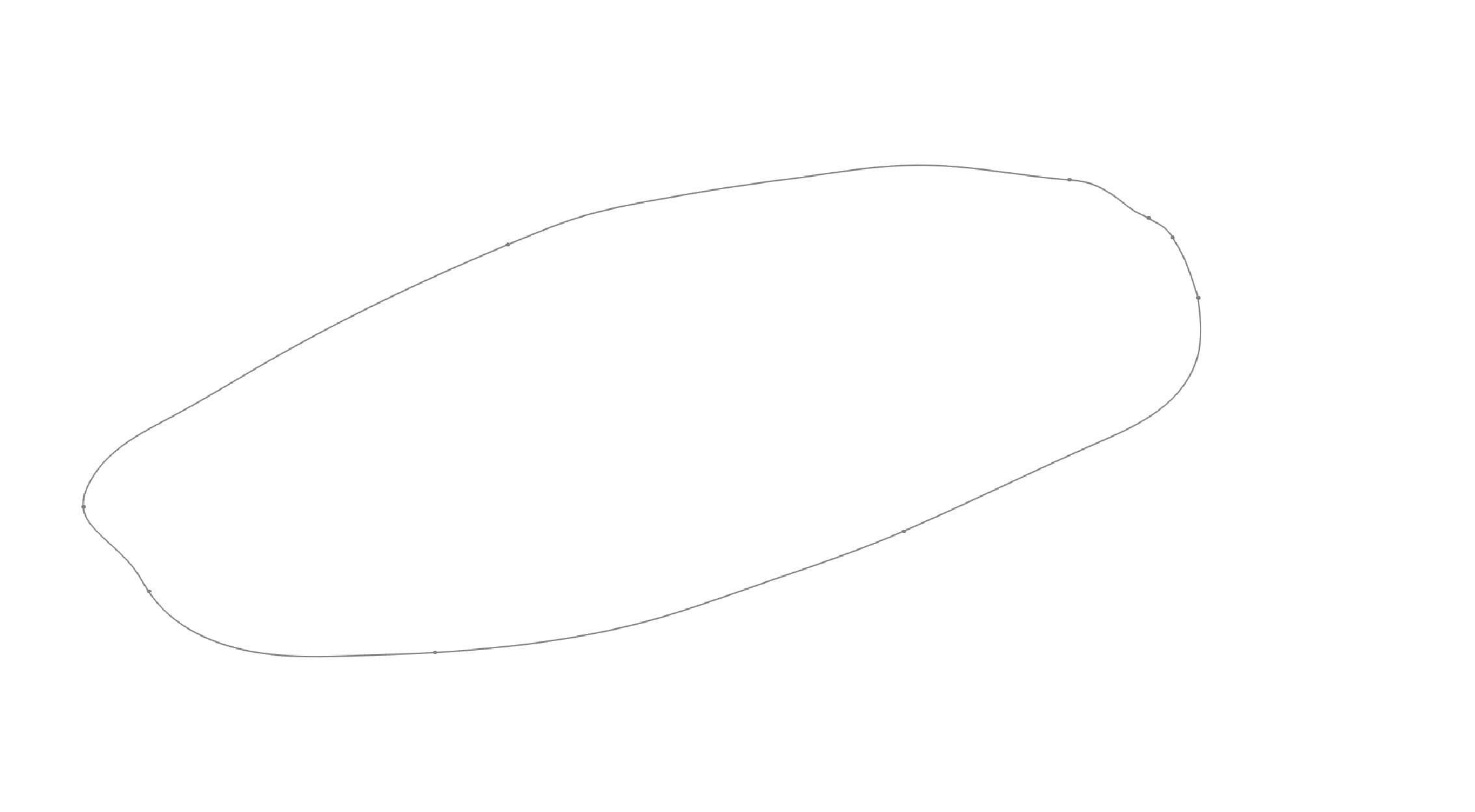

Supplement: Supplementary file 4 — Supporting Information [file ADVS-10-2203062-s013.zip › advs202203062-sup-0004-Supplementary-DataS3/Supplementary Data S3/151.jpg]

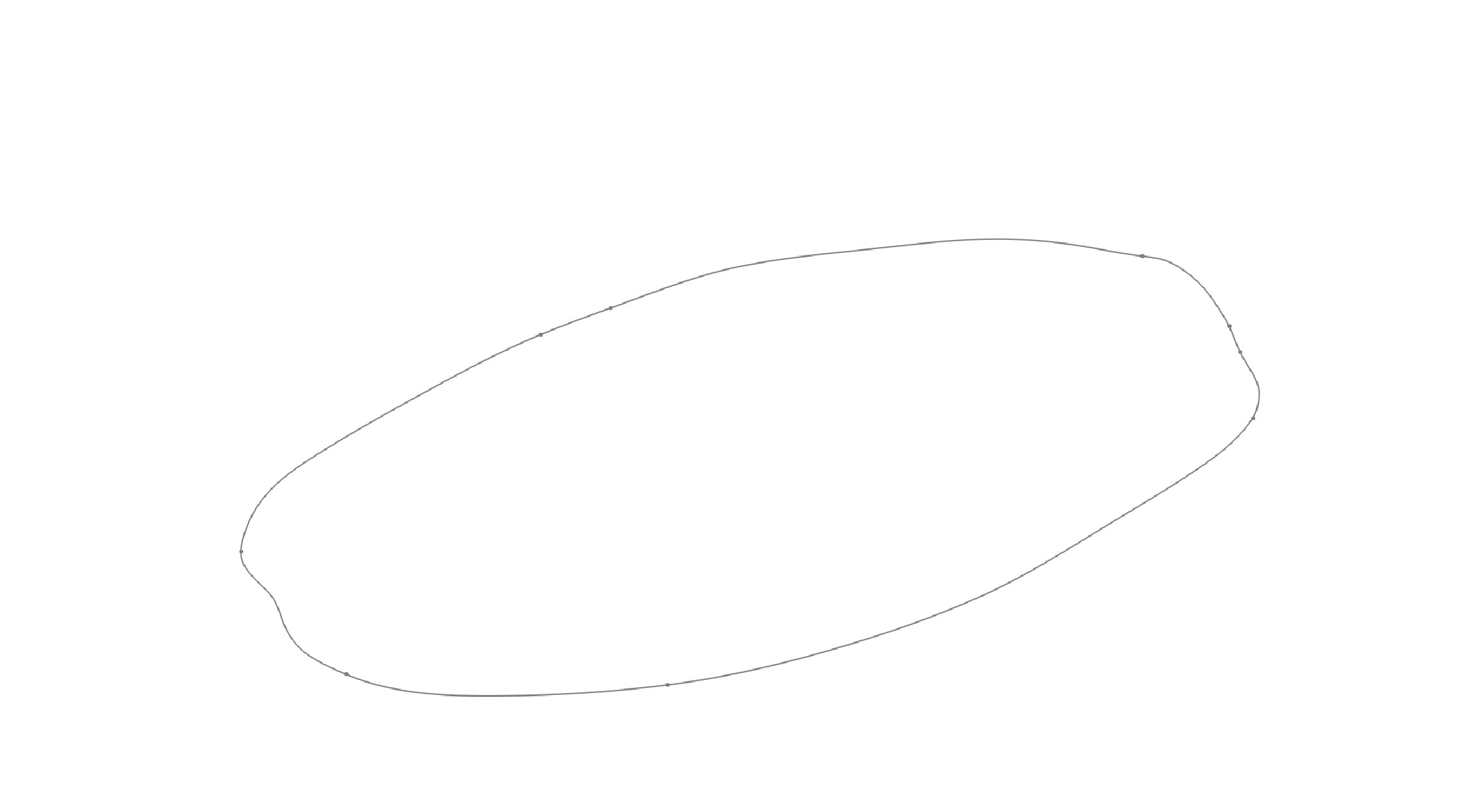

Supplement: Supplementary file 4 — Supporting Information [file ADVS-10-2203062-s013.zip › advs202203062-sup-0004-Supplementary-DataS3/Supplementary Data S3/152.jpg]

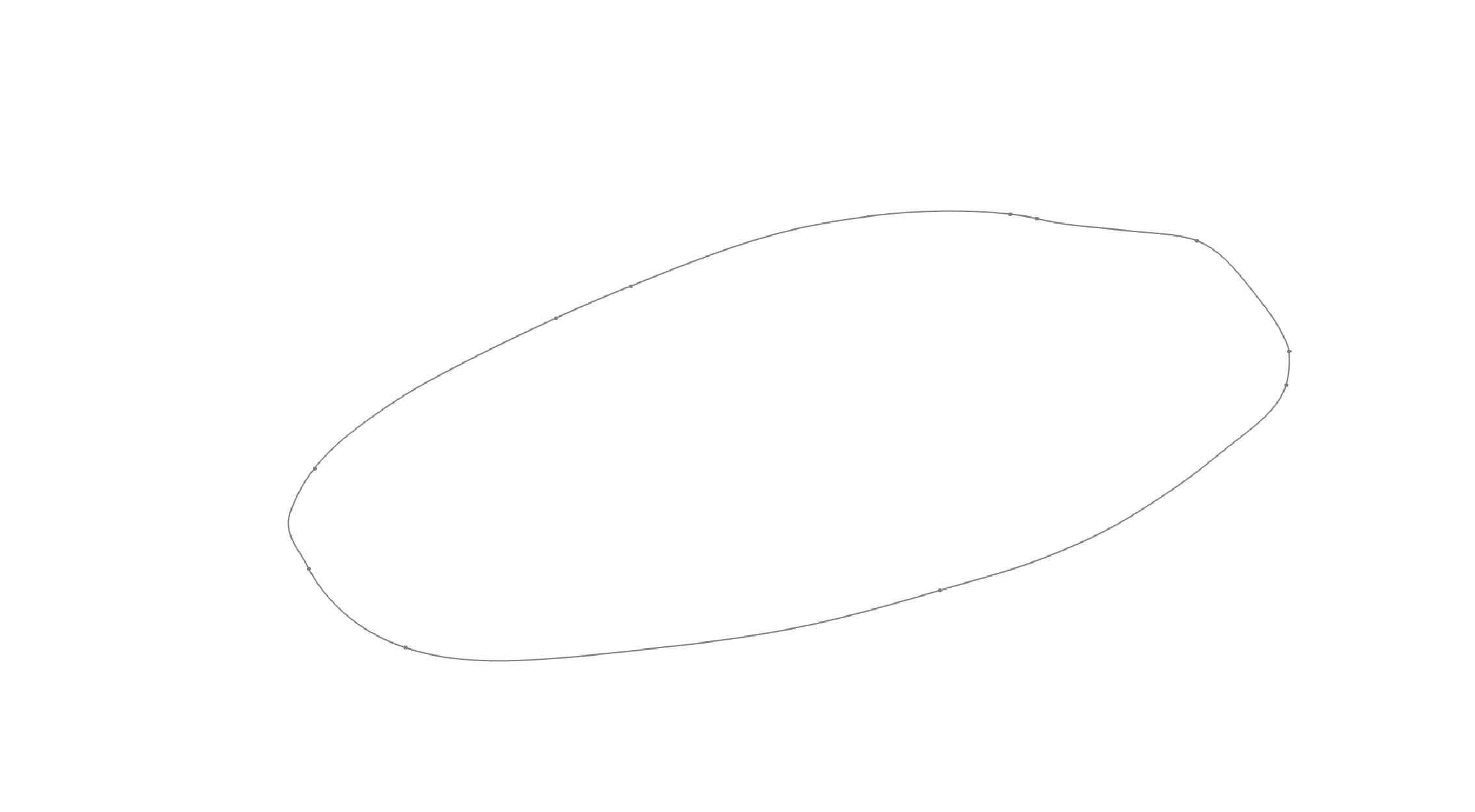

Supplement: Supplementary file 4 — Supporting Information [file ADVS-10-2203062-s013.zip › advs202203062-sup-0004-Supplementary-DataS3/Supplementary Data S3/153.jpg]

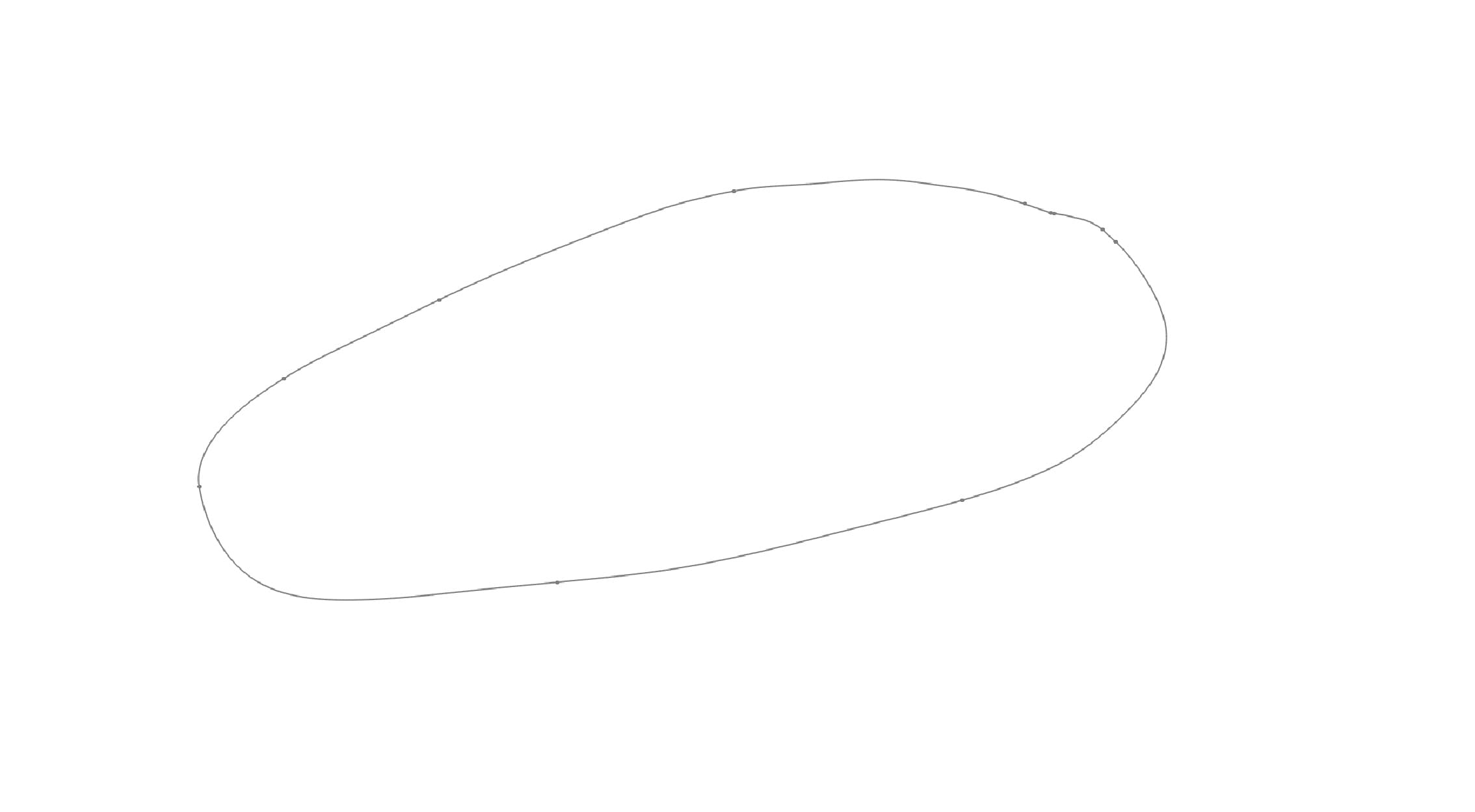

Supplement: Supplementary file 4 — Supporting Information [file ADVS-10-2203062-s013.zip › advs202203062-sup-0004-Supplementary-DataS3/Supplementary Data S3/154.jpg]

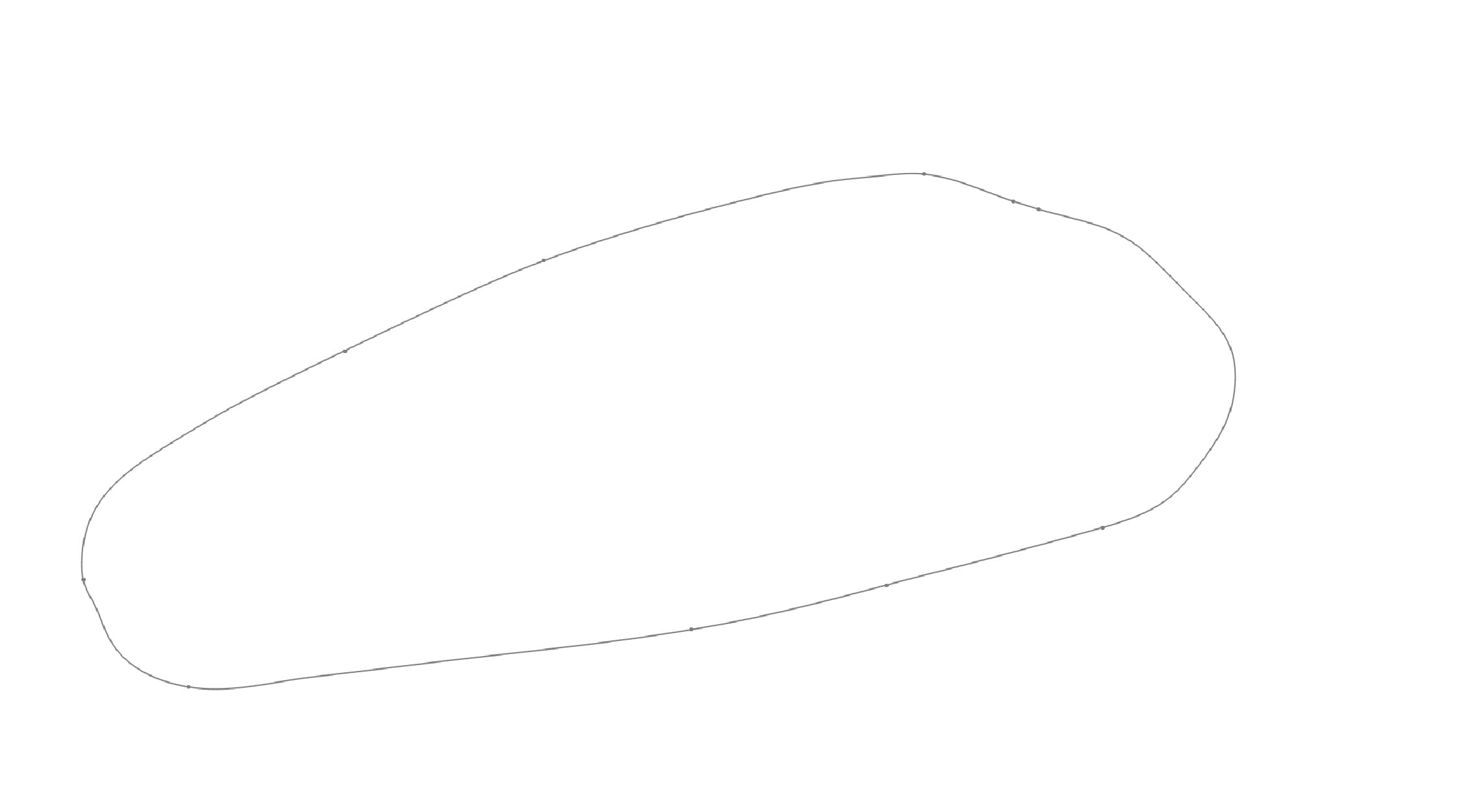

Supplement: Supplementary file 4 — Supporting Information [file ADVS-10-2203062-s013.zip › advs202203062-sup-0004-Supplementary-DataS3/Supplementary Data S3/155.jpg]

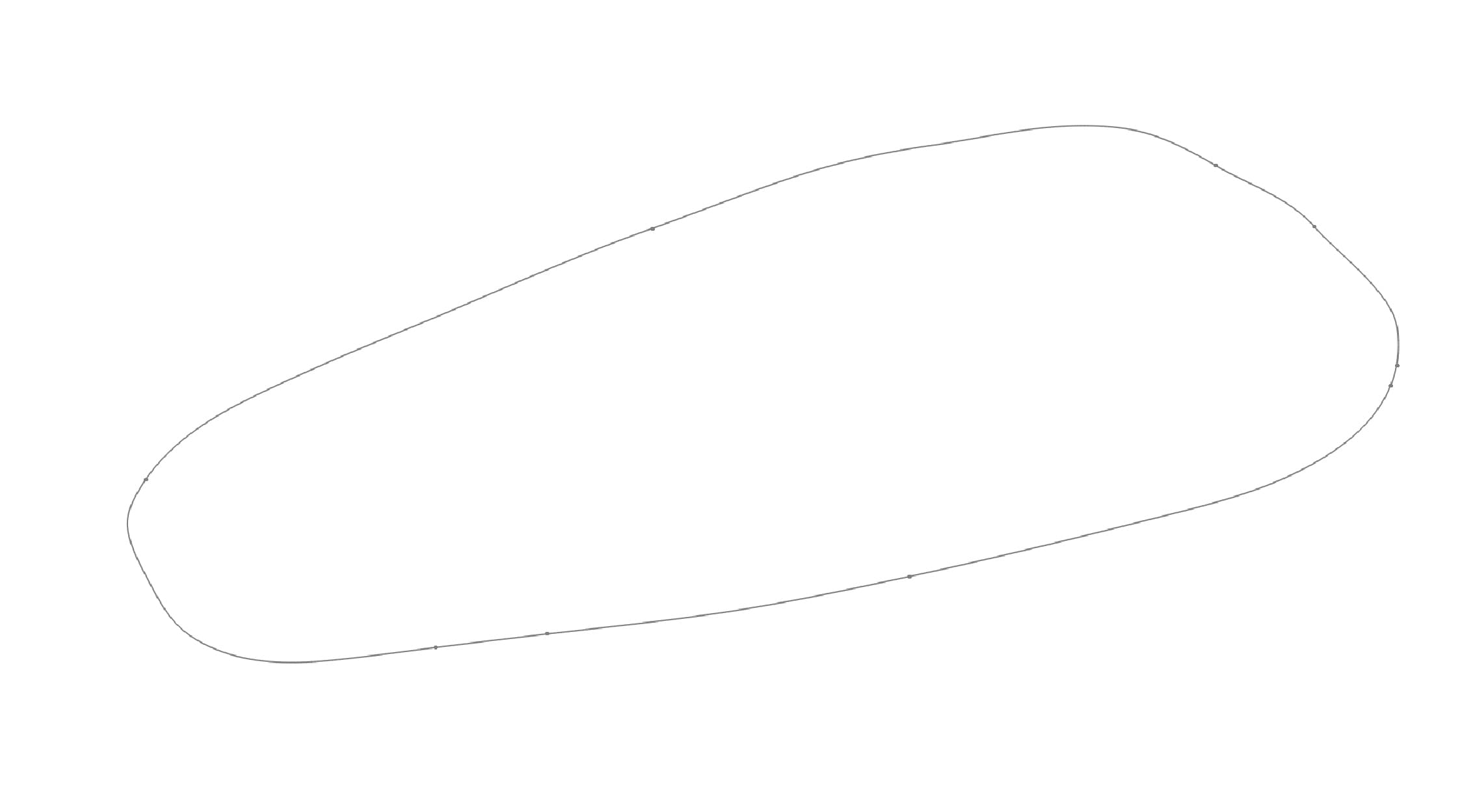

Supplement: Supplementary file 4 — Supporting Information [file ADVS-10-2203062-s013.zip › advs202203062-sup-0004-Supplementary-DataS3/Supplementary Data S3/156.jpg]

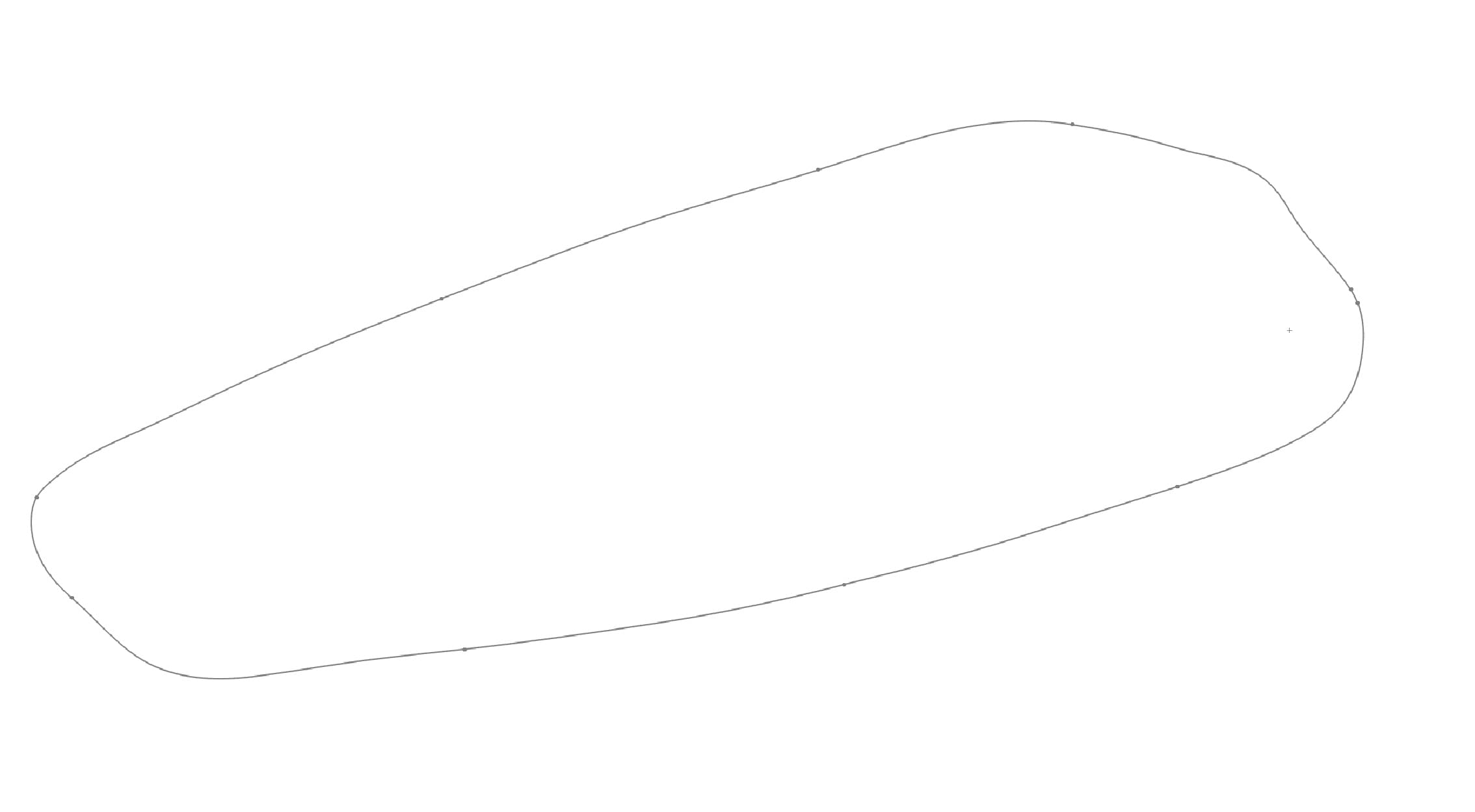

Supplement: Supplementary file 4 — Supporting Information [file ADVS-10-2203062-s013.zip › advs202203062-sup-0004-Supplementary-DataS3/Supplementary Data S3/157.jpg]

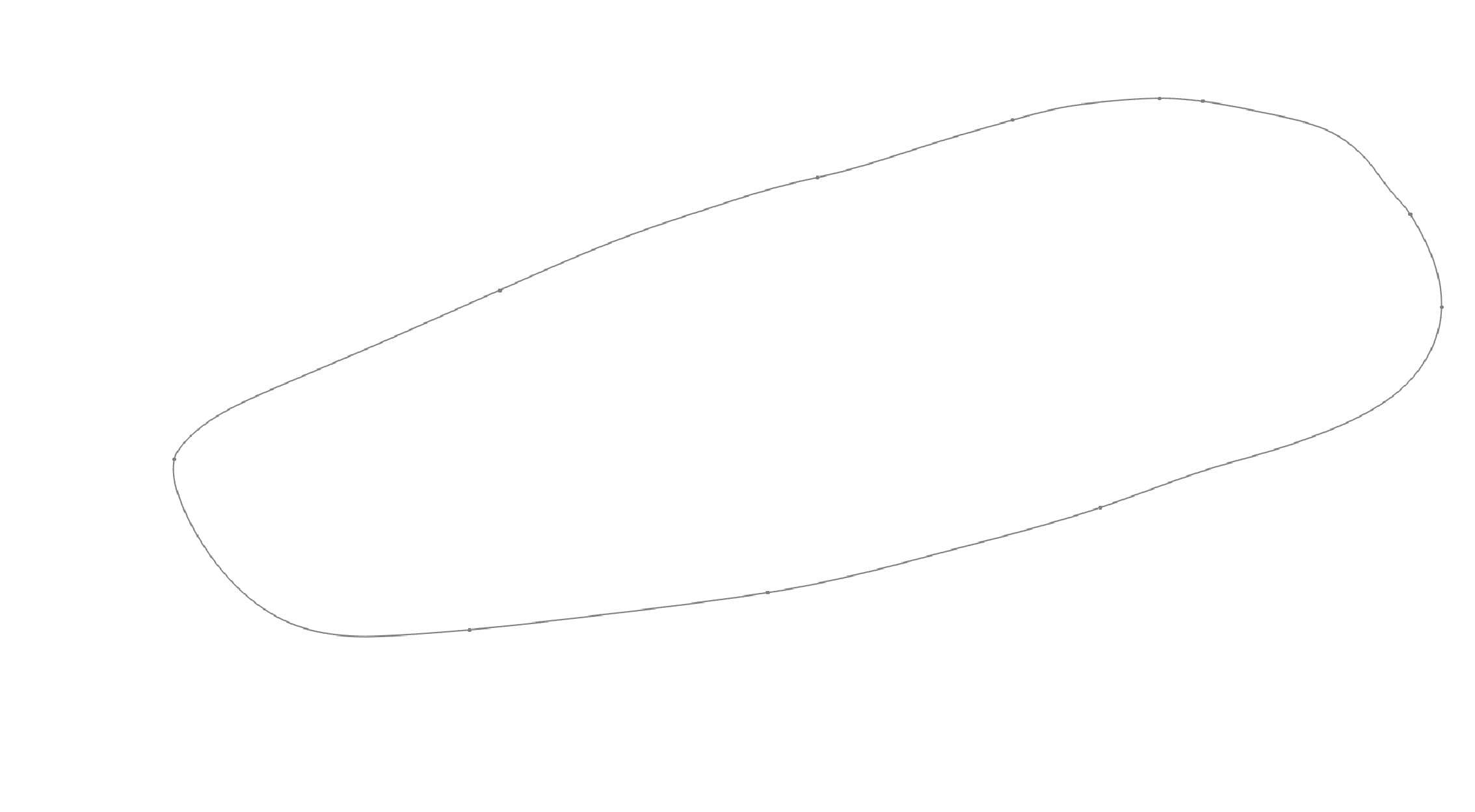

Supplement: Supplementary file 4 — Supporting Information [file ADVS-10-2203062-s013.zip › advs202203062-sup-0004-Supplementary-DataS3/Supplementary Data S3/158.jpg]

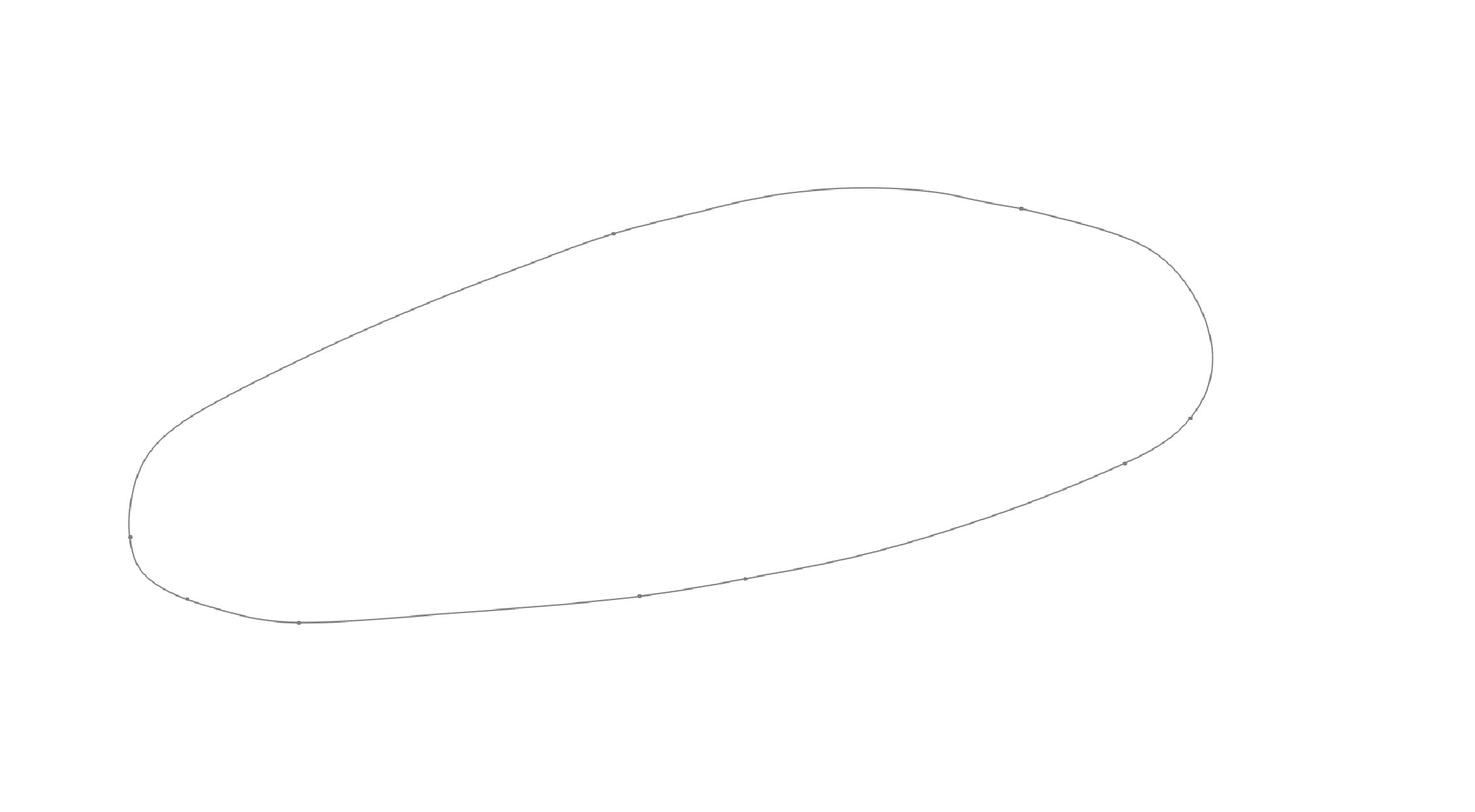

Supplement: Supplementary file 4 — Supporting Information [file ADVS-10-2203062-s013.zip › advs202203062-sup-0004-Supplementary-DataS3/Supplementary Data S3/159.jpg]

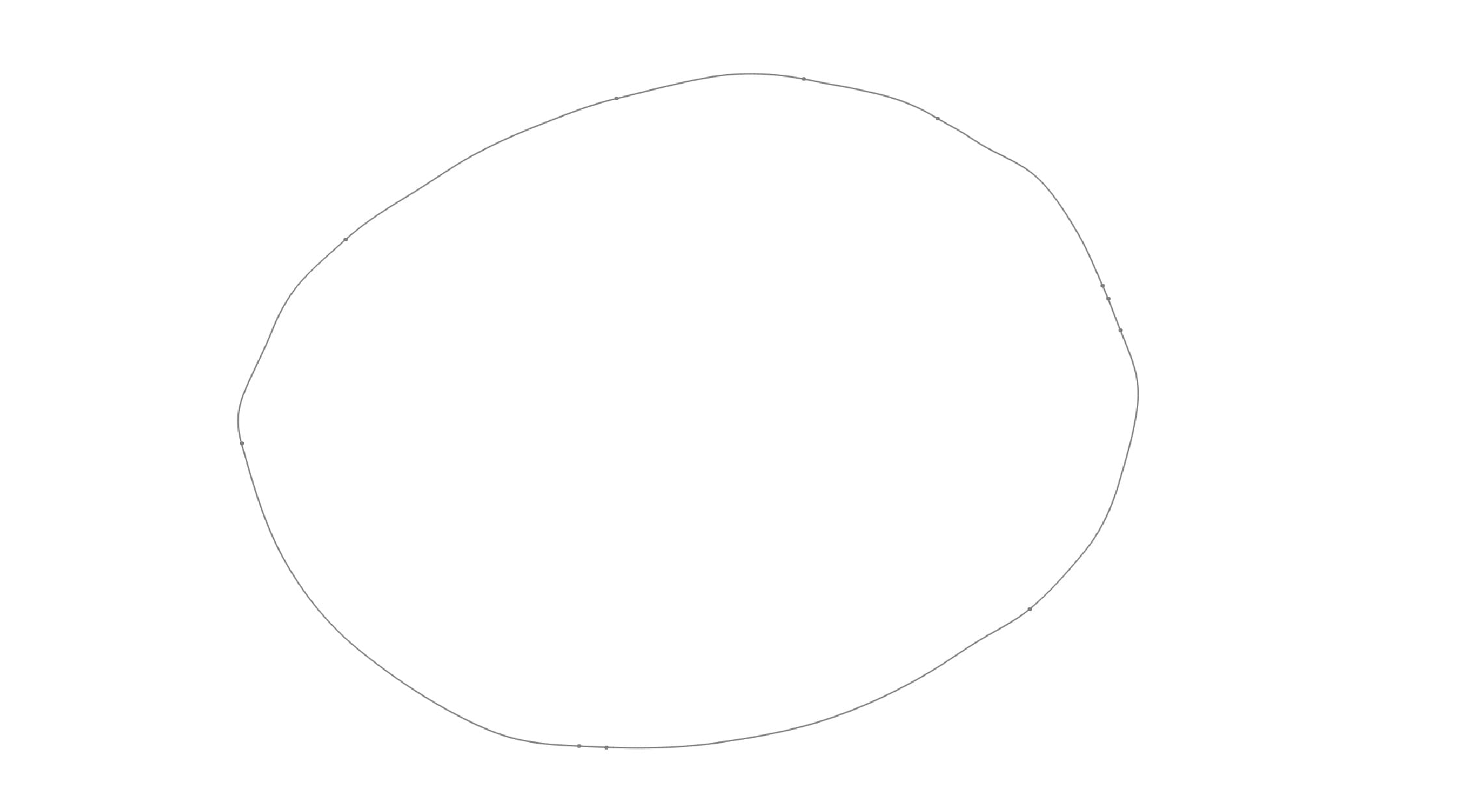

Supplement: Supplementary file 4 — Supporting Information [file ADVS-10-2203062-s013.zip › advs202203062-sup-0004-Supplementary-DataS3/Supplementary Data S3/16.jpg]

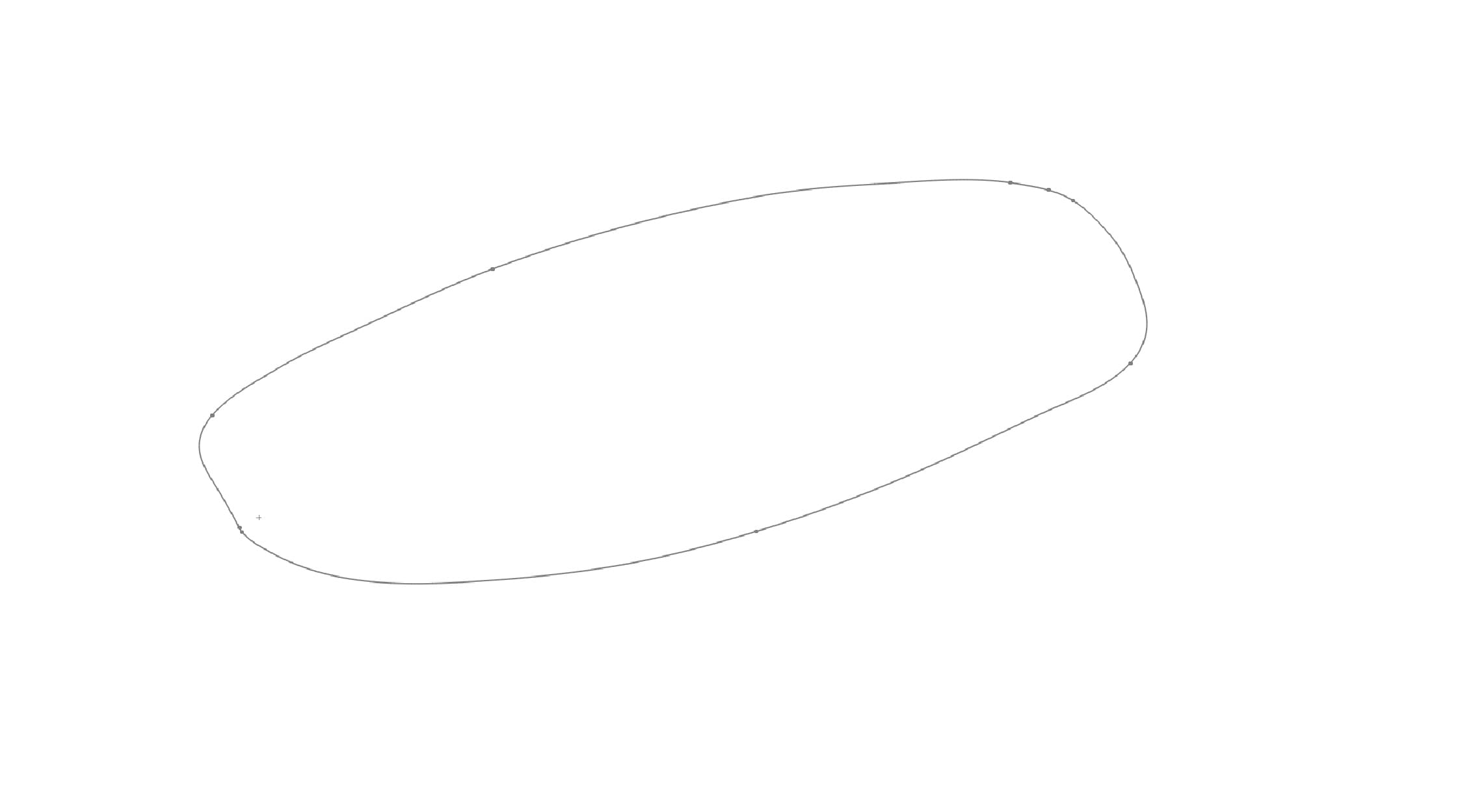

Supplement: Supplementary file 4 — Supporting Information [file ADVS-10-2203062-s013.zip › advs202203062-sup-0004-Supplementary-DataS3/Supplementary Data S3/160.jpg]

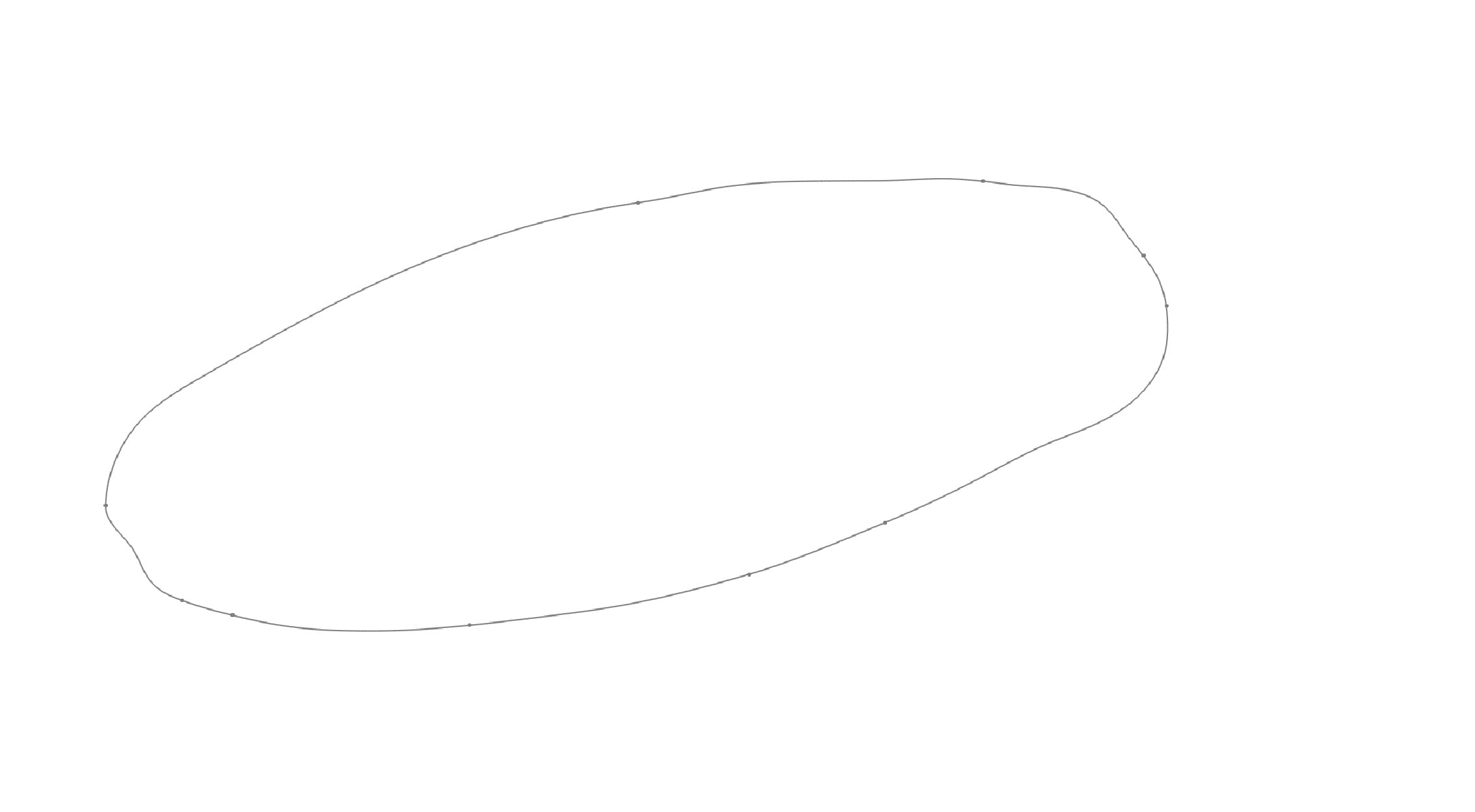

Supplement: Supplementary file 4 — Supporting Information [file ADVS-10-2203062-s013.zip › advs202203062-sup-0004-Supplementary-DataS3/Supplementary Data S3/161.jpg]

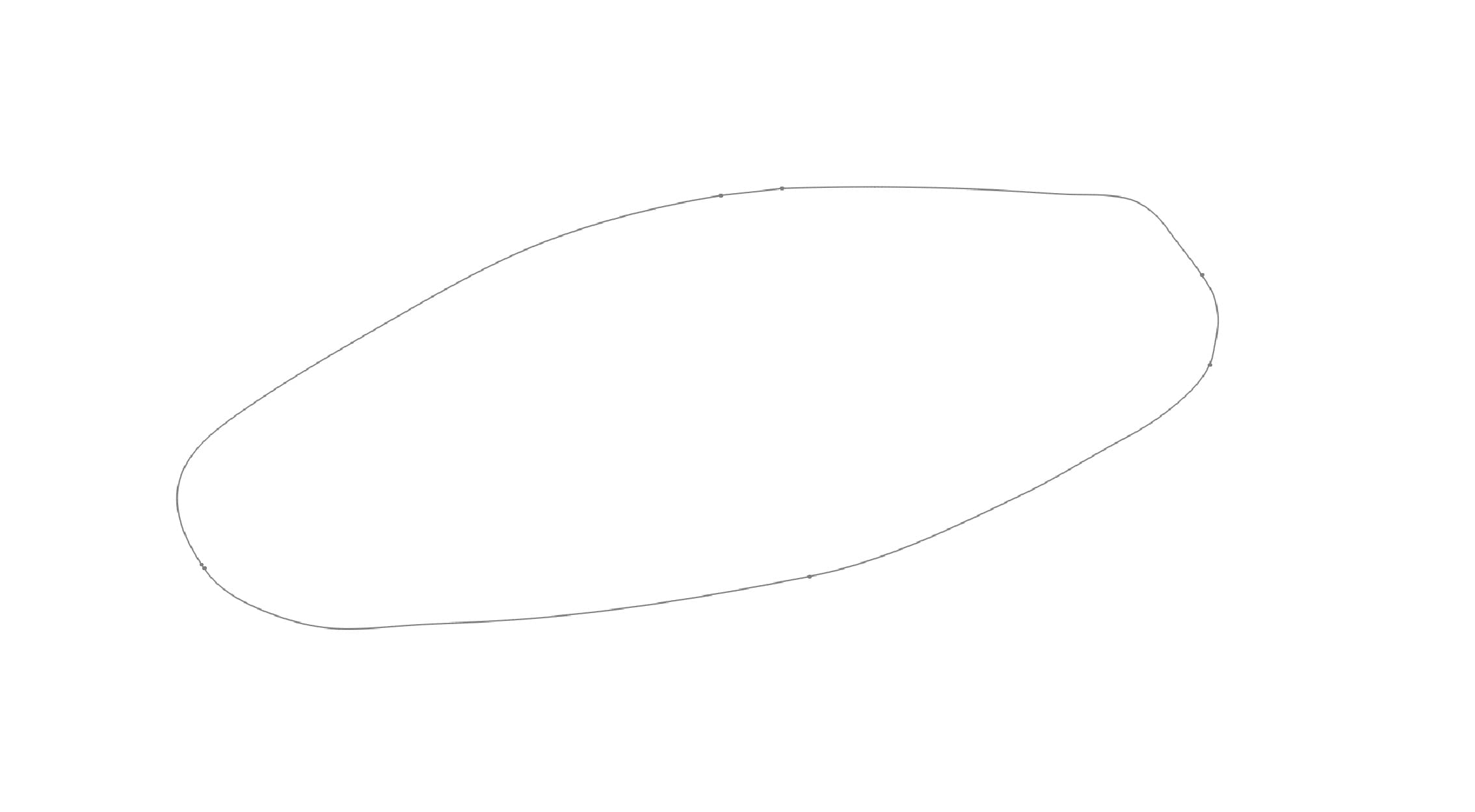

Supplement: Supplementary file 4 — Supporting Information [file ADVS-10-2203062-s013.zip › advs202203062-sup-0004-Supplementary-DataS3/Supplementary Data S3/162.jpg]

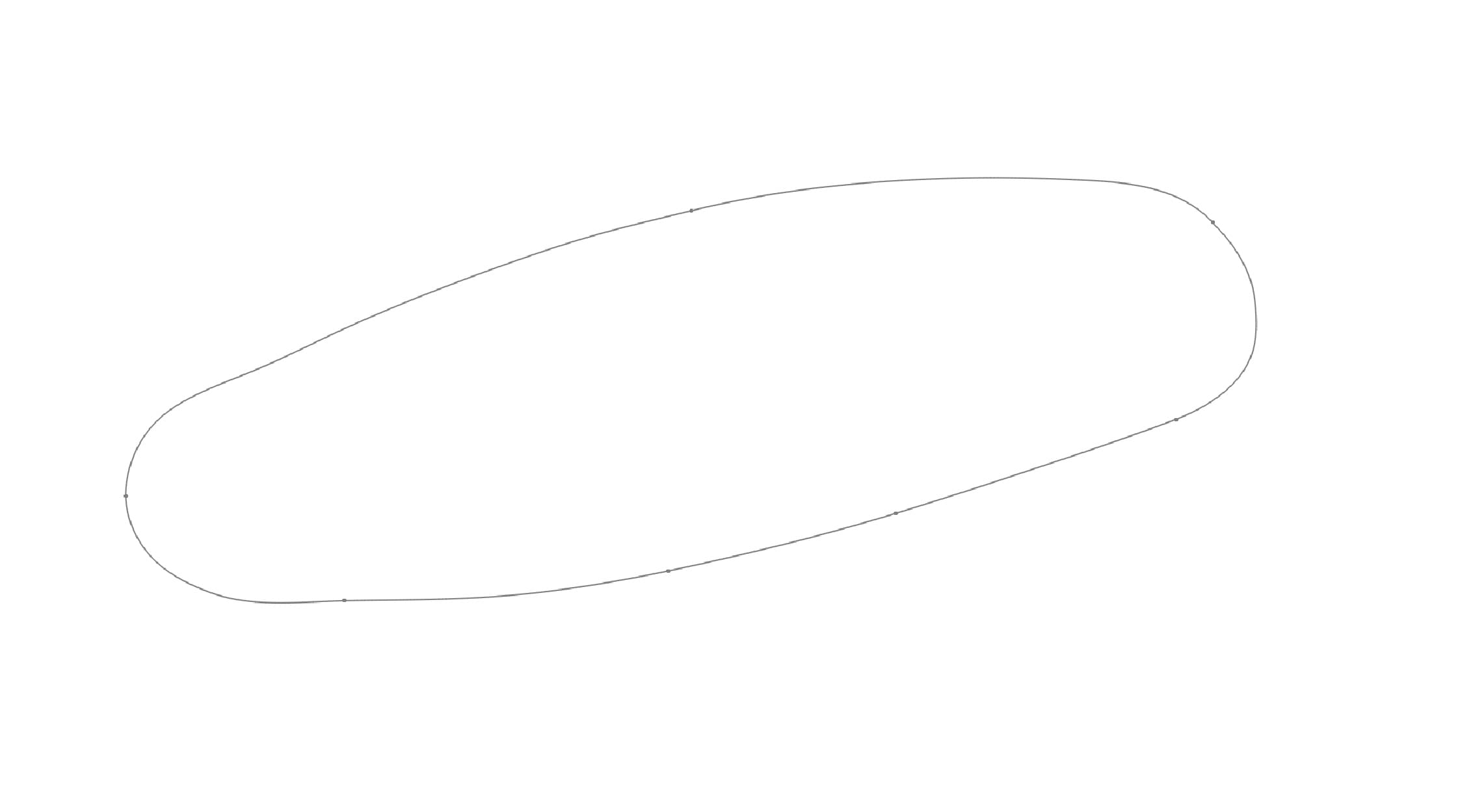

Supplement: Supplementary file 4 — Supporting Information [file ADVS-10-2203062-s013.zip › advs202203062-sup-0004-Supplementary-DataS3/Supplementary Data S3/163.jpg]

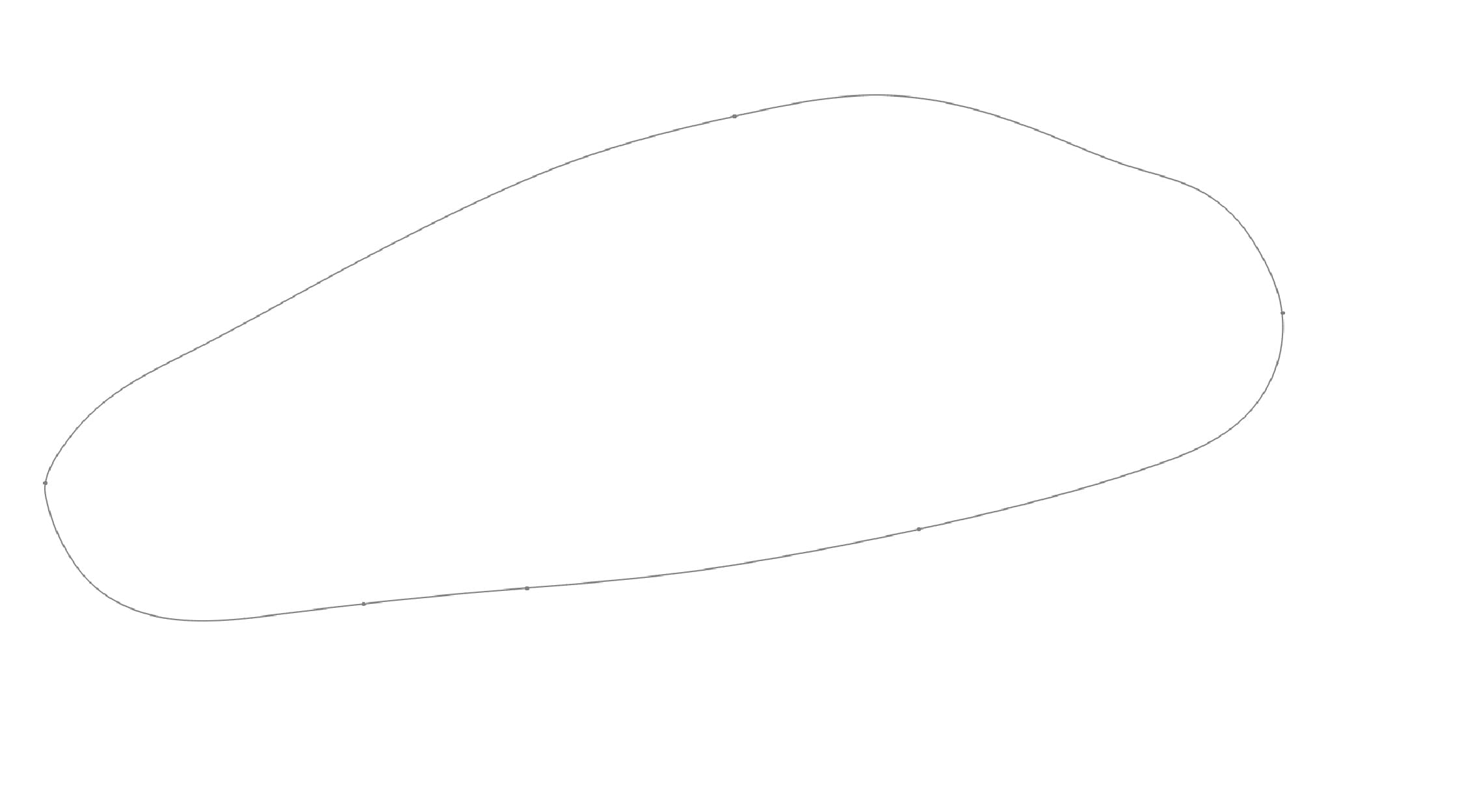

Supplement: Supplementary file 4 — Supporting Information [file ADVS-10-2203062-s013.zip › advs202203062-sup-0004-Supplementary-DataS3/Supplementary Data S3/164.jpg]

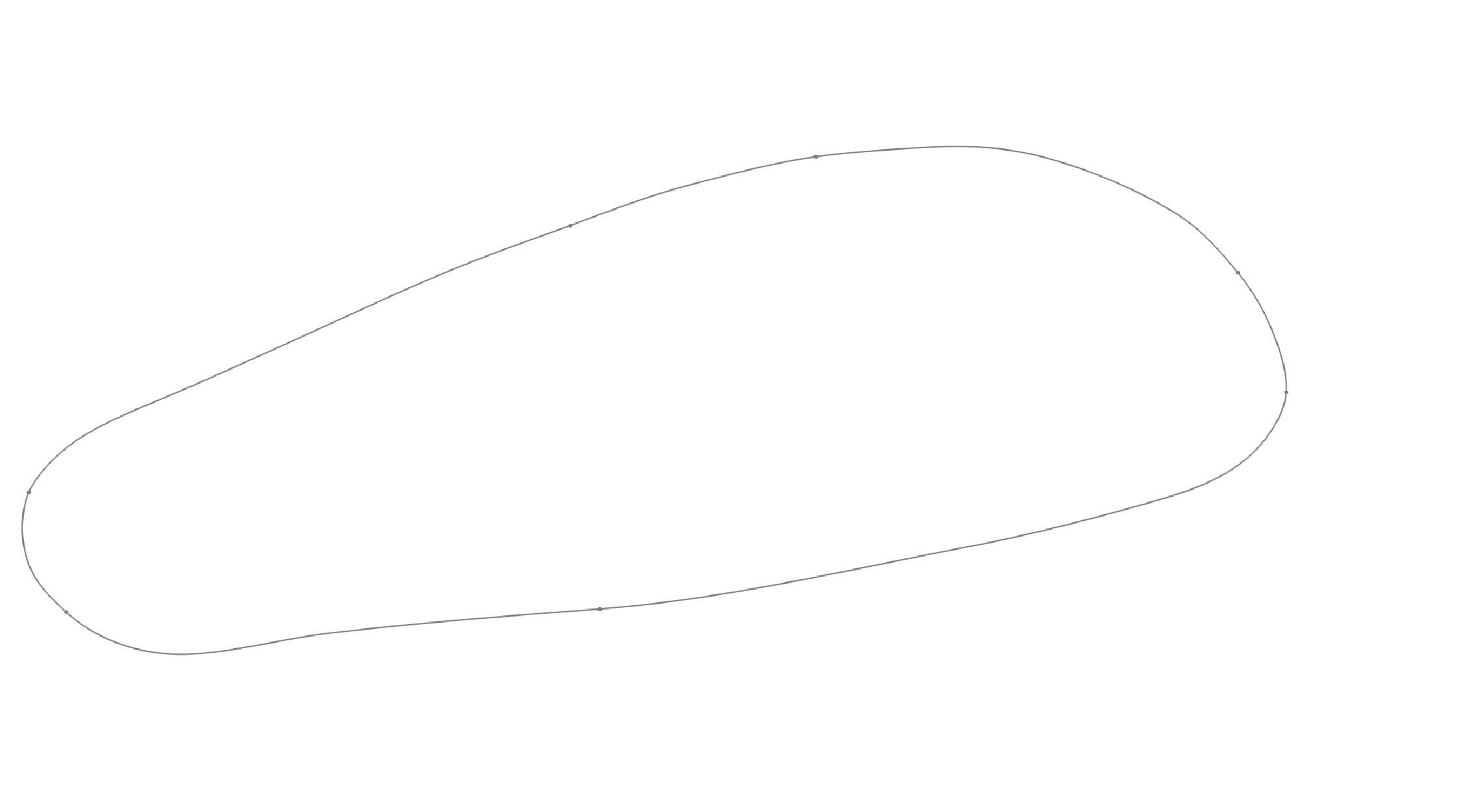

Supplement: Supplementary file 4 — Supporting Information [file ADVS-10-2203062-s013.zip › advs202203062-sup-0004-Supplementary-DataS3/Supplementary Data S3/165.jpg]

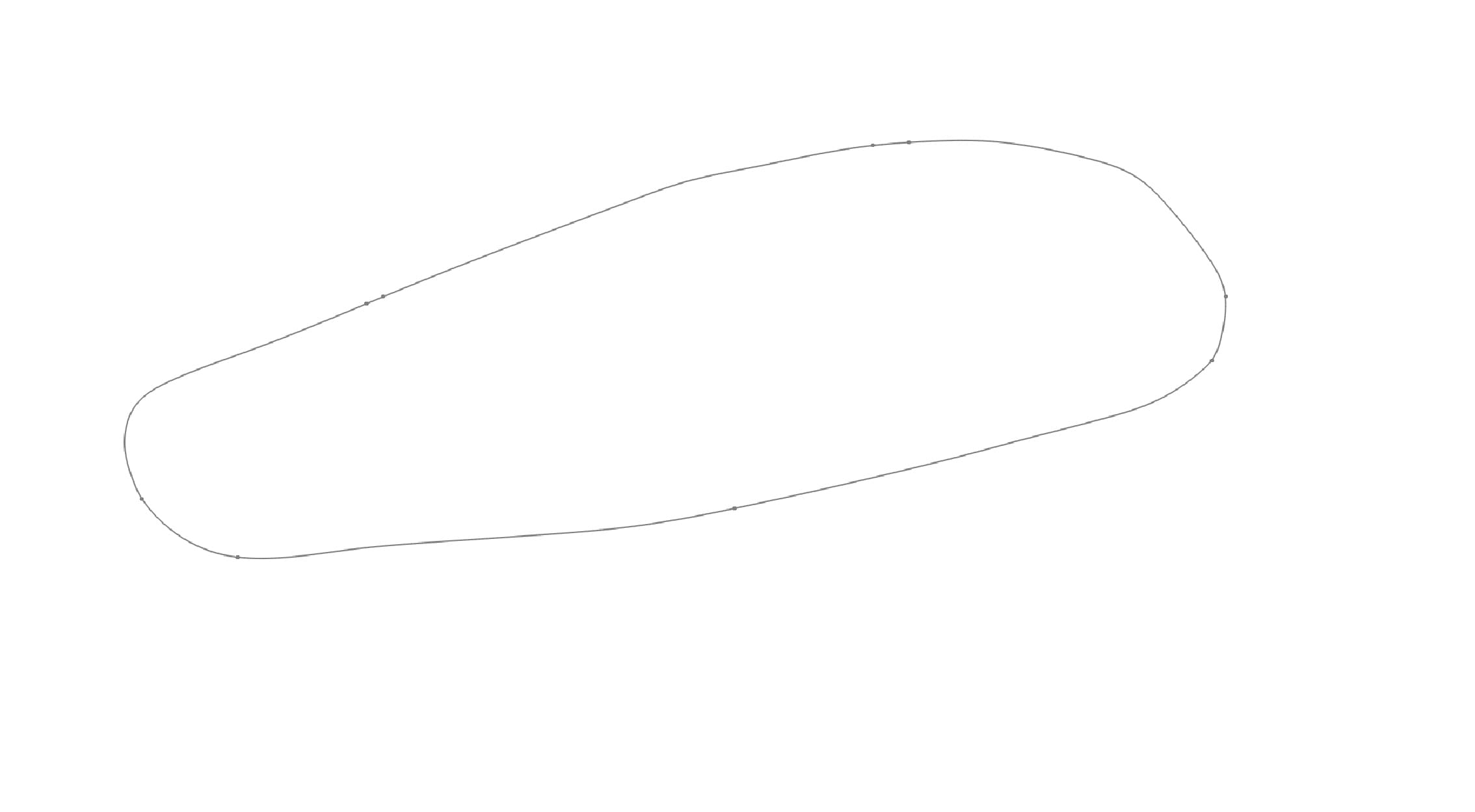

Supplement: Supplementary file 4 — Supporting Information [file ADVS-10-2203062-s013.zip › advs202203062-sup-0004-Supplementary-DataS3/Supplementary Data S3/166.jpg]

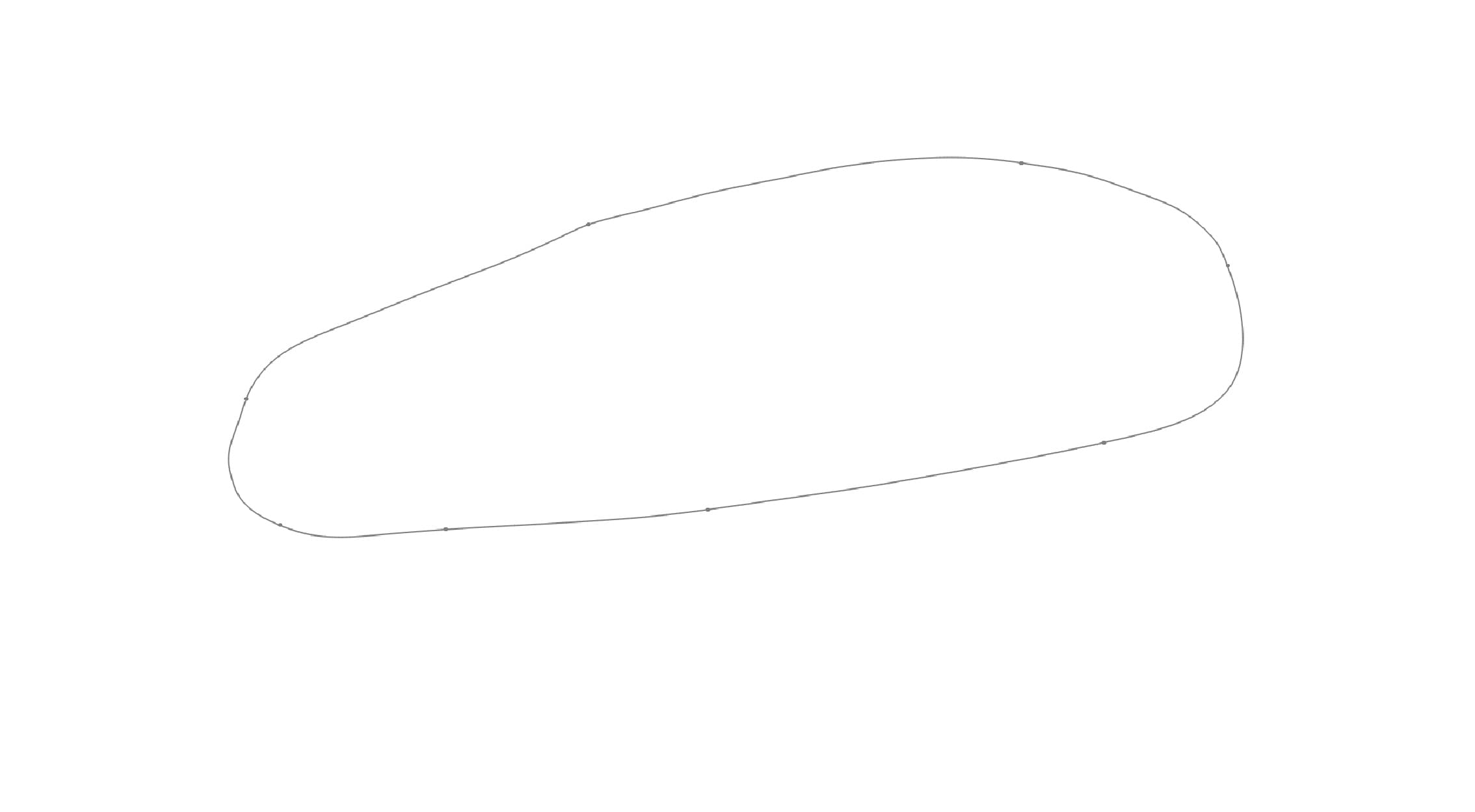

Supplement: Supplementary file 4 — Supporting Information [file ADVS-10-2203062-s013.zip › advs202203062-sup-0004-Supplementary-DataS3/Supplementary Data S3/167.jpg]

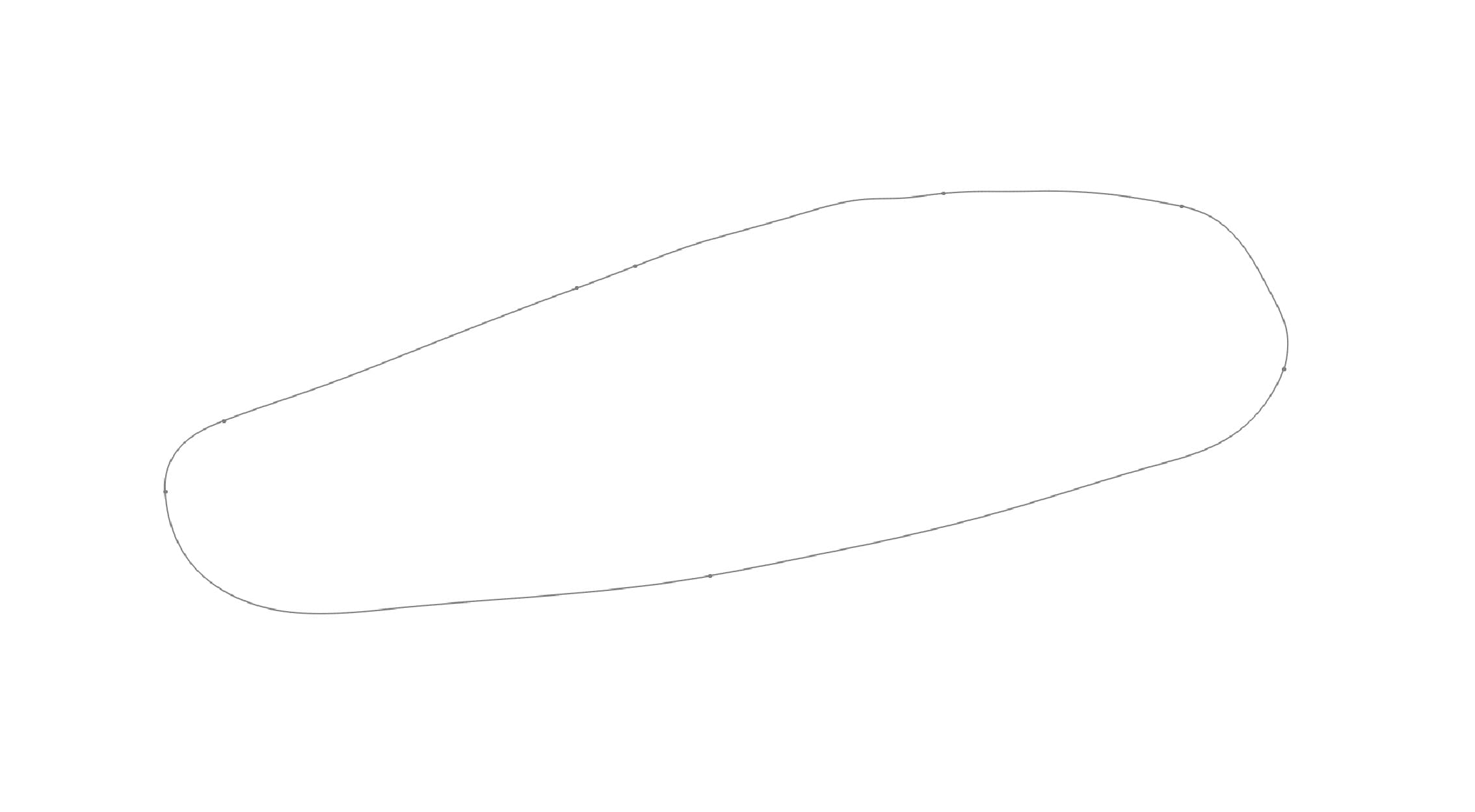

Supplement: Supplementary file 4 — Supporting Information [file ADVS-10-2203062-s013.zip › advs202203062-sup-0004-Supplementary-DataS3/Supplementary Data S3/168.jpg]

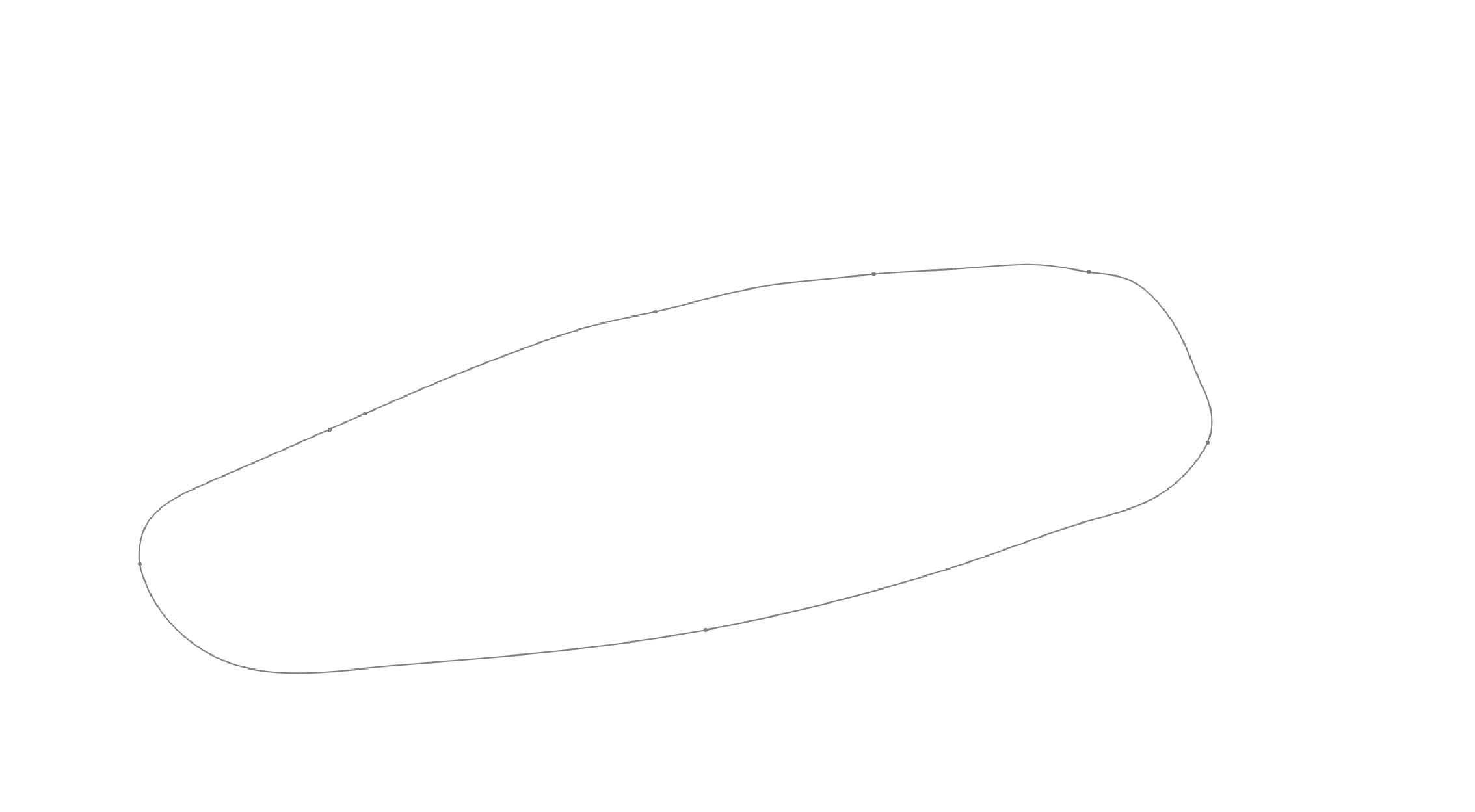

Supplement: Supplementary file 4 — Supporting Information [file ADVS-10-2203062-s013.zip › advs202203062-sup-0004-Supplementary-DataS3/Supplementary Data S3/169.jpg]

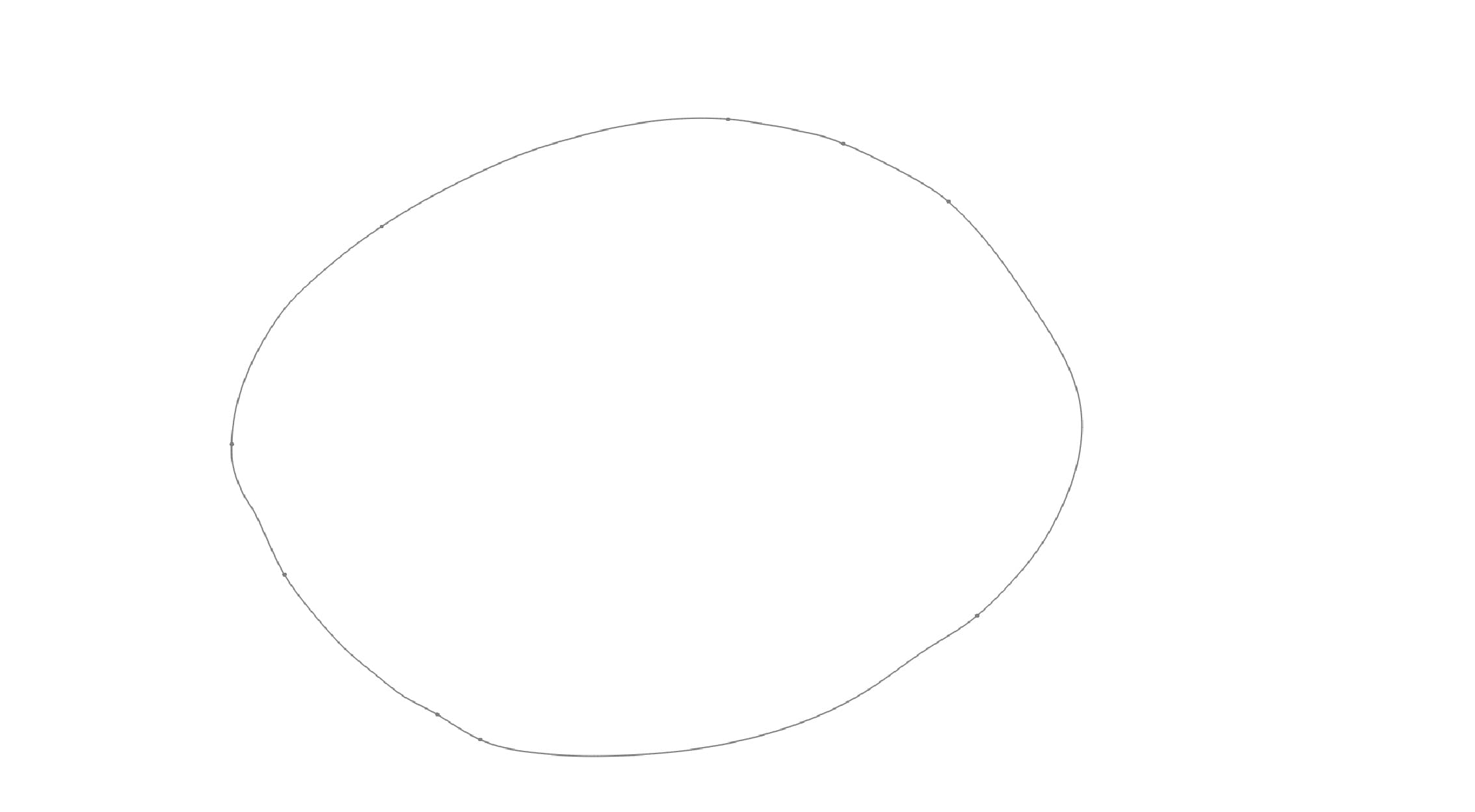

Supplement: Supplementary file 4 — Supporting Information [file ADVS-10-2203062-s013.zip › advs202203062-sup-0004-Supplementary-DataS3/Supplementary Data S3/17.jpg]

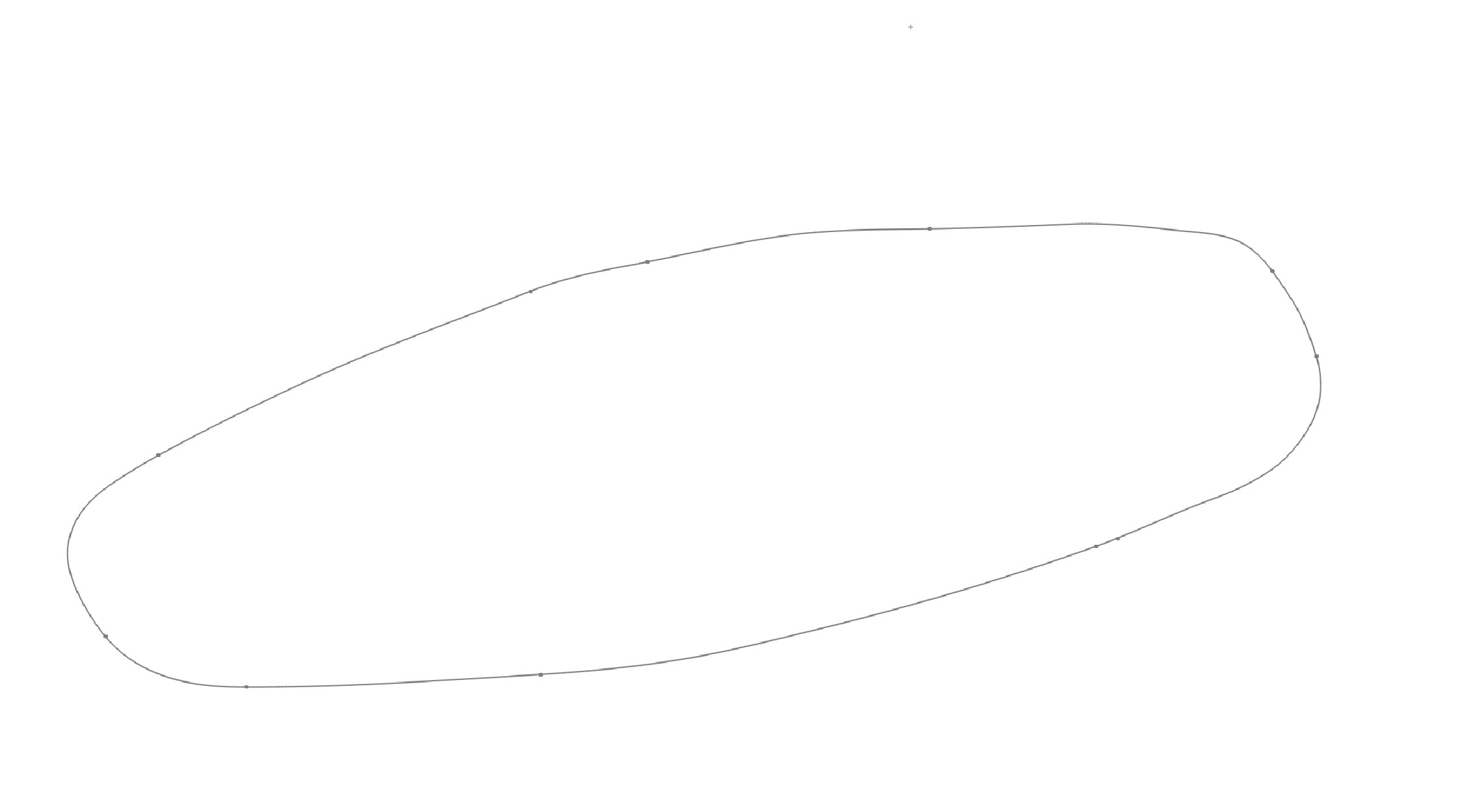

Supplement: Supplementary file 4 — Supporting Information [file ADVS-10-2203062-s013.zip › advs202203062-sup-0004-Supplementary-DataS3/Supplementary Data S3/170.jpg]

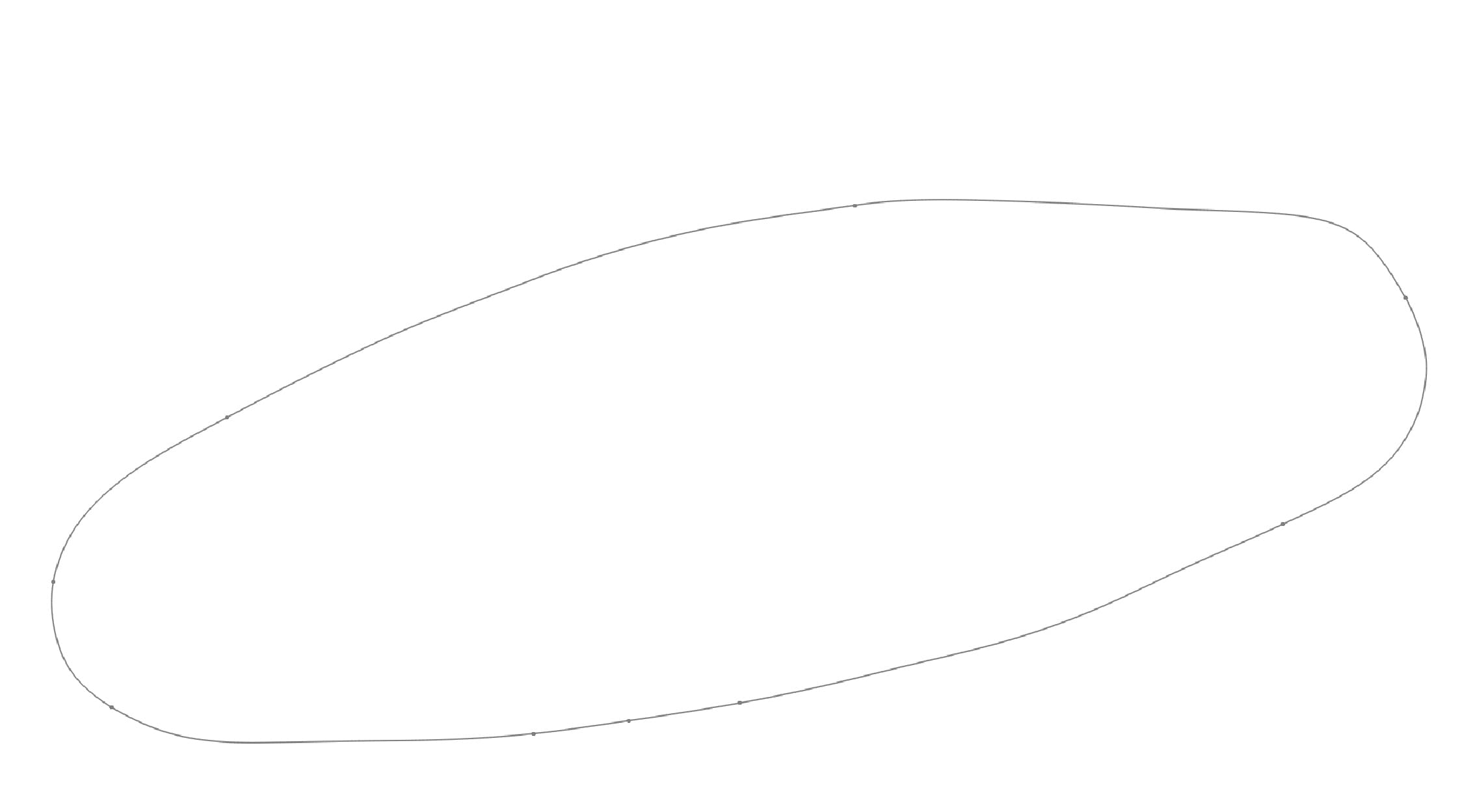

Supplement: Supplementary file 4 — Supporting Information [file ADVS-10-2203062-s013.zip › advs202203062-sup-0004-Supplementary-DataS3/Supplementary Data S3/171.jpg]

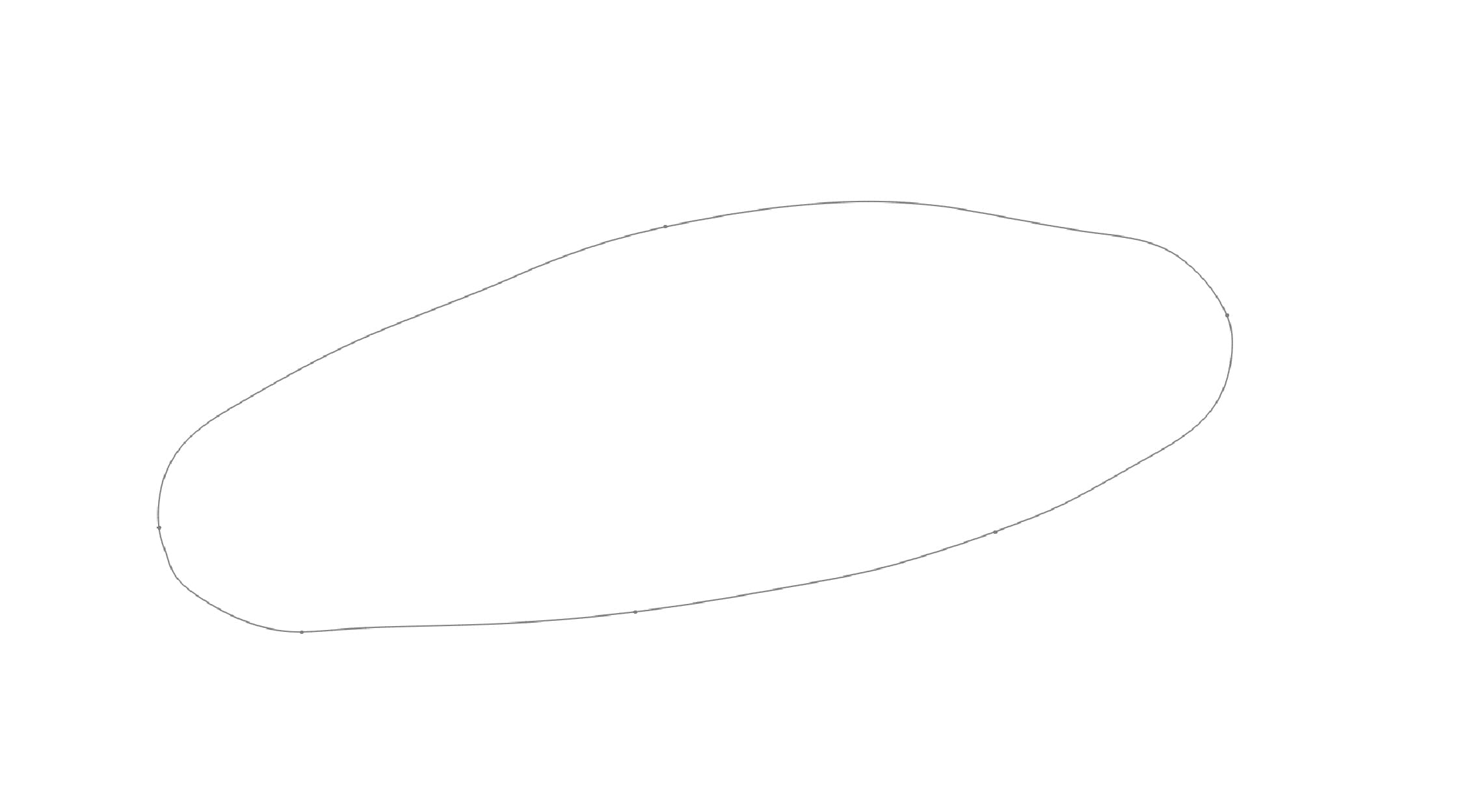

Supplement: Supplementary file 4 — Supporting Information [file ADVS-10-2203062-s013.zip › advs202203062-sup-0004-Supplementary-DataS3/Supplementary Data S3/172.jpg]

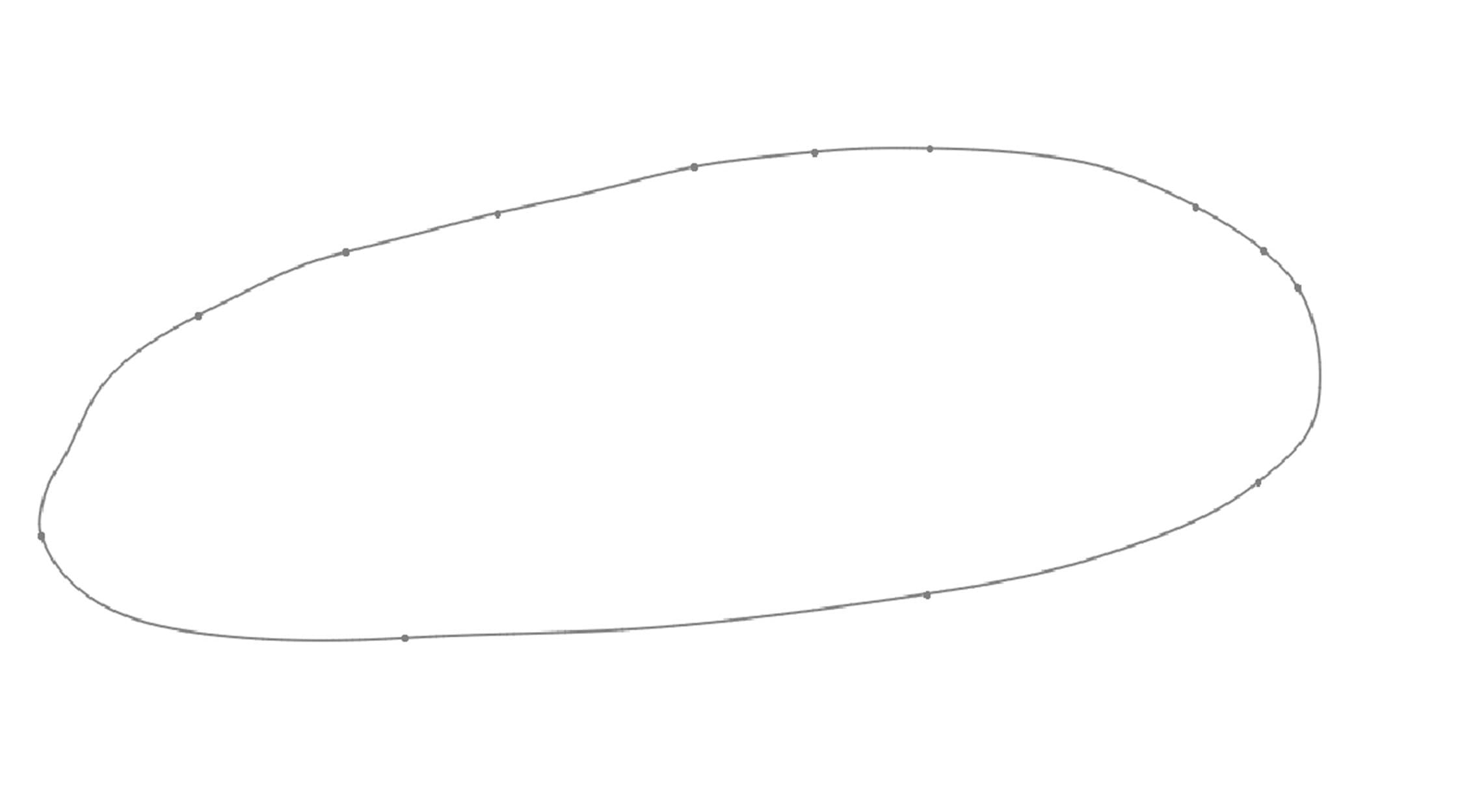

Supplement: Supplementary file 4 — Supporting Information [file ADVS-10-2203062-s013.zip › advs202203062-sup-0004-Supplementary-DataS3/Supplementary Data S3/173.jpg]

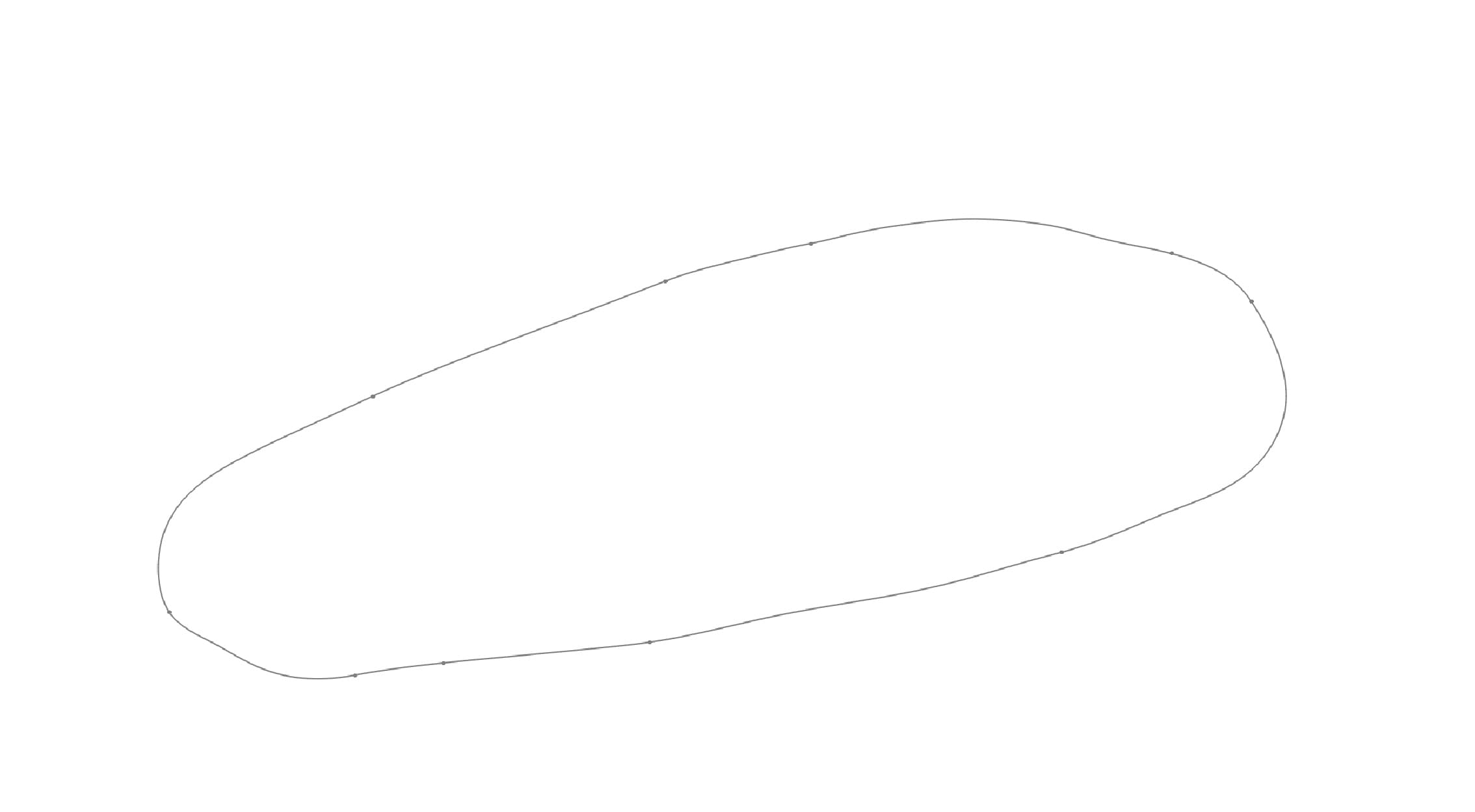

Supplement: Supplementary file 4 — Supporting Information [file ADVS-10-2203062-s013.zip › advs202203062-sup-0004-Supplementary-DataS3/Supplementary Data S3/174.jpg]

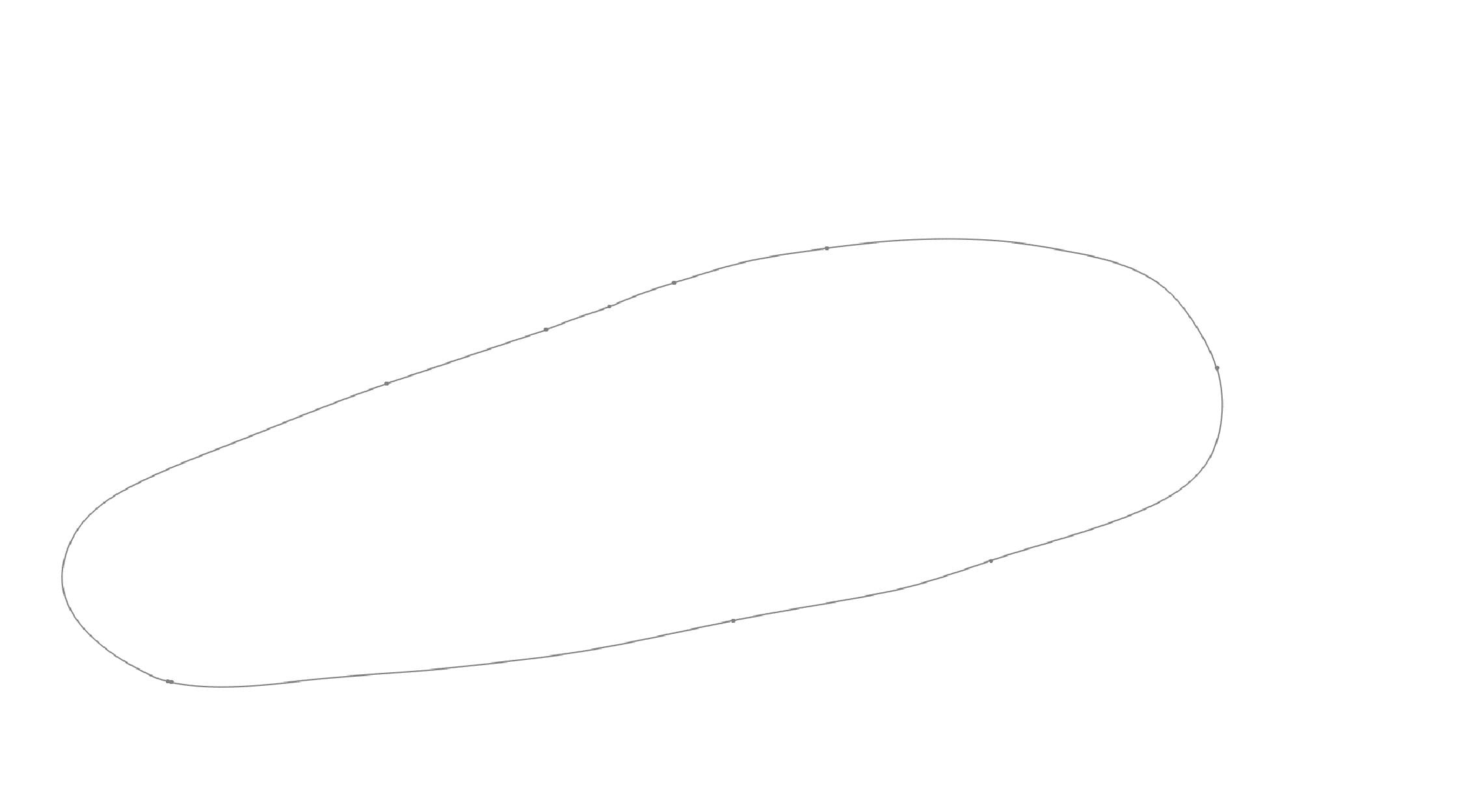

Supplement: Supplementary file 4 — Supporting Information [file ADVS-10-2203062-s013.zip › advs202203062-sup-0004-Supplementary-DataS3/Supplementary Data S3/175.jpg]

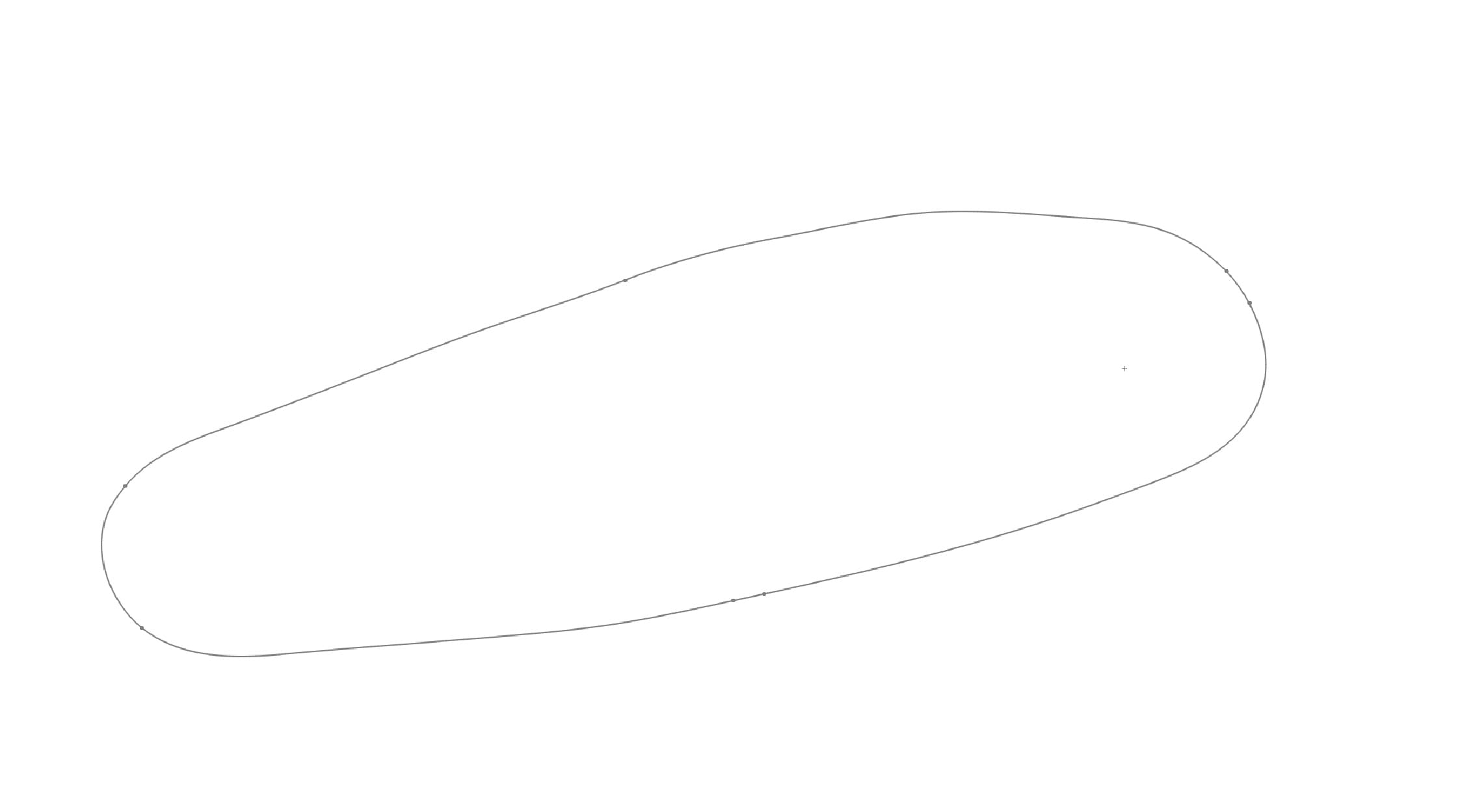

Supplement: Supplementary file 4 — Supporting Information [file ADVS-10-2203062-s013.zip › advs202203062-sup-0004-Supplementary-DataS3/Supplementary Data S3/176.jpg]

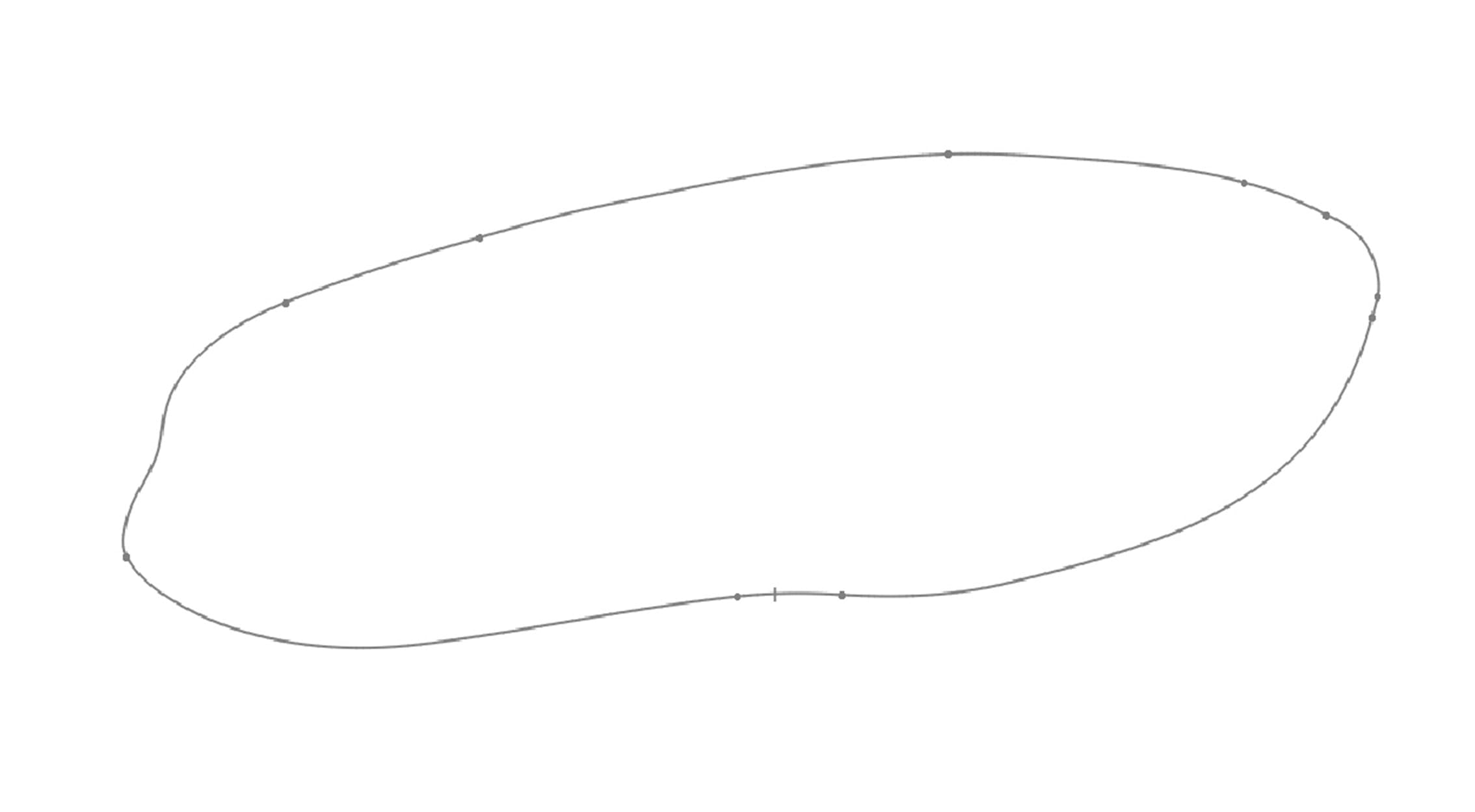

Supplement: Supplementary file 4 — Supporting Information [file ADVS-10-2203062-s013.zip › advs202203062-sup-0004-Supplementary-DataS3/Supplementary Data S3/177.jpg]

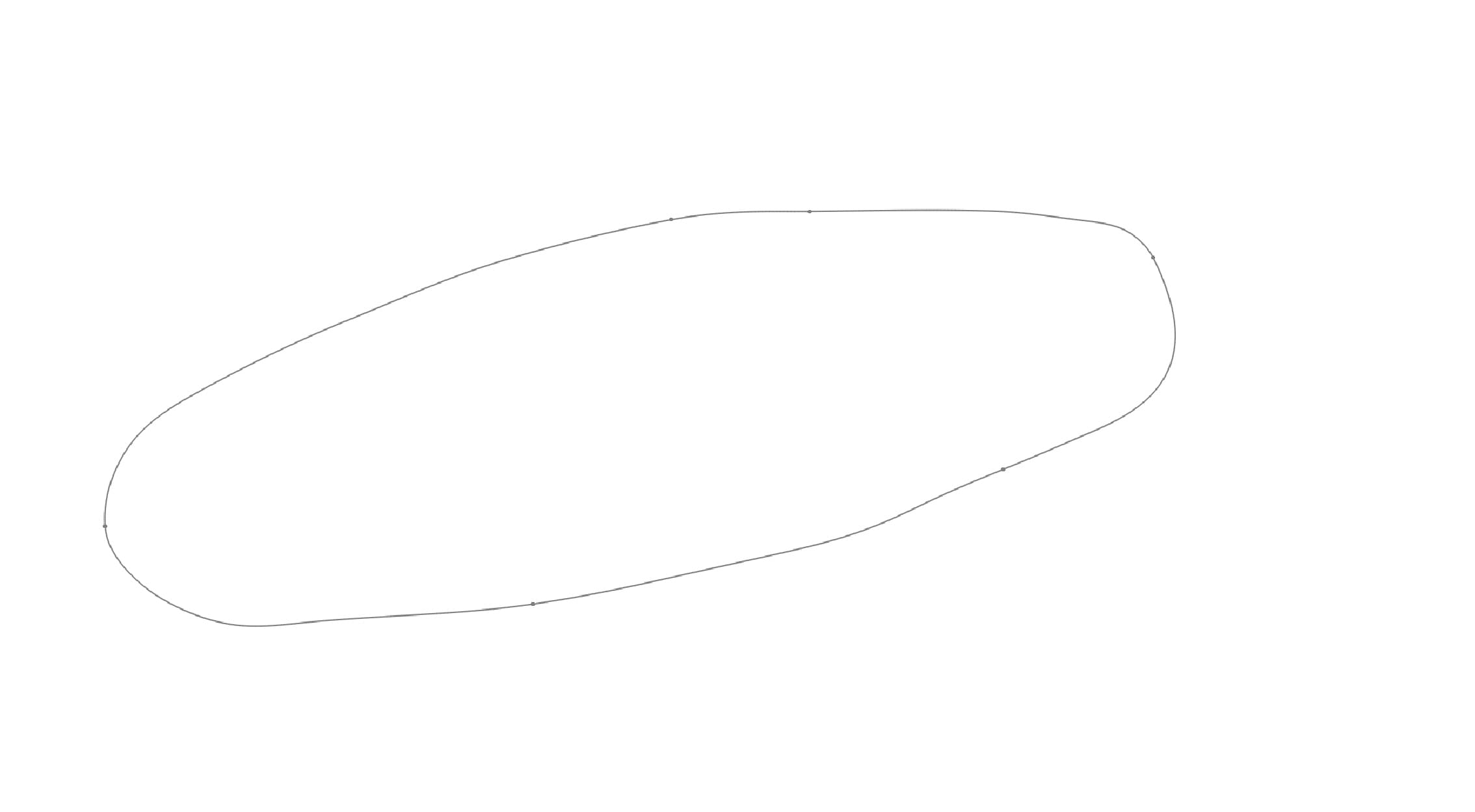

Supplement: Supplementary file 4 — Supporting Information [file ADVS-10-2203062-s013.zip › advs202203062-sup-0004-Supplementary-DataS3/Supplementary Data S3/178.jpg]

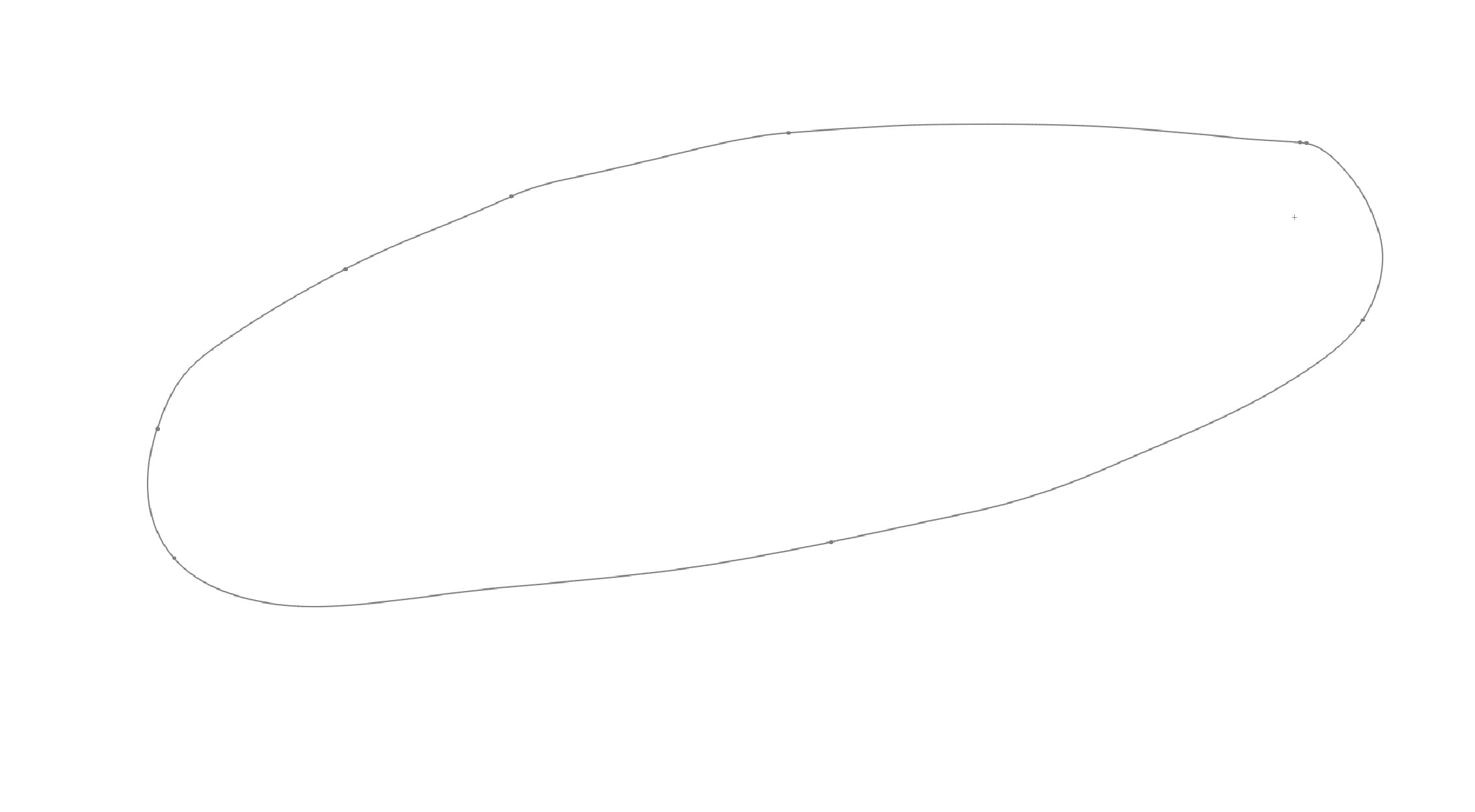

Supplement: Supplementary file 4 — Supporting Information [file ADVS-10-2203062-s013.zip › advs202203062-sup-0004-Supplementary-DataS3/Supplementary Data S3/179.jpg]

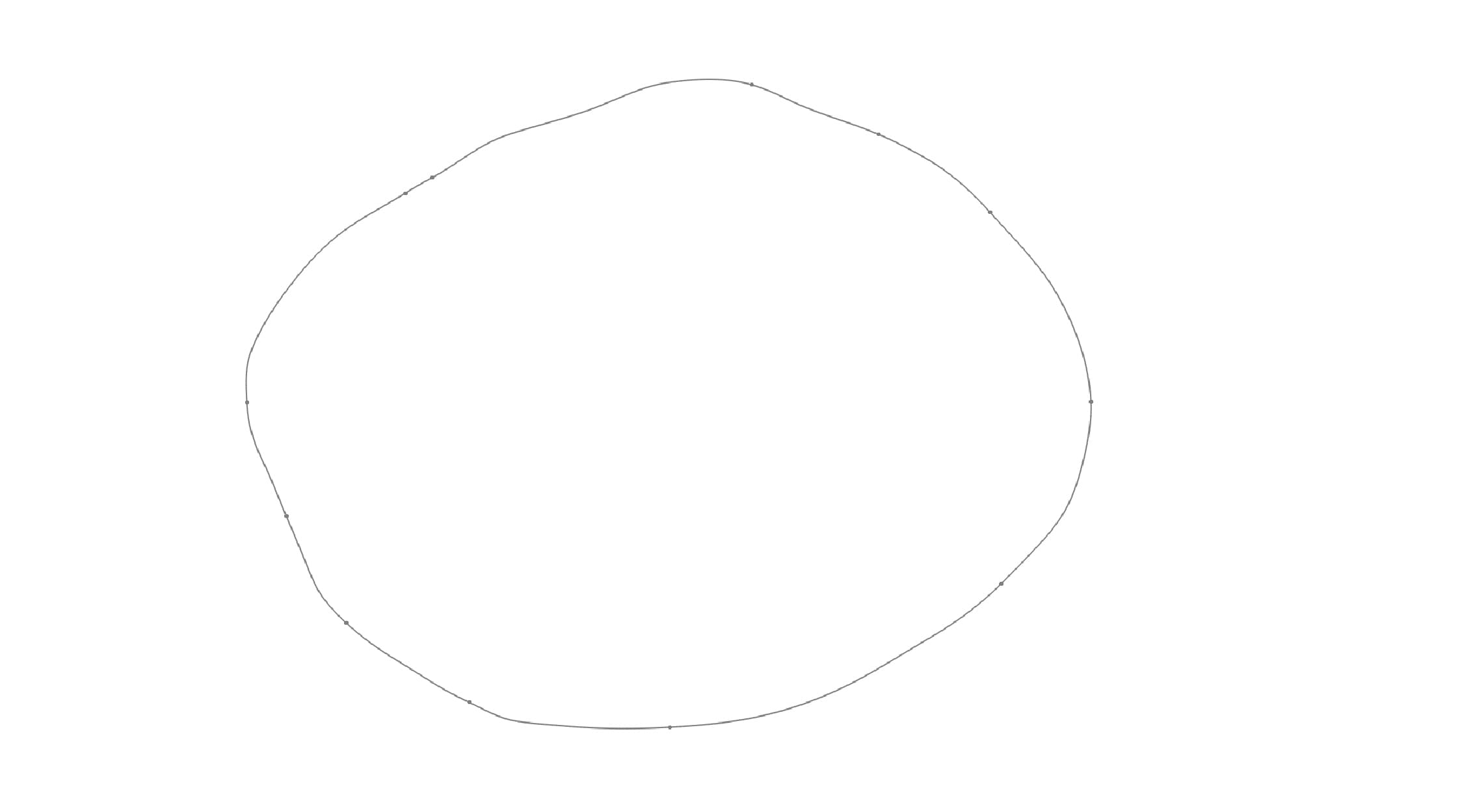

Supplement: Supplementary file 4 — Supporting Information [file ADVS-10-2203062-s013.zip › advs202203062-sup-0004-Supplementary-DataS3/Supplementary Data S3/18.jpg]

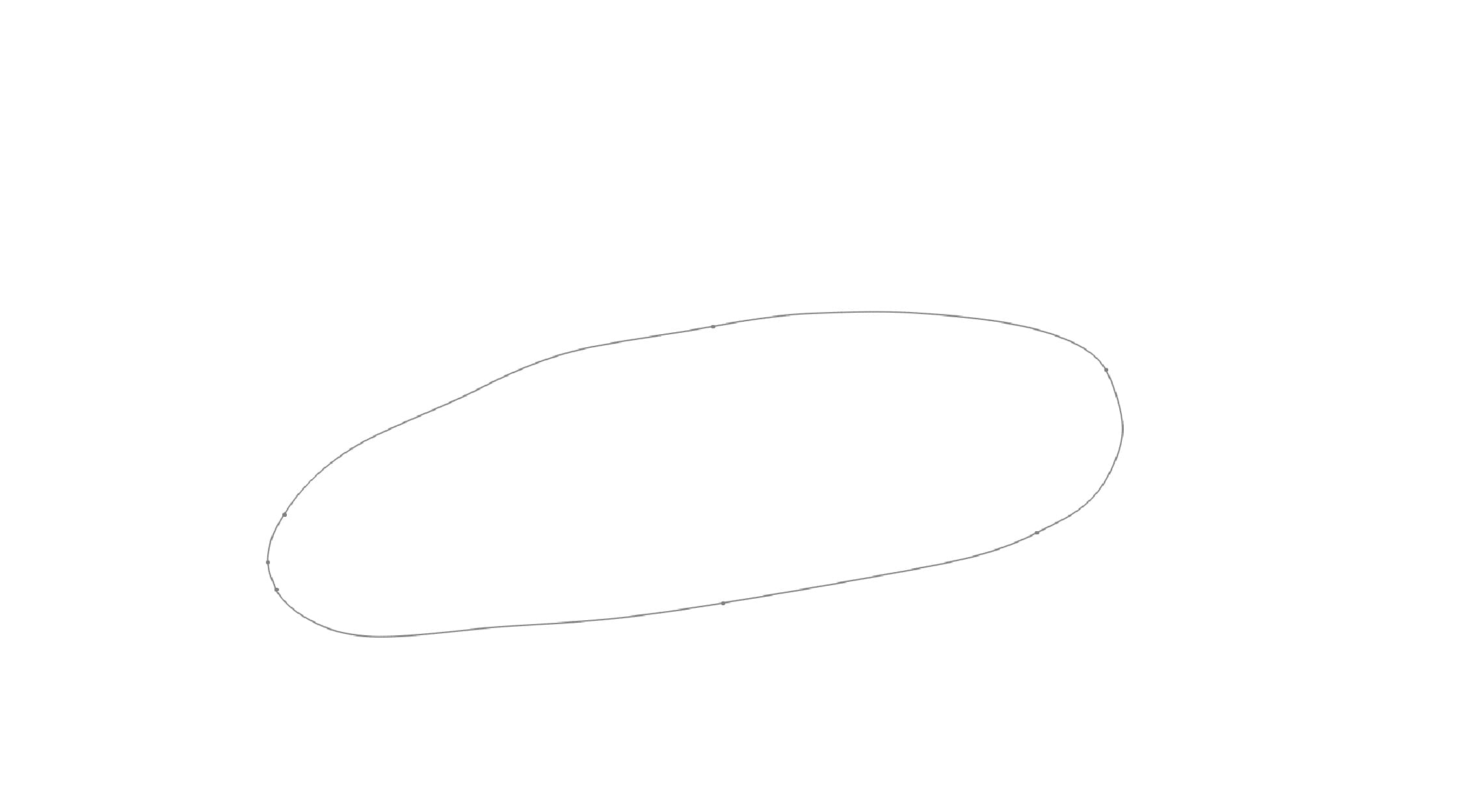

Supplement: Supplementary file 4 — Supporting Information [file ADVS-10-2203062-s013.zip › advs202203062-sup-0004-Supplementary-DataS3/Supplementary Data S3/180.jpg]

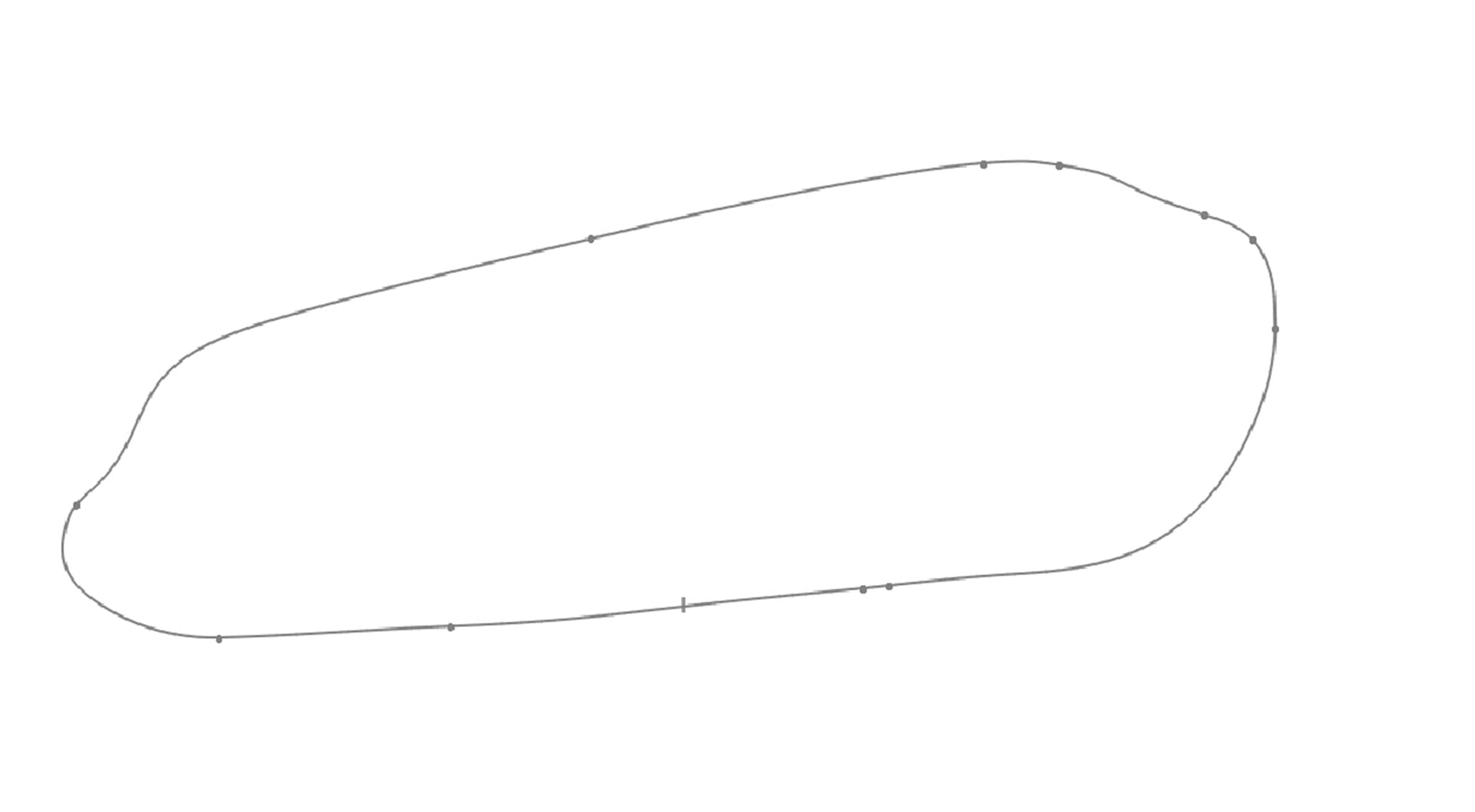

Supplement: Supplementary file 4 — Supporting Information [file ADVS-10-2203062-s013.zip › advs202203062-sup-0004-Supplementary-DataS3/Supplementary Data S3/181.jpg]

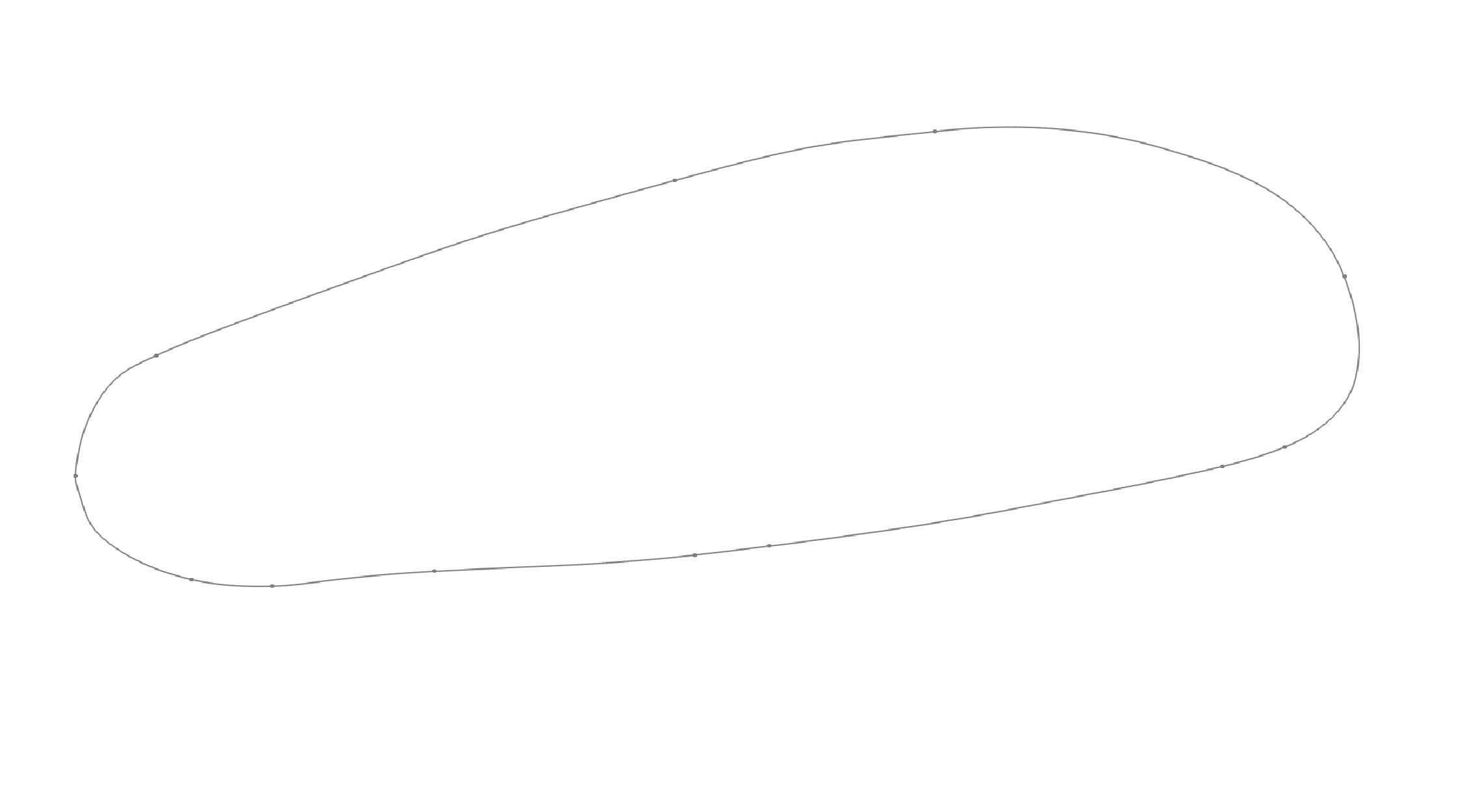

Supplement: Supplementary file 4 — Supporting Information [file ADVS-10-2203062-s013.zip › advs202203062-sup-0004-Supplementary-DataS3/Supplementary Data S3/182.jpg]

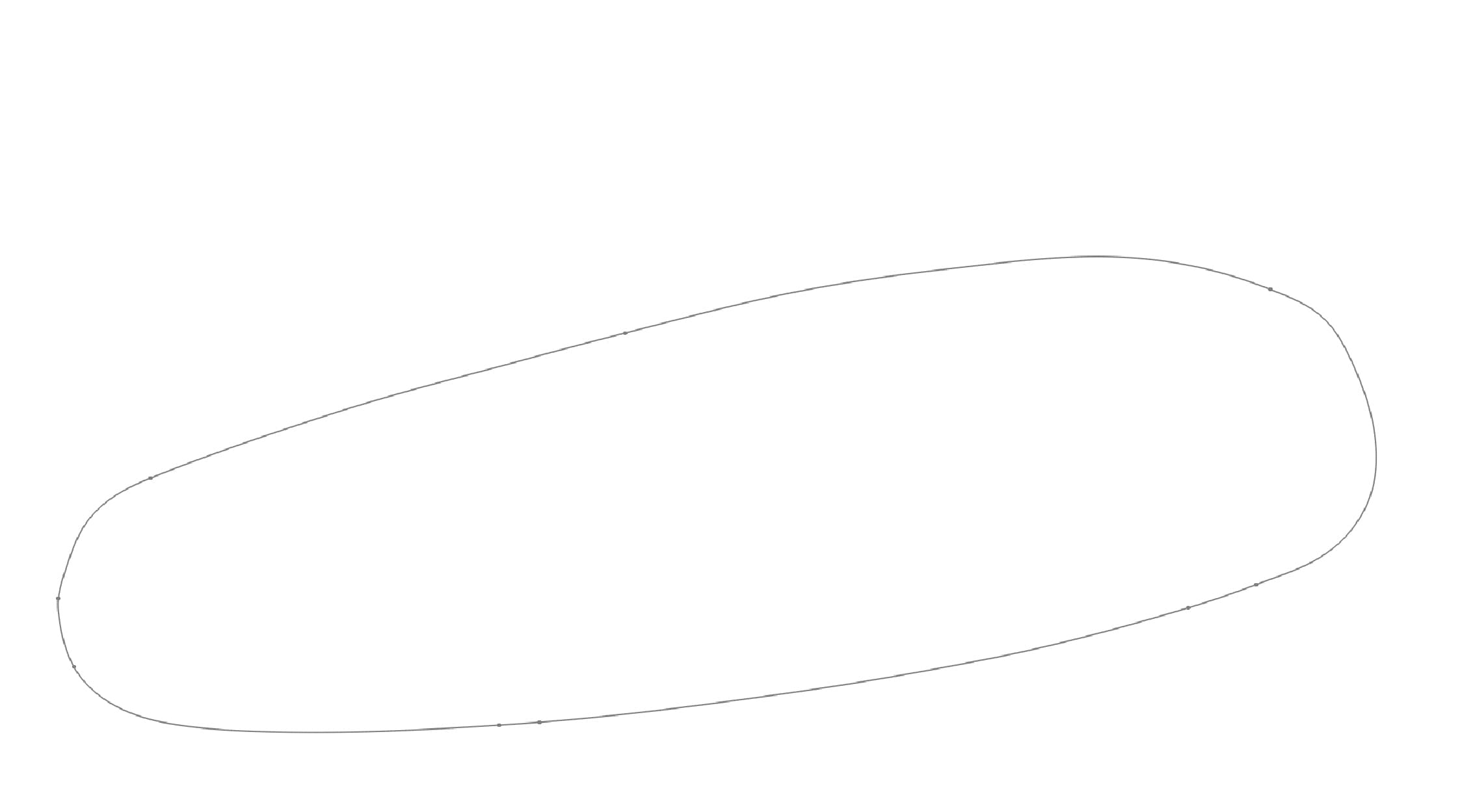

Supplement: Supplementary file 4 — Supporting Information [file ADVS-10-2203062-s013.zip › advs202203062-sup-0004-Supplementary-DataS3/Supplementary Data S3/183.jpg]

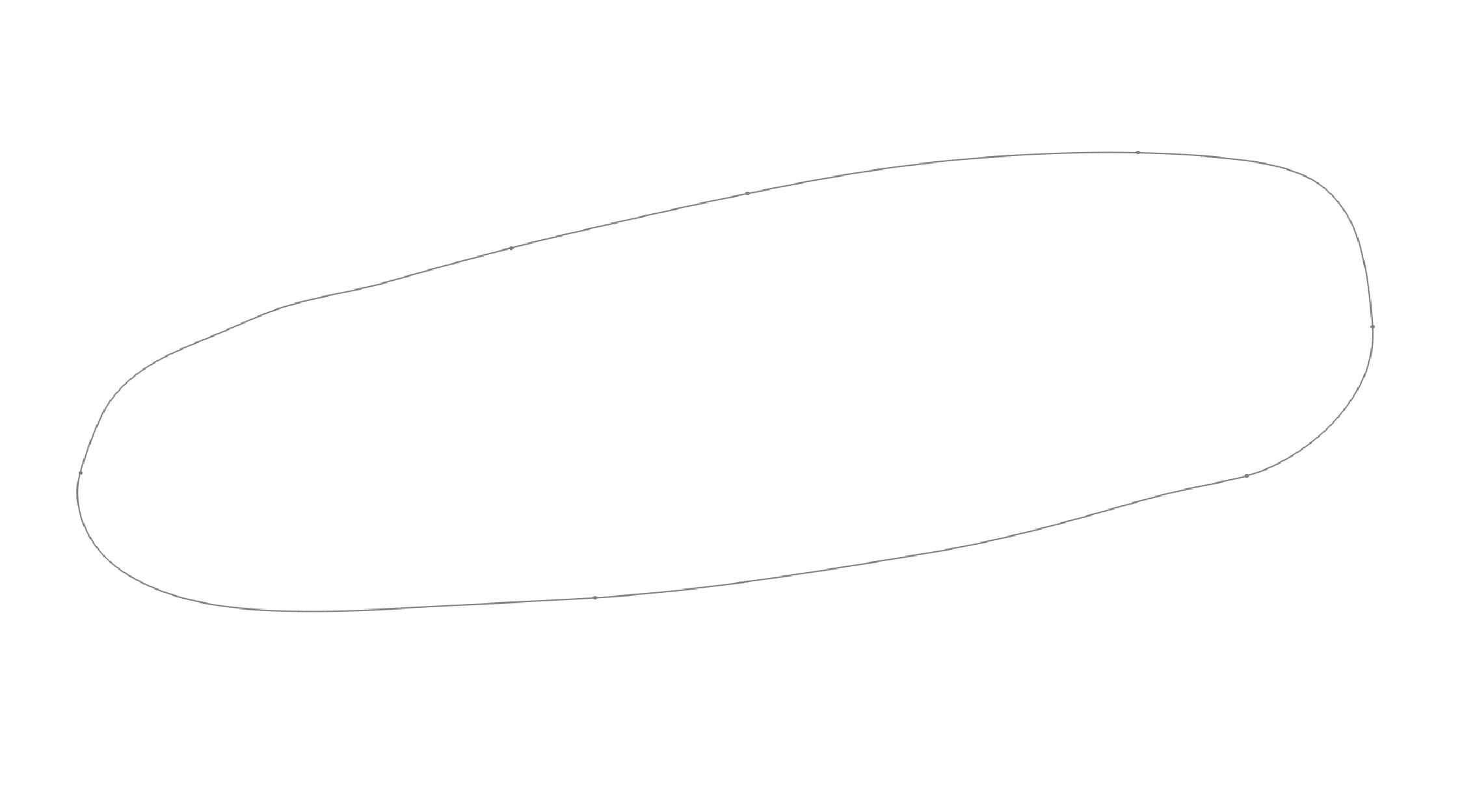

Supplement: Supplementary file 4 — Supporting Information [file ADVS-10-2203062-s013.zip › advs202203062-sup-0004-Supplementary-DataS3/Supplementary Data S3/184.jpg]

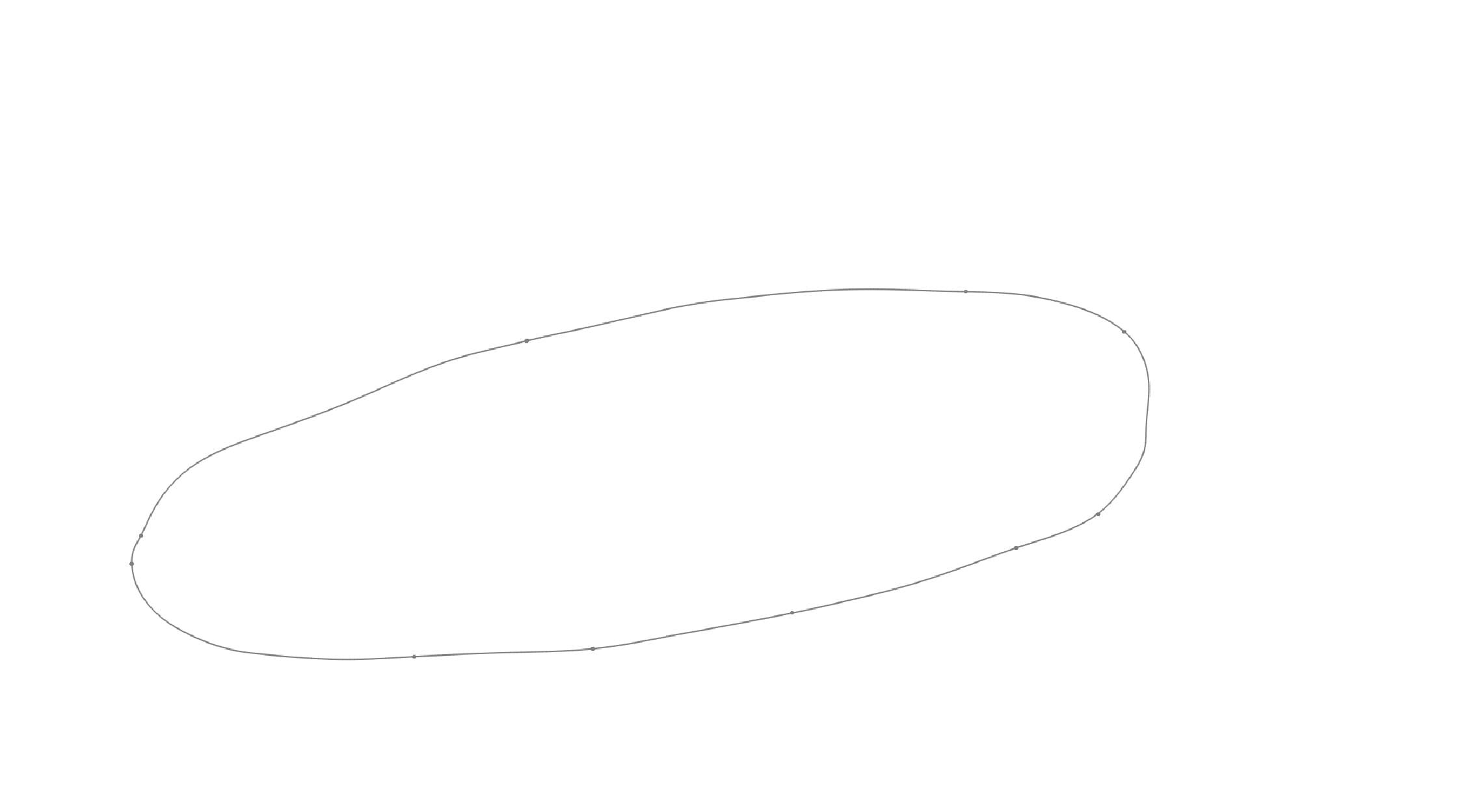

Supplement: Supplementary file 4 — Supporting Information [file ADVS-10-2203062-s013.zip › advs202203062-sup-0004-Supplementary-DataS3/Supplementary Data S3/185.jpg]

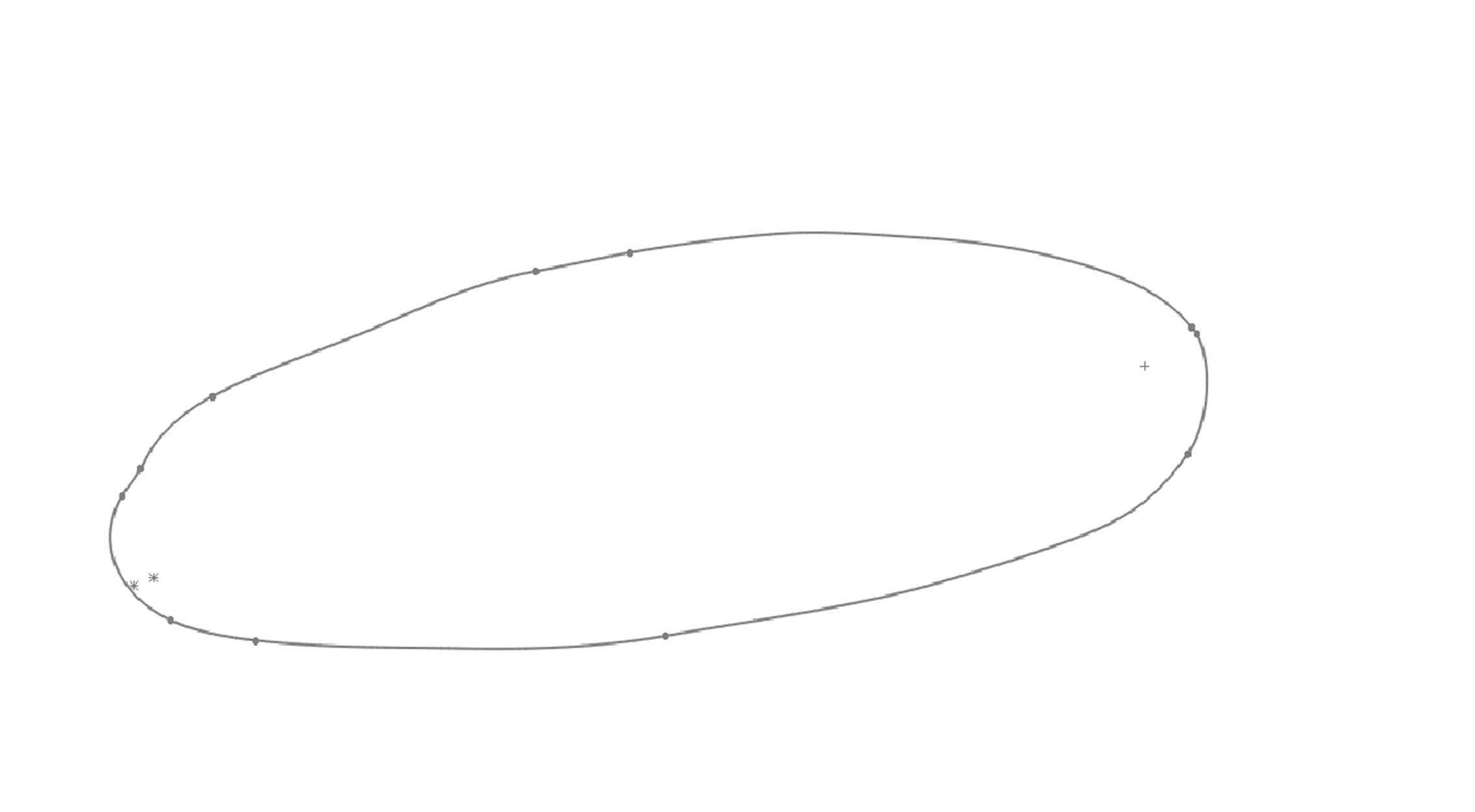

Supplement: Supplementary file 4 — Supporting Information [file ADVS-10-2203062-s013.zip › advs202203062-sup-0004-Supplementary-DataS3/Supplementary Data S3/186.jpg]

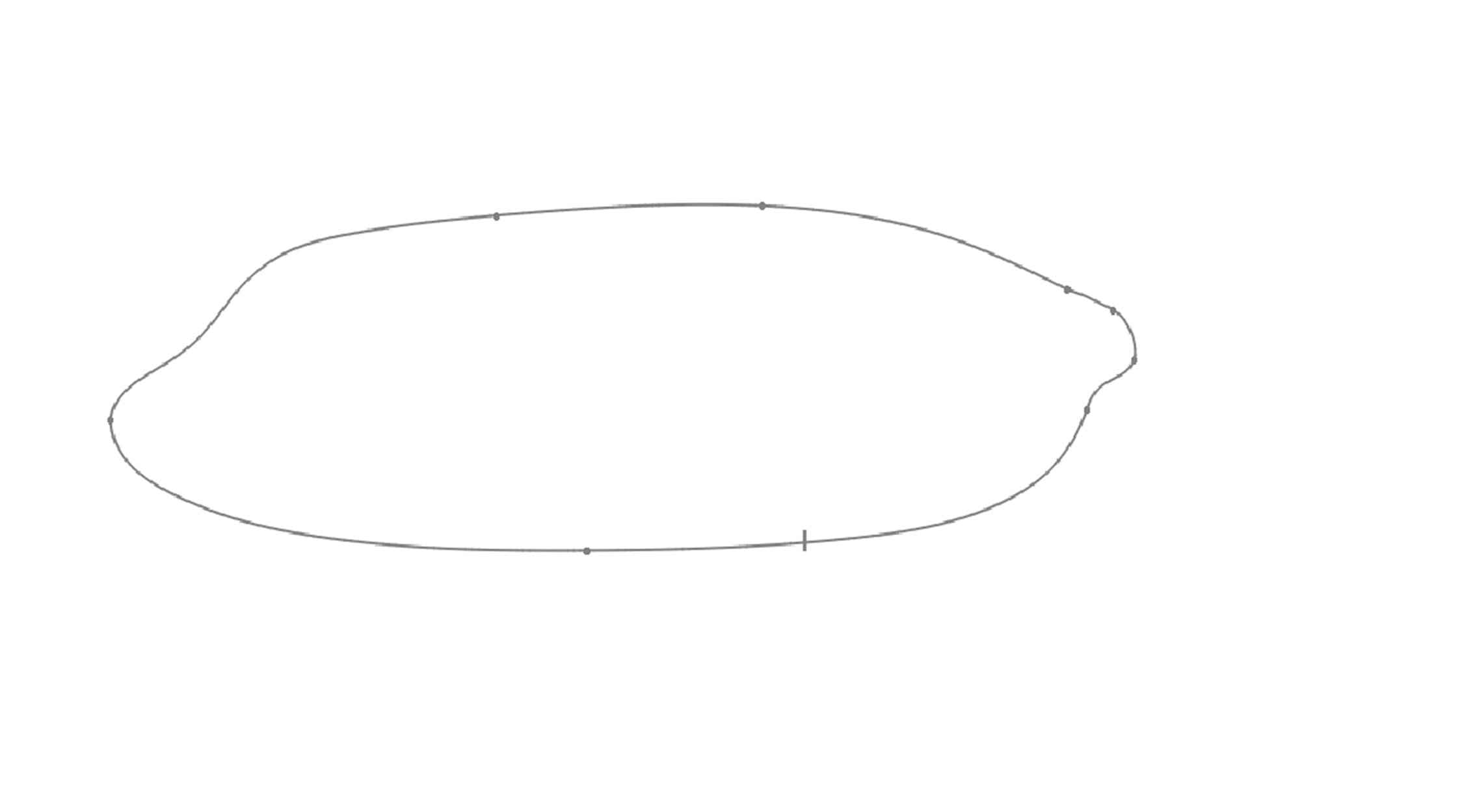

Supplement: Supplementary file 4 — Supporting Information [file ADVS-10-2203062-s013.zip › advs202203062-sup-0004-Supplementary-DataS3/Supplementary Data S3/187.jpg]

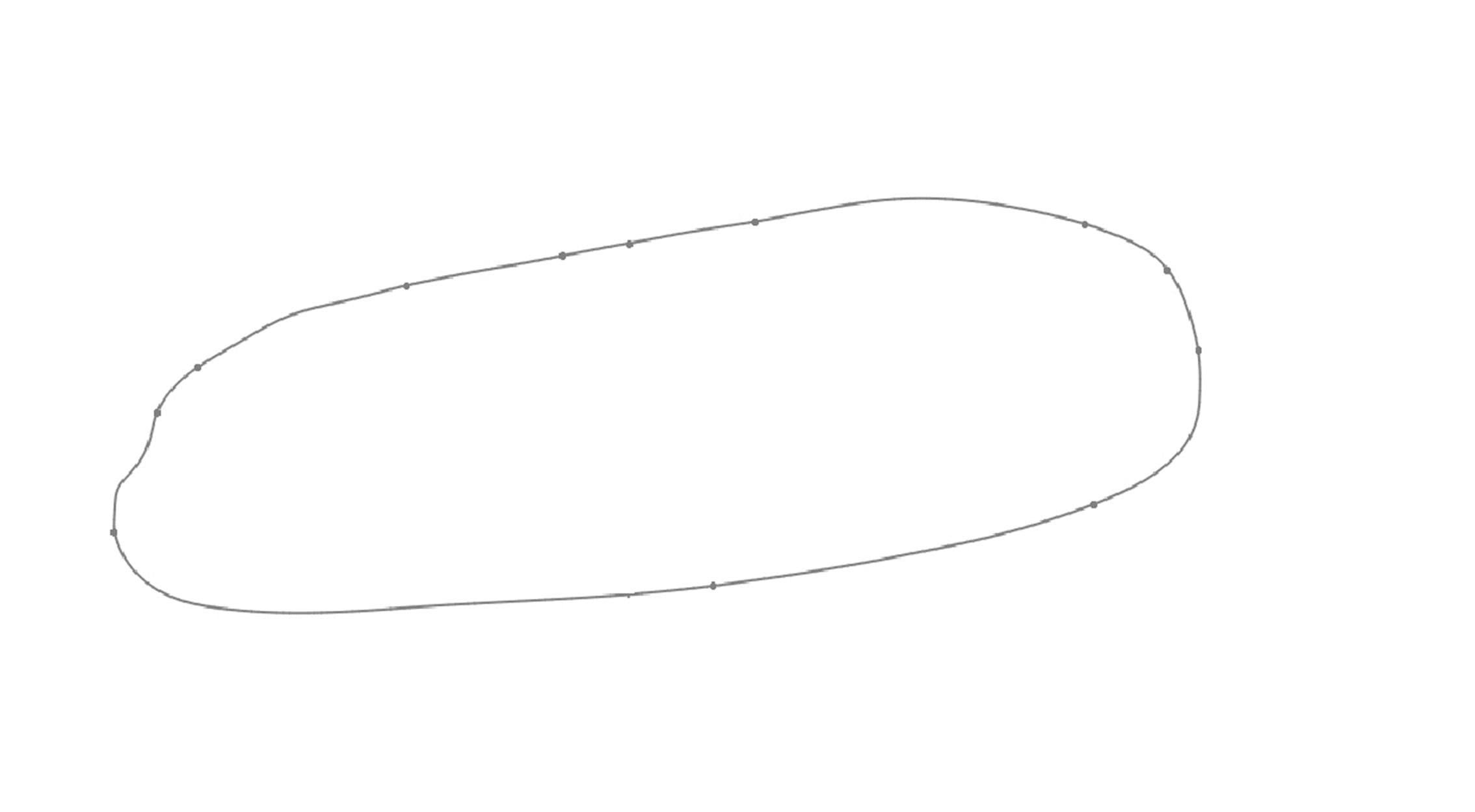

Supplement: Supplementary file 4 — Supporting Information [file ADVS-10-2203062-s013.zip › advs202203062-sup-0004-Supplementary-DataS3/Supplementary Data S3/188.jpg]

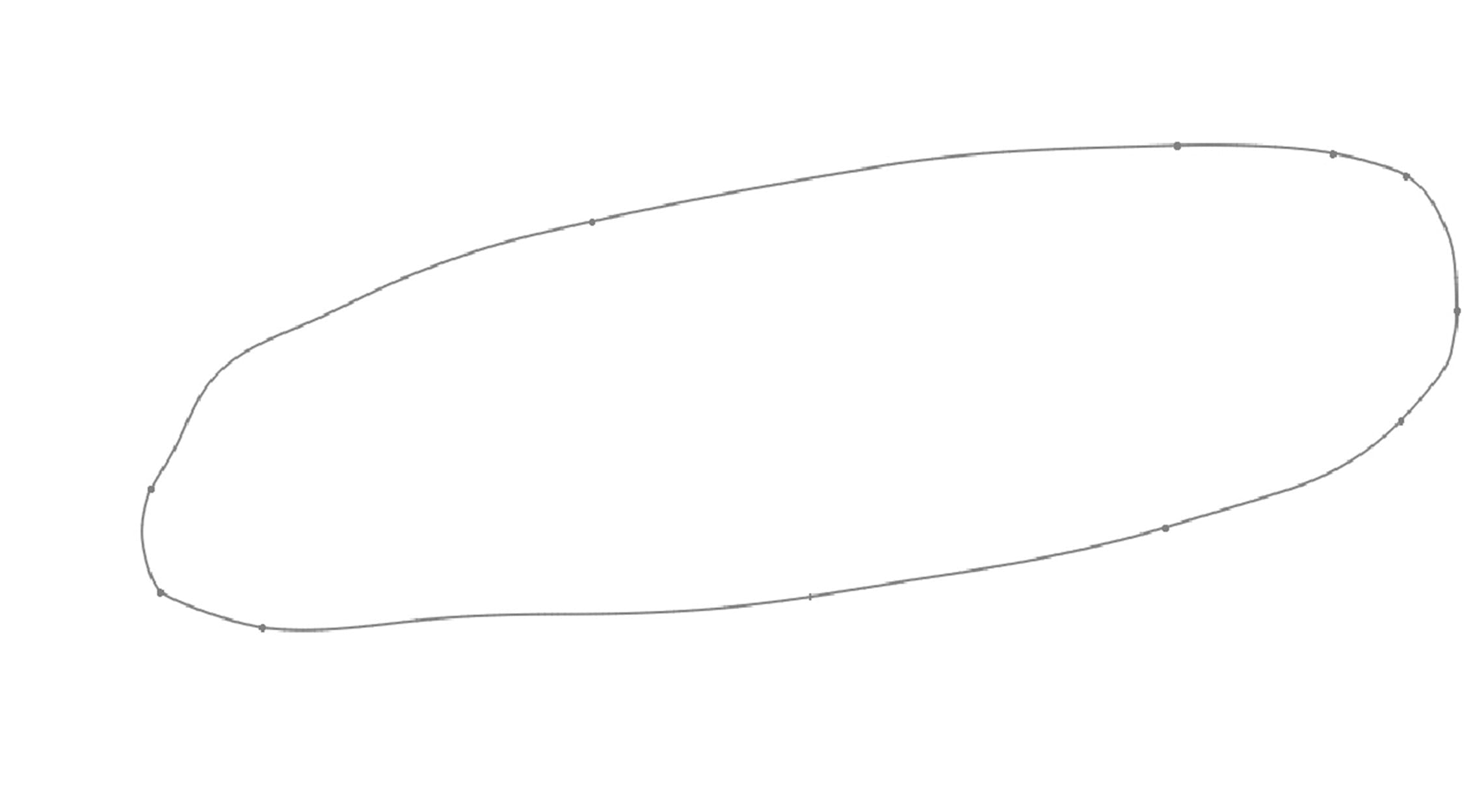

Supplement: Supplementary file 4 — Supporting Information [file ADVS-10-2203062-s013.zip › advs202203062-sup-0004-Supplementary-DataS3/Supplementary Data S3/189.jpg]
