# Supplementary material for: Wavy Whiskers in Wakes: Explaining the Trail‐Tracking Capabilities of Whisker Arrays on Seal Muzzles
Source: Adv Sci (Weinh). 2022 Nov 20;10(2):2203062. doi: 10.1002/advs.202203062 (PMC9839859; doi:10.1002/advs.202203062)
Supplement: Supplementary file 2 — Supporting Information [file ADVS-10-2203062-s009.zip › Supplementary Data S1/3D PDF files.zip!/3D PDF files/Constructed gray seal whisker.pdf]

SOLIDWORKS Educational Product. For Instructional Use Only.
